# Carbene-catalysed reductive coupling of nitrobenzyl bromides and activated ketones or imines via single-electron-transfer process

**DOI:** 10.1038/ncomms12933

**Published:** 2016-09-27

**Authors:** Bao-Sheng Li, Yuhuang Wang, Rupert S. J. Proctor, Yuexia Zhang, Richard D. Webster, Song Yang, Baoan Song, Yonggui Robin Chi

**Affiliations:** 1 Division of Chemistry & Biological Chemistry, Nanyang Technological University, School of Physical & Mathematical Sciences, Singapore 637371, Singapore; 2 Laboratory Breeding Base of Green Pesticide and Agricultural Bioengineering, Key Laboratory of Green Pesticide and Agricultural Bioengineering, Ministry of Education, Guizhou University, Huaxi District, Guiyang 550025, China

## Abstract

Benzyl bromides and related molecules are among the most common substrates in organic synthesis. They are typically used as electrophiles in nucleophilic substitution reactions. These molecules can also be activated via single-electron-transfer (SET) process for radical reactions. Representative recent progress includes α-carbon benzylation of ketones and aldehydes via photoredox catalysis. Here we disclose the generation of (nitro)benzyl radicals via *N*-heterocyclic carbene (NHC) catalysis under reductive conditions. The radical intermediates generated via NHC catalysis undergo formal 1,2-addition with ketones to eventually afford tertiary alcohol products. The overall process constitutes a formal polarity-inversion of benzyl bromide, allowing a direct coupling of two initially electrophilic carbons. Our study provides a new carbene-catalysed reaction mode that should enable unconventional transformation of (nitro)benzyl bromides under mild organocatalytic conditions.

## Abstract (web summary)

Benzyl bromides can be activated for radical reactions by photocatalytic single electron transfer. Here the authors show that *N*-heterocyclic carbenes are also capable of activating this pathway for nitrobenzyl bromides, and that the radical intermediates can add to the carbonyl group of ketones.

## Graphical abstract

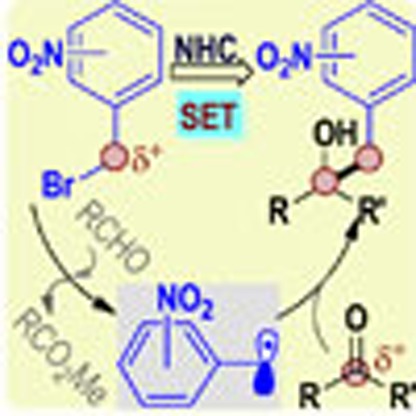

Alkyl halides, such as benzyl bromides, are readily available and inexpensive raw materials in chemical synthesis. Due to the electronegative nature of the halide atoms, these molecules are typically used as electrophiles to react with nucleophilic atoms in substitution reactions^1^,^2^. Alkyl halides can also be activated to form radical intermediates for various reactions. Indeed, the use of alkyl halides to generate radical intermediates^3^,^4^,^5^,^6^ for α-carbon alkylation of aldehydes^7^,^8^,^9^,^10^ and ketones^11^ has recently received intensive attention (Fig. 1a)^12^. For instance, MacMillan has pioneered the use of ruthenium photoredox catalysts to enable single-electron-transfer (SET) processes under light to form radicals that can then react with enamines catalytically generated from aldehydes and amine catalysts^7^,^8^. Melchiorre has realized asymmetric α-carbon alkylation of aldehydes via a radical process driven by the formation of a donor–acceptor complex and light^9^,^10^.

Our laboratory is interested in using *N*-heterocyclic carbine (NHC) organic catalysts to develop new reaction modes for rapid synthesis of functional molecules^13^,^14^. In our recent efforts in developing NHC-enabled radical reactions, we have found that the presence of a nitro group (in either substrates^15^ or reagents^16^) is important to initiate a SET process under NHC catalysis. Here we report that benzyl bromides, bearing a nitro substituent on the benzene ring, can undergo an NHC-mediated SET process using an aldehyde as a formal reductant (Fig. 1a). The catalytically generated benzyl radical intermediate then undergoes a formal 1,2-addition to a ketone to eventually form a tertiary alcohol product. The overall reaction is a formal reductive coupling of nitrobenzyl bromide **1** and ketone **2** (Fig. 1b).

Notably, the use of alkyl halides to react with another electrophile typically need to go through pre-formed organometallic reagents (such as Grignard reagents)^17^,^18^. Catalytic variants of the approaches with organometallic reagents have also been developed for the coupling of alkyl halides with another electrophilic substrate^19^,^20^,^21^. However, sensitive functional groups such as nitro and ketone substituents often cannot survive under conditions with organometallic reagents. In the area of organic catalysis, NHCs have mainly been used in electron-pair-transfer reactions^22^,^23^,^24^,^25^,^26^,^27^,^28^,^29^. Despite the fact that *vitamin B1* (an NHC precursor)-mediated reactions in biological systems go through SET processes^30^, NHC-mediated radical reactions in synthetic chemistry are much less developed. Studer^31^ reported the oxidation of aldehydes to carboxylic esters using TEMPO as a single-electron oxidant via NHC catalysis. We developed the dimerisation of nitroalkenes via NHC-mediated generation of radical anion intermediates from nitroalkenes^15^. Rovis'^32^ and our^16^ laboratories independently reported β-hydroxylation of unsaturated aldehydes through SET processes^33^ under NHC catalysis. Our present study in catalytically turning (nitro)benzyl bromides to radical intermediates for unusual reaction offers a new reaction mode of NHC catalysis that should provide unique opportunities in chemical synthesis.

## Results

### Reaction optimization

Key results of initial studies are summarized in Table 1 (the detailed conditions please see the Supplementary Table 1). The 4-nitrobenzyl bromide **1a**, and α,β-unsaturated ketoester **2a** were used as model substrates. Optimal conditions for the formation of alcohol product **4a** (in 75% isolated yield) were found when triazolium salt **A**^34^ was used as an NHC precursor, aryl aldehyde **3a** as a reductant, and *N,N*-diisopropylethylamine as a base (entry 1). The reactions under darkness (the reaction flask was covered with aluminium foil) or normal room lighting conditions gave nearly the same outcomes, indicating that the generation of radical intermediates under our system was not driven by light. Both the NHC catalyst (entry 2) and the aryl aldehyde reductant (entry 3) were required for the reductive coupling reaction to proceed. The nitro group in substrate **1a** was critical for this reaction to occur, and its roval or replacement with other substituents (for example, **1b**, **1c** or **1d**) led to no detectable formation of **4a** (entry 4). The substituents on the aryl aldehyde reductant could also affect the reaction efficiency (entries 5, 6). When aryl aldehyde such as **3b** and **3c** were used as the reductant, **4a** was formed in much lower yield. In these cases (entries 4–6), most of the benzyl bromide and ketoester substrates remained unreacted, and the aryl aldehyde substrate was consumed to form benzoin adducts and the corresponding carboxylic ester. The *N*-aryl substituents in the triazolium-based NHC pre-catalysts (entries 1, 7 and 8) could affect the reaction outcomes. Imidazolium NHC pre-catalyst **D** could also mediate this reaction, albeit with a low yield (23%, entry 9). A thiazolium pre-catalyst (**E**) could not mediate this reaction (entry 10). When catalyst **B** or **C** were used, a significant amount of benzoin condensation products of the aldehyde were observed (yields of benzoin adduct:<5% with catalyst **A**; 45% with catalyst **B**; 40% with catalyst **C**).

### Substrates scope with α-keto esters

**Figure 2:**
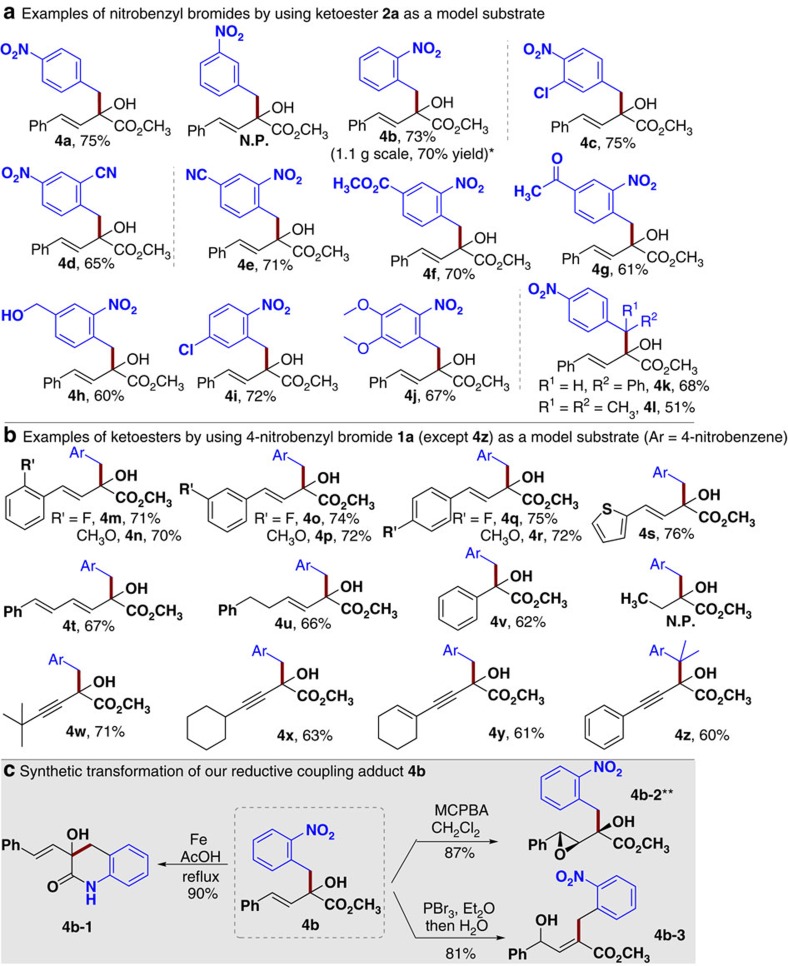
α-Ketoester as substrates and its transformations. Scope of the reaction (**a**,**b**) and synthetic transformation of the reductive coupling product (**c**). The reactions were performed on 0.1 mmol scale (condition as in Table 1, entry 1). Isolated yields base on ketoesters **2**. *The reaction was carried out at 5 mmol (ketoester) scale. **Relative configuration. MCPBA, 3-chloroperoxybenzoic acid.

With an acceptable condition in hand, we examined the scope of the reductive coupling reaction (Fig. 2). Examples of nitrobenzyl bromides that could react effectively (with ketoester **2a** as a model ketone substrate) are given in Fig. 2a. The use of benzyl bromides with the NO_2_ group at the para (**4a**) or ortho (**4b**) position of the benzene ring both worked well, while meta-nitro benzyl bromide could not react to give the proposed reductive coupling product. Placing additional substituents on the para-nitro benzyl bromides (**4c**, **4d**) and ortho-nitro benzyl bromides (**4e**–**j**) were all tolerated. For example, having an ester (**4f**) or ketone (**4g)** moiety on the ortho-nitrobenzyl bromide did not affect the reaction outcomes. It should be noted that such electrophilic functional groups (for example, esters, ketones) are likely problematic in conventional approaches using organometallic reagents^17^,^18^. Secondary and tertiary nitrobenzyl bromides (**4k**, **4l**) are also effective substrates. Examples of ketoester substrates are given in Fig. 2b. Different substituents and substitution patterns on the phenyl ring of unsaturated ketoester **2a** were tolerated (**4m**–**r)**. The phenyl unit of **2a** could be replaced with a thiofuran (**4s**), alkene (**4t**) or alkyl (**4u**) unit. Phenyl ketoester **4v** could effectively react with nitrobenzyl bromide **1a**, while the use of alkyl ketoesters (such as methyl 2-oxobutanoate) did not lead to the proposed products. The carbon–carbon double bond of unsaturated ketoester **2a** could be replaced with a carbon-carbon triple bond (**4w**–**z**), with little influence on the reaction yields. The reaction, carried out under mild conditions, can be easily scaled up. For example, product **4b** could be prepared in gram scale without loss in yield.

The reductive coupling adduct from our reactions can be readily converted to other functional molecules (Fig. 2c). For example, reduction of the NO_2_ group in **4b** followed by an intramolecular ester-to-amide exchange could give lactam **4b-1** that bears a quinolone core widely found in natural products and bioactive molecules^35^. The alkene in allylic alcohol **4b** could be easily converted to epoxide **4b-2** in 87% yield with excellent stereo-selectivity (only one diastereoisomer was obtained). The hydroxyl group in **4b** could undergo a formal 1,3-migration under acidic conditions to form γ-hydroxyl α, β-unsaturated ester **4b-3** in 81% yield.

### Substrates scope with isatins

**Figure 3:**
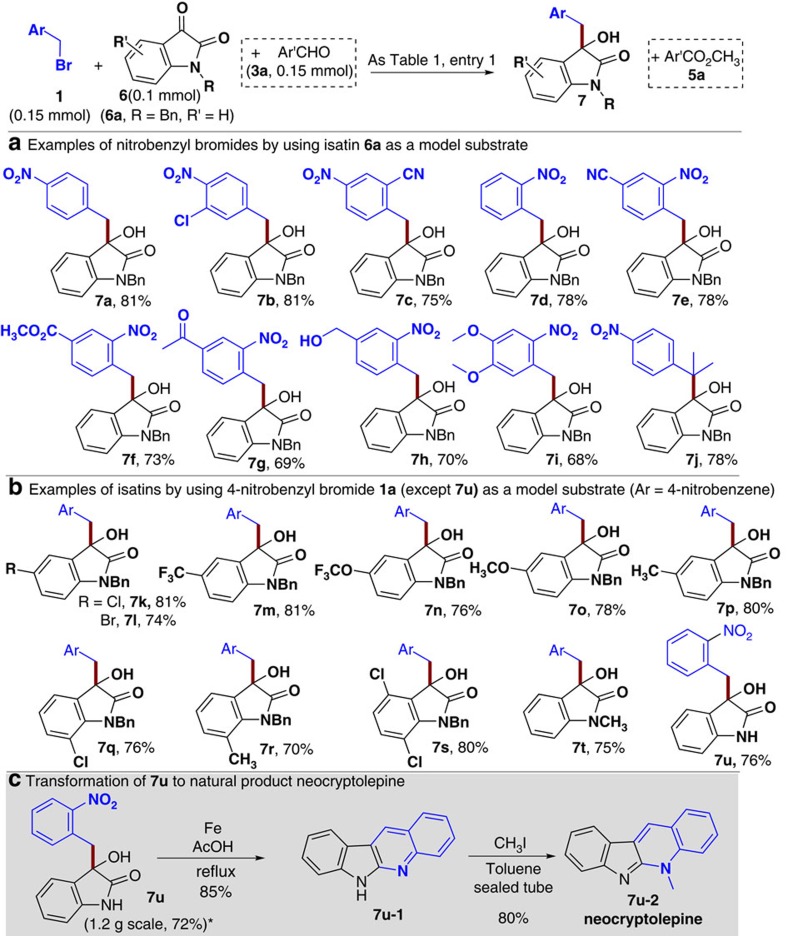
Isatin as substrates and its application. Isatin as the substrates (**a**,**b**), and transformation of 7u to natural product (**c**). The condition as in Table 1, entry 1. Isolated yields base on isatin (**6**). * The reaction was carried out at 5 mmol (ketoester) scale.

To further expand the scope of our reductive coupling reactions, we next examined isatin derivatives as ketone substrates to react with the nitrobenzyl bromides (Fig. 3). To our delight, the standard conditions used above for the ketoester substrates (Table 1, entry 1) worked effectively for the isatin substrates without any further optimization. The reaction yields here were slightly higher than those reactions using ketoesters. Nitrobenzyl bromides with different substituents and substitution patterns were well tolerated, giving the desired alcohol products in 68–81% yields (**7a–j**, Fig. 3a). The substituents on the isatin had little effect on the reaction yields (**7k–t**, Fig. 3b). The isatin without an *N*-substituent (**7u**) reacted effectively as well. Further synthetic transformations of the reductive coupling products can provide a shortcut to functional molecules (Fig. 3c). For example, reduction and cyclization of **7u** followed by a facile methylation gave neocyptoplepine **7u-2**, a natural product isolated from the bark of the roots of *Cryptolepis sanguinolenta*^36^,^37^. Neocyptoplepine and its analogs exhibit a strong antiplasmodial activity against *Plasmodium falciparum* chloroquine-resistant strains^38^.

### Substrates scope with imines

**Figure 4:**
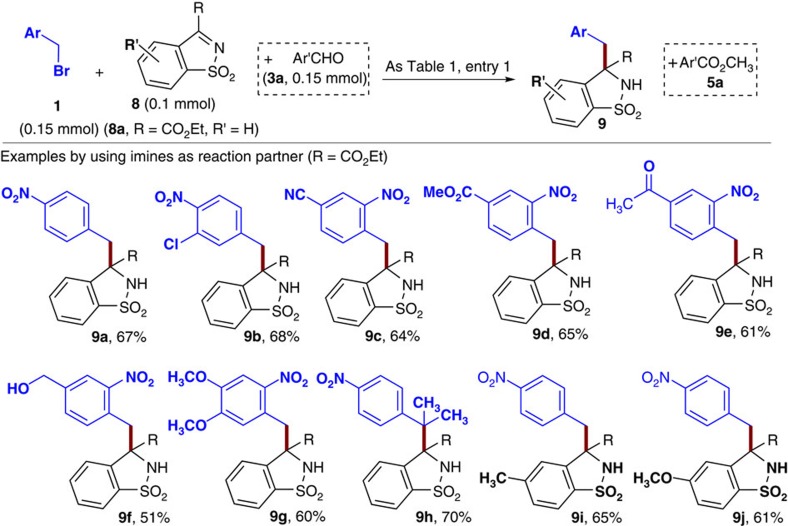
Imines as the substrates. The condition as in Table 1, entry 1. Isolated yields base on imines (**8**).

Imines as reaction partners also worked effectively under our catalytic condition (Fig. 4). The results further illustrated the broad applicability of our NHC-mediated SET approach.

## Discussion

**Figure 5:**
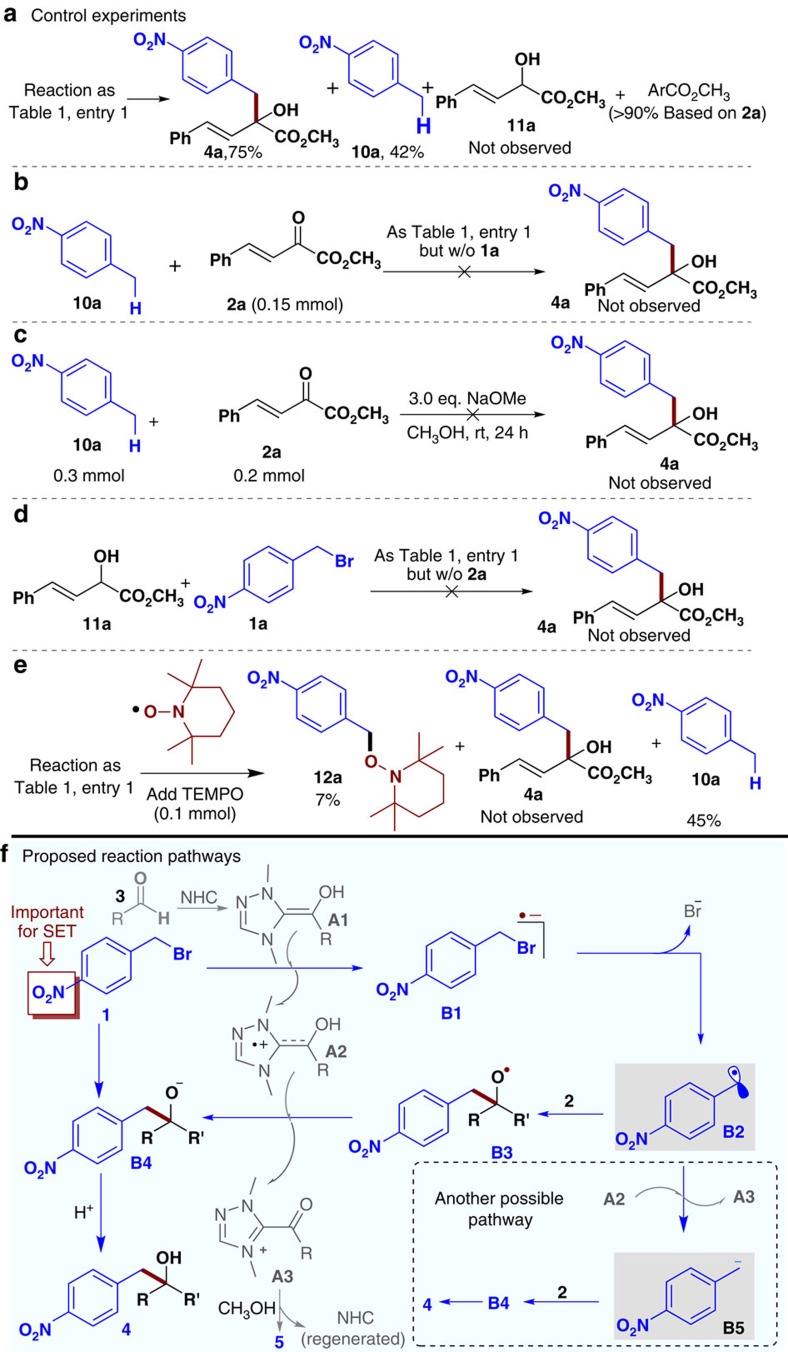
Experiments to elucidate the reaction mechanism and possible reaction pathways. (**a**) The experiment indicates that the α-ketoester has no effect for the generation of nitrobenzyl radical. (**b**) The result suggest that the 1,2-addition product unlikely come from 4-nitro toluene in the current reaction system. (**c**) The coupling product could not be produced in the presence of NaOMe. (**d**) The final product could not be obtained via the alkylation of a-hydroxyl ester. (**e**) The result indicates that the nitro benzyl radical is existent in current reaction system. (**f**) The nitrobenzyl radical **B2** could be formed via oxidation of Breslow intermediate **A1** and leaving of bromide anion from **B1**. Subsequently, the carbon-carbon bond can be formed via 1,2-addition of nitrobenzyl radical intermediate **B3** or ionic intermediate **B5** to carbonyl compounds.

To understand the reaction pathway (as postulated in Fig. 5f), we analysed the side reactions and performed multiple control experiments (Fig. 5, additional control experiments see Supplementary Methods). Under the standard conditions (Table 1, entry 1), a debromination product (**10a**) of nitrobenzyl bromide **1a** was observed in 42% yield (Fig. 5a; the yield of **10a** is based on ketone ester **2a**-the limiting reagent). This side product (**10a**) likely come from a further reduction of nitrobenzyl radical intermediate **B2** (Fig. 5a). Specifically, the reduction of **B2** formed a nitrobenzyl anion that underwent spontaneous protonation in CH_3_OH as the solvent and proton source to form **10a**. The nitrotoluene substrate **10a** could not react with our ketone substrate **2a** (Fig. 5b,c), suggesting that the formation of **4a** in our catalytic reaction unlikely came from a nucleophilic addition of nitrobenzyl anion to the ketone substrate. A possible reduction^39^ of the ketone substrate **2a** to form α-hydroxyl ester **11a** was not observed. To rule out the possibility that **9a** was formed *in situ* and quickly reacted with the bromide substrate **1a**. We prepared **11a** and evaluated its reaction with **1a** (Fig. 5d). No formation of **4a** was observed under various conditions (Fig. 5d).

We have also measured the reduction potential of compounds **1a**, **2a** and **6a** *via* cyclic voltammetry (see Supplementary Method and Supplementary Fig. 130). The experiments showed that compound **1a** (*E*_p_^red^=−1.18 V vs) has a higher reduction potential than compounds **2a** (*E*_p_^red^=−1.47 V vs) and **6a** (*E*_p_^red^=−1.38 V vs). The results suggested that most likely **1a** is easier to be reduced, supporting our mechanistic proposal (Fig. 5f) that the SET process first occurs on nitrobenzyl bromide **1a**. Finally, the addition of radical trapping agent 2,2,6,6-tetramethyl-1-piperidinyloxy (TEMPO) to our reaction diminished the formation of **4a** (Fig. 5e). A radical–radical coupling adduct **12a** between TEMPO and nitrobenzyl radical **B2** was observed in 7% yield (Fig. 5e). Similar observations in trapping of benzyl radical intermediates with TEMPO under photocatalysis were reported by Melchiorre^9^ and Meggers^11^, respectively.

A postulated reaction pathway is illustrated in Fig. 5f. Aldehyde reagent **3** reacts with the NHC catalyst to form a Breslow intermediate^40^ **A1** that then serves as a single-electron reductant to convert nitrobenzyl bromide **1a** into radical anion intermediate **B1**. Breslow intermediate **A1** is oxidized to a radical cation intermediate^31^ **A2** in this process. The leaving of a bromide anion (Br^−^) from **B1** affords nitrobenzyl radical intermediate **B2**. On the one hand, addition of radical intermediate **B2** to ketone^41^,^42^,^43^,^44^ substrate **2** forms radical intermediate **B3**. Subsequent reduction of **B3** by Breslow intermediate-derived radical cation **A2** forms **B4**, which can then be protonated to form alcohol product **4**. The oxidized **A2** becomes azolium ester intermediate **A3** that can then be trapped by CH_3_OH to form ester **5** with regeneration of the NHC catalyst. Alternatively, reduction of radical intermediate **B2** to form intermediate **B5** with an anionic benzylic carbon may occur. Nucleophilic addition of **B5** to ketone substrate **2** can also lead to product **4**. At this point, this ionic pathway (via intermediate **B5**) cannot be ruled out. In the overall process, the initially electrophilic nitrobenzyl bromide is catalytically activated to directly react with another electrophilic substrate (the ketone substrate).

In summary, we have developed a new organic catalytic method for the generation of nitrobenzyl radical intermediates from nitrobenzyl bromides under mild conditions. The catalytically formed radical intermediates react with ketones, resulting in a formal reductive coupling of initially electrophilic nitrobenzyl bromides and ketones. The products from our catalytic reactions can be readily transformed to functional molecules and natural products. Our study provides a new reaction mode that can likely allow for unique design in organic synthesis. It may also encourage further adventures in developing novel organocatalytic activations by single-electron-transfer processes. Mechanistic studies and additional development of this reaction mode for challenging substrates are in progress.

## Methods

### General strategy of reduce coupling reactions

To a reaction tube equipped with a stirrer bar were added NHC catalyst salt **A** (0.005 mmol), aldehyde **3** (0.15 mmol), ketone (**2, 6** or **8**, 0.1 mmol), and benzyl bromide **1** (0.15 mmol). The tube was sealed, evacuated and purged, and a solution of *N,N*-disopropylethylamine (0.15 mmol, 19.4 mg, 25 μl) in methanol (1.0 ml) was added. The reaction was stirred for 8 h. The solvent was removed *in vacuo* and the residue was purified by flash column chromatography.

### Data availability

For ^1^H, ^13^C NMR and high-performance liquid chromatography spectra of compounds in this manuscript, see Supplementary Figs 1–129. For Oak Ridge thermal ellipsoid plot (ORTEP) of products **4w** and **7l**, see Supplementary Information. The X-ray crystallographic coordinates for structures reported in this article have been deposited at the Cambridge Crystallographic Data Centre (**4w:** CCDC 1451838; **7l:** CCDC 1451840). These data could be obtained free of charge from The Cambridge Crystallographic Data Centre via www.ccdc.cam.ac.uk/data_request/cif.

## Additional information

**How to cite this article:** Li, B.-S. *et al*. Carbene-catalysed reductive coupling of nitrobenzyl bromides and activated ketones or imines via single-electron-transfer process. *Nat. Commun.* **7,** 12933 doi: 10.1038/ncomms12933 (2016).

## Supplementary Material

Supplementary Information Supplementary Figures 1-131, Supplementary Table 1, Supplementary Methods and Supplementary References

## Figures and Tables

**Figure 1:**
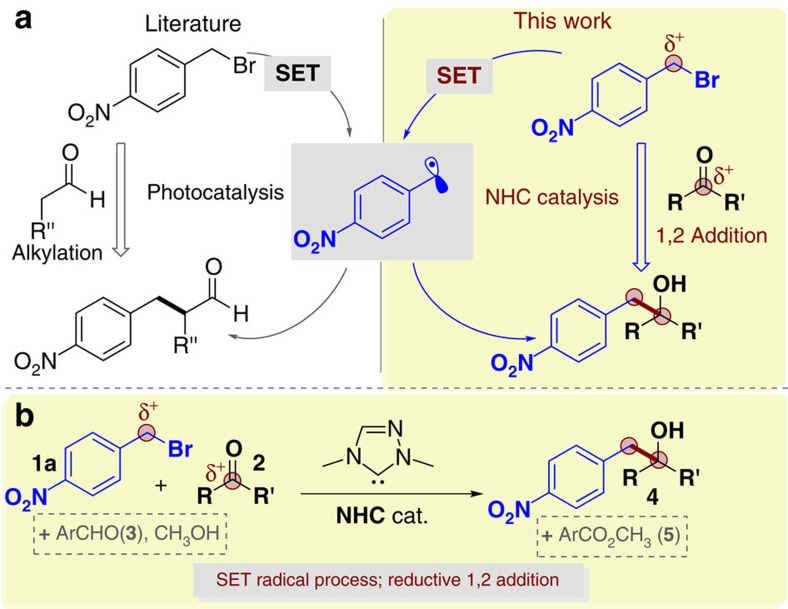
NHC-catalysed reduction coupling of nitrobenzyl bromide with activated carbonyl compounds. (**a**) Formation of benzyl radical via photoredox or NHC catalysis. (**b**) Reductive coupling of nitrobenzyl bromides and ketones.

**Table 1:**
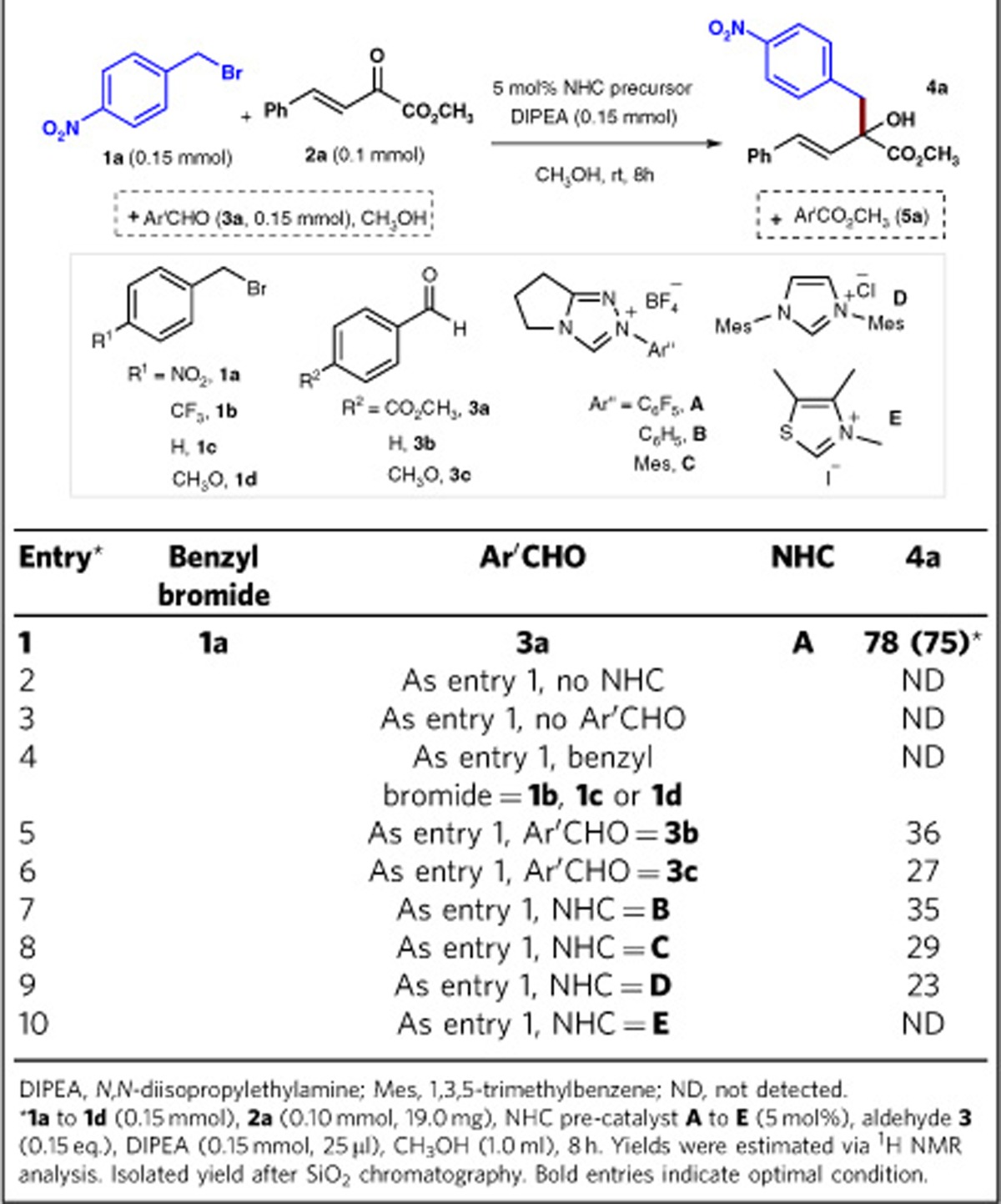
Key results of condition optimizations.
