# Supplementary material for: Carbene-catalysed reductive coupling of nitrobenzyl bromides and activated ketones or imines via single-electron-transfer process
Source: Nat Commun. 2016 Sep 27;7:12933. doi: 10.1038/ncomms12933 (PMC5052654; doi:10.1038/ncomms12933)
Supplement: Supplementary Information — Supplementary Figures 1-131, Supplementary Table 1, Supplementary Methods and Supplementary References [file ncomms12933-s1.pdf]

Supplementary Figures

Supplementary Figure 1. ^{1}H NMR Spectrum of substrate 4a

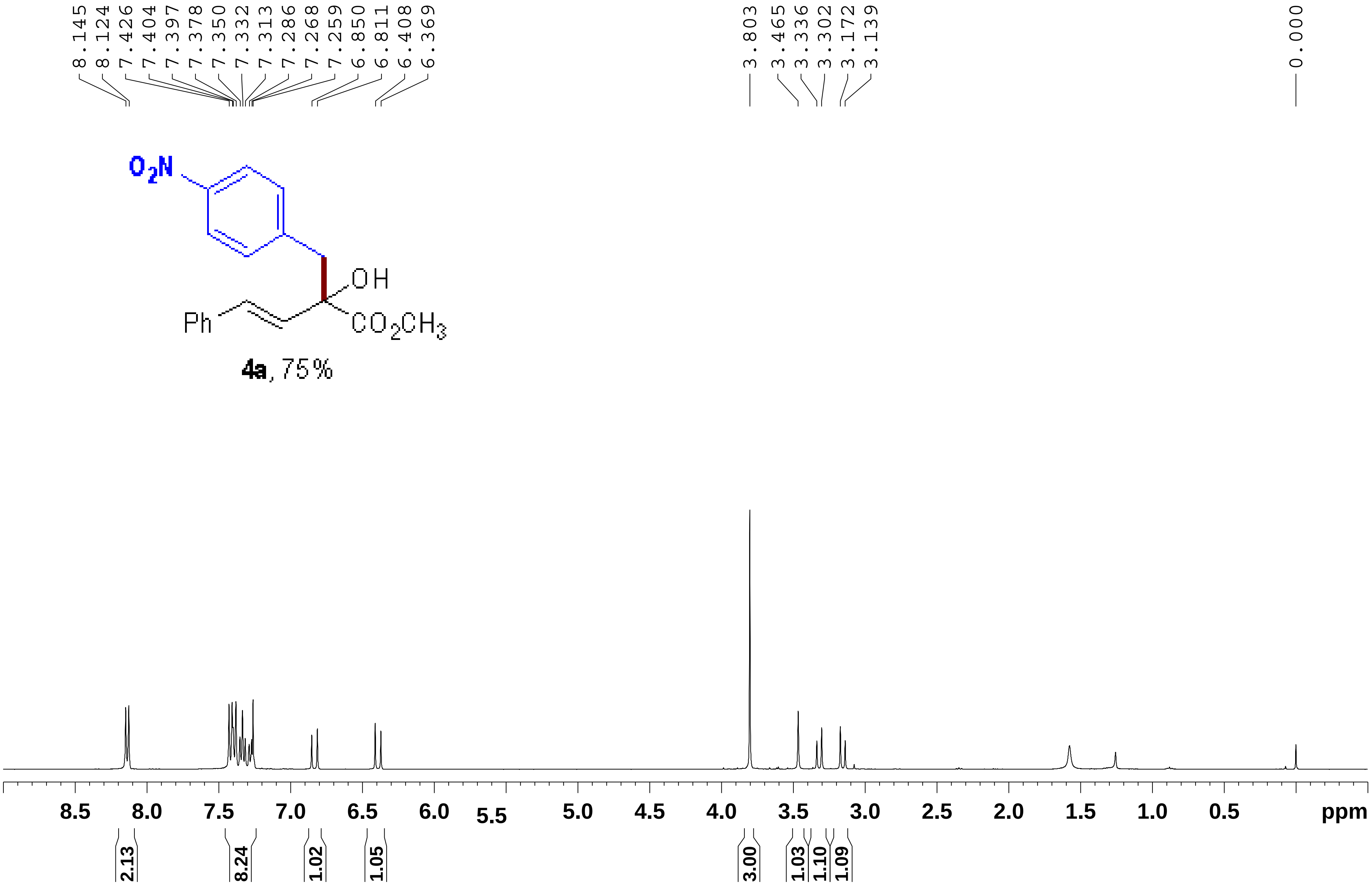

Supplementary Figure 2. ^{13}**C NMR Spectrum of substrate 4a**

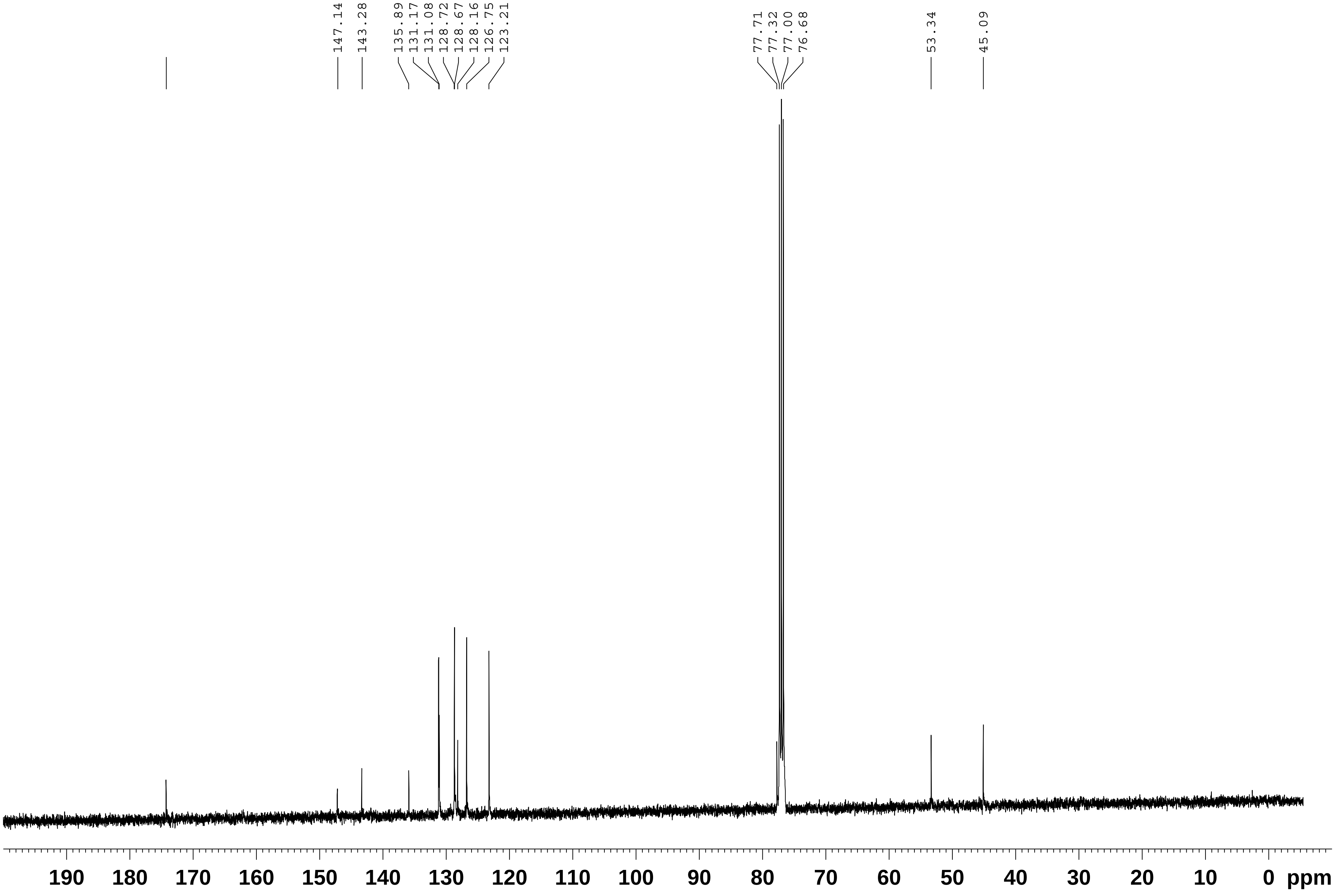

Supplementary Figure 3. ^{1}H NMR Spectrum of substrate 4b

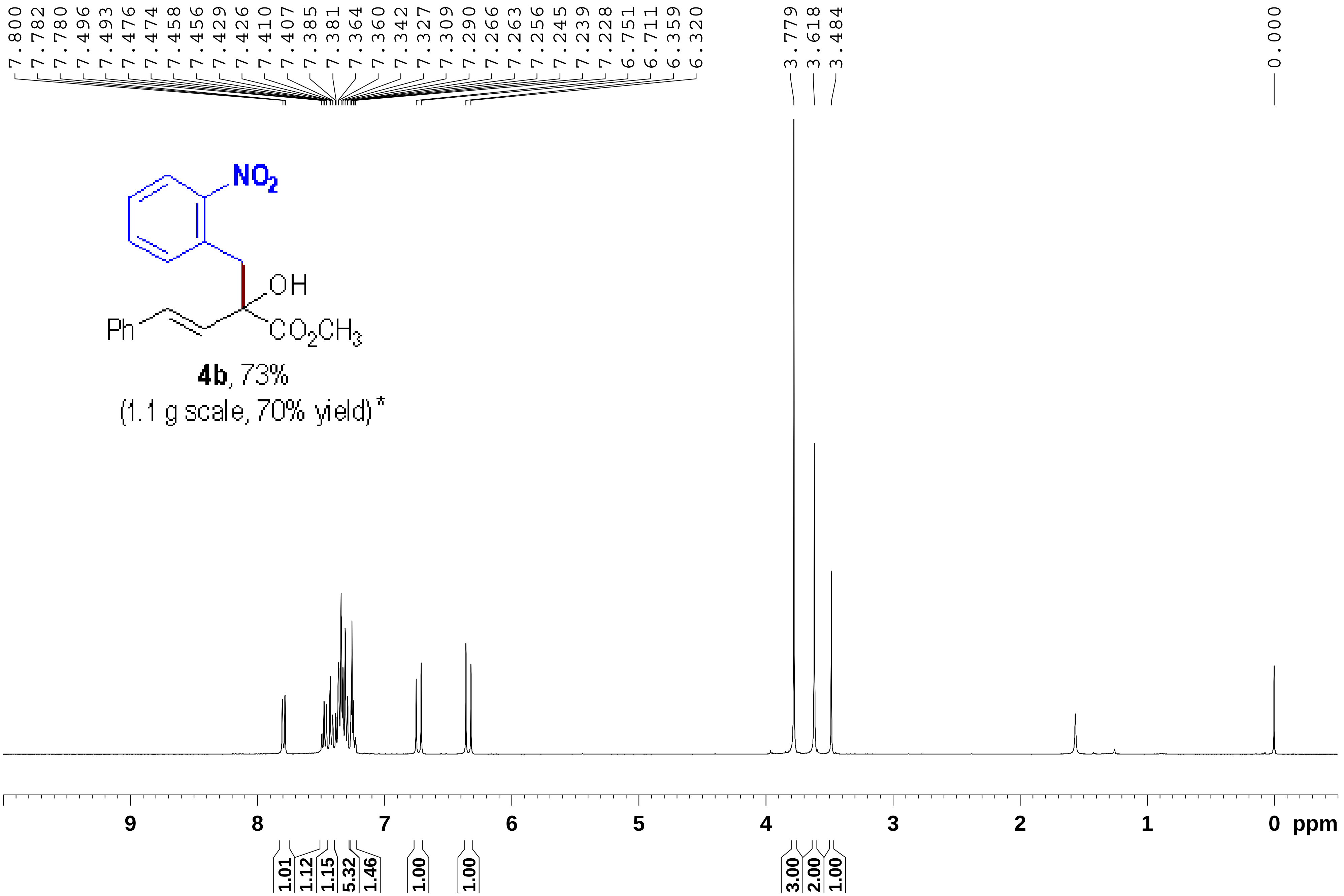

Supplementary Figure 4. ^{13}C NMR Spectrum of substrate 4b

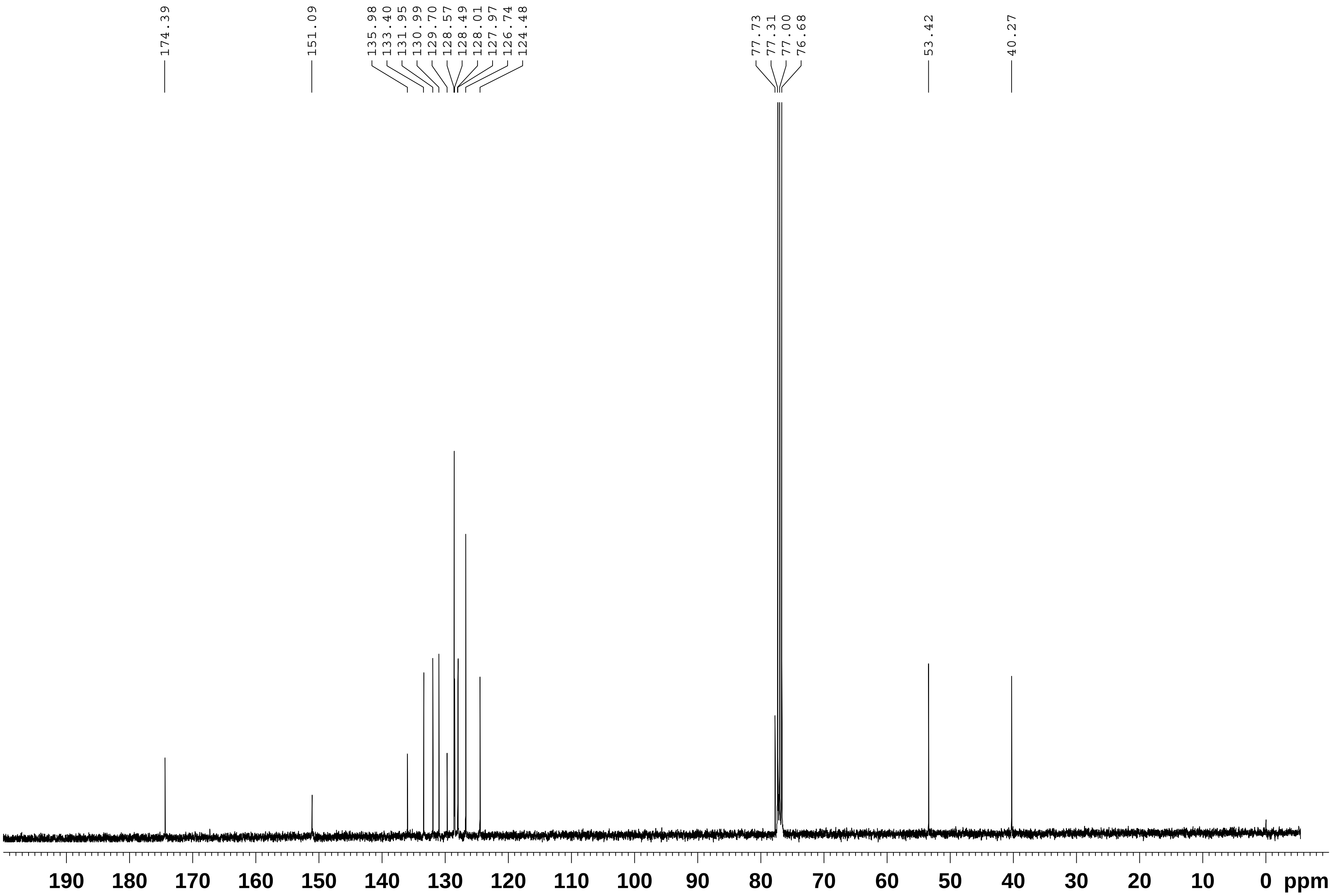

Supplementary Figure 5. ^{1}H NMR Spectrum of substrate 4c

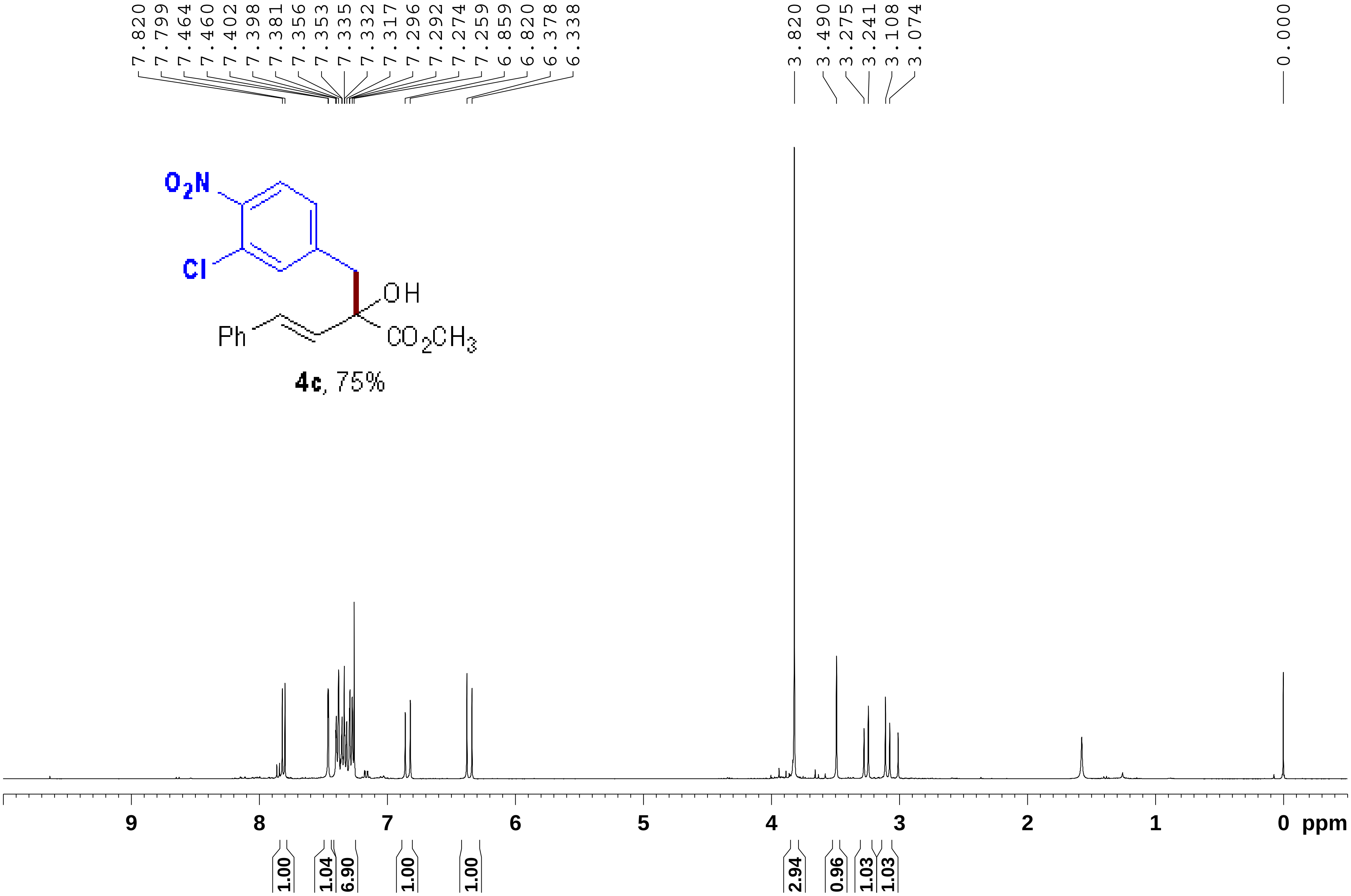

Supplementary Figure 6. ^{13}C NMR Spectrum of substrate 4c

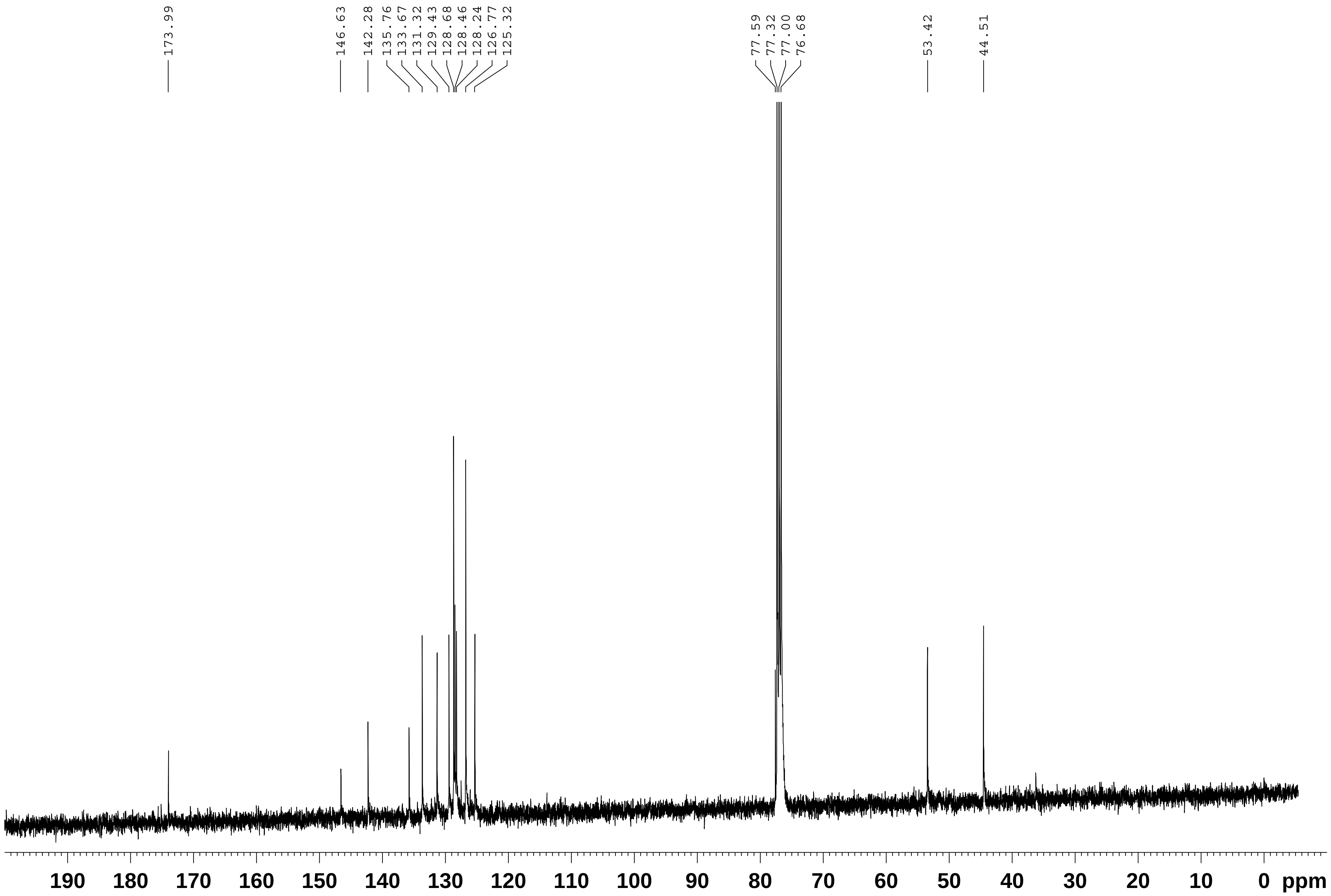

Supplementary Figure 7. ^{1}H NMR Spectrum of substrate 4d

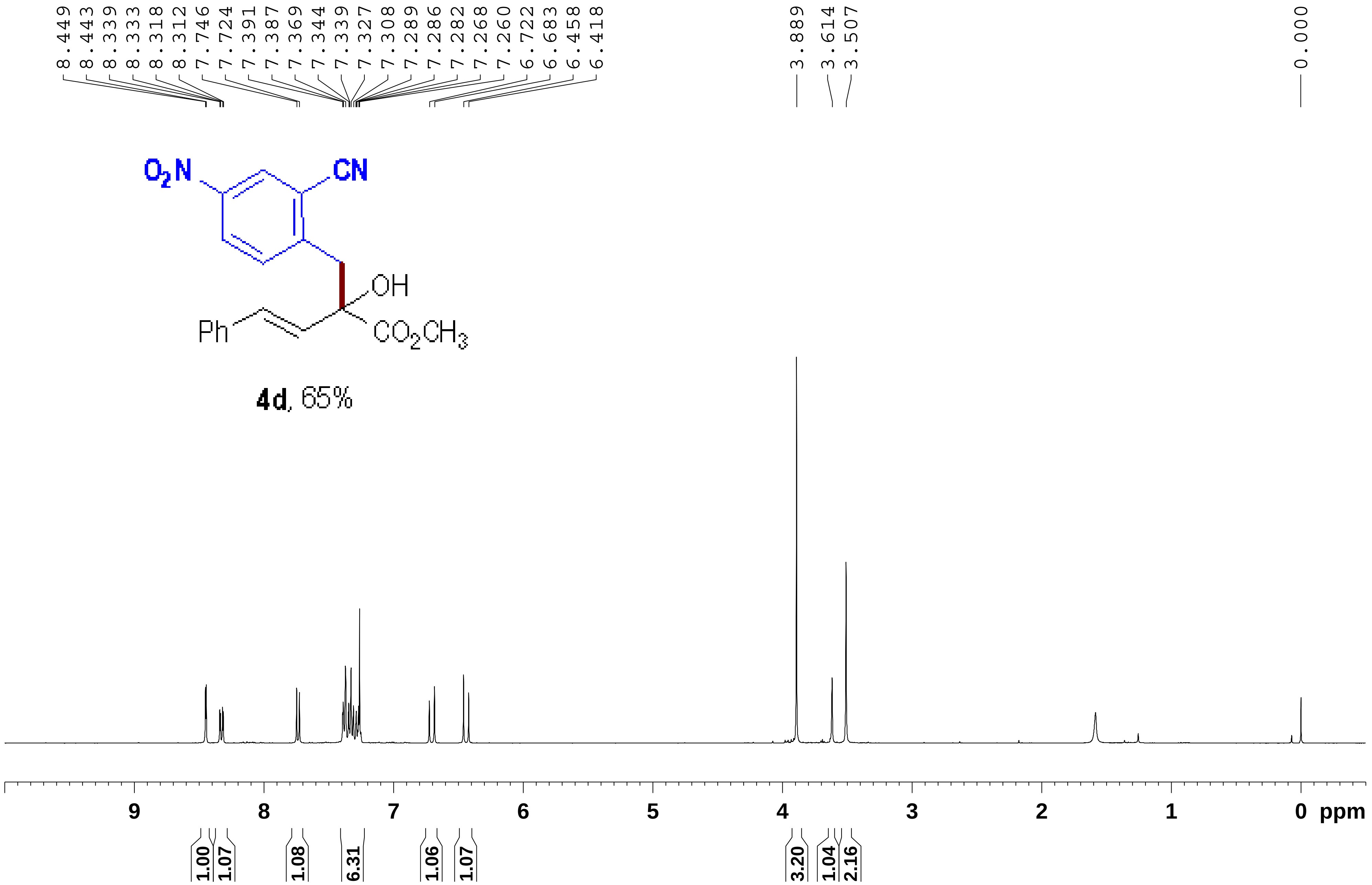

Supplementary Figure 8. ^{13}C NMR Spectrum of substrate 4d

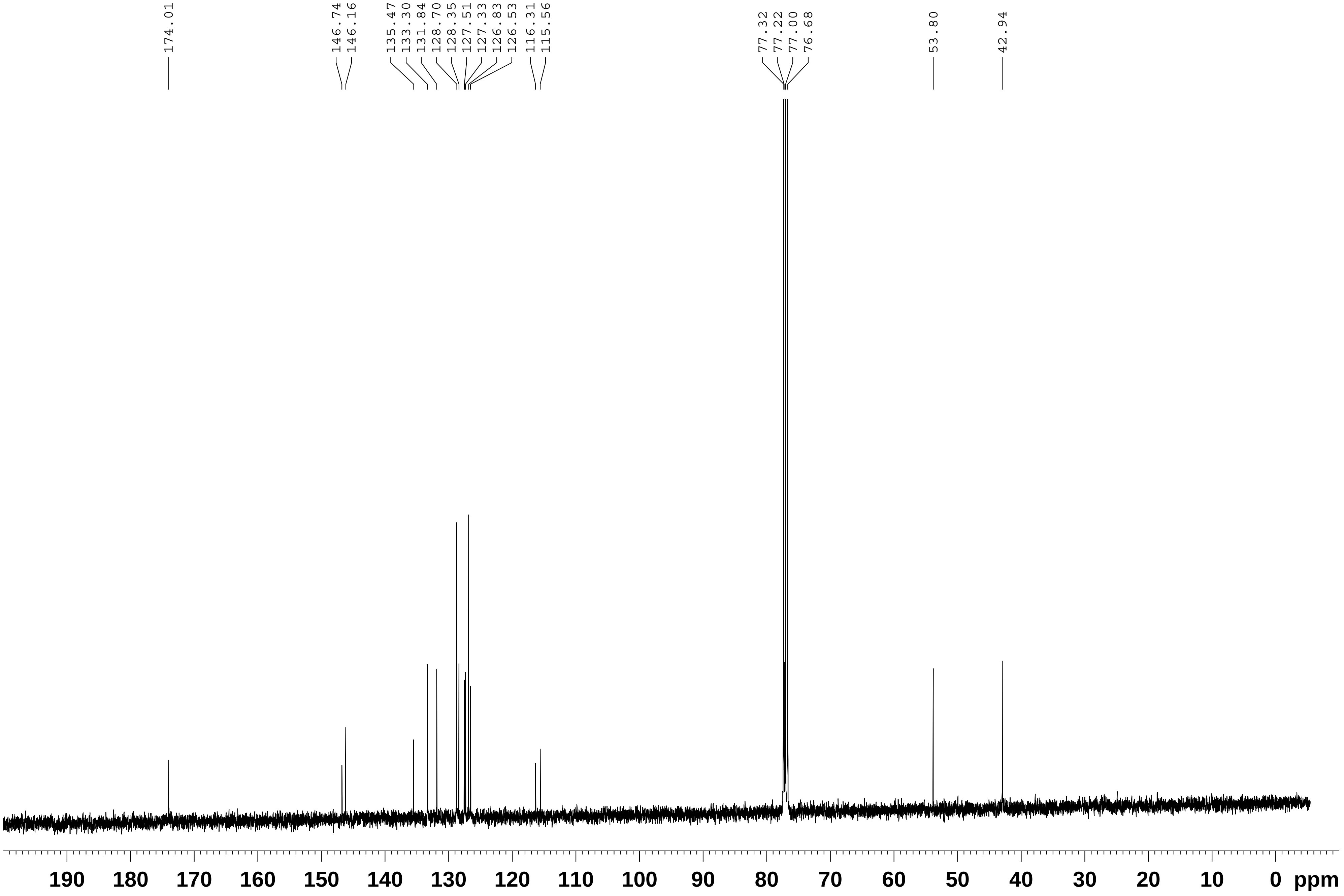

Supplementary Figure 9. ^{1}H NMR Spectrum of substrate 4e

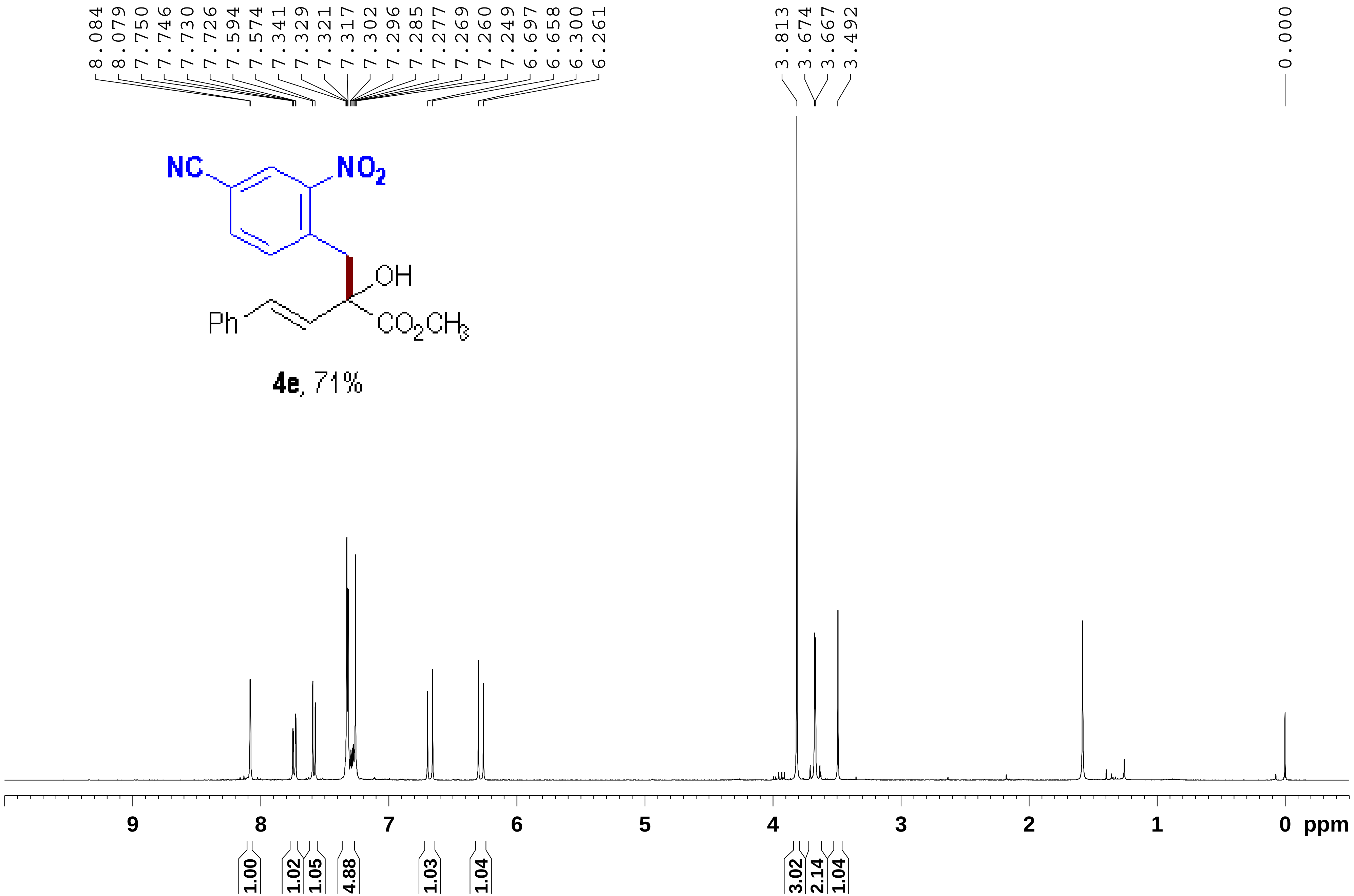

Supplementary Figure 10. ^{13}C NMR Spectrum of substrate 4e

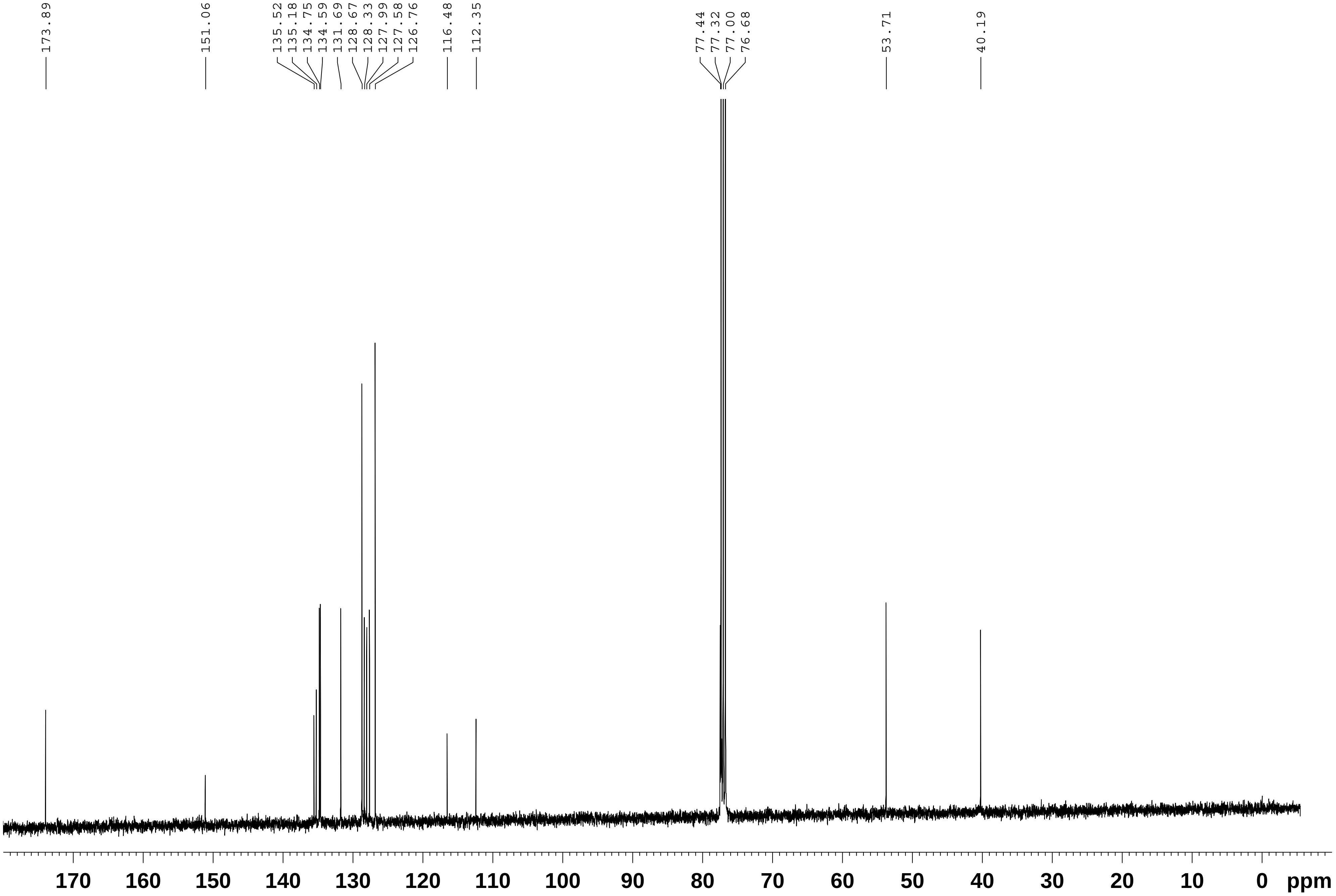

Supplementary Figure 11. ^1H NMR Spectrum of substrate 4f

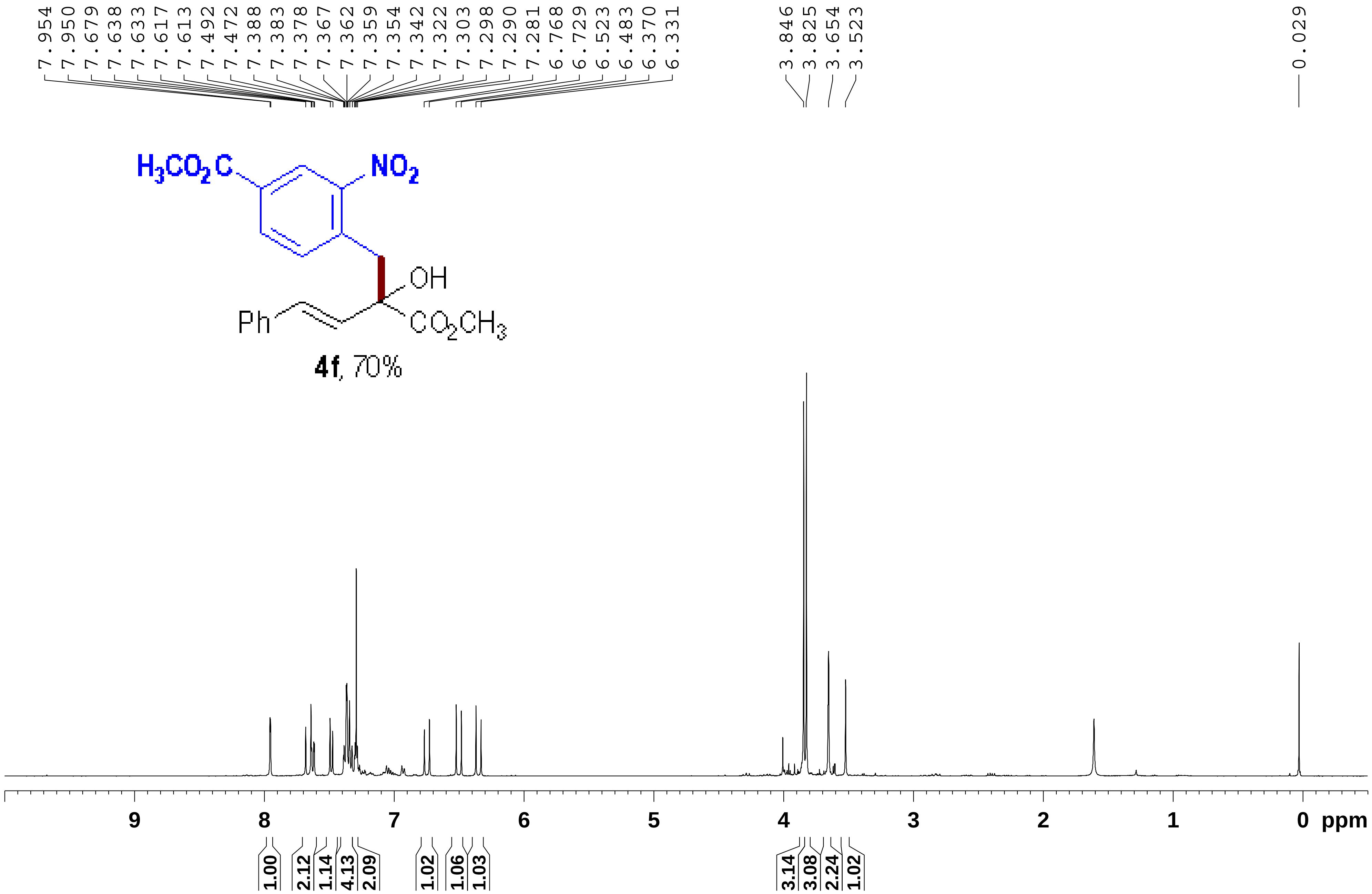

Supplementary Figure 12. ^{13}C NMR Spectrum of substrate 4f

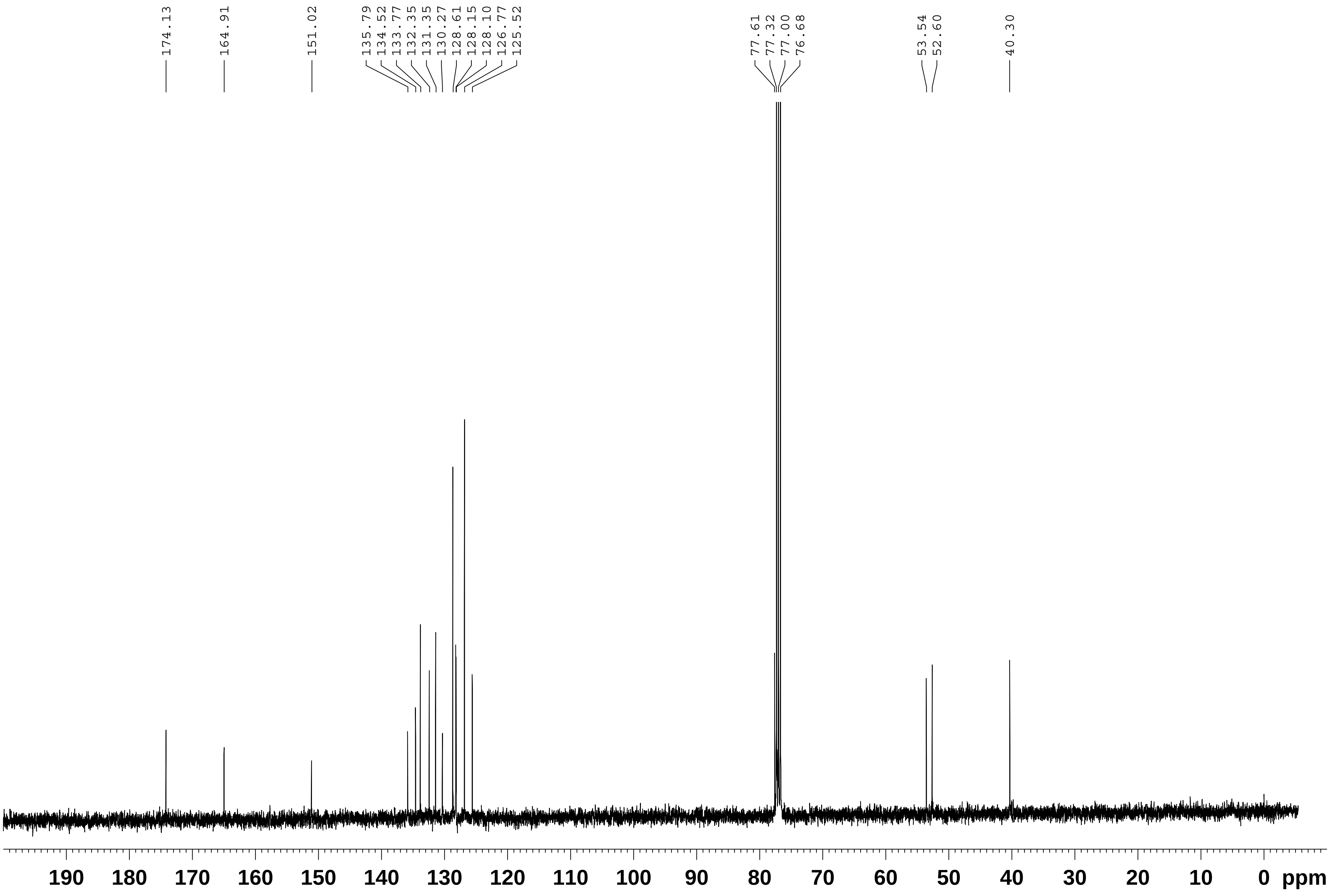

Supplementary Figure 13. ^{1}H NMR Spectrum of substrate 4g

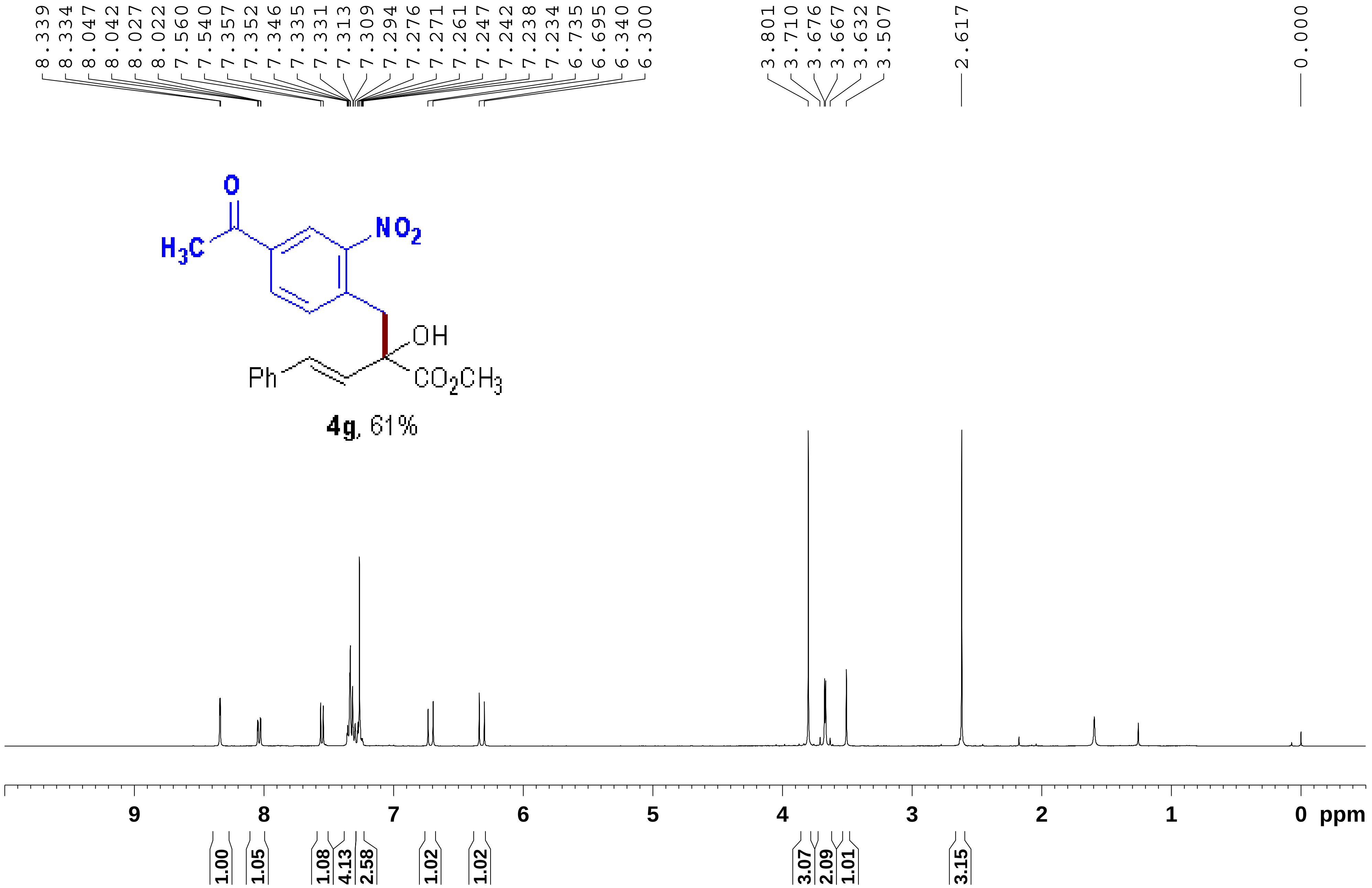

Supplementary Figure 14. ^{13}C NMR Spectrum of substrate 4g

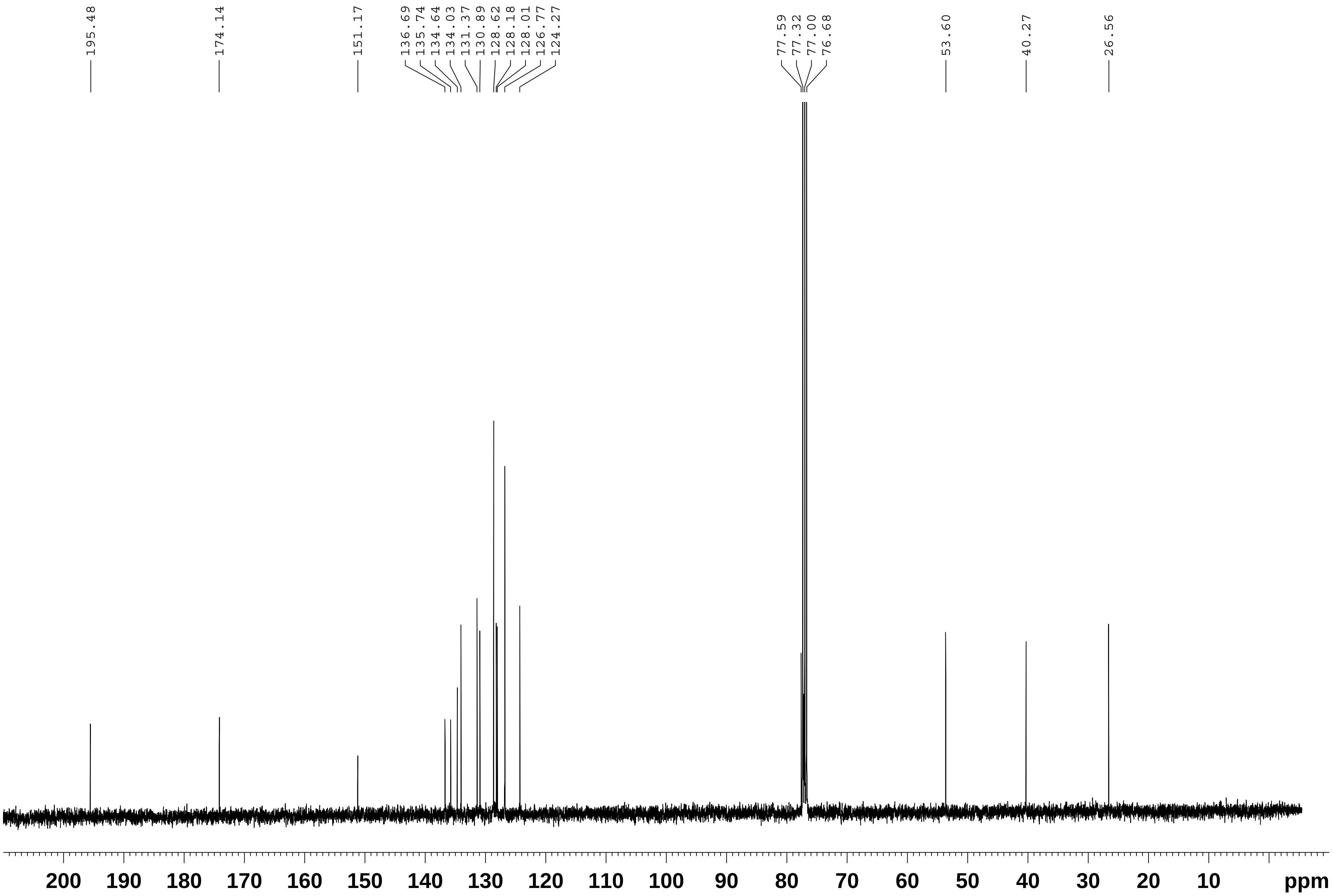

Supplementary Figure 15. ^1H NMR Spectrum of substrate 4h

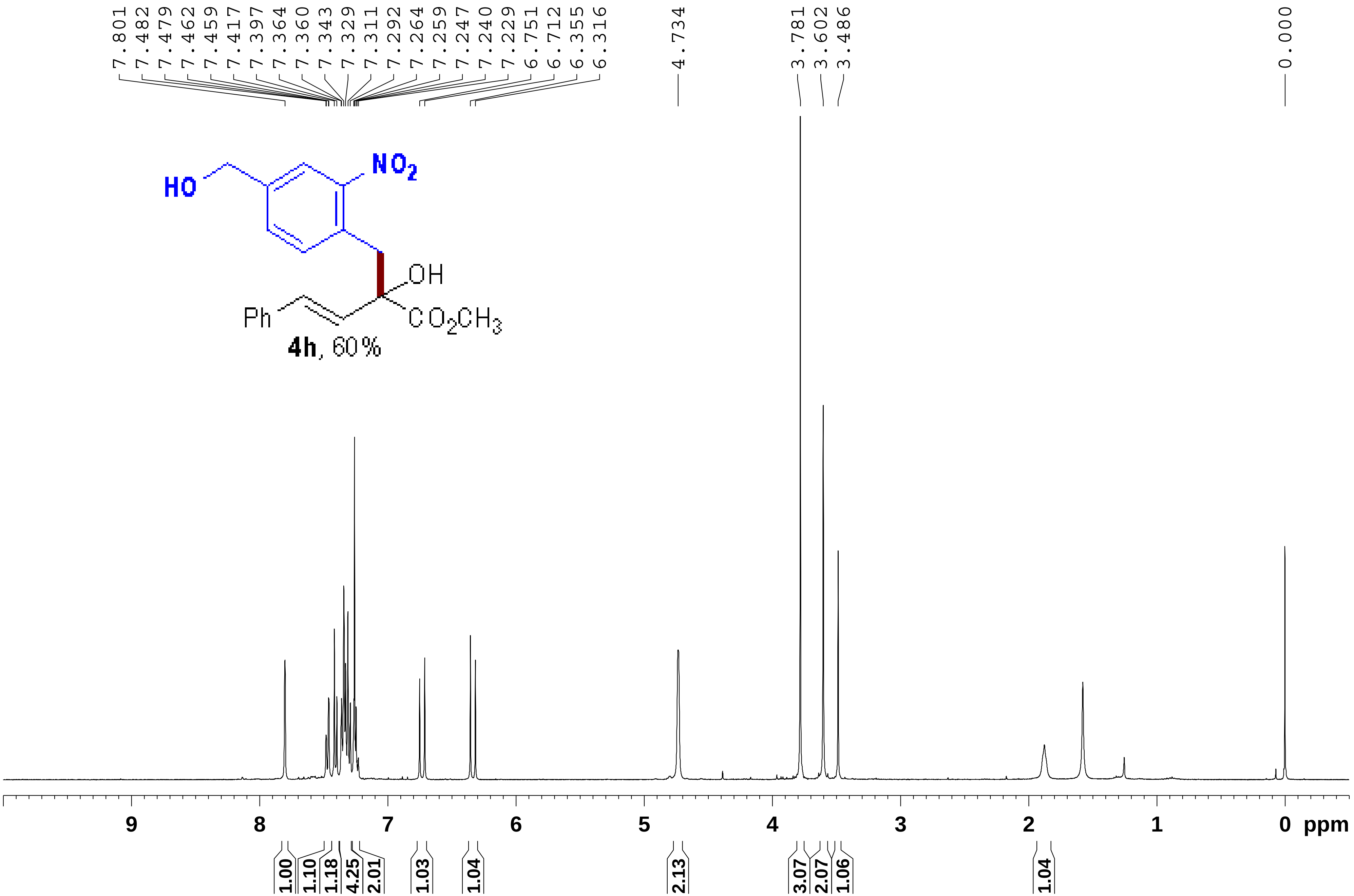

Supplementary Figure 16. ^{13}C NMR Spectrum of substrate 4h

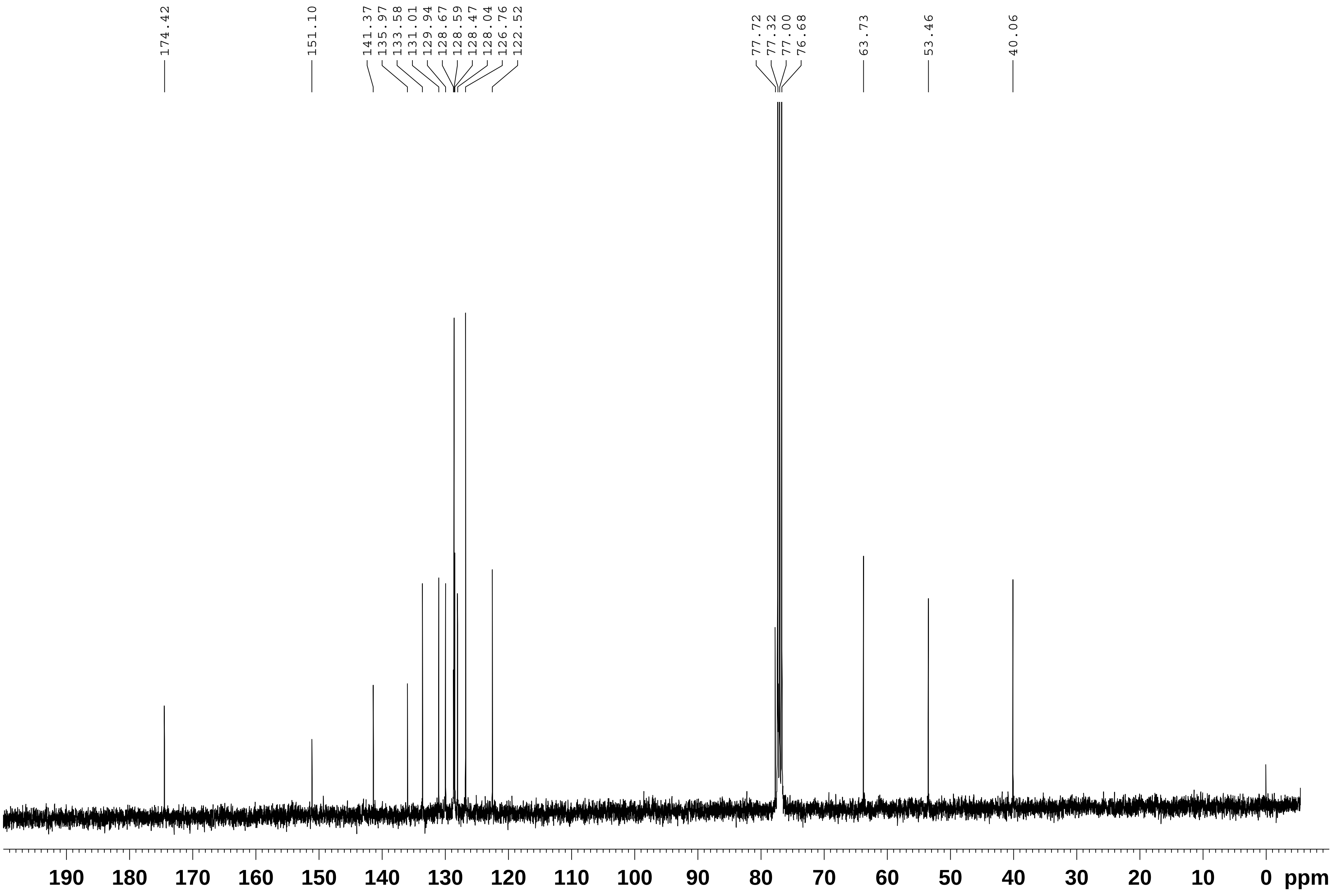

Supplementary Figure 17. ^{1}H NMR Spectrum of substrate 4i

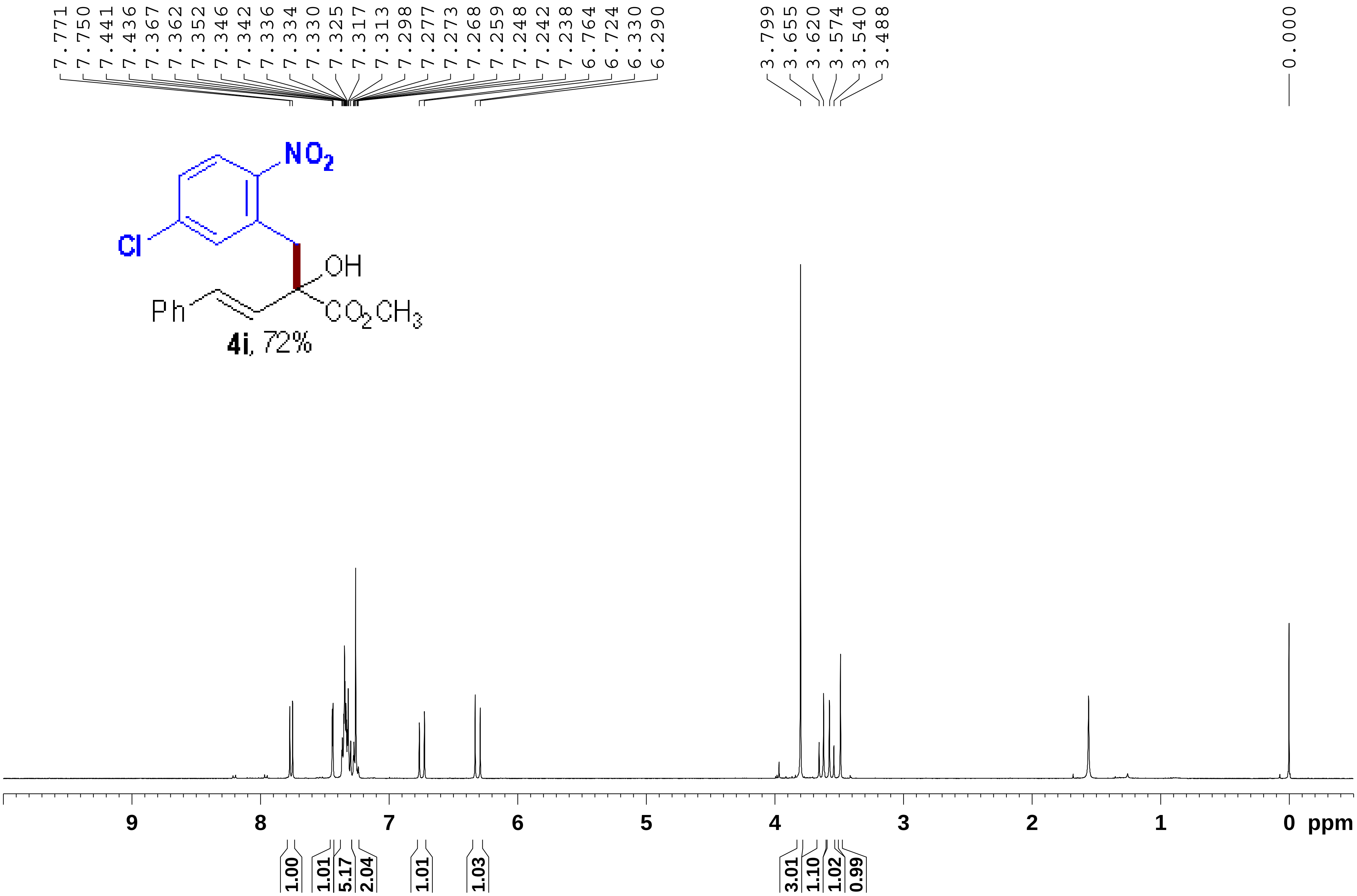

Supplementary Figure 18. ^{13}C NMR Spectrum of substrate 4i

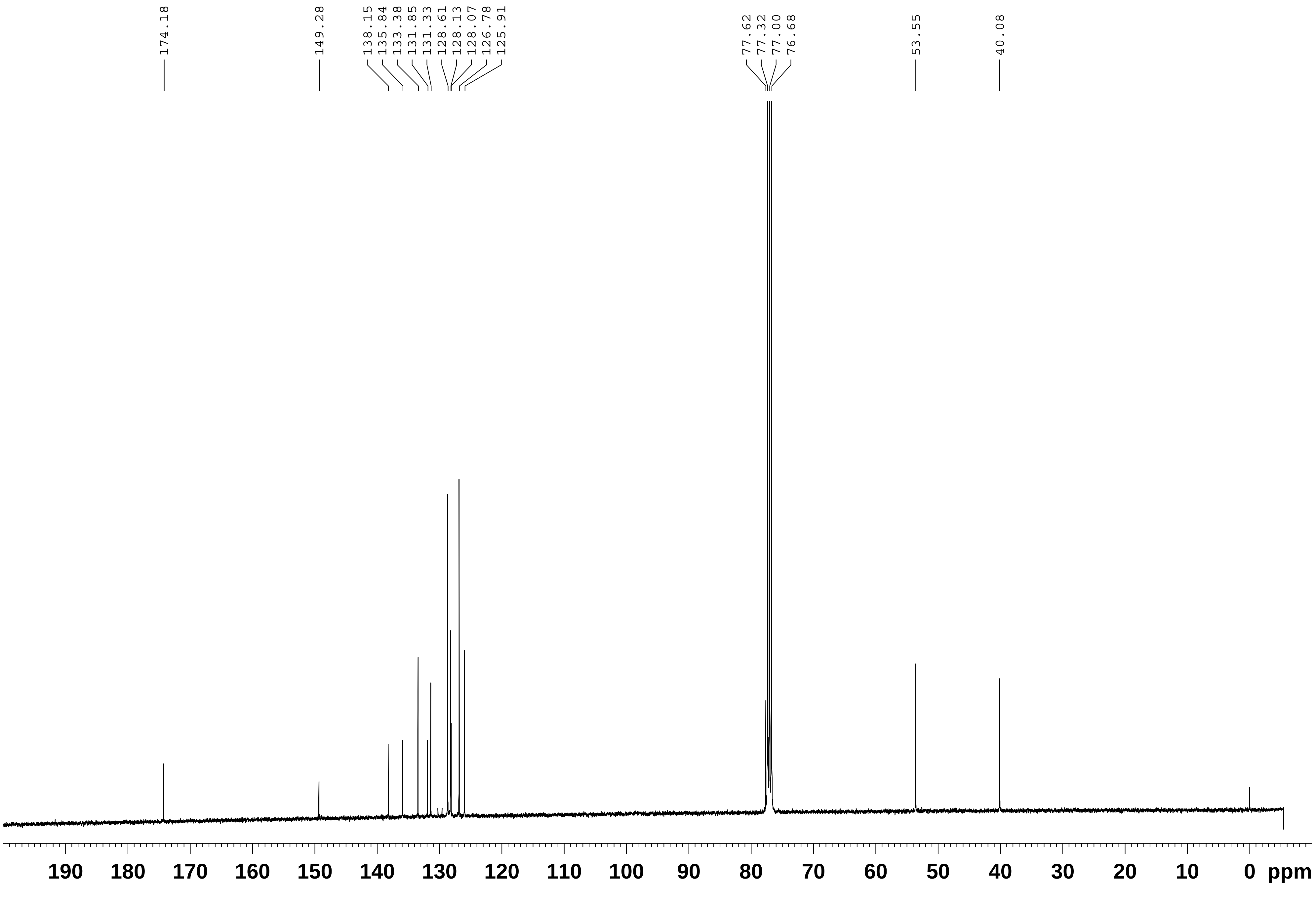

Supplementary Figure 19. ^{1}H NMR Spectrum of substrate 4j

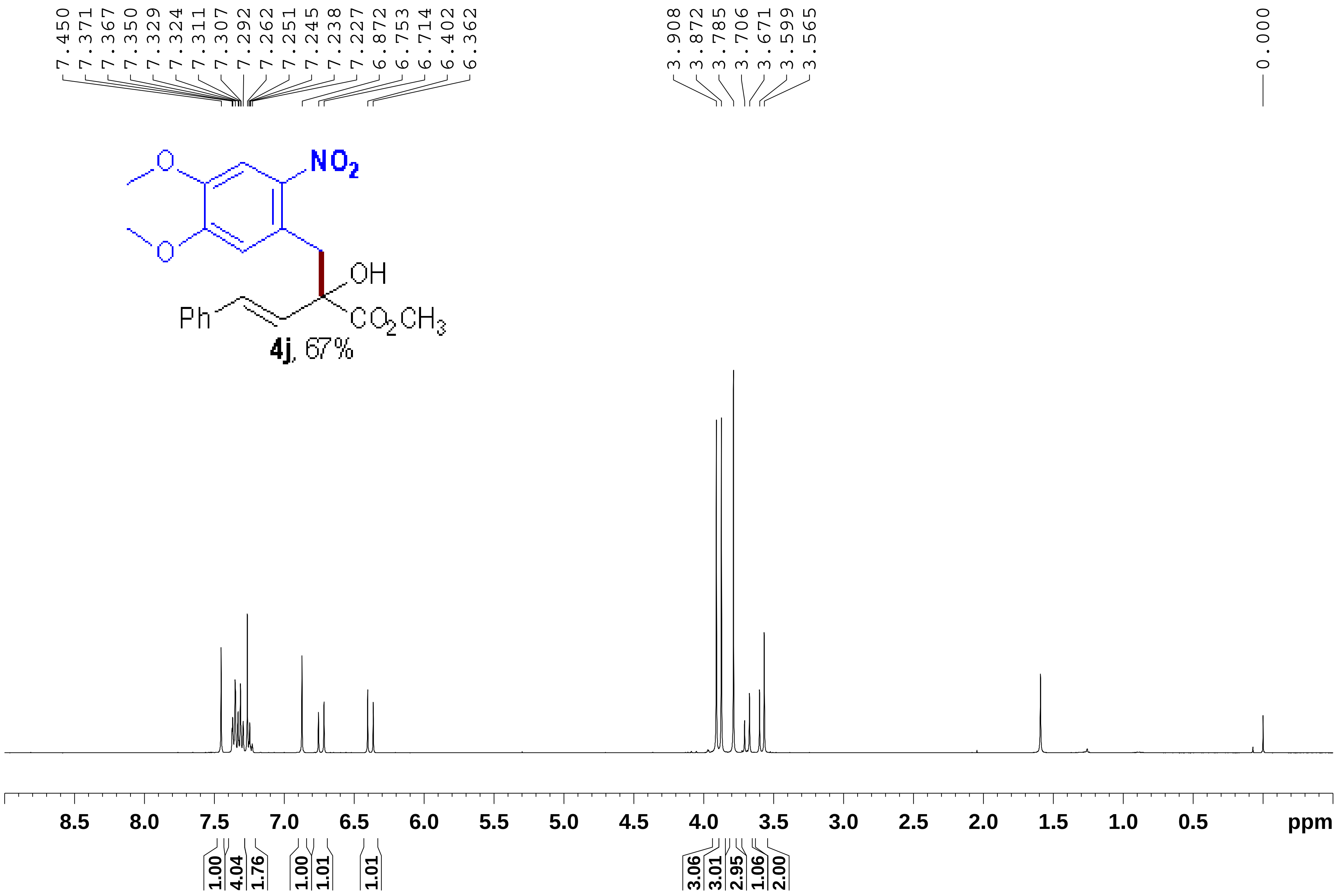

Supplementary Figure 20. ^{13}C NMR Spectrum of substrate 4j

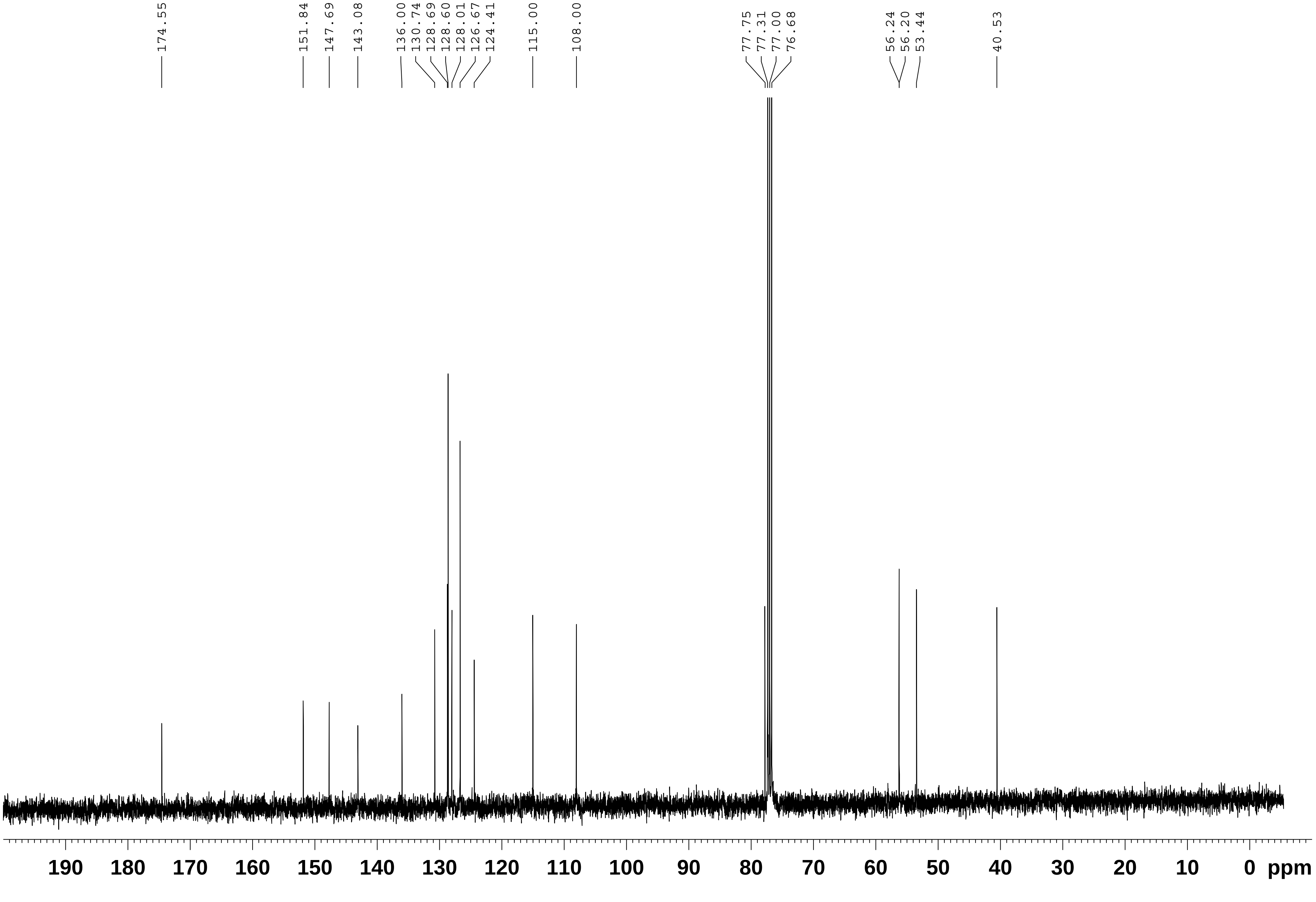

Supplementary Figure 21. ^{1}H NMR Spectrum of substrate 4k

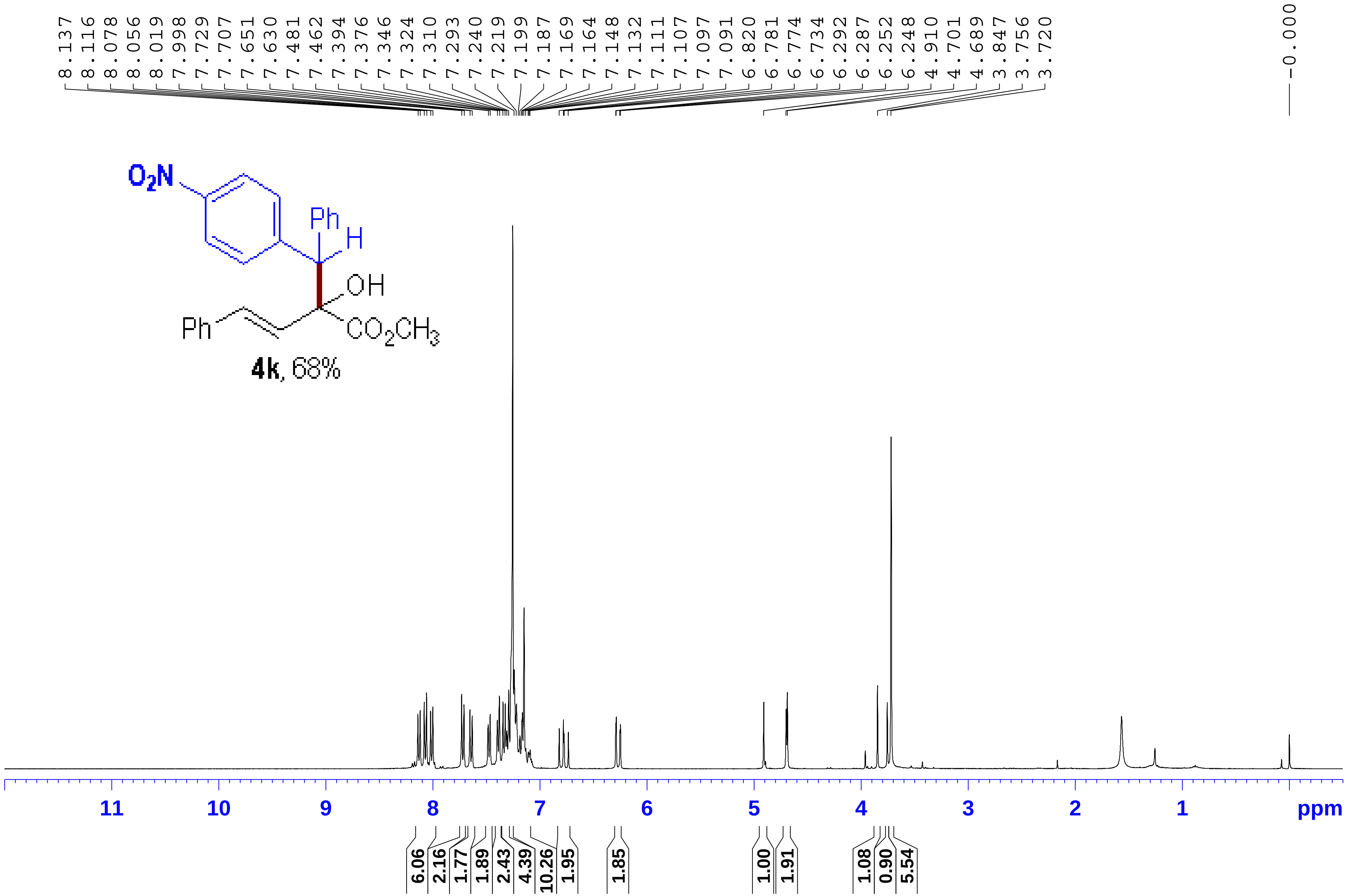

Supplementary Figure 22. ^{13}C NMR Spectrum of substrate 4k

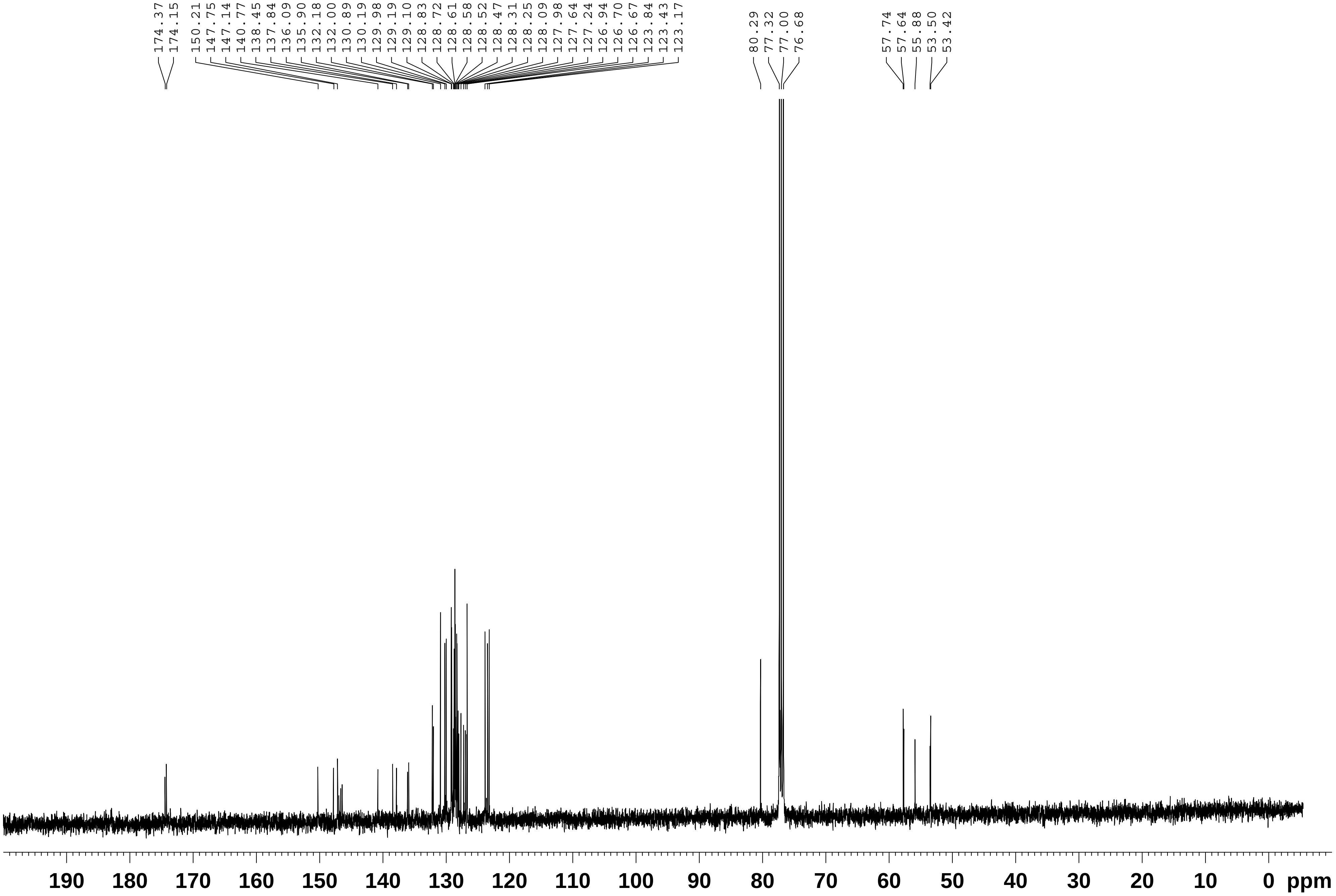

Supplementary Figure 23. ^{1}H NMR Spectrum of substrate 4l

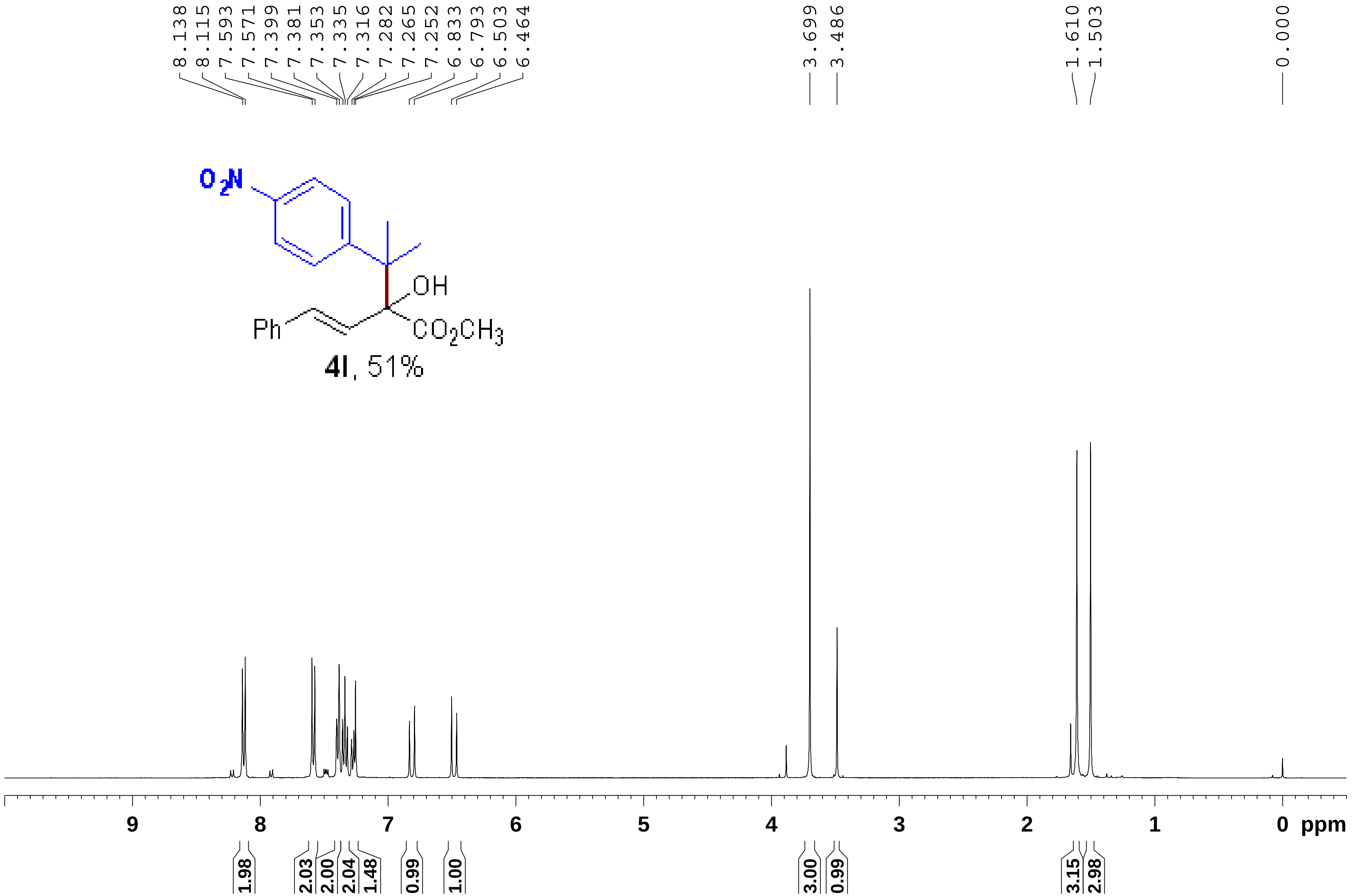

Supplementary Figure 24. ^{13}C NMR Spectrum of substrate 4l

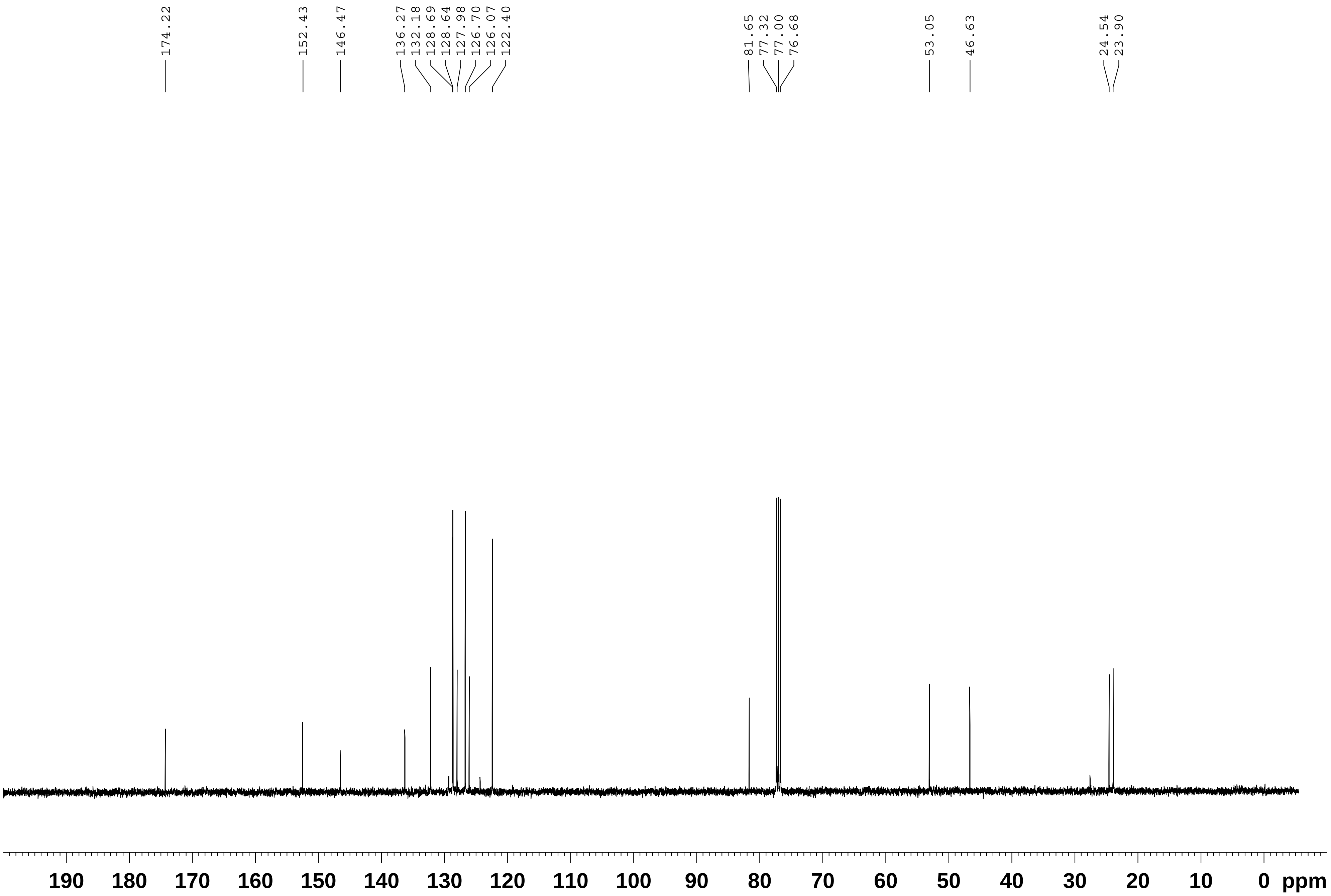

Supplementary Figure 25. ^{1}H NMR Spectrum of substrate 4m

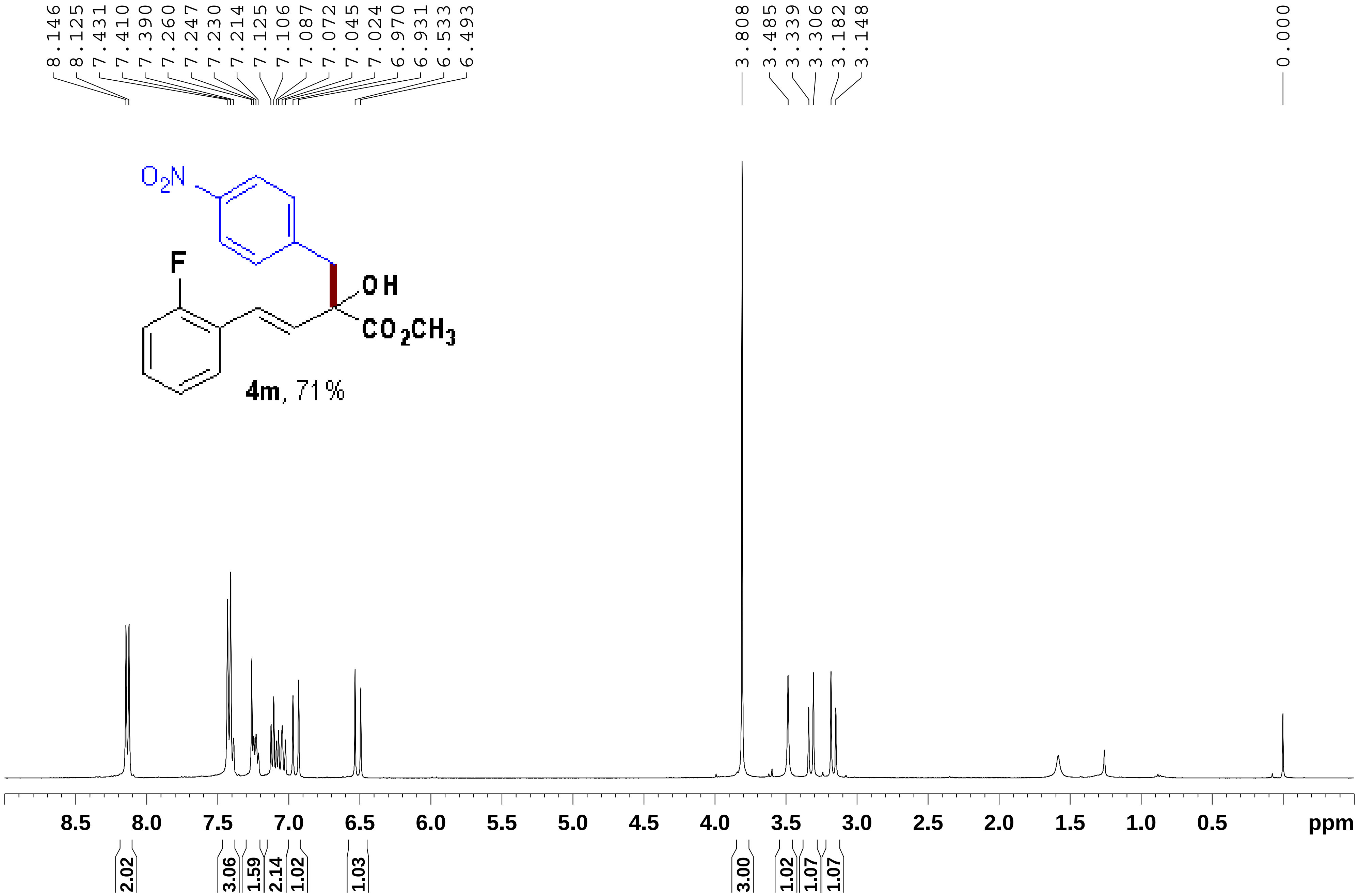

Supplementary Figure 26. ^{13}C NMR Spectrum of substrate 4m

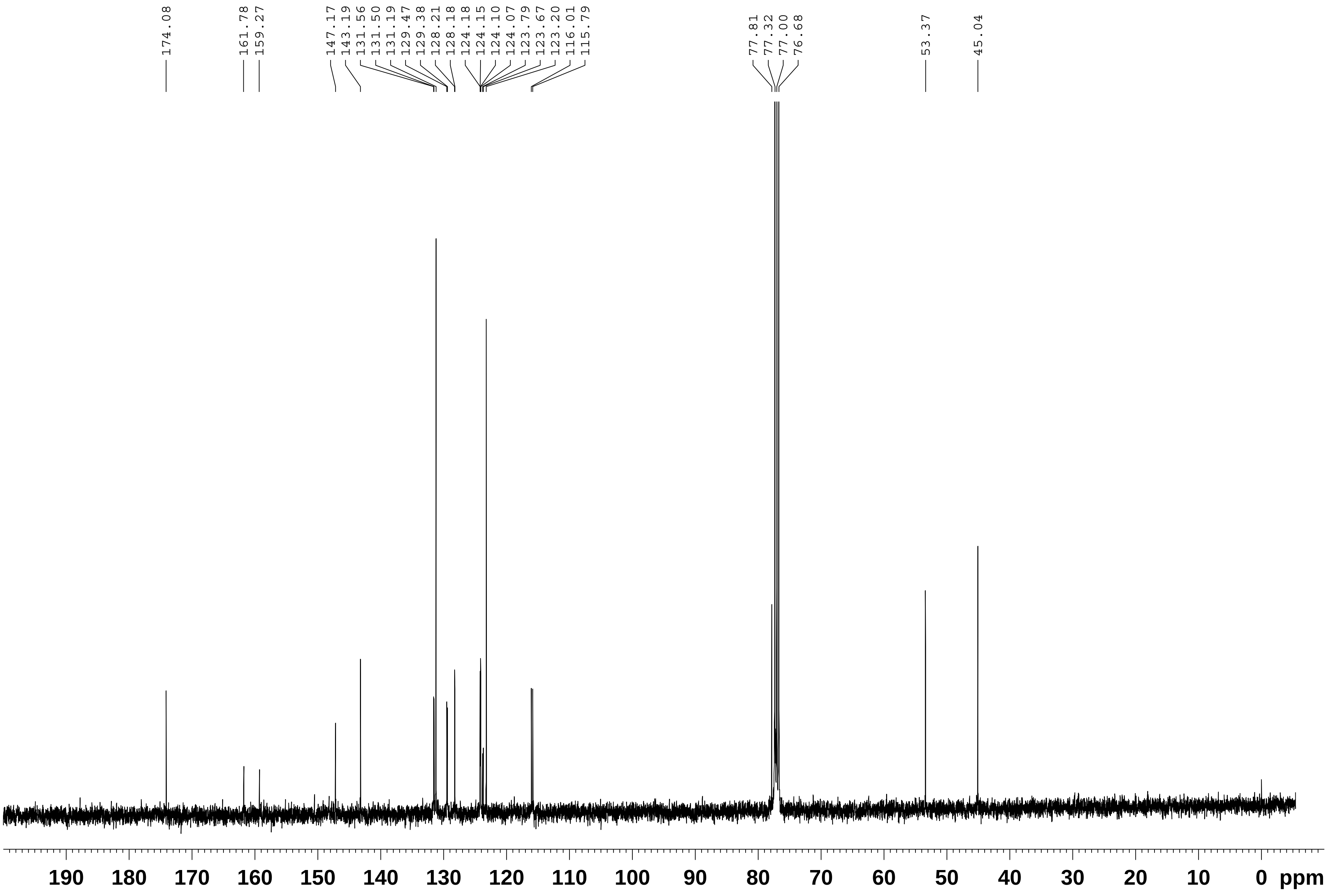

Supplementary Figure 27. ^{19}F NMR Spectrum of substrate 4m

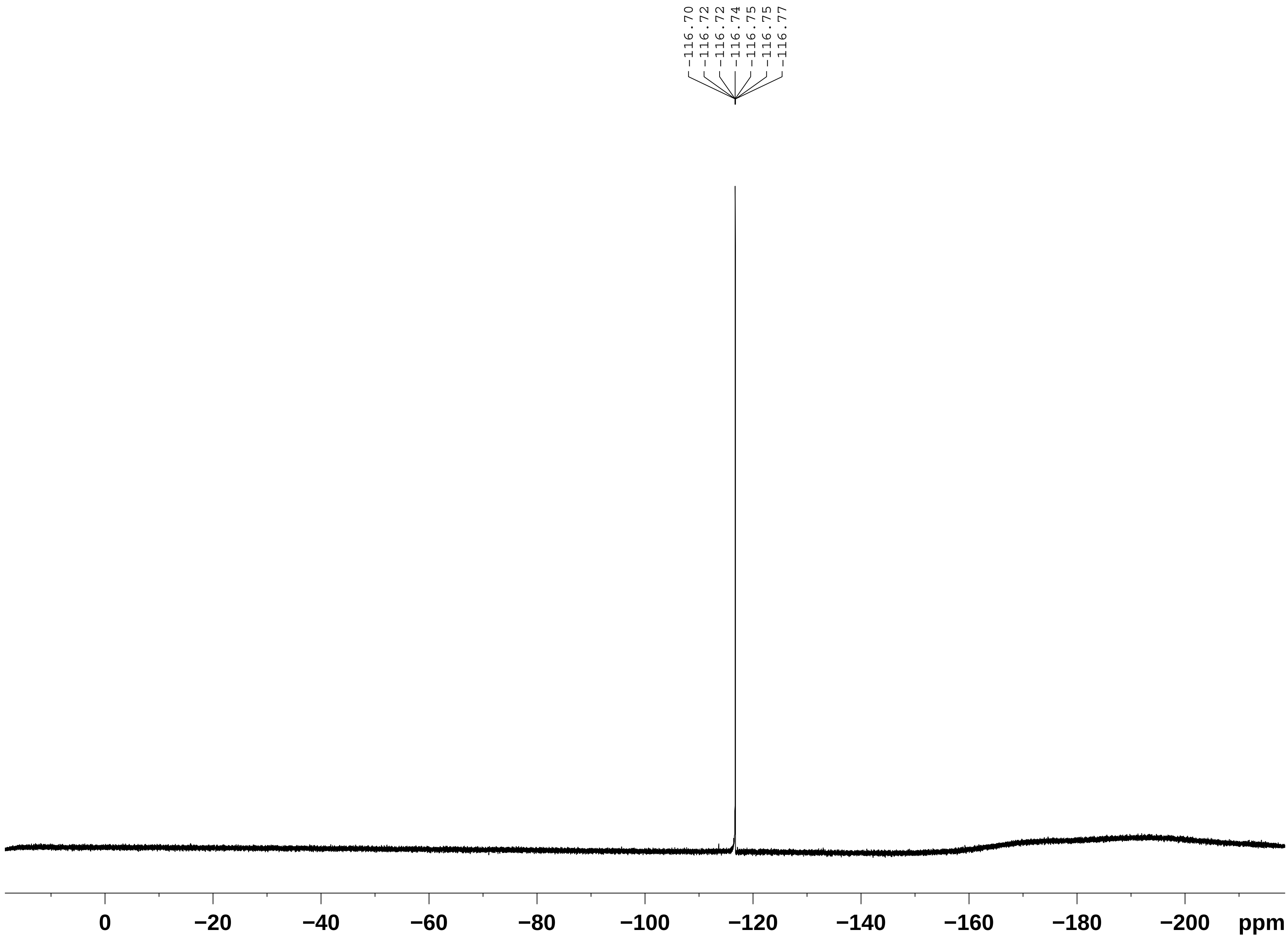

Supplementary Figure 28. ^{1}H NMR Spectrum of substrate 4n

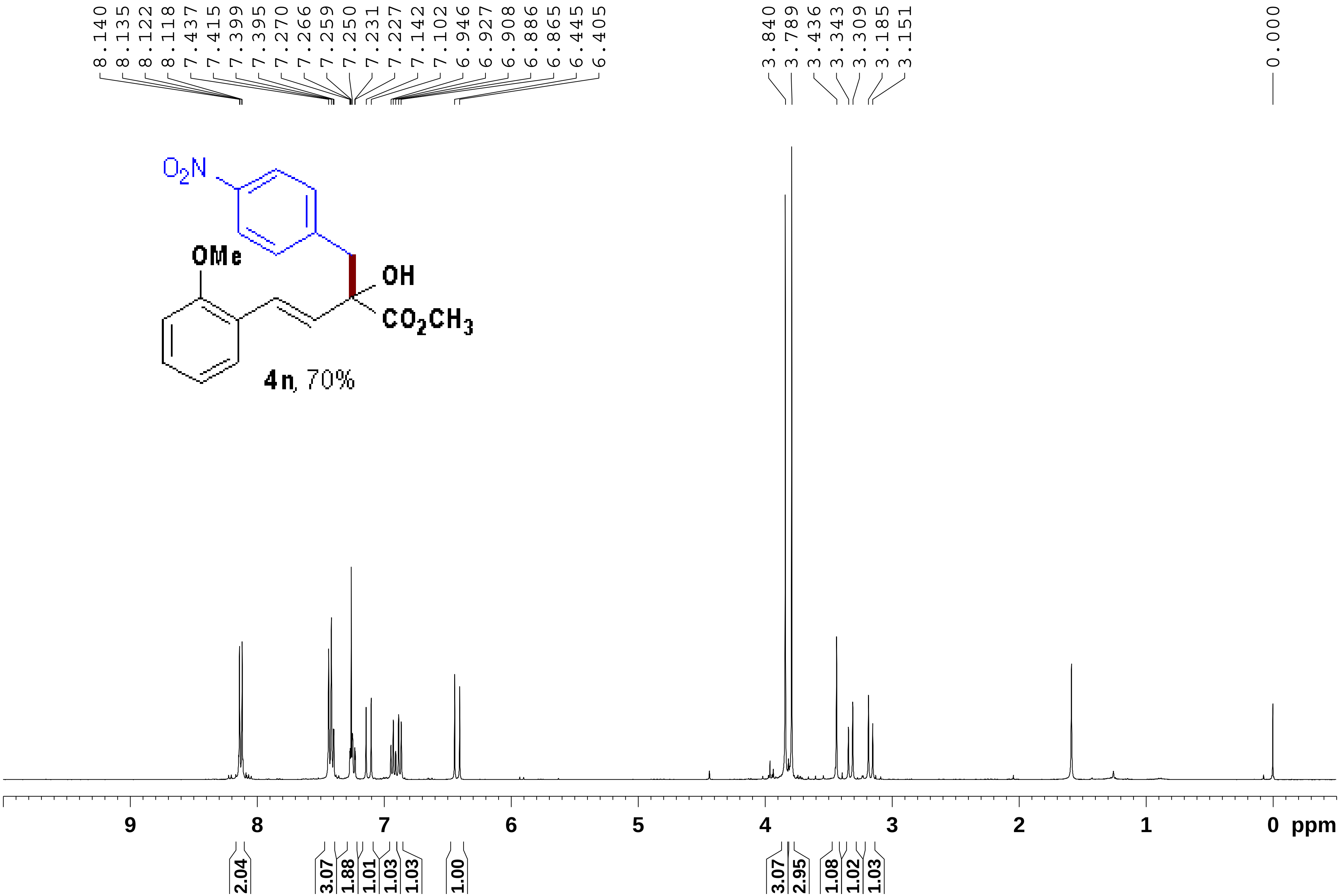

Supplementary Figure 29. ^{13}C NMR Spectrum of substrate 4n

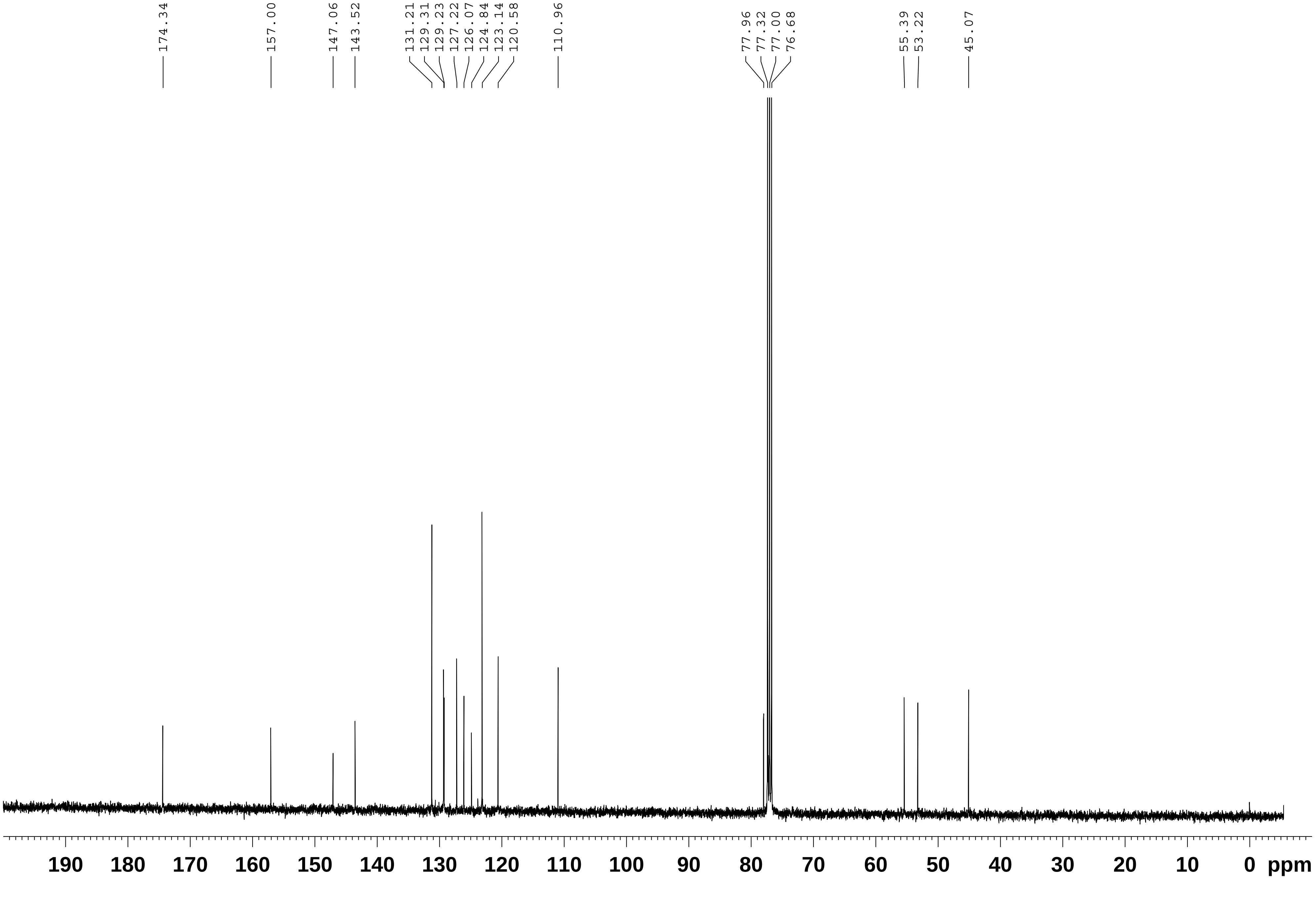

Supplementary Figure 30. ^{1}H NMR Spectrum of substrate 4o

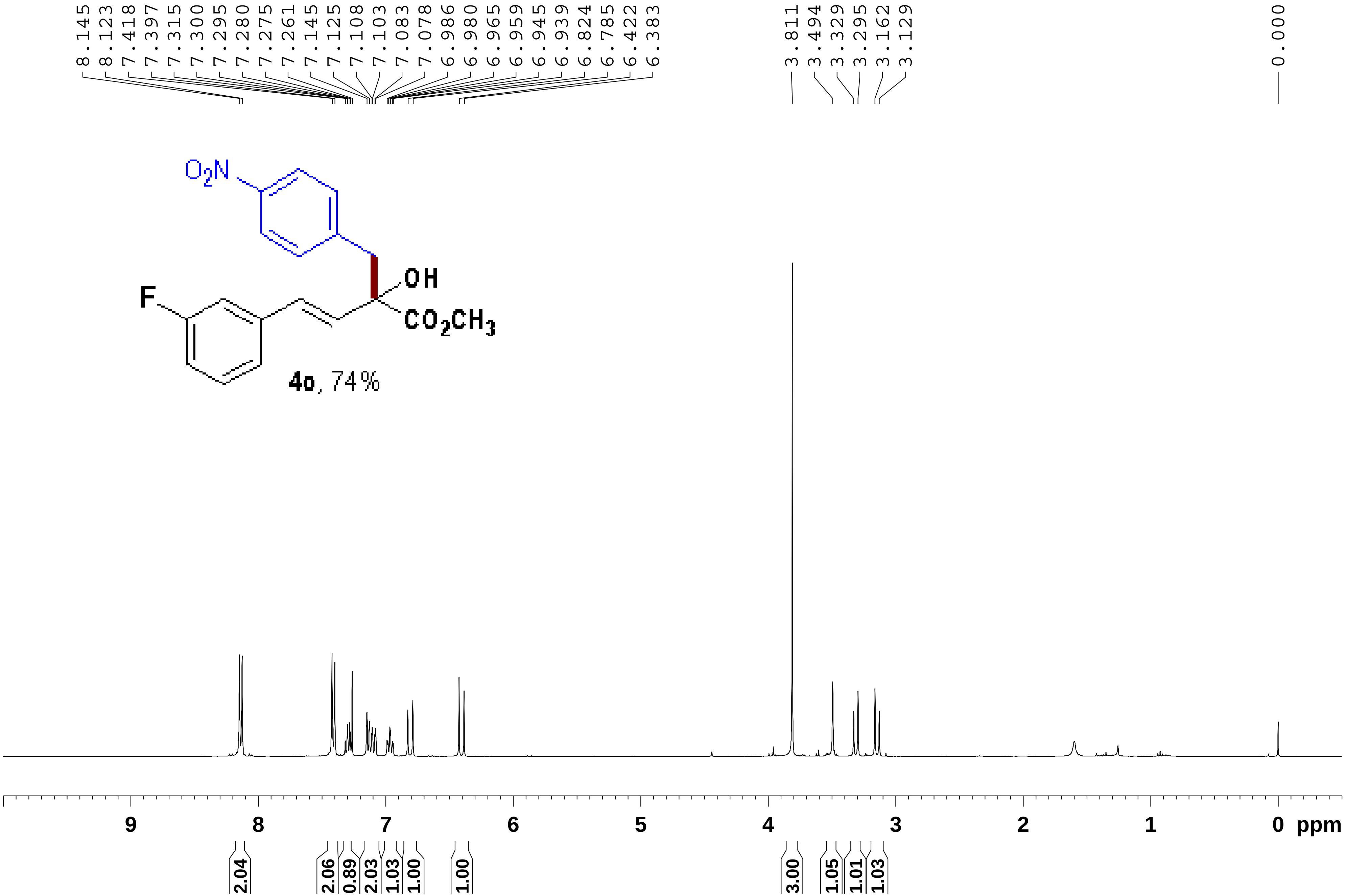

Supplementary Figure 31. ^{13}C NMR Spectrum of substrate 4o

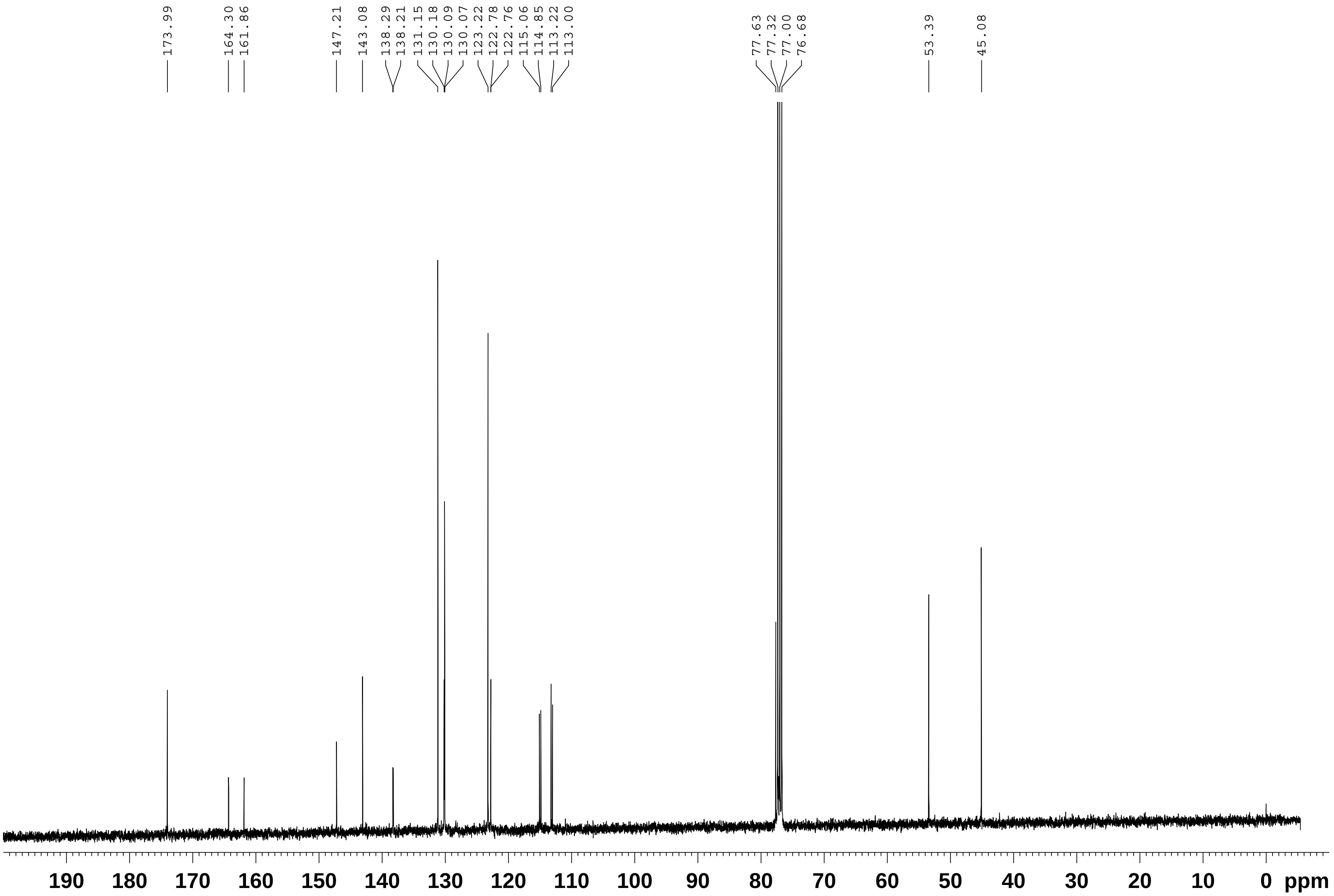

Supplementary Figure 32. ^{19}F NMR Spectrum of substrate 4o

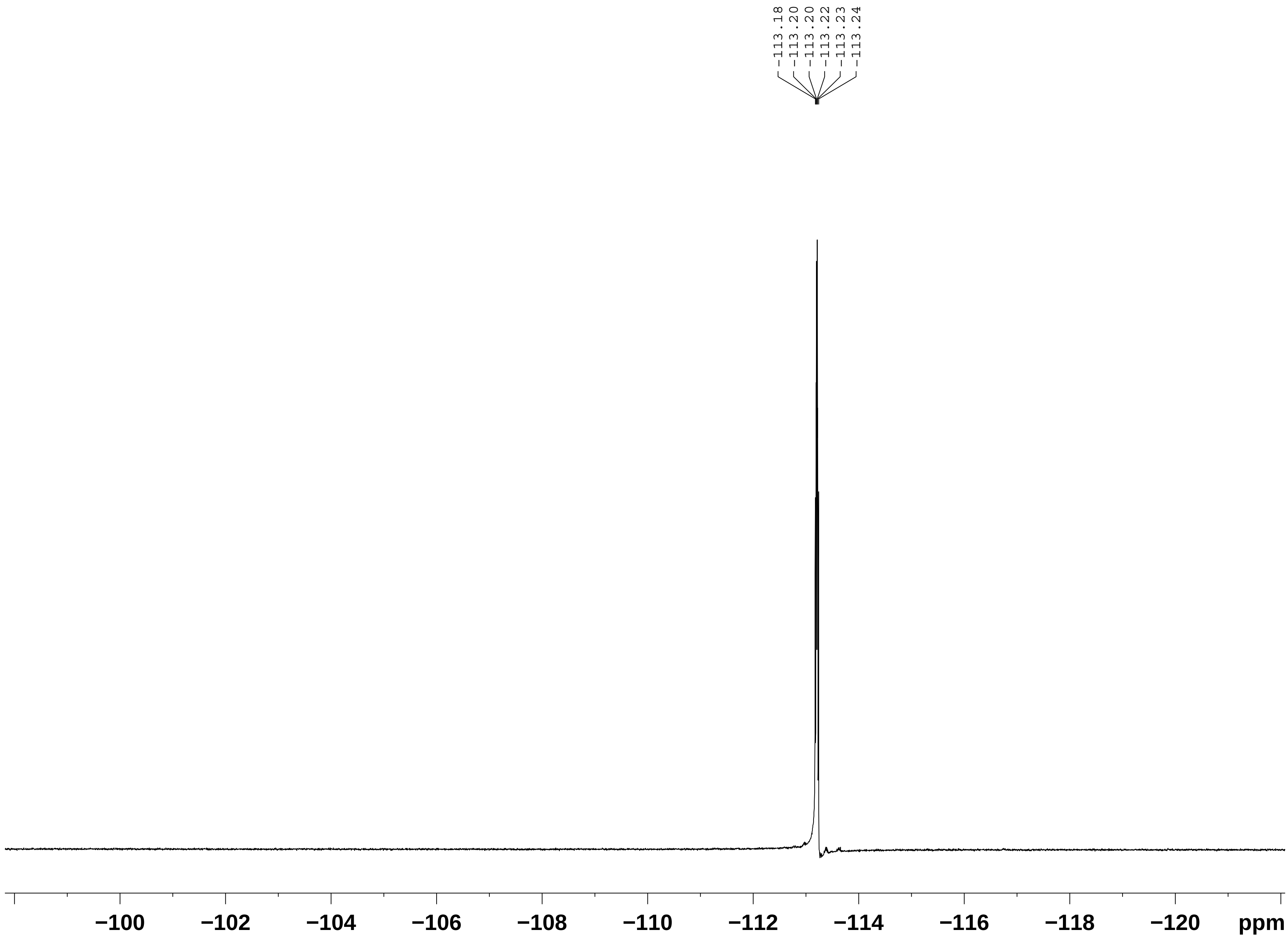

Supplementary Figure 33. ^{1}**H NMR Spectrum of substrate 4p**

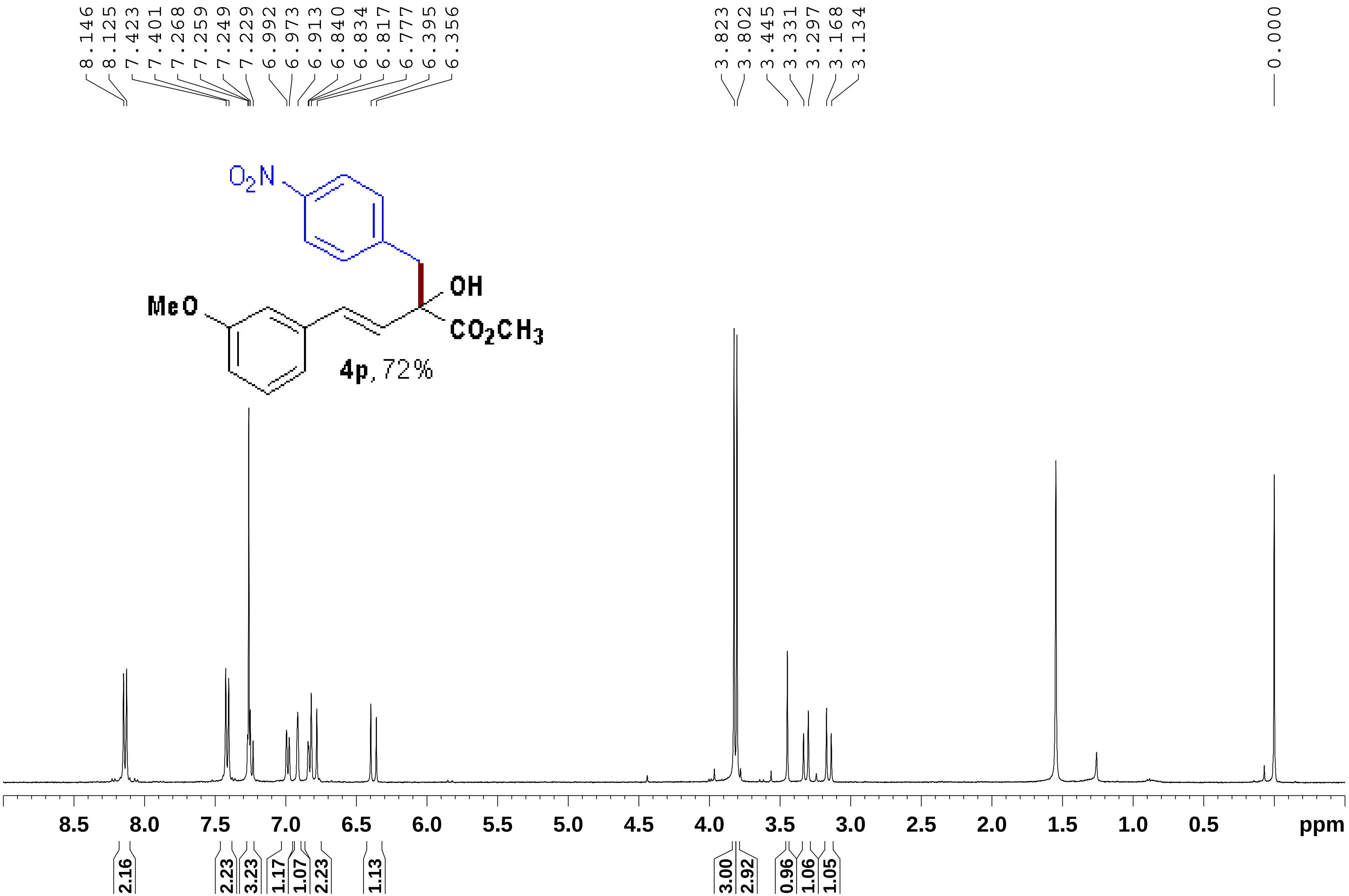

Supplementary Figure 34. ^{13}C NMR Spectrum of substrate 4p

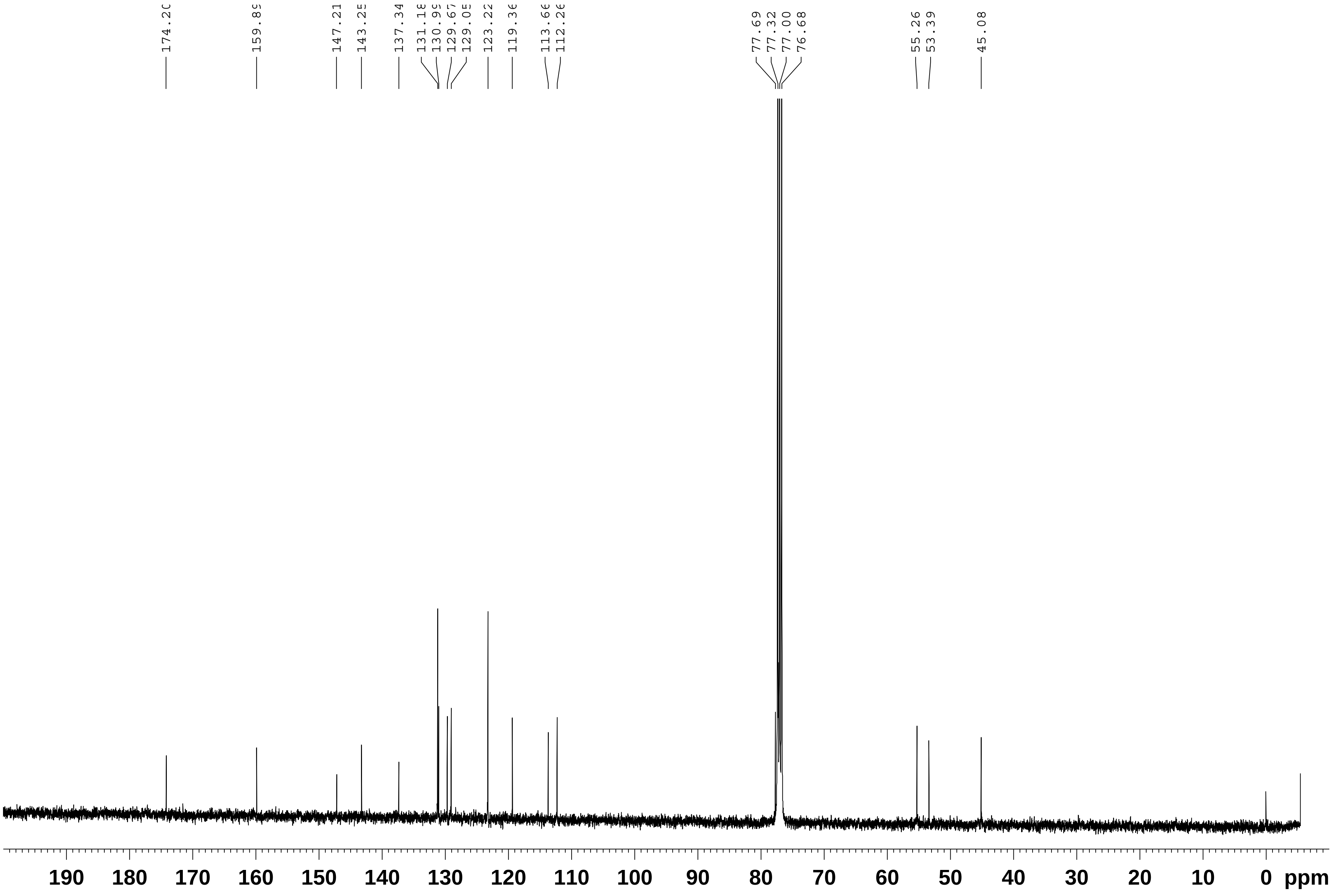

Supplementary Figure 35. ^{1}H NMR Spectrum of substrate 4q

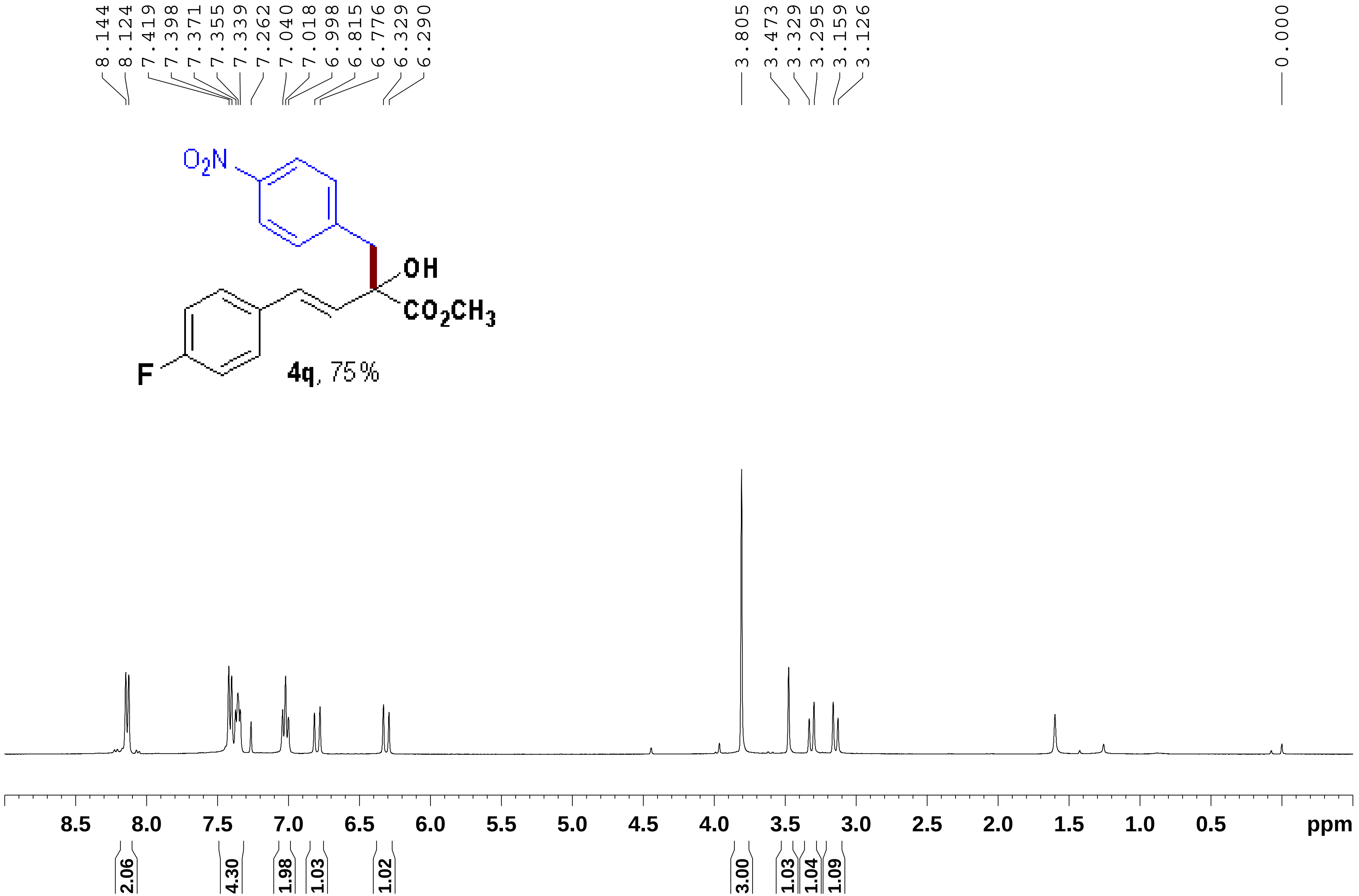

Supplementary Figure 36. ^{13}C NMR Spectrum of substrate 4q

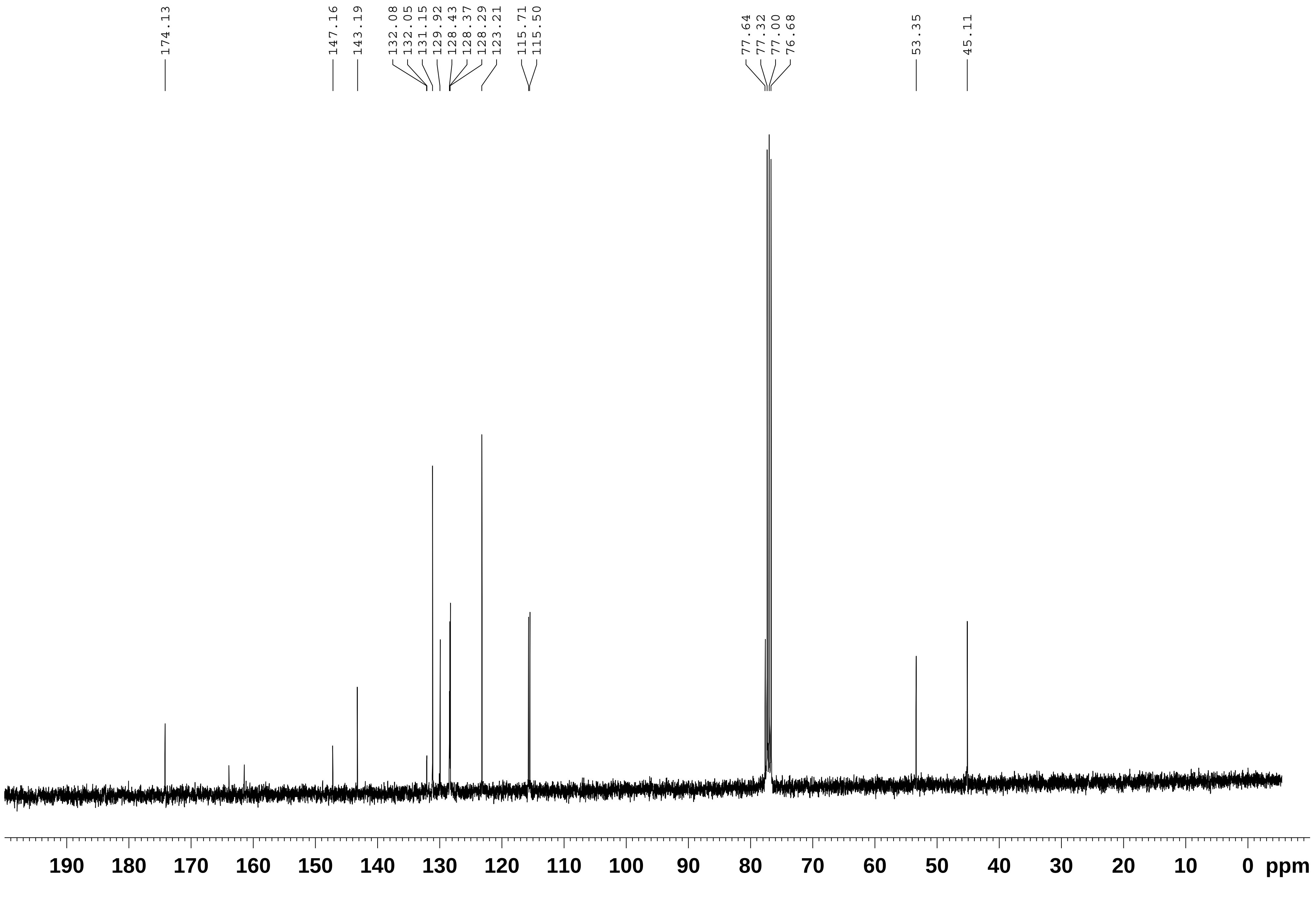

Supplementary Figure 37. ¹H NMR Spectrum of substrate 4q

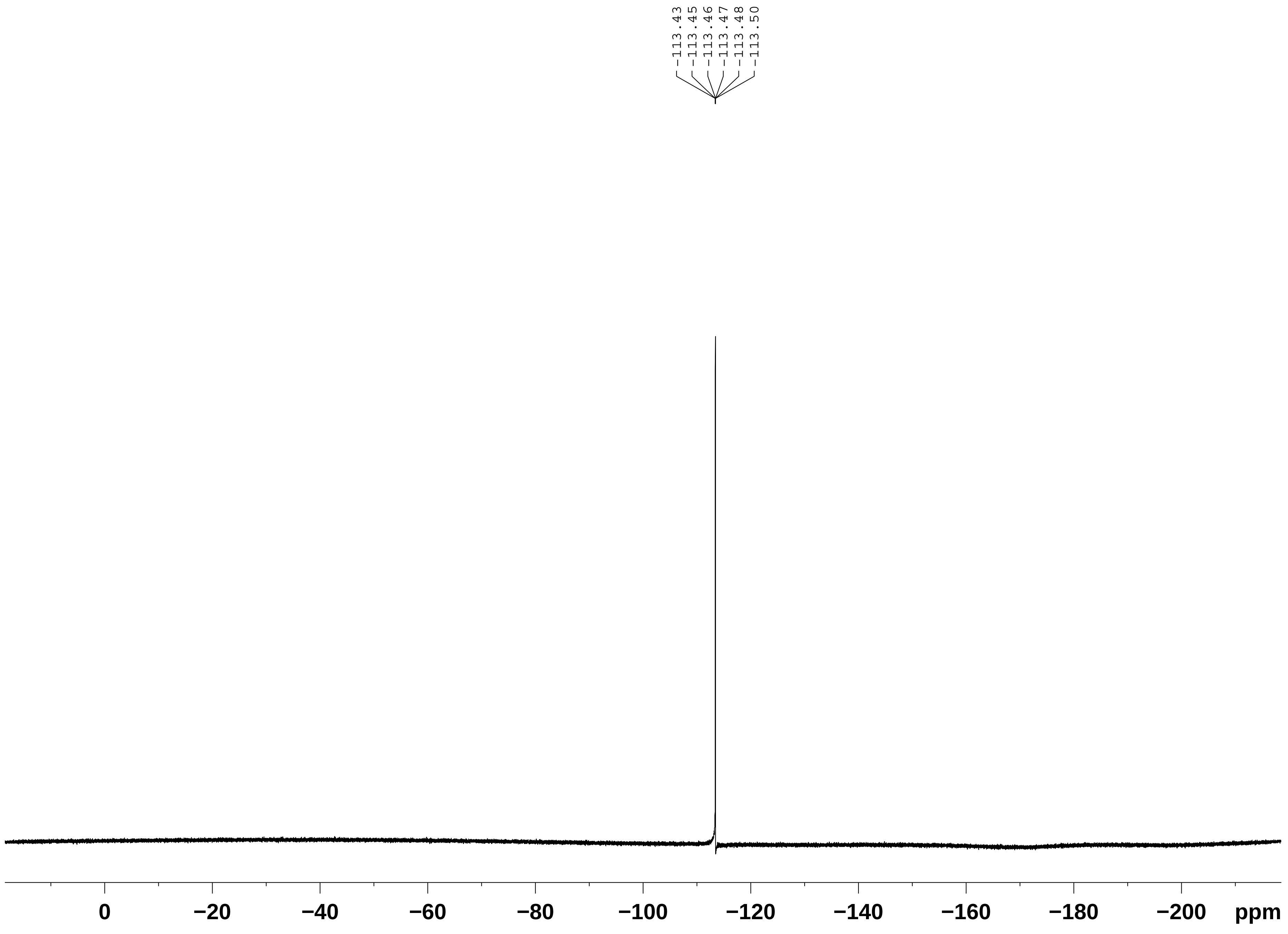

Supplementary Figure 38. ^{1}H NMR Spectrum of substrate 4r

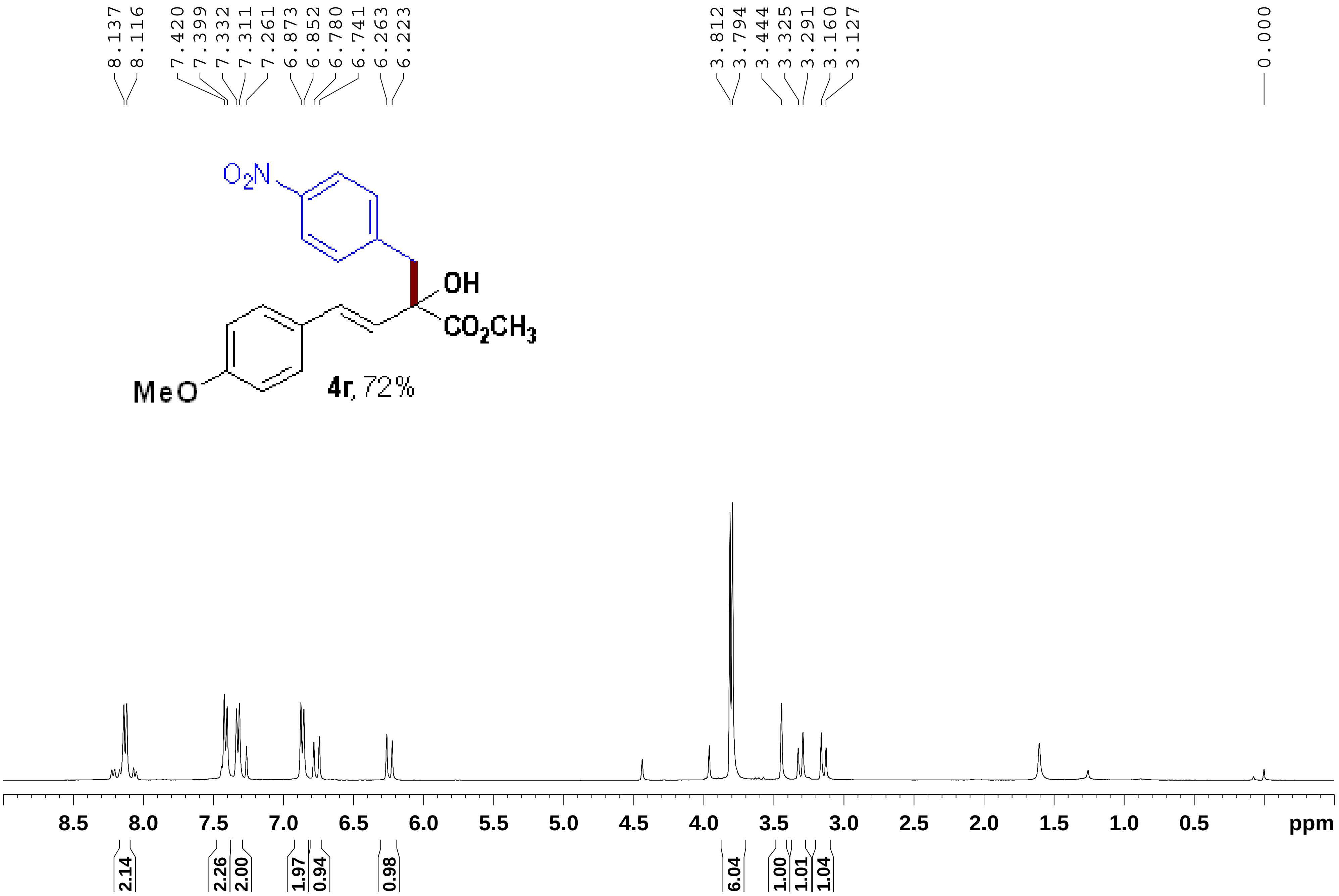

Supplementary Figure 39. ^{13}C NMR Spectrum of substrate 4r

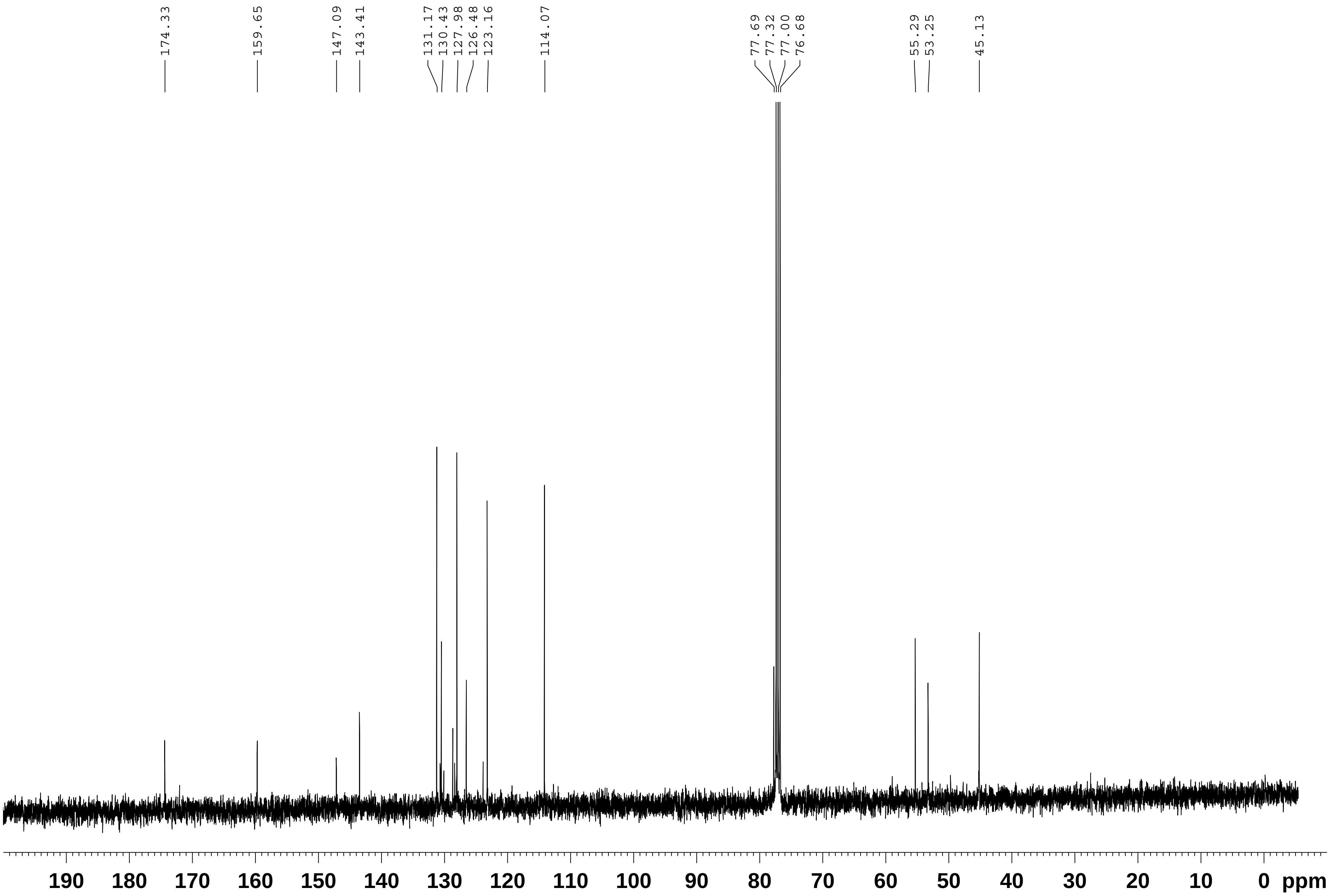

Supplementary Figure 40. ^{1}H NMR Spectrum of substrate 4s

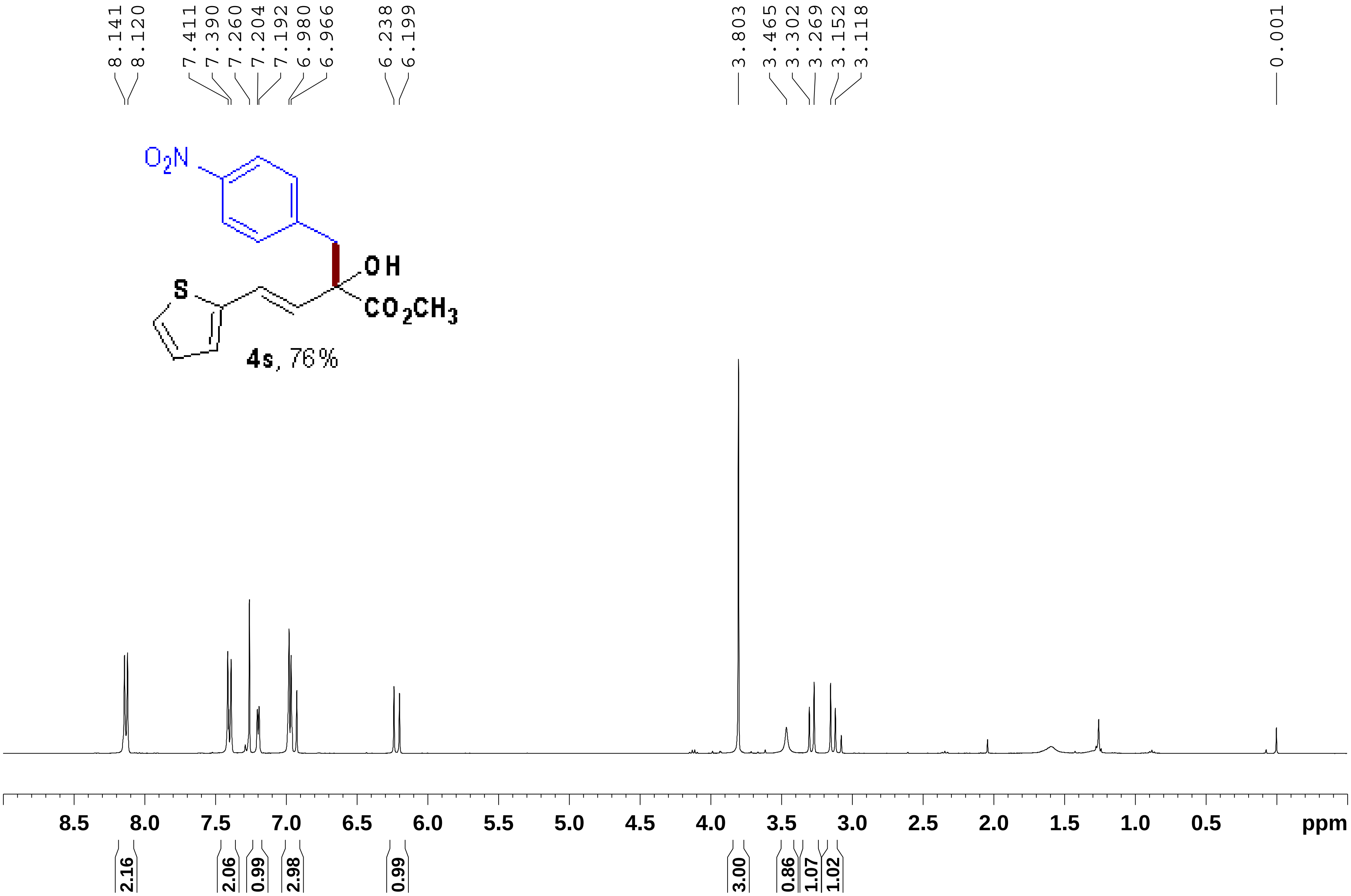

Supplementary Figure 41. ^{13}C NMR Spectrum of substrate 4s

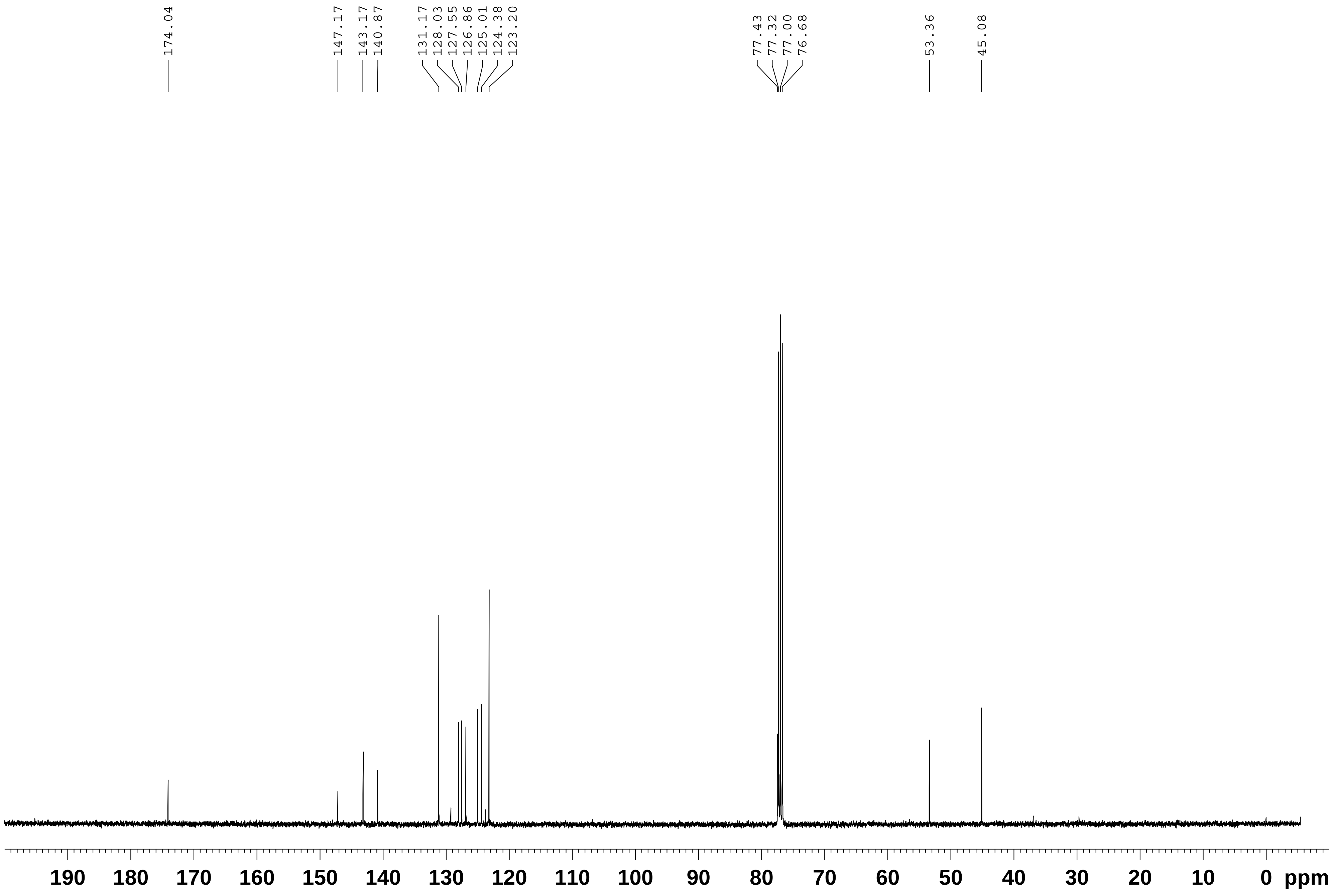

Supplementary Figure 42. ^{1}H NMR Spectrum of substrate 4t

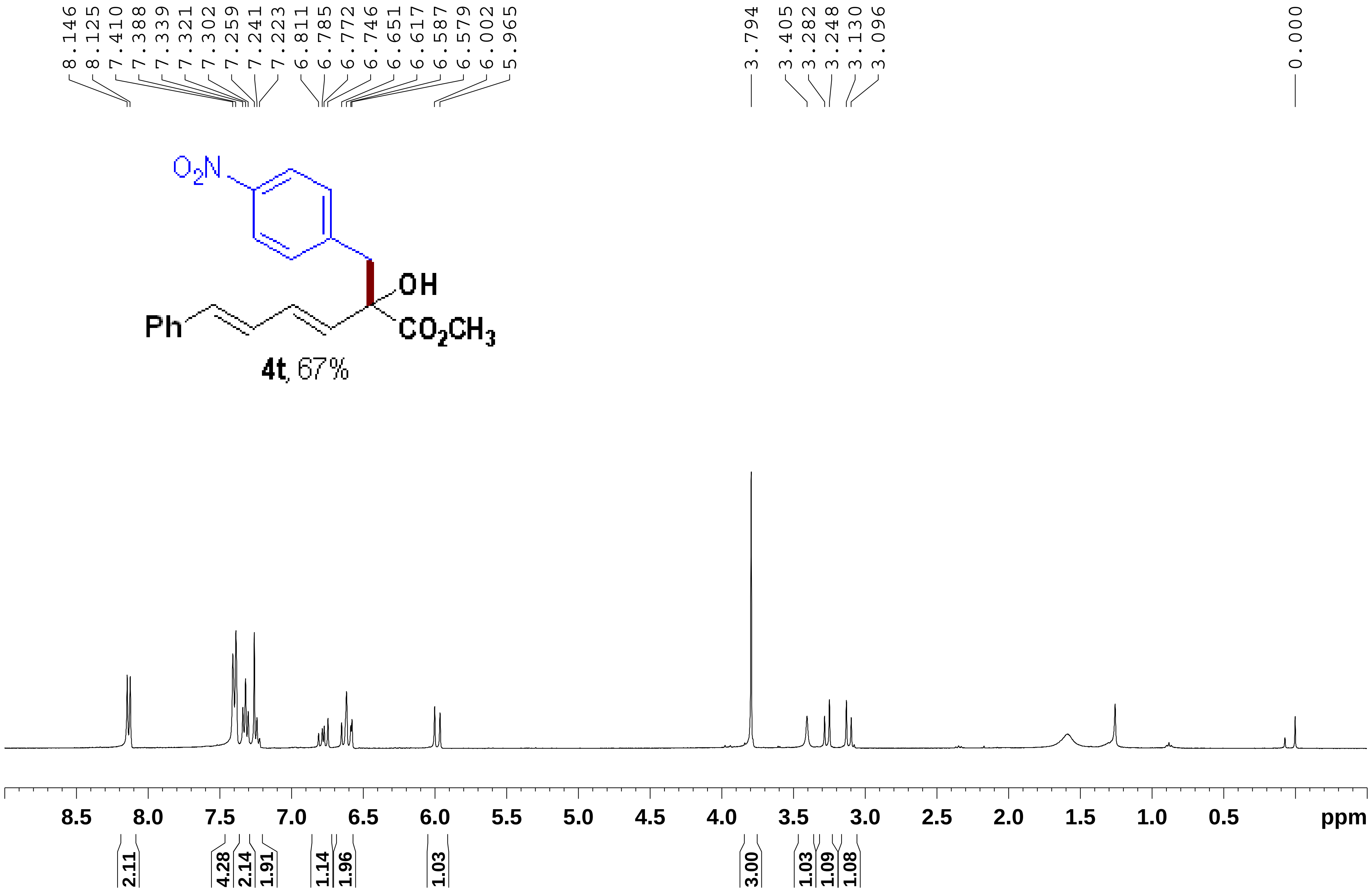

Supplementary Figure 43. ^{13}C NMR Spectrum of substrate 4t

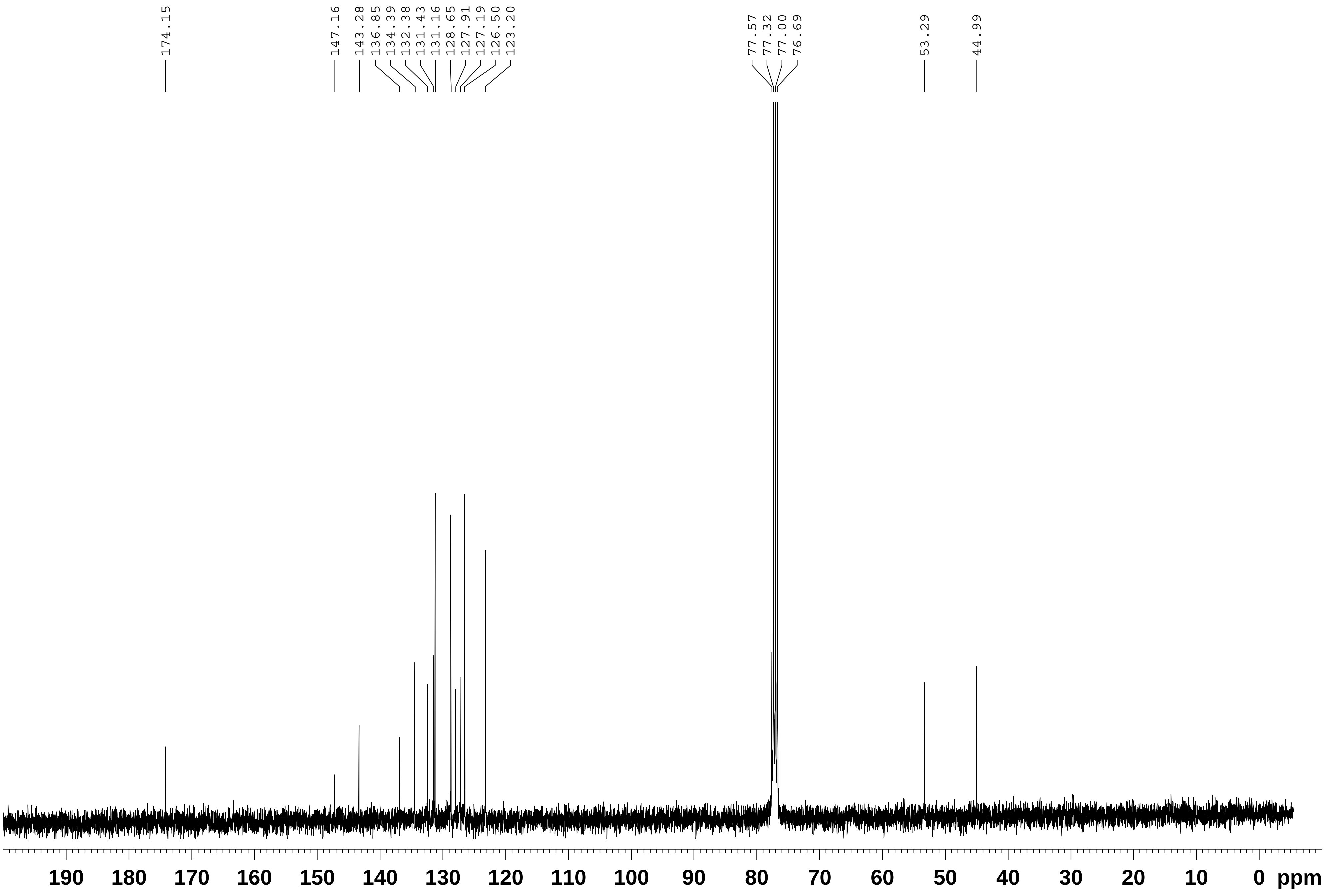

Supplementary Figure 44. ^{1}H NMR Spectrum of substrate 4u

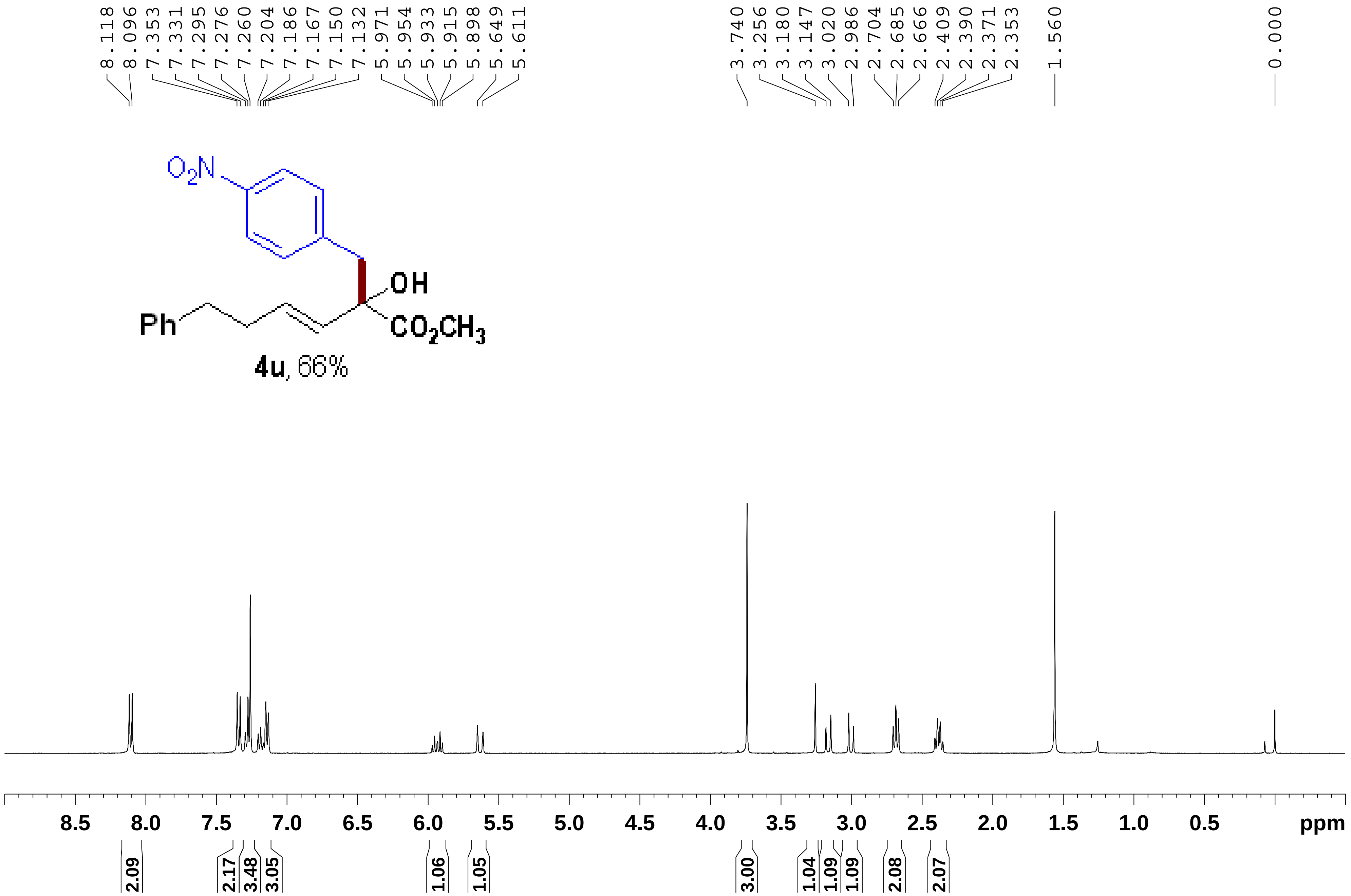

Supplementary Figure 45. ^{13}C NMR Spectrum of substrate 4u

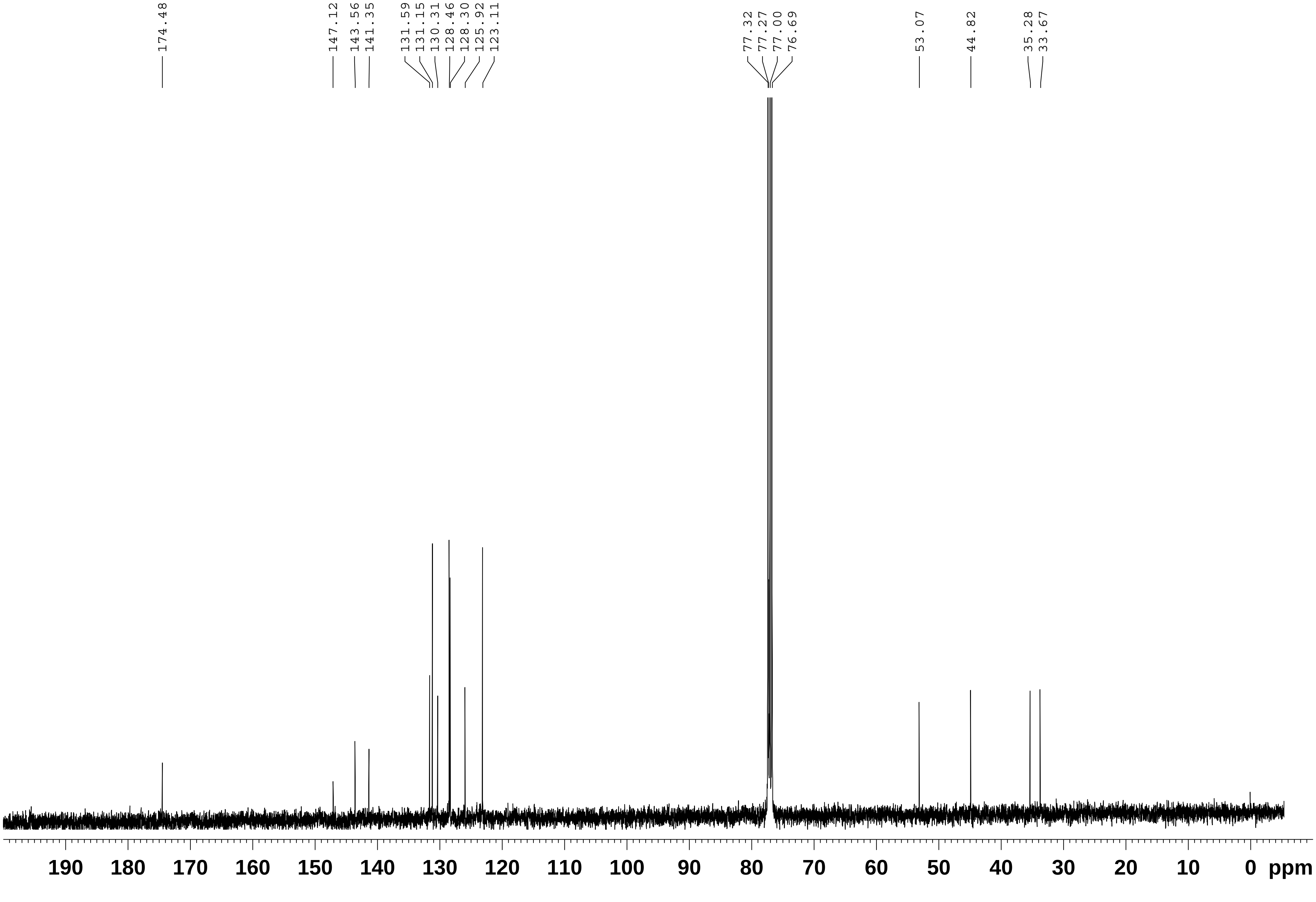

Supplementary Figure 46. ^{1}H NMR Spectrum of substrate 4v

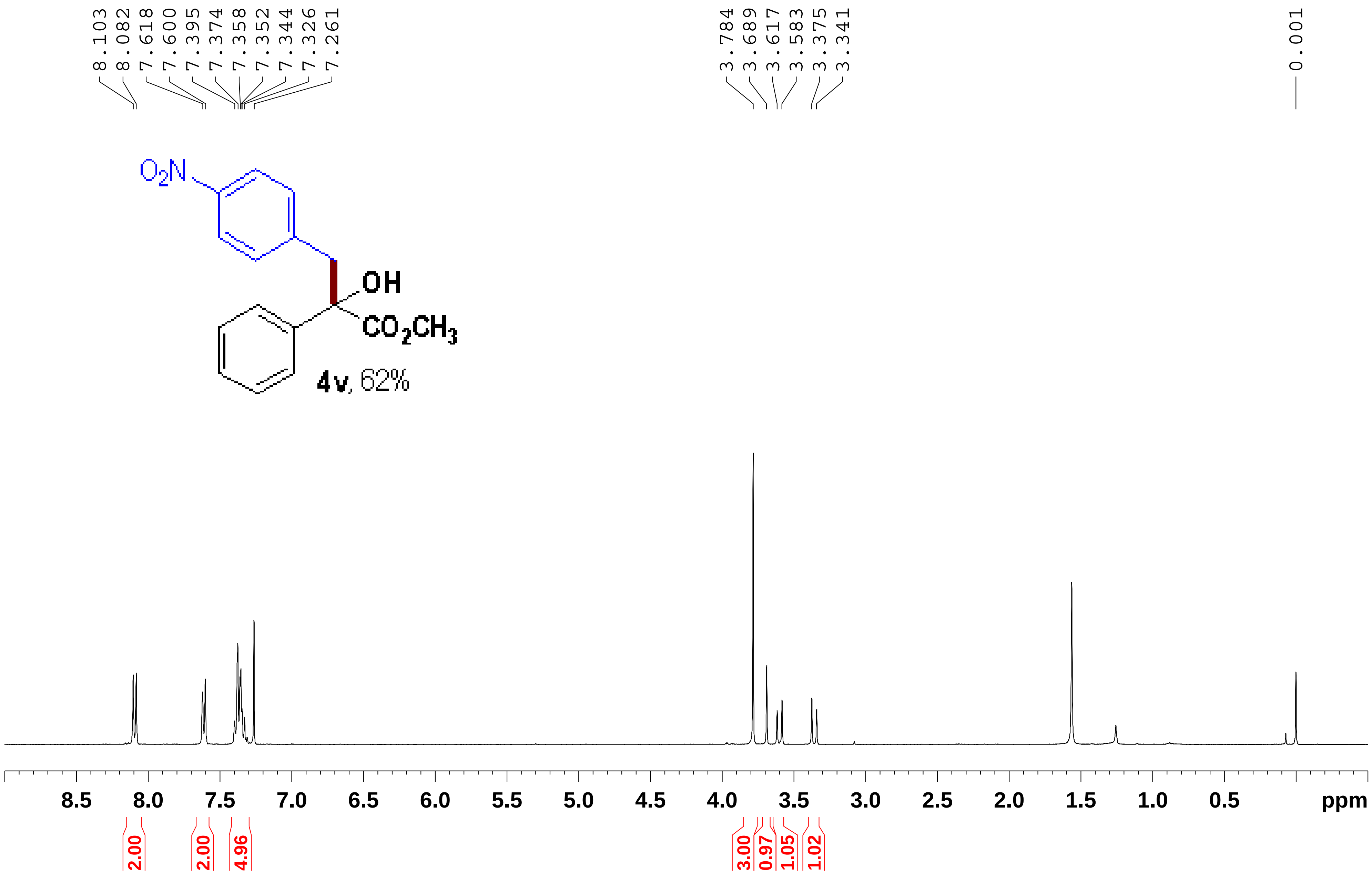

Supplementary Figure 47. ^{13}C NMR Spectrum of substrate 4v

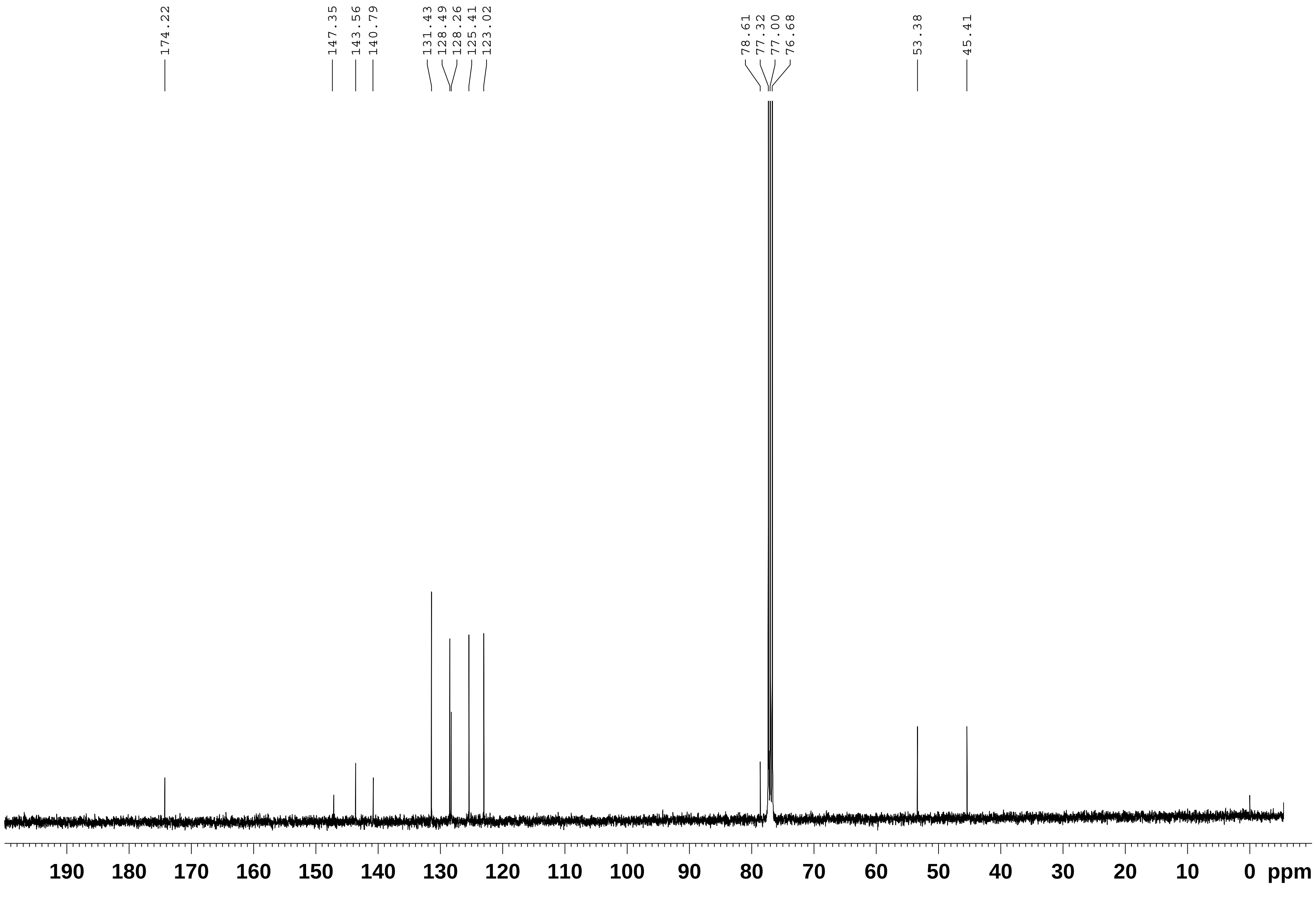

Supplementary Figure 48. ^{1}H NMR Spectrum of substrate 4w

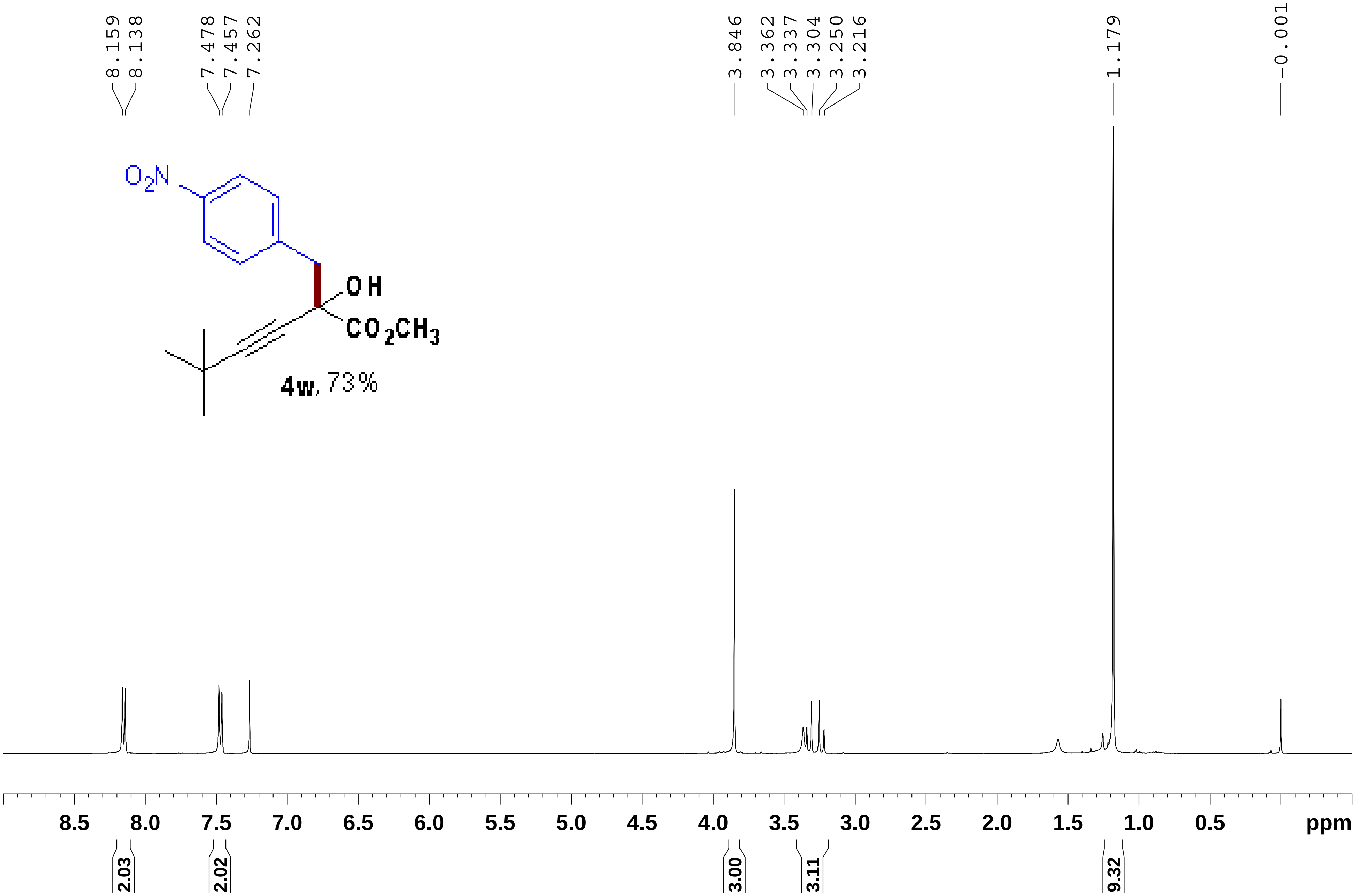

Supplementary Figure 49. ^{13}C NMR Spectrum of substrate 4w

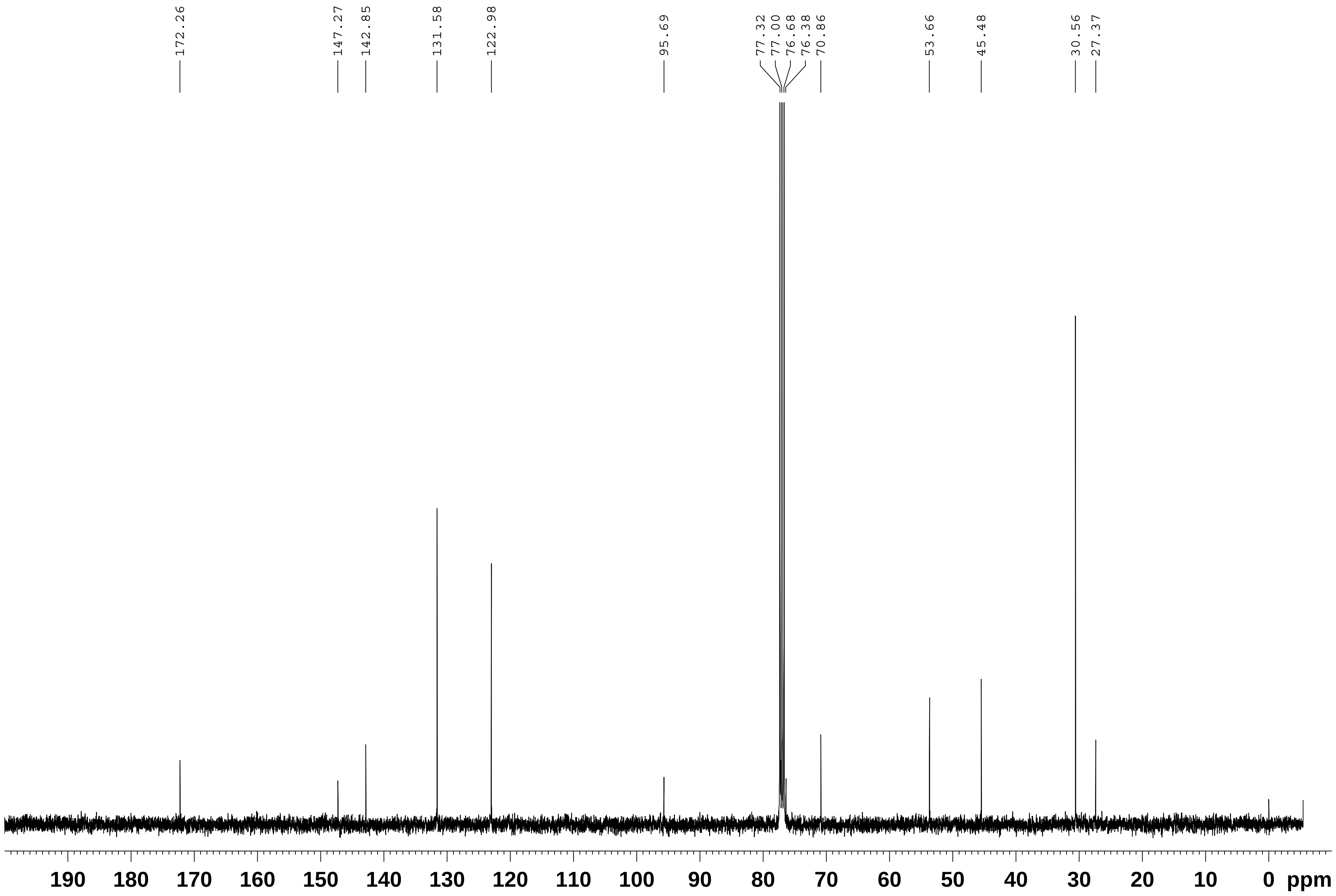

Supplementary Figure 50. ^{1}H NMR Spectrum of substrate 4x

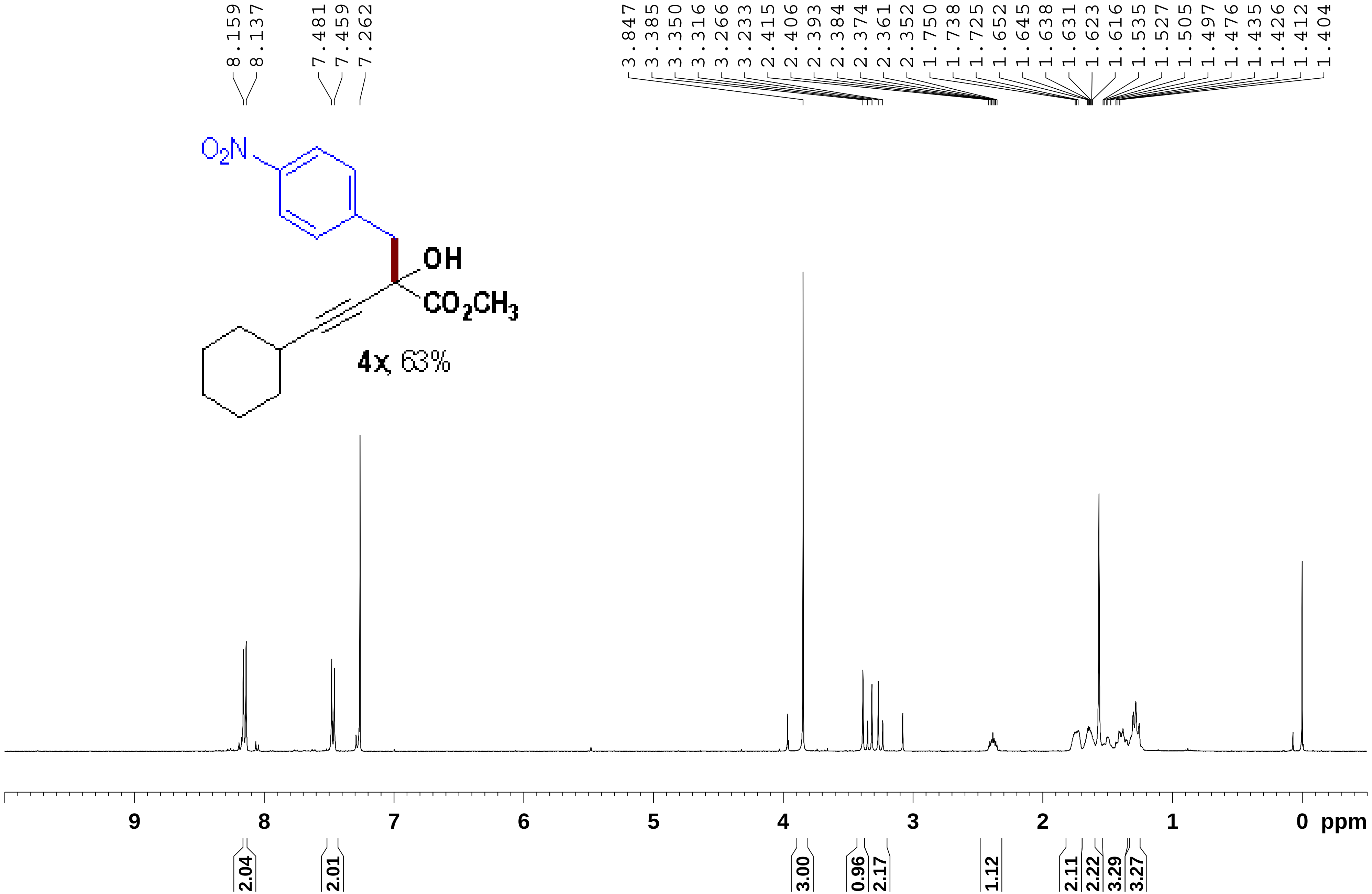

Supplementary Figure 51. ^{13}C NMR Spectrum of substrate 4x

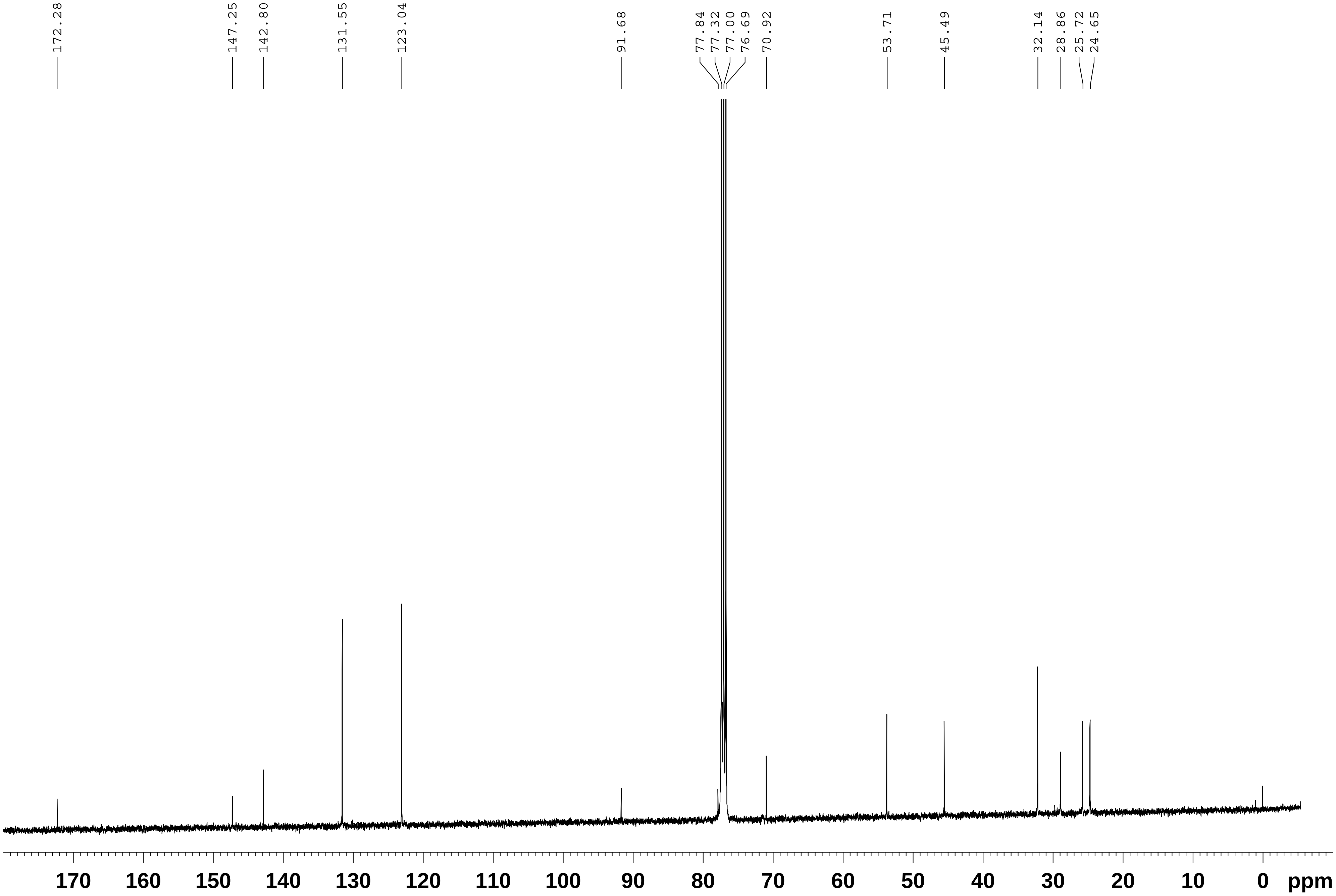

Supplementary Figure 52. ^{1}H NMR Spectrum of substrate 4y

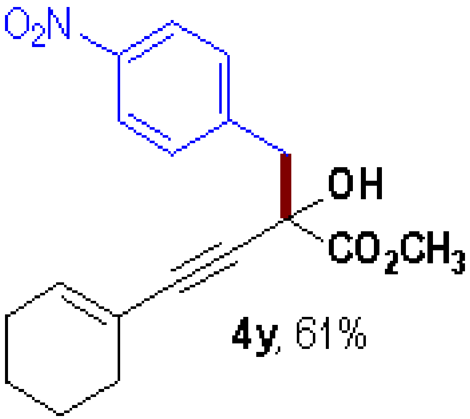

4y, 61%

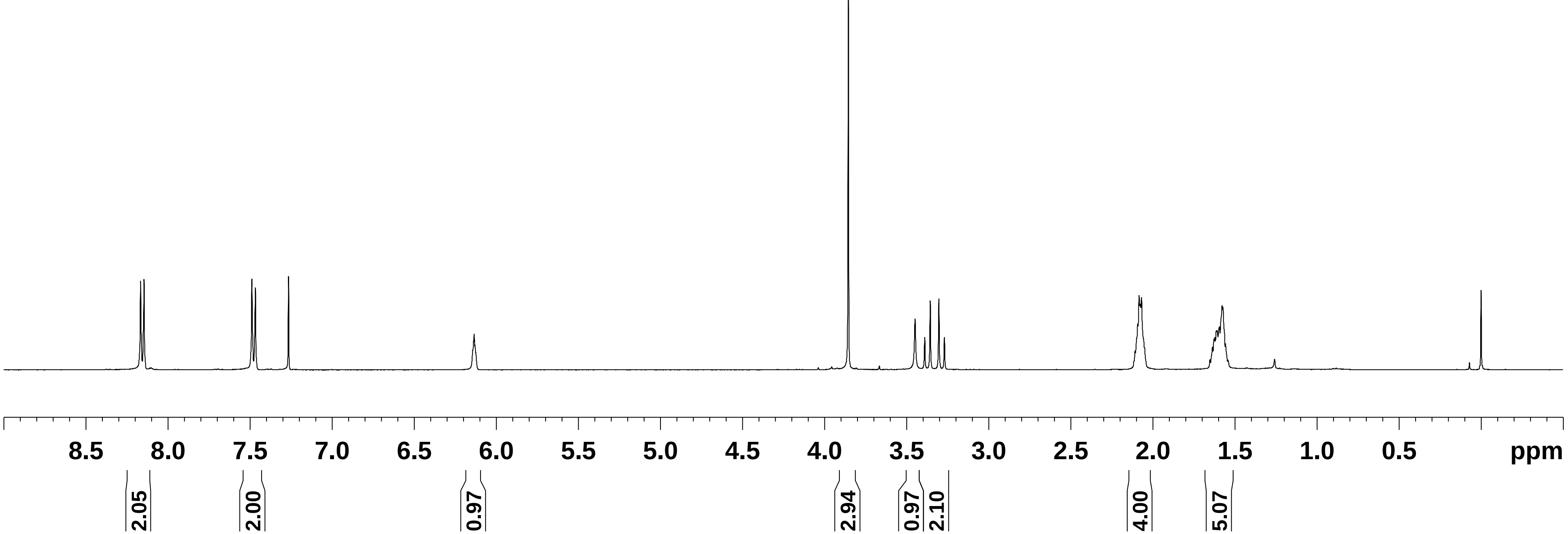

Supplementary Figure 53. ^{13}C NMR Spectrum of substrate 4y

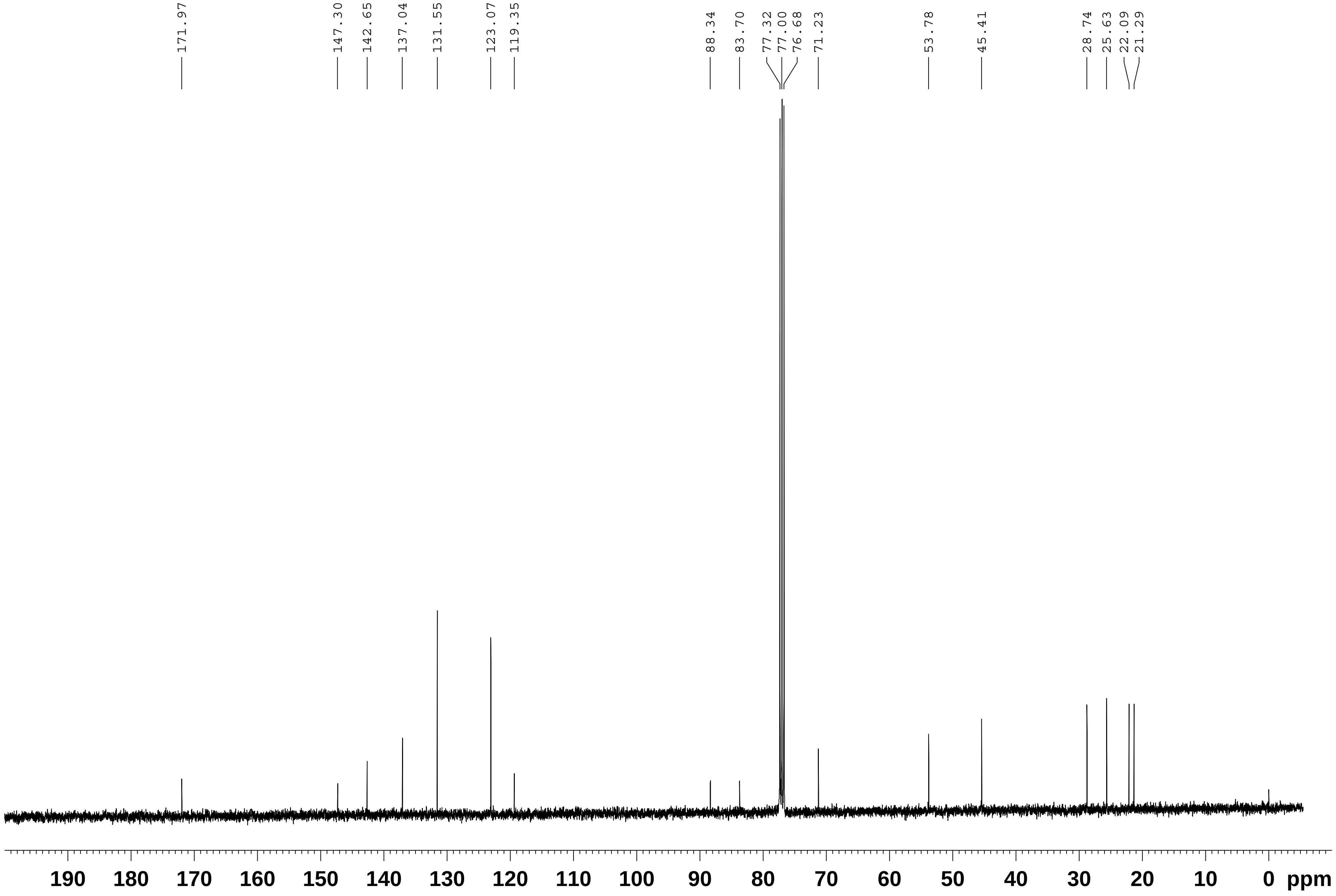

Supplementary Figure 54. ^{1}H NMR Spectrum of substrate 4z

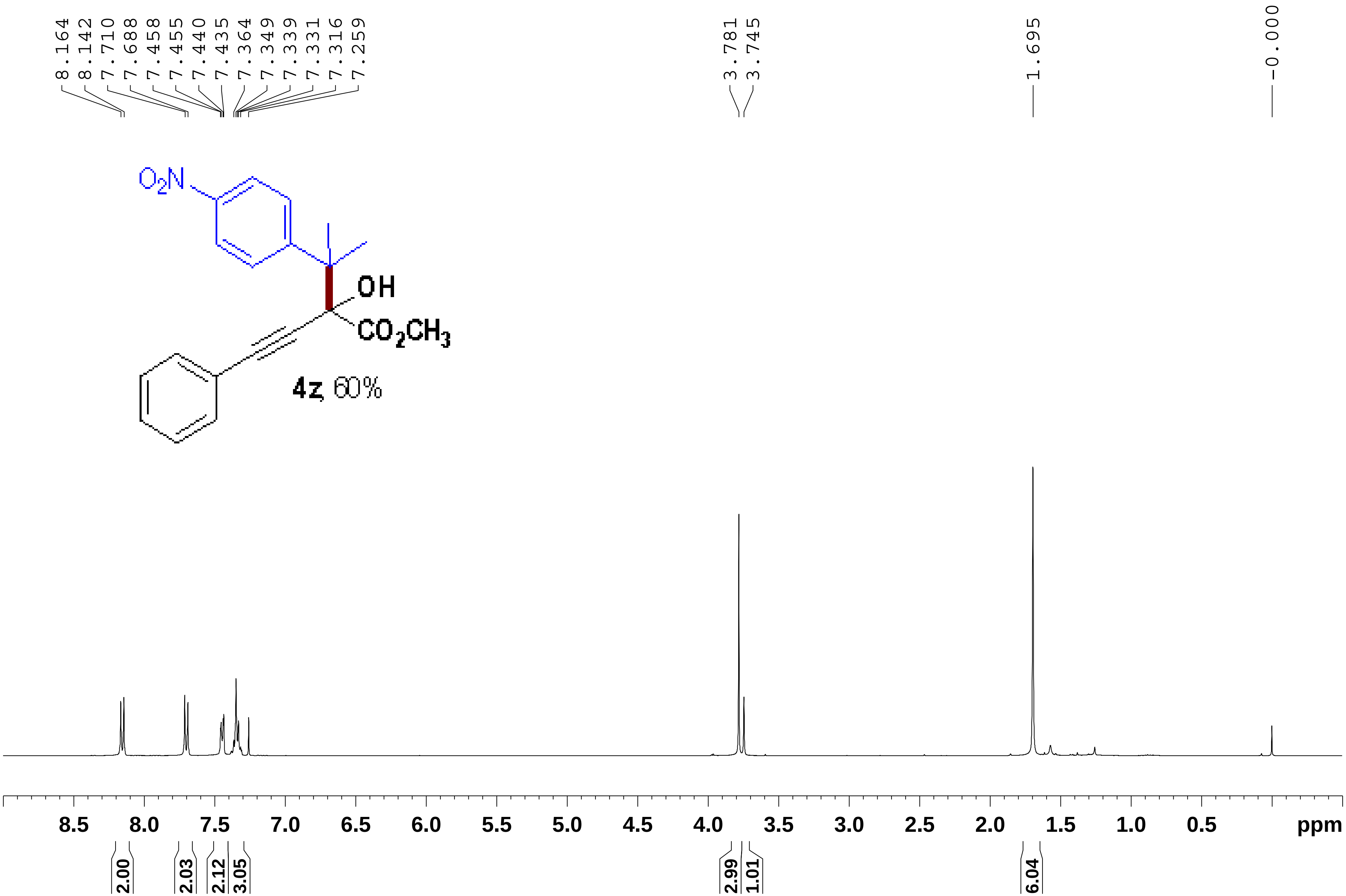

Supplementary Figure 55. ^{13}C NMR Spectrum of substrate 4z

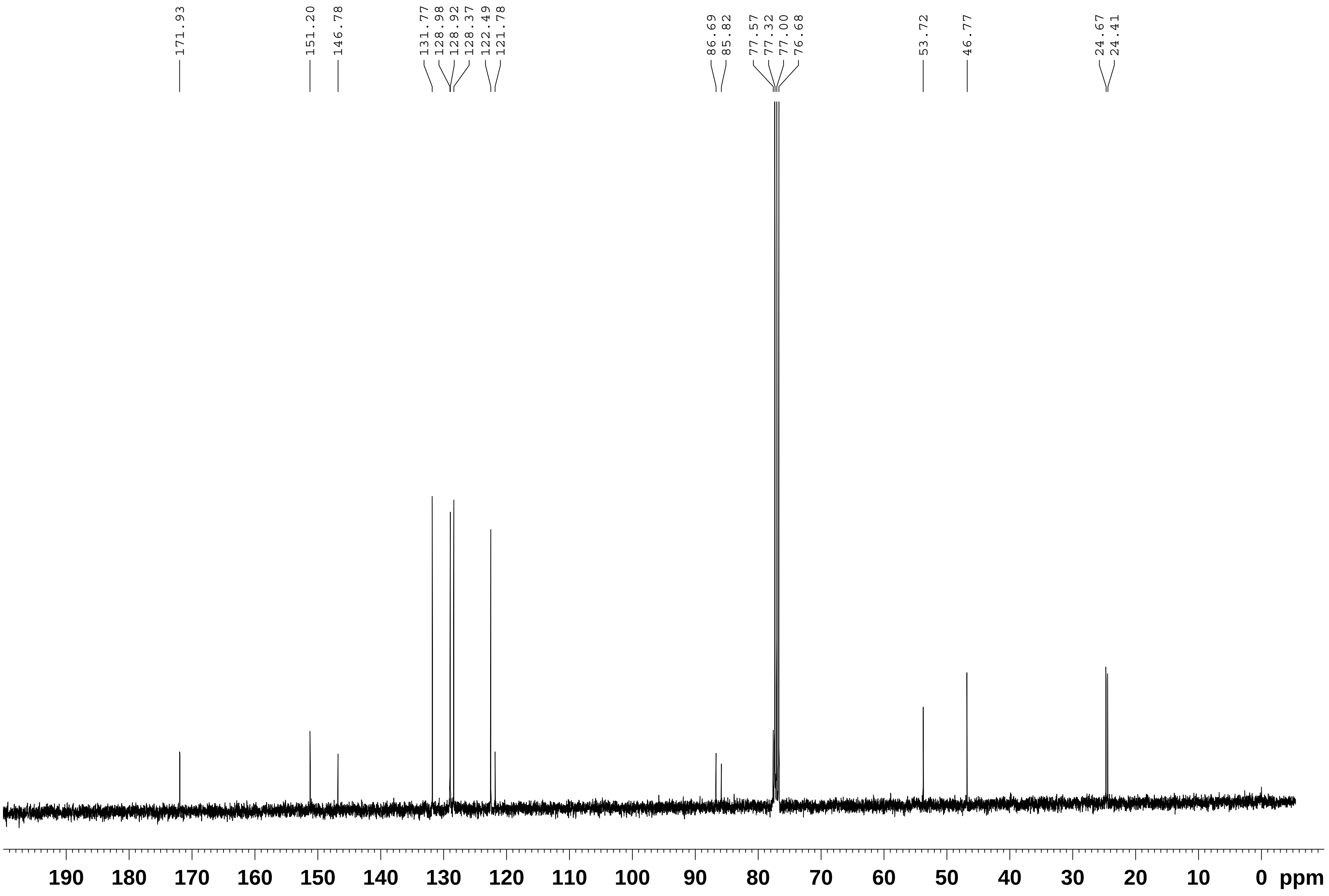

Supplementary Figure 56. ^{1}H NMR Spectrum of substrate 7a

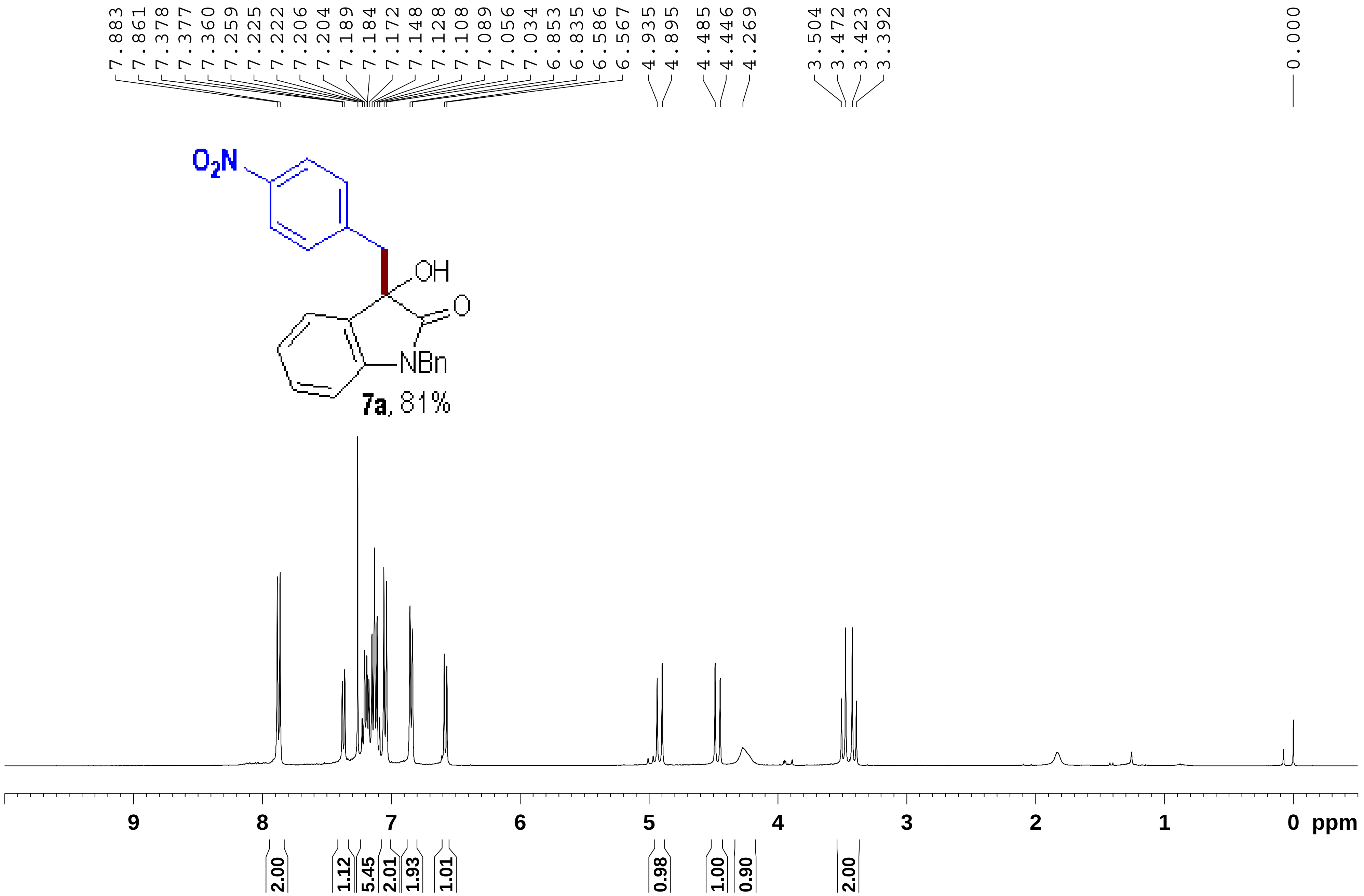

Supplementary Figure 57. ^{13}C NMR Spectrum of substrate 7a

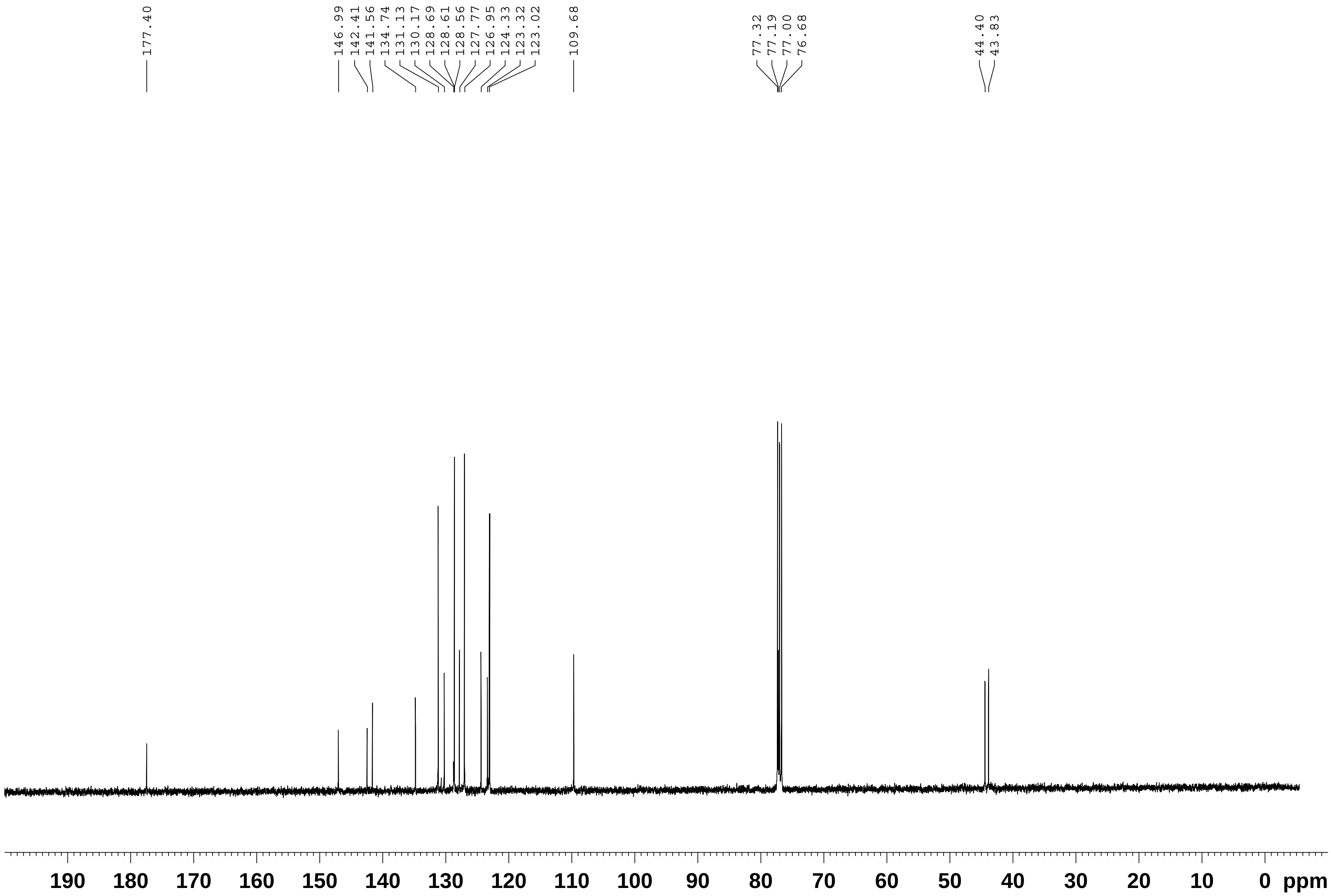

Supplementary Figure 58. ^{1}H NMR Spectrum of substrate 7b

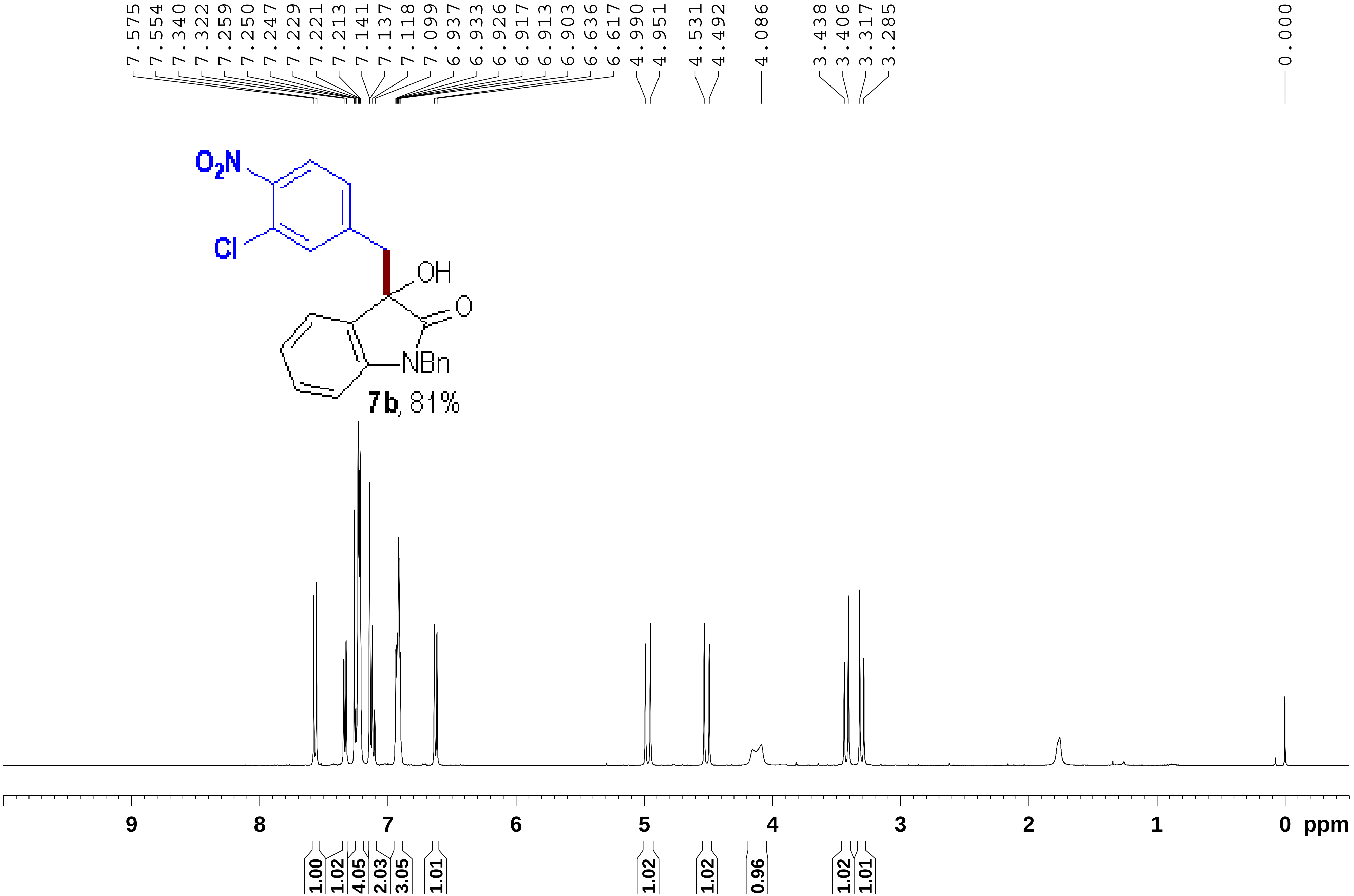

Supplementary Figure 59. ^{13}C NMR Spectrum of substrate 7b

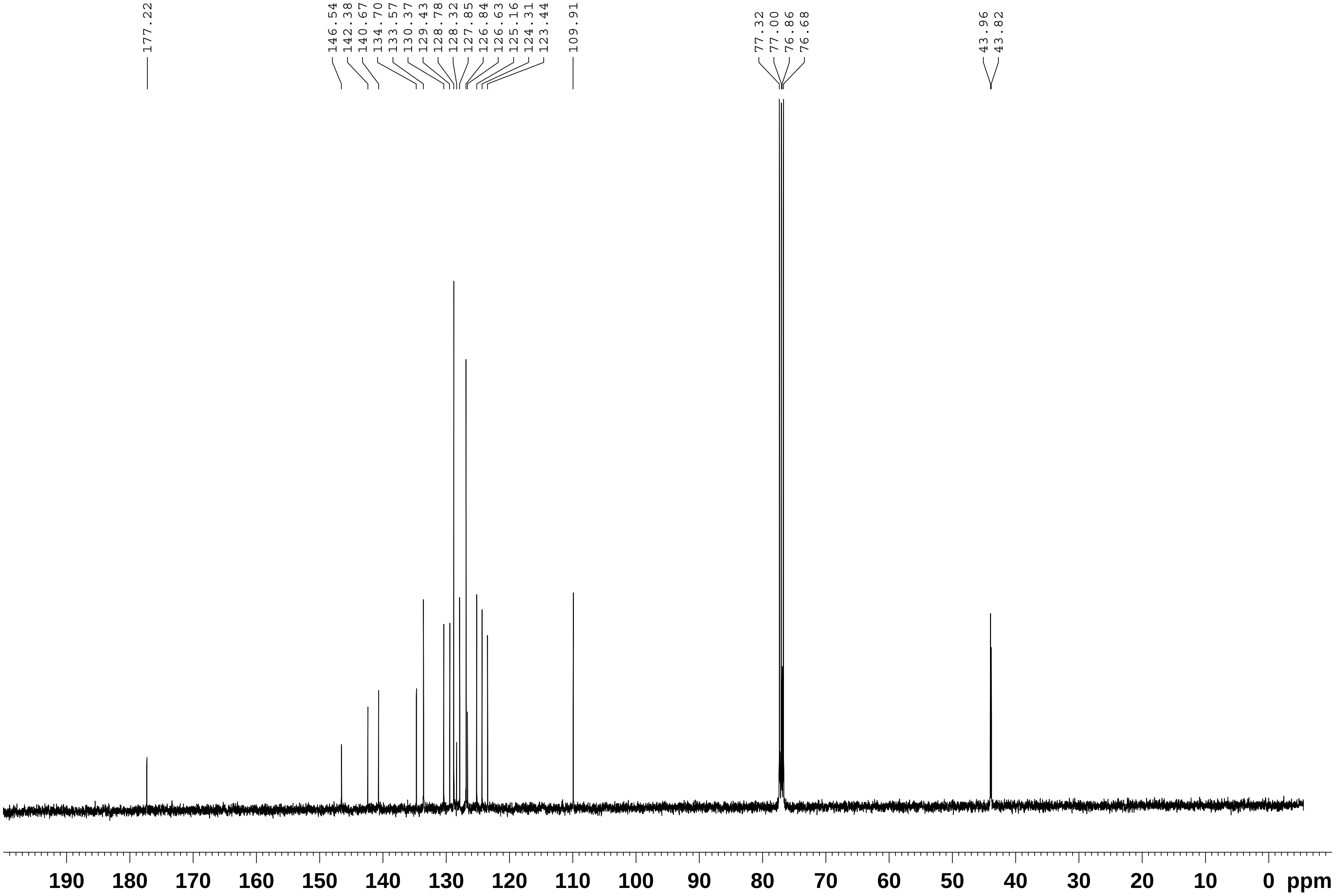

Supplementary Figure 60. ^{1}H NMR Spectrum of substrate 7c

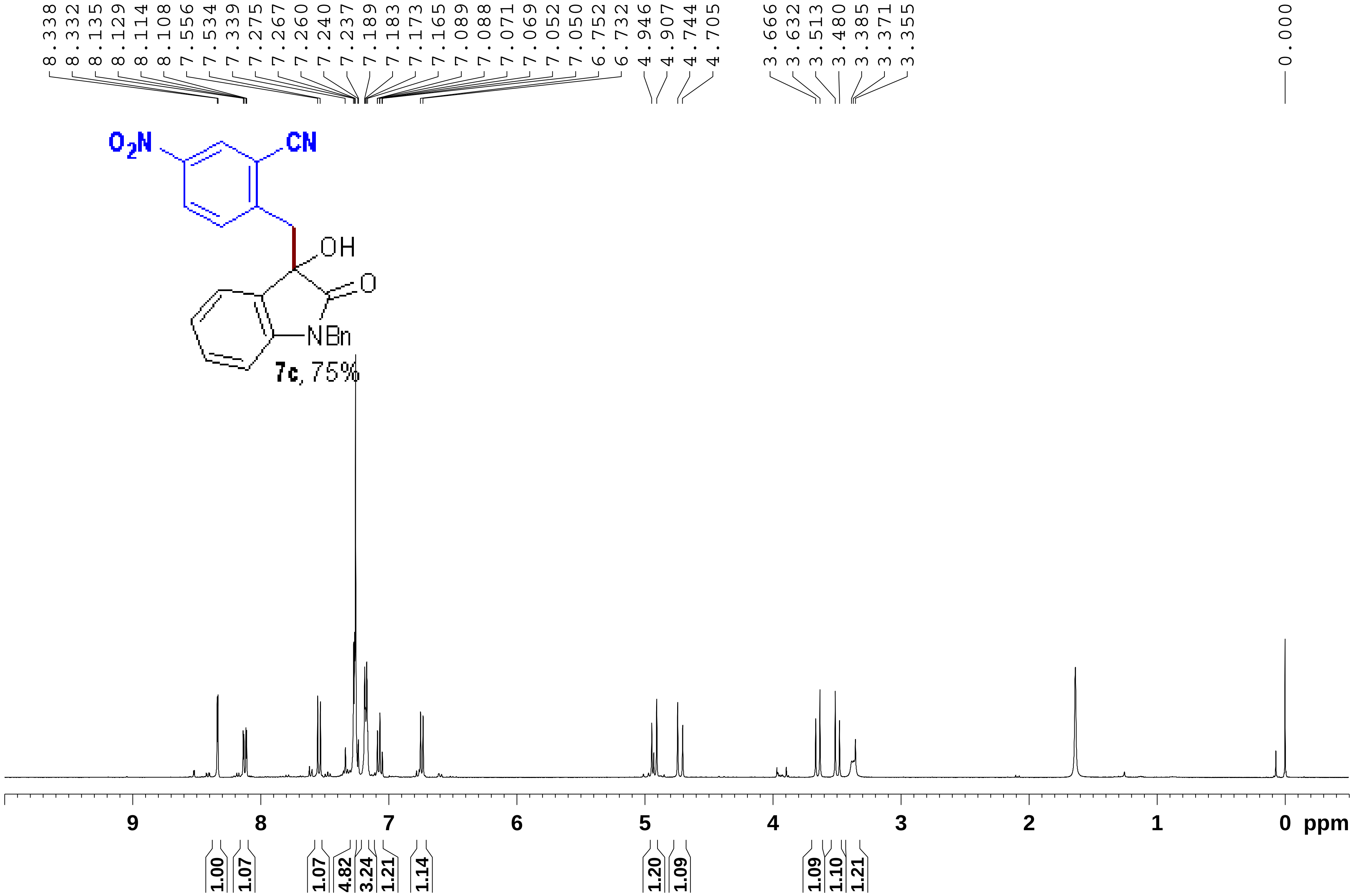

Supplementary Figure 61. ^{13}C NMR Spectrum of substrate 7c

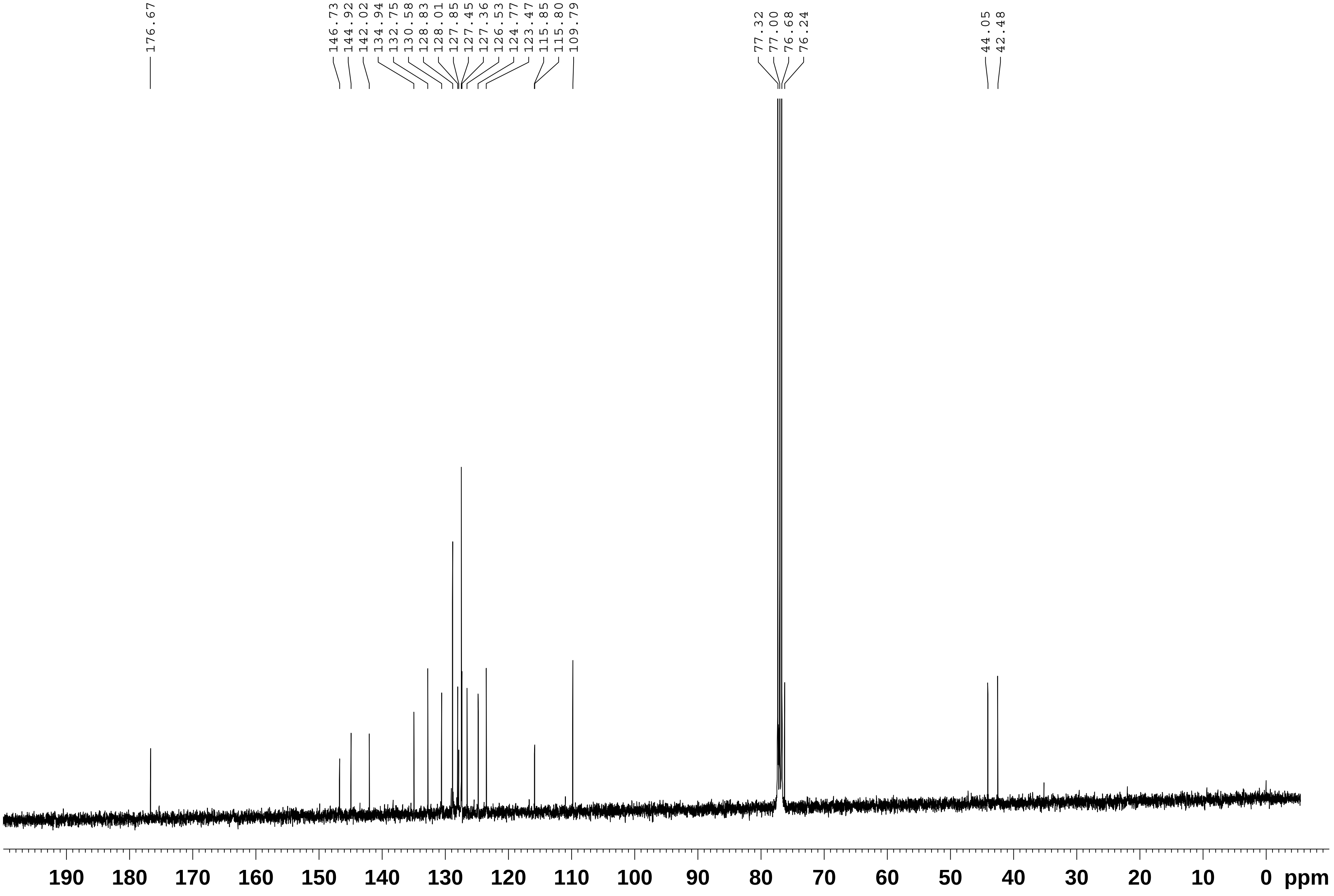

Supplementary Figure 62. ^{1}H NMR Spectrum of substrate 7d

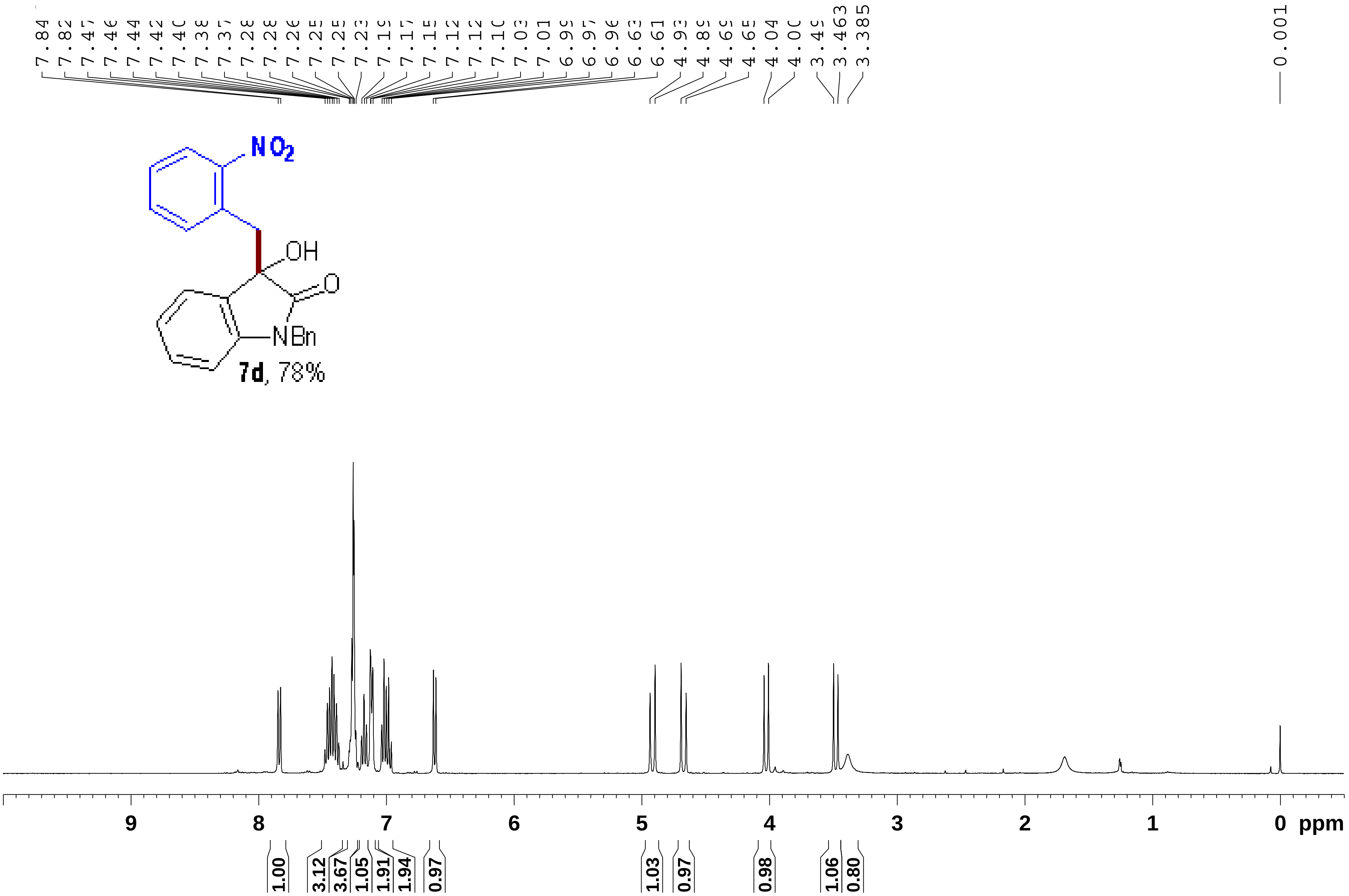

Supplementary Figure 63. ^{13}C NMR Spectrum of substrate 7d

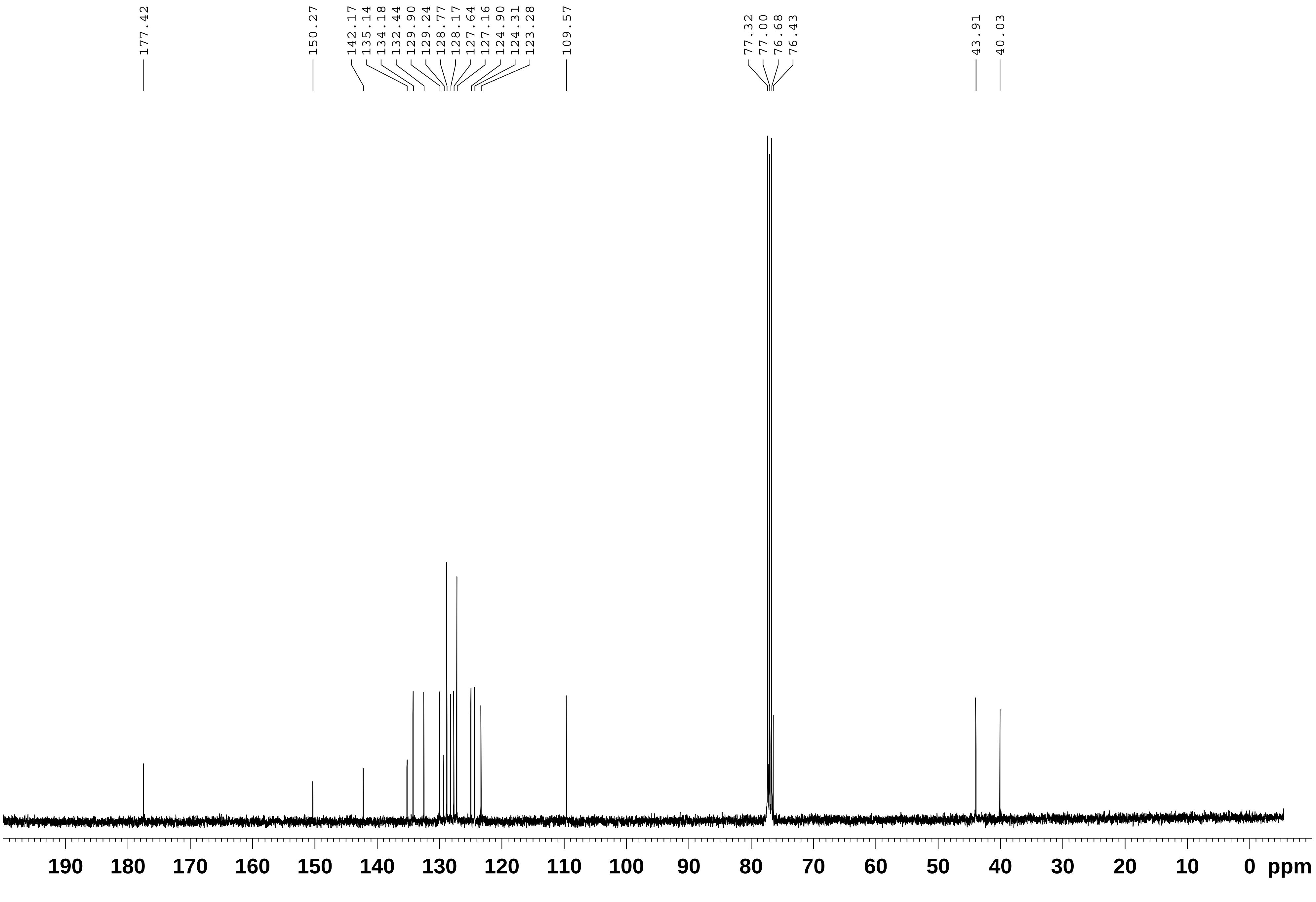

Supplementary Figure 64. ^{1}H NMR Spectrum of substrate 7e

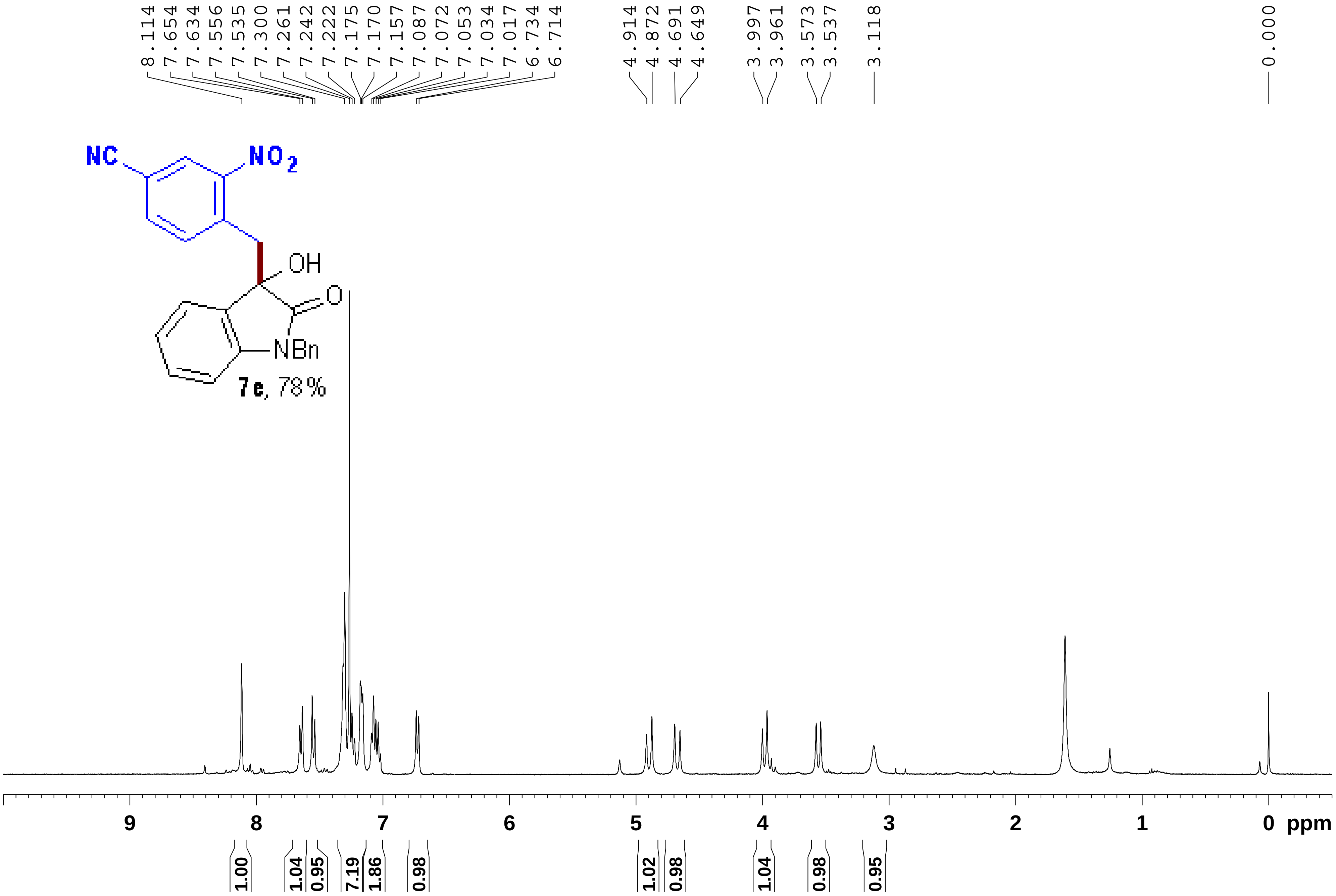

Supplementary Figure 65. ^{13}C NMR Spectrum of substrate 7e

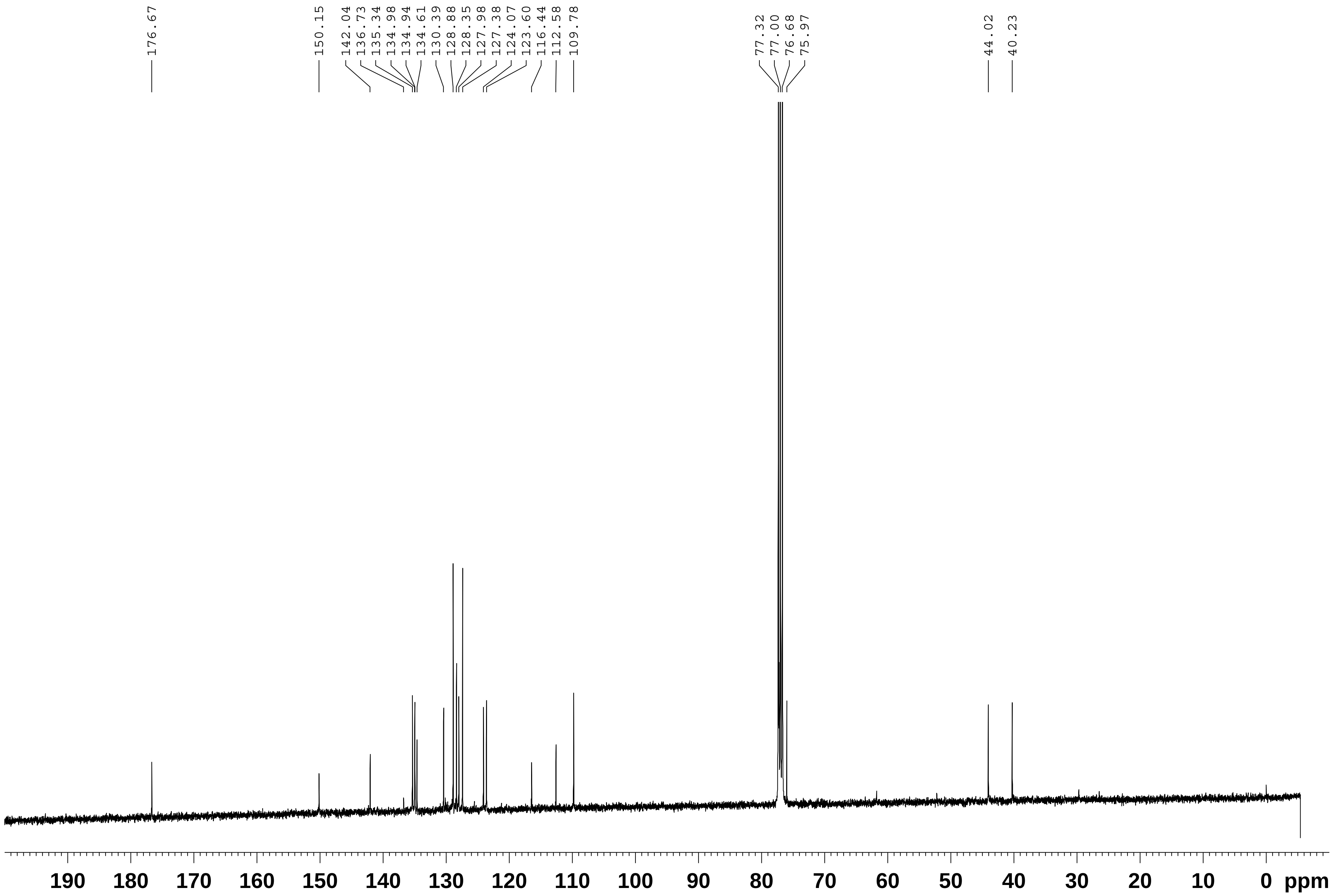

Supplementary Figure 66. ¹H NMR Spectrum of substrate 7f

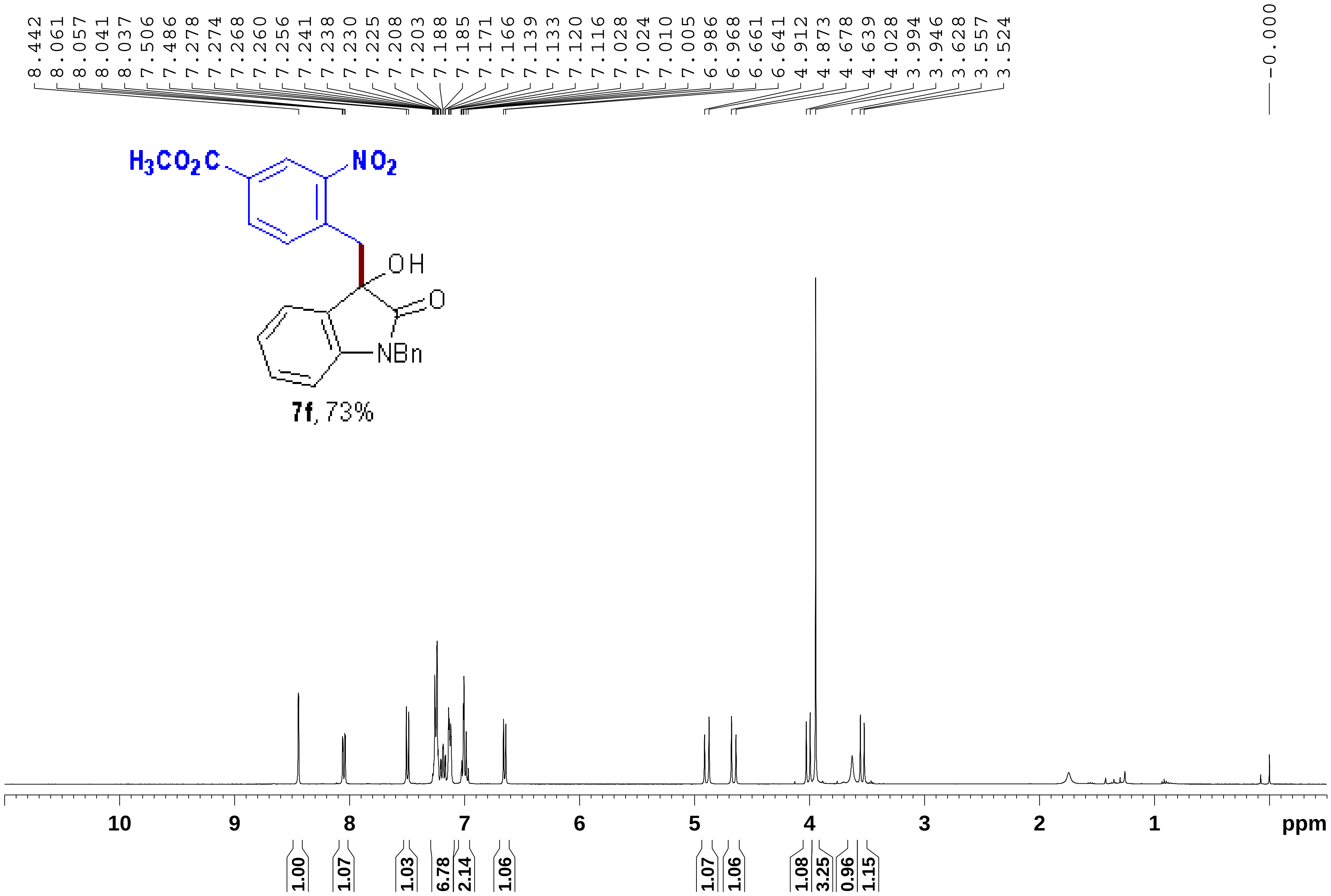

Supplementary Figure 67. ^{13}C NMR Spectrum of substrate 7f

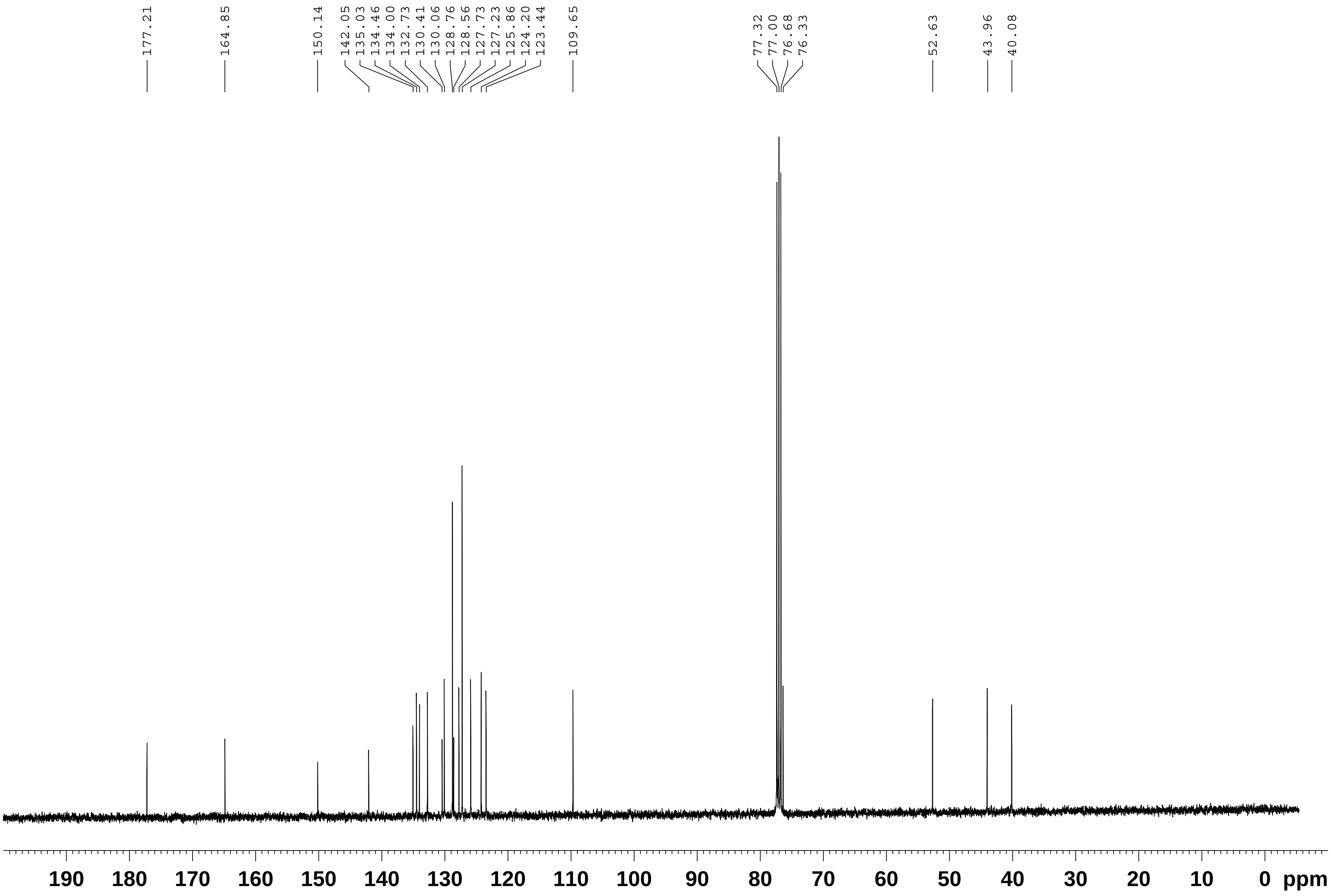

Supplementary Figure 68. ^{1}H NMR Spectrum of substrate 7g

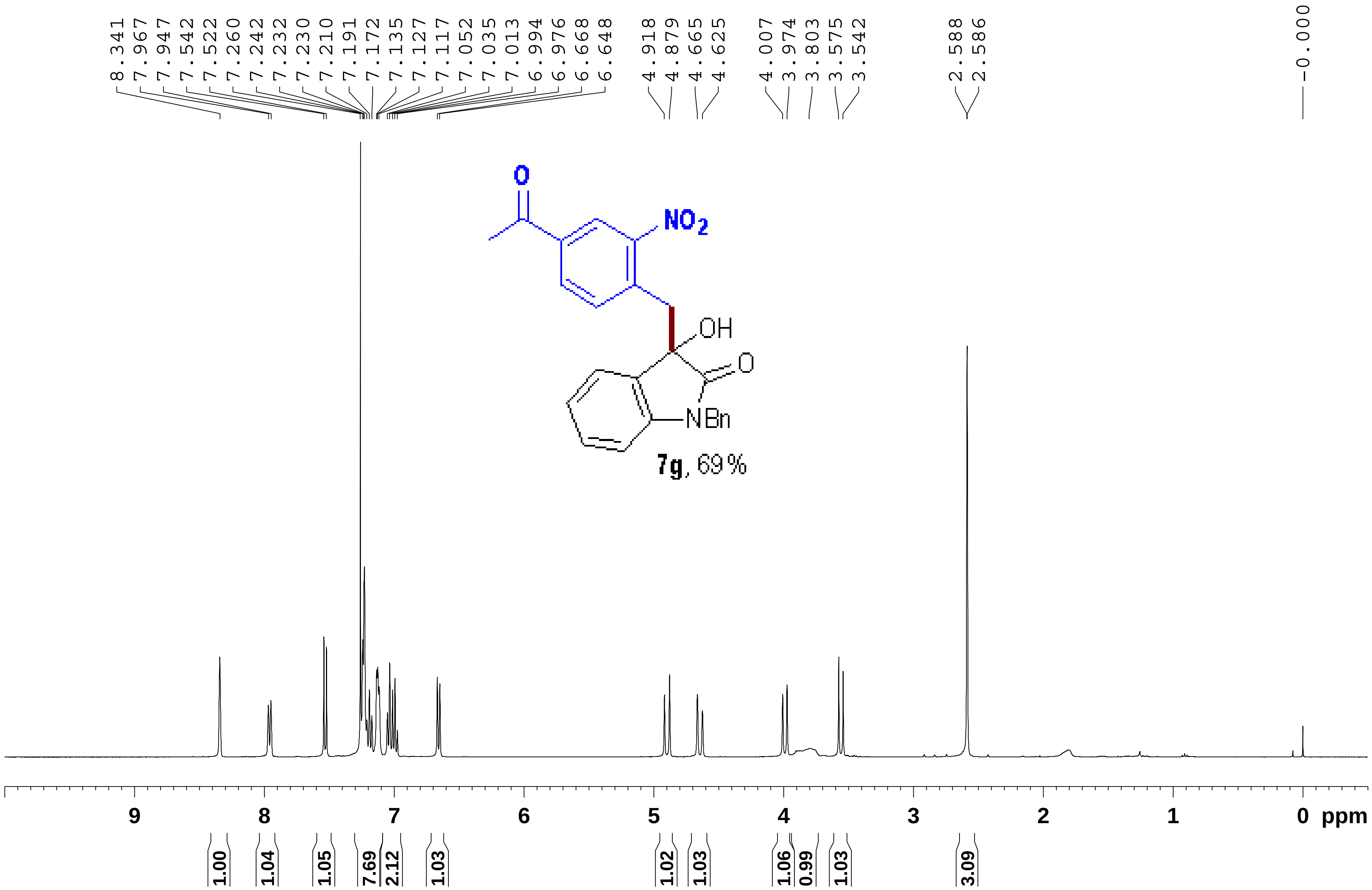

Supplementary Figure 69. ^{13}C NMR Spectrum of substrate 7g

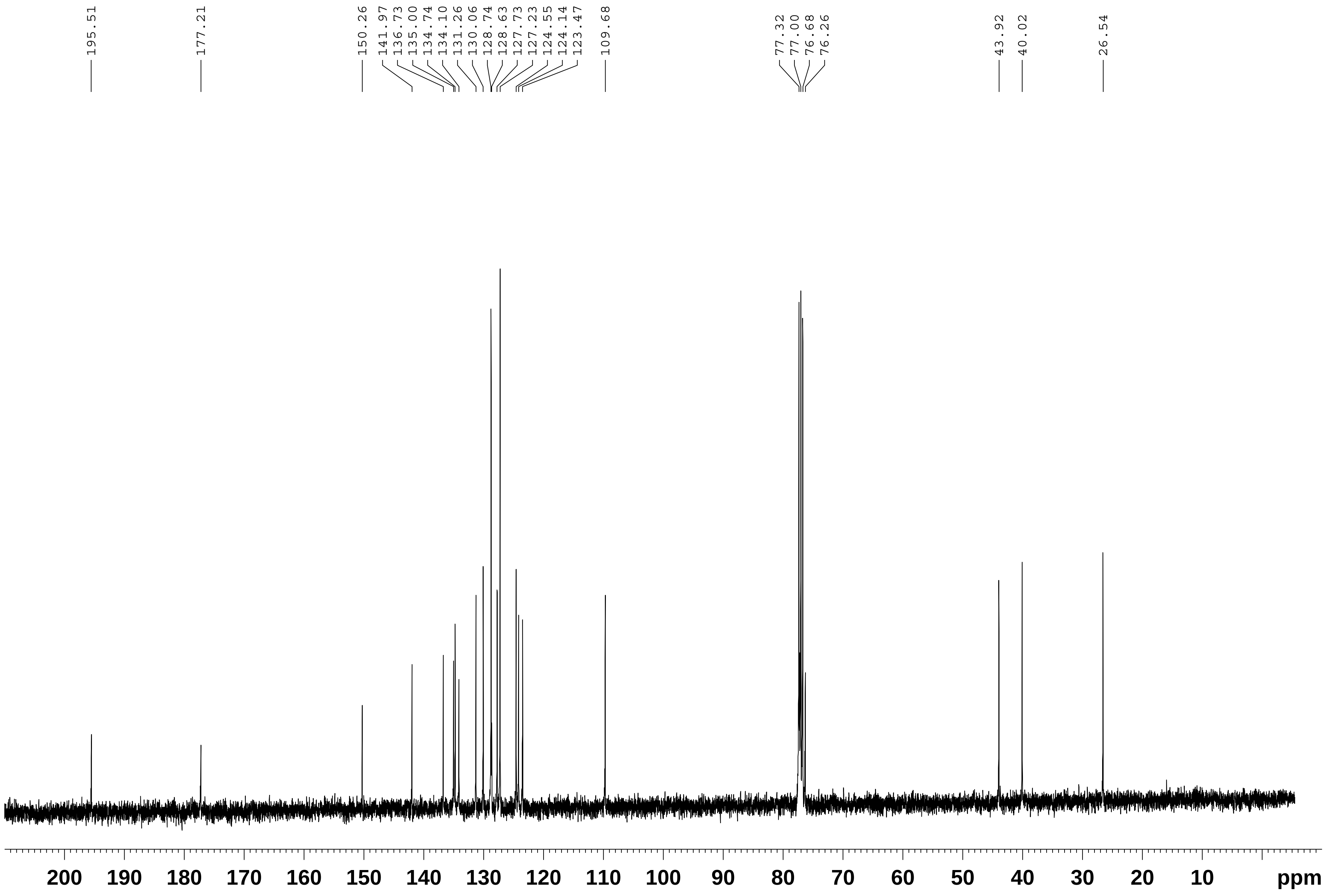

Supplementary Figure 70. ^{1}H NMR Spectrum of substrate 7h

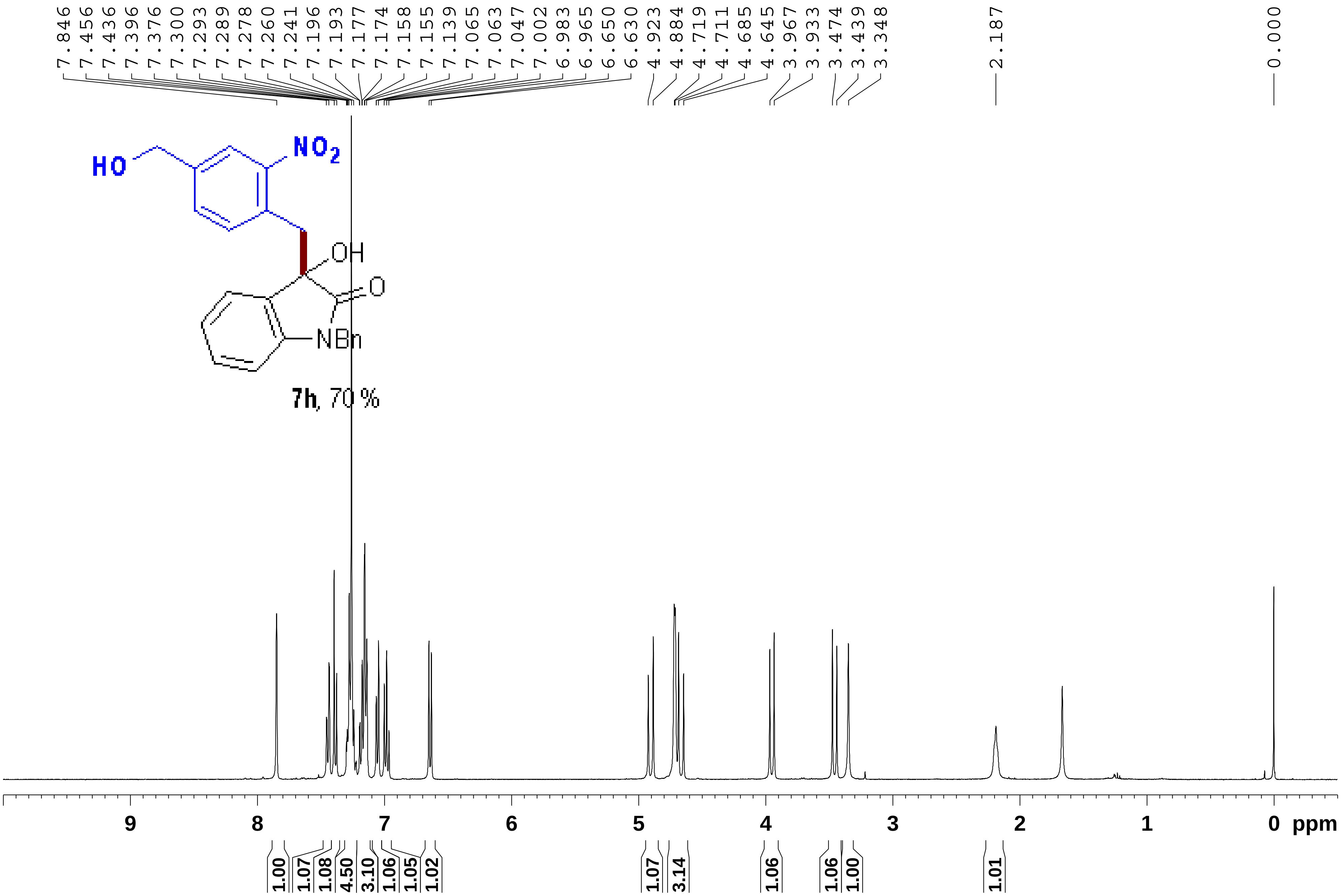

Supplementary Figure 71. ^{13}C NMR Spectrum of substrate 7h

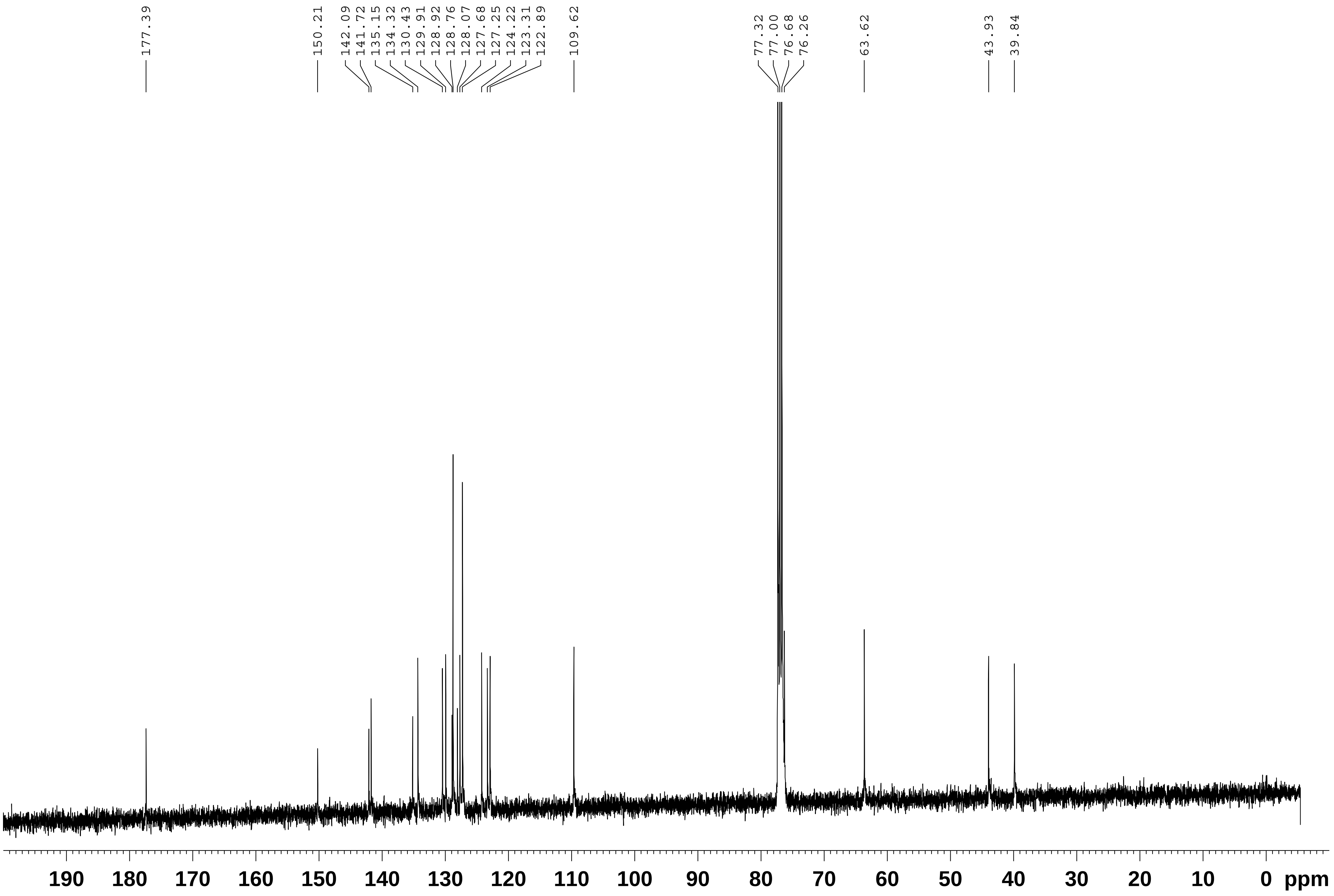

Supplementary Figure 72. ^{1}H NMR Spectrum of substrate 7i

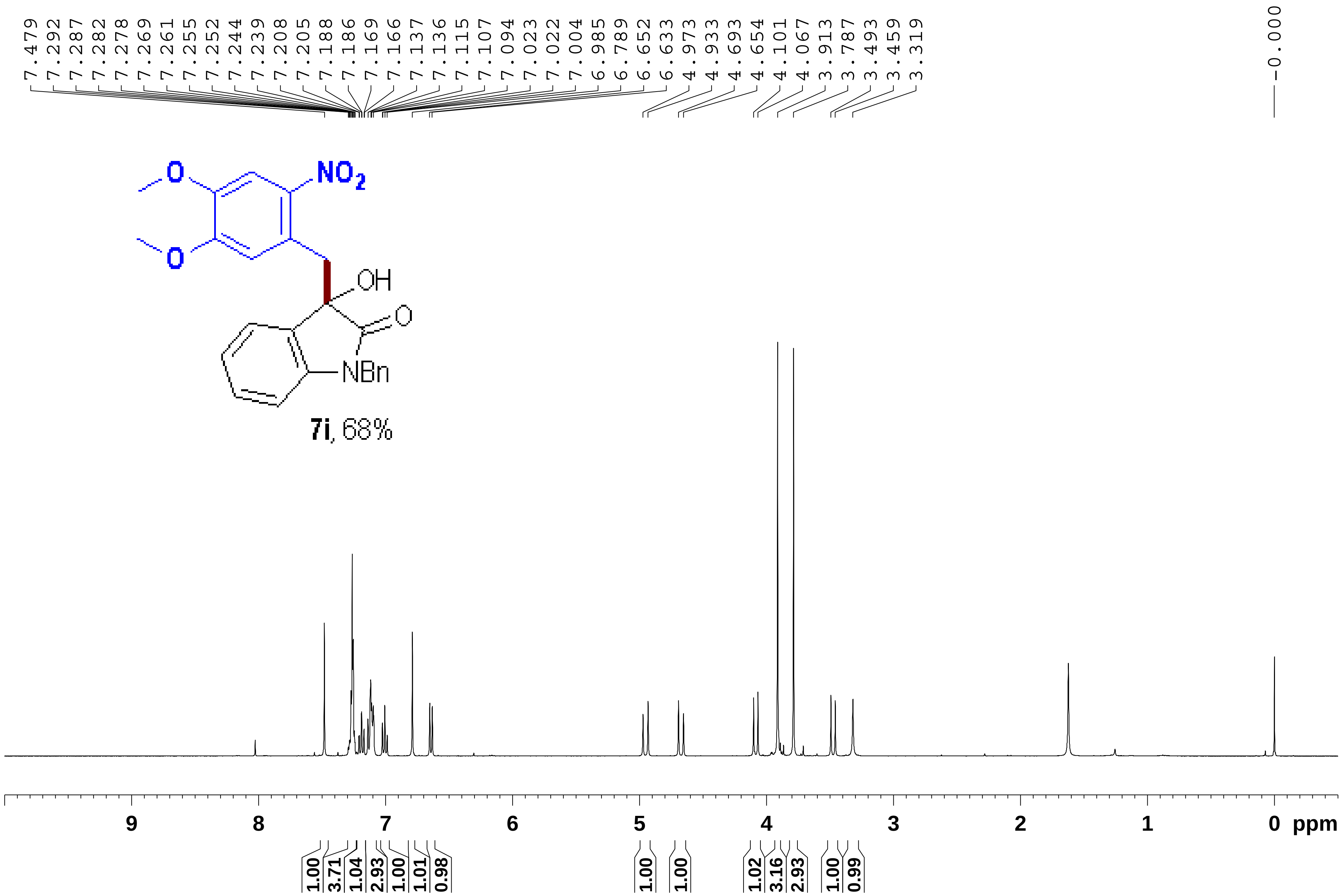

Supplementary Figure 73. ^{13}C NMR Spectrum of substrate 7i

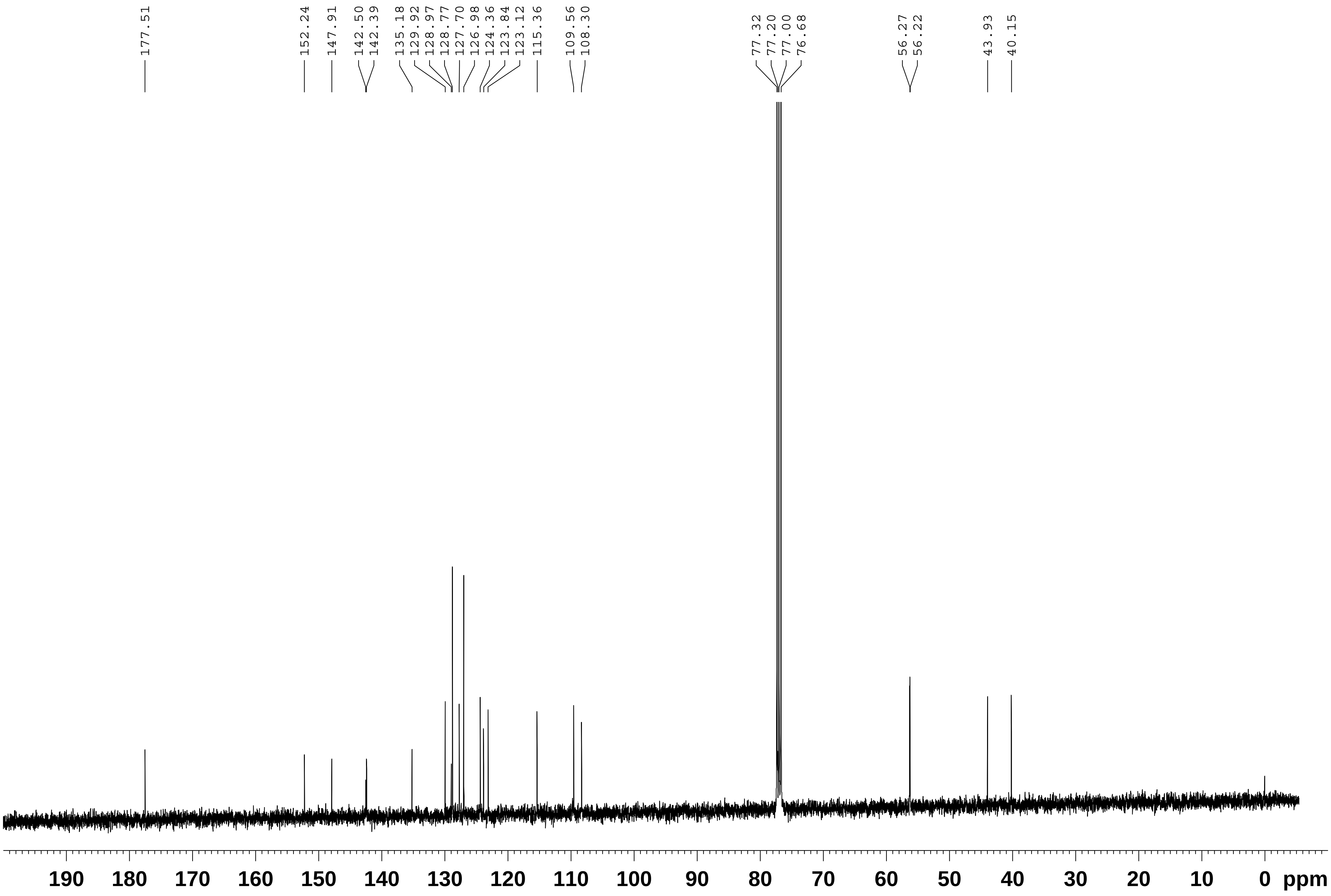

Supplementary Figure 74. ^{1}H NMR Spectrum of substrate 7j

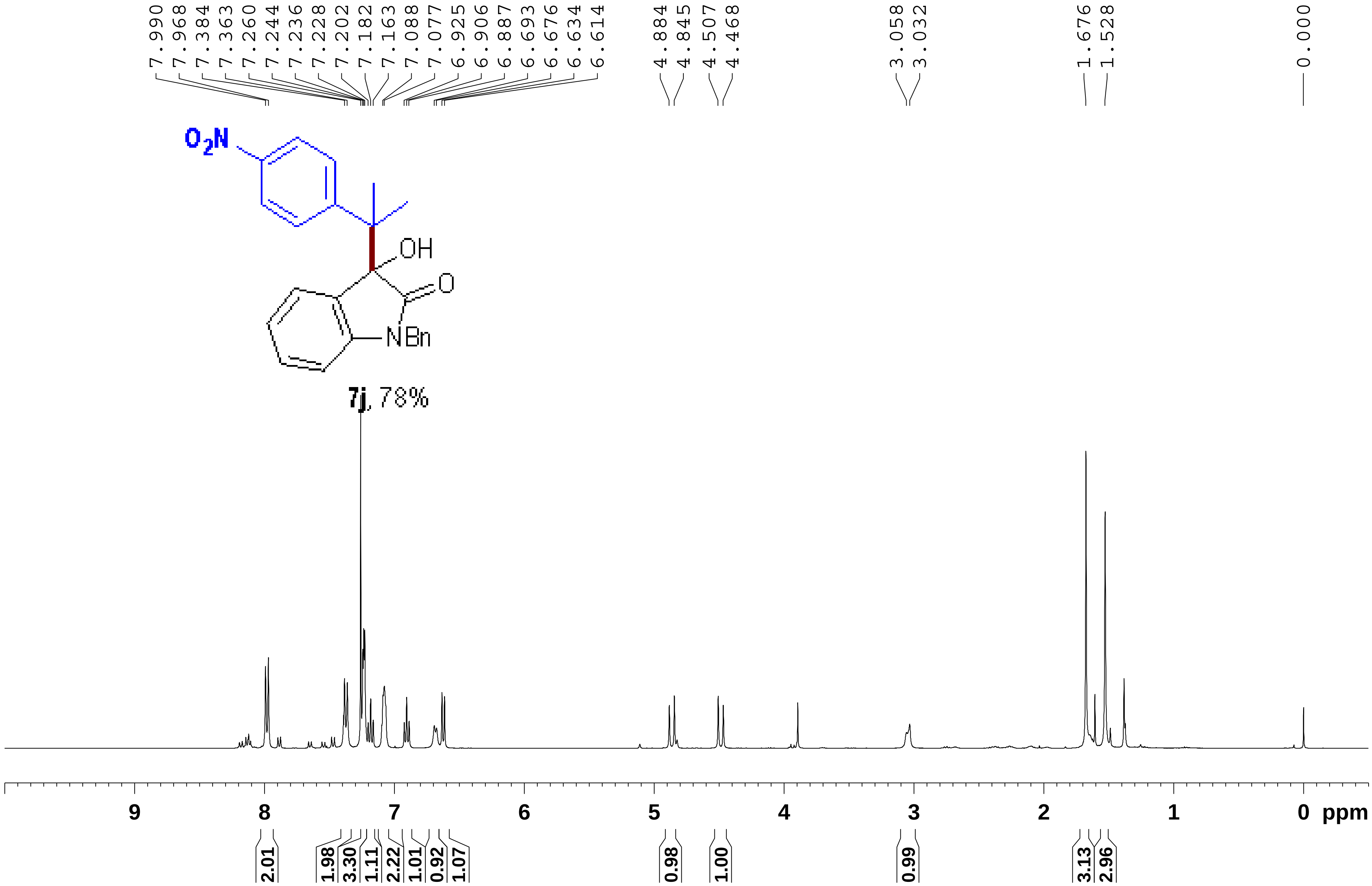

Supplementary Figure 75. ^{13}C NMR Spectrum of substrate 7j

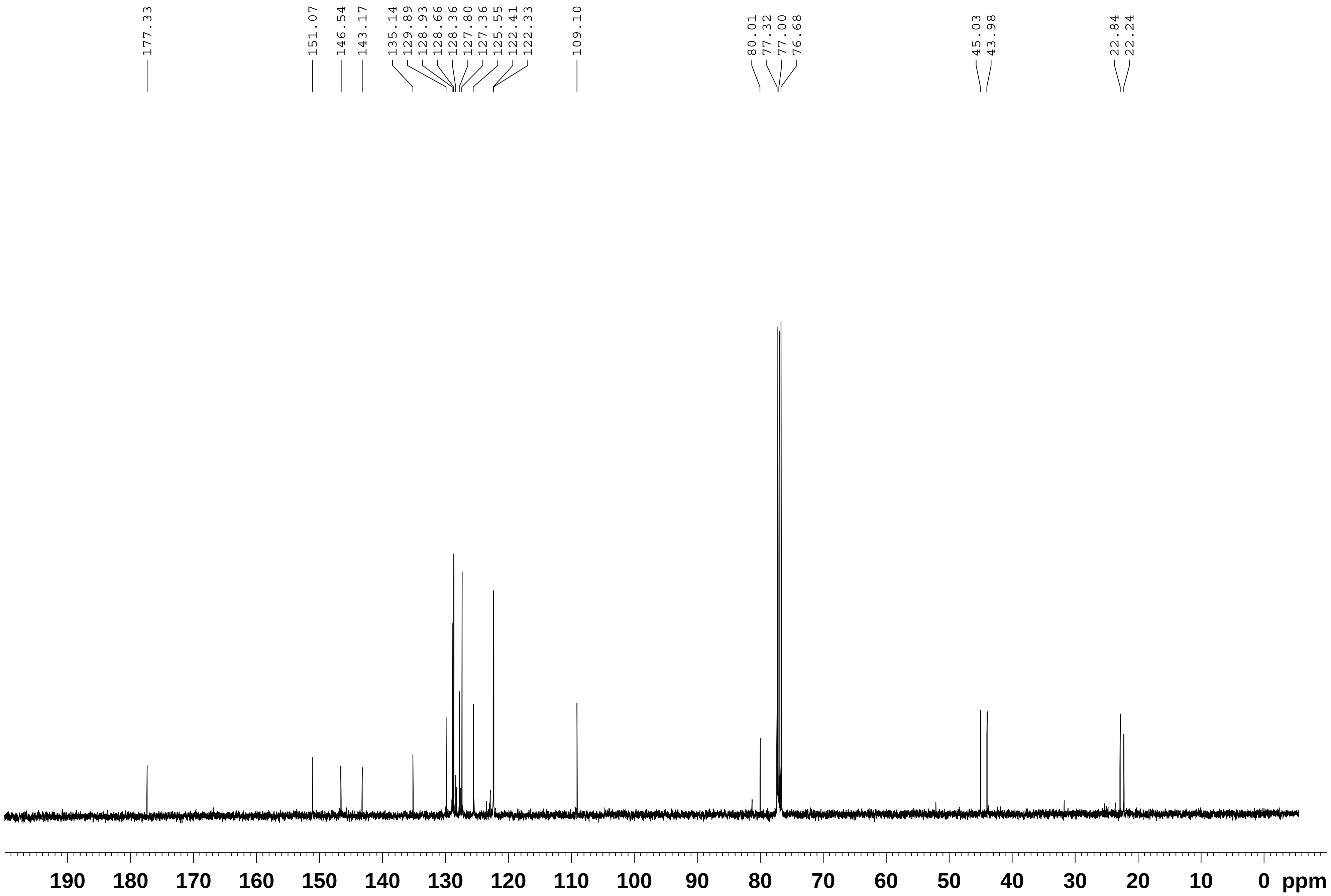

Supplementary Figure 76. ^{1}H NMR Spectrum of substrate 7k

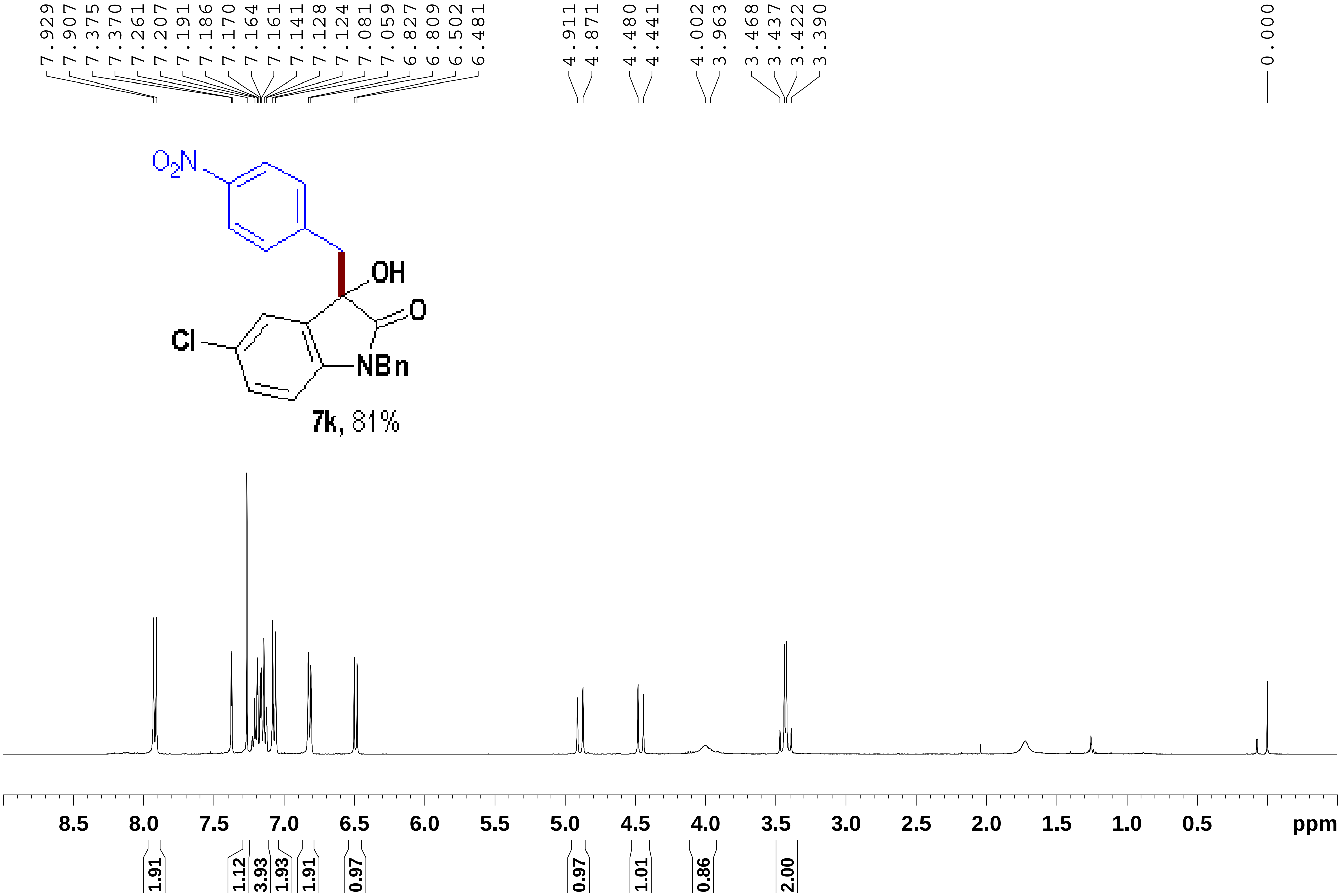

Supplementary Figure 77. ^{13}C NMR Spectrum of substrate 7k

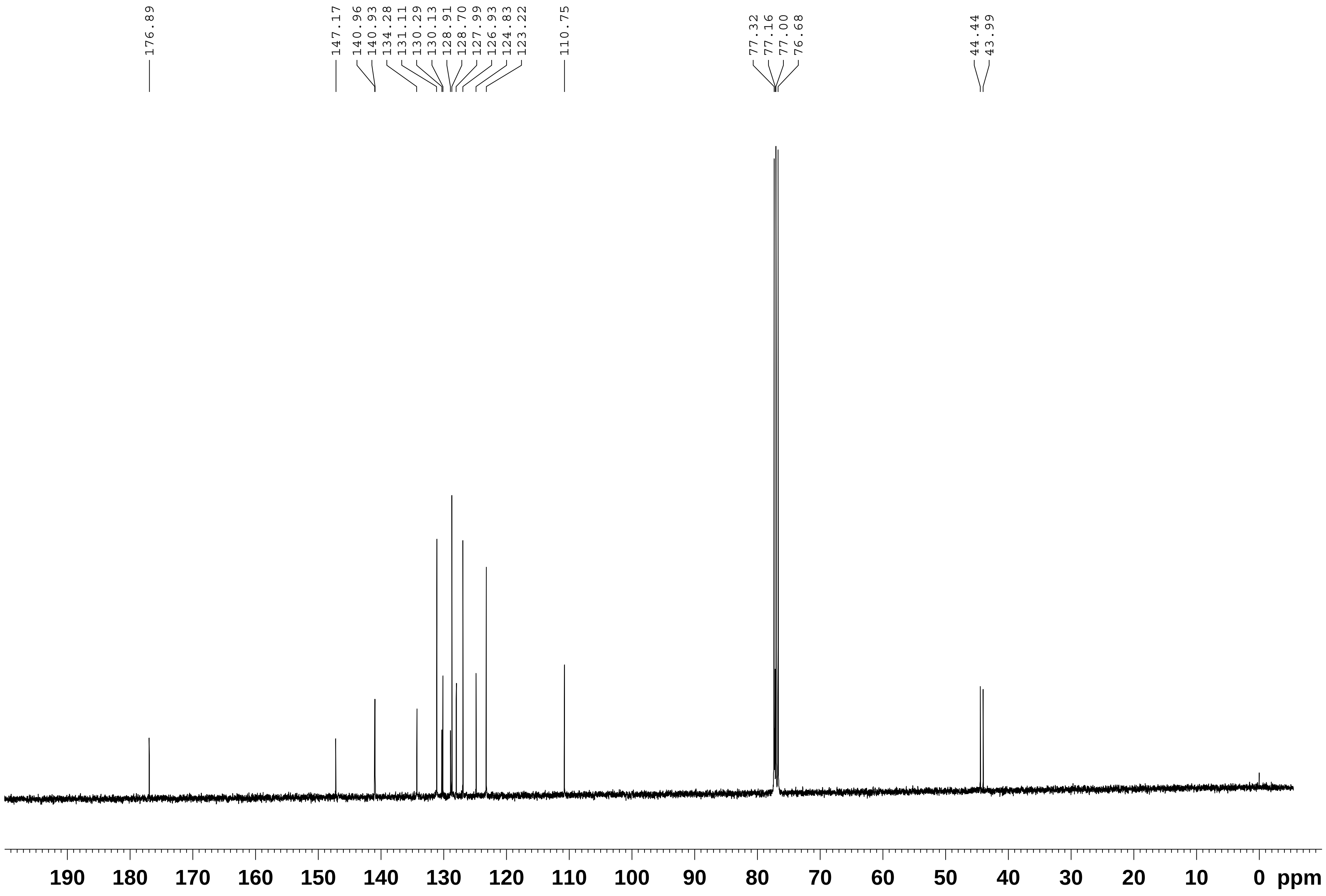

Supplementary Figure 78. ^{1}H NMR Spectrum of substrate 7l

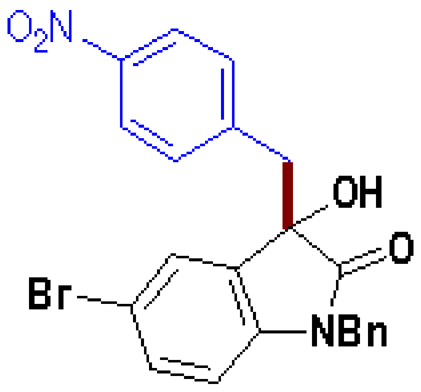

7l, 74%

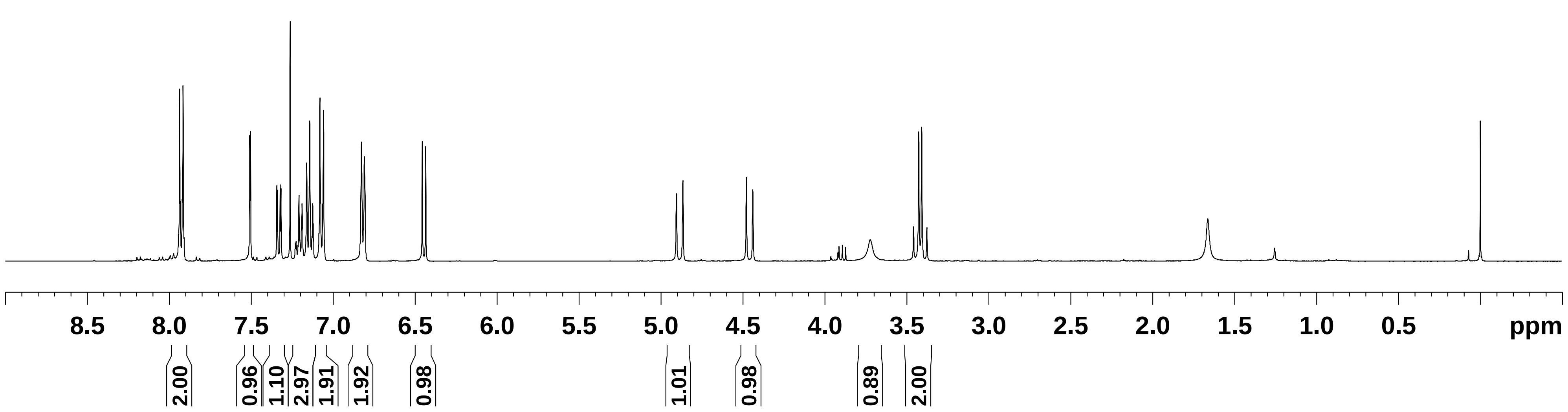

Supplementary Figure 79. ^{13}C NMR Spectrum of substrate 7l

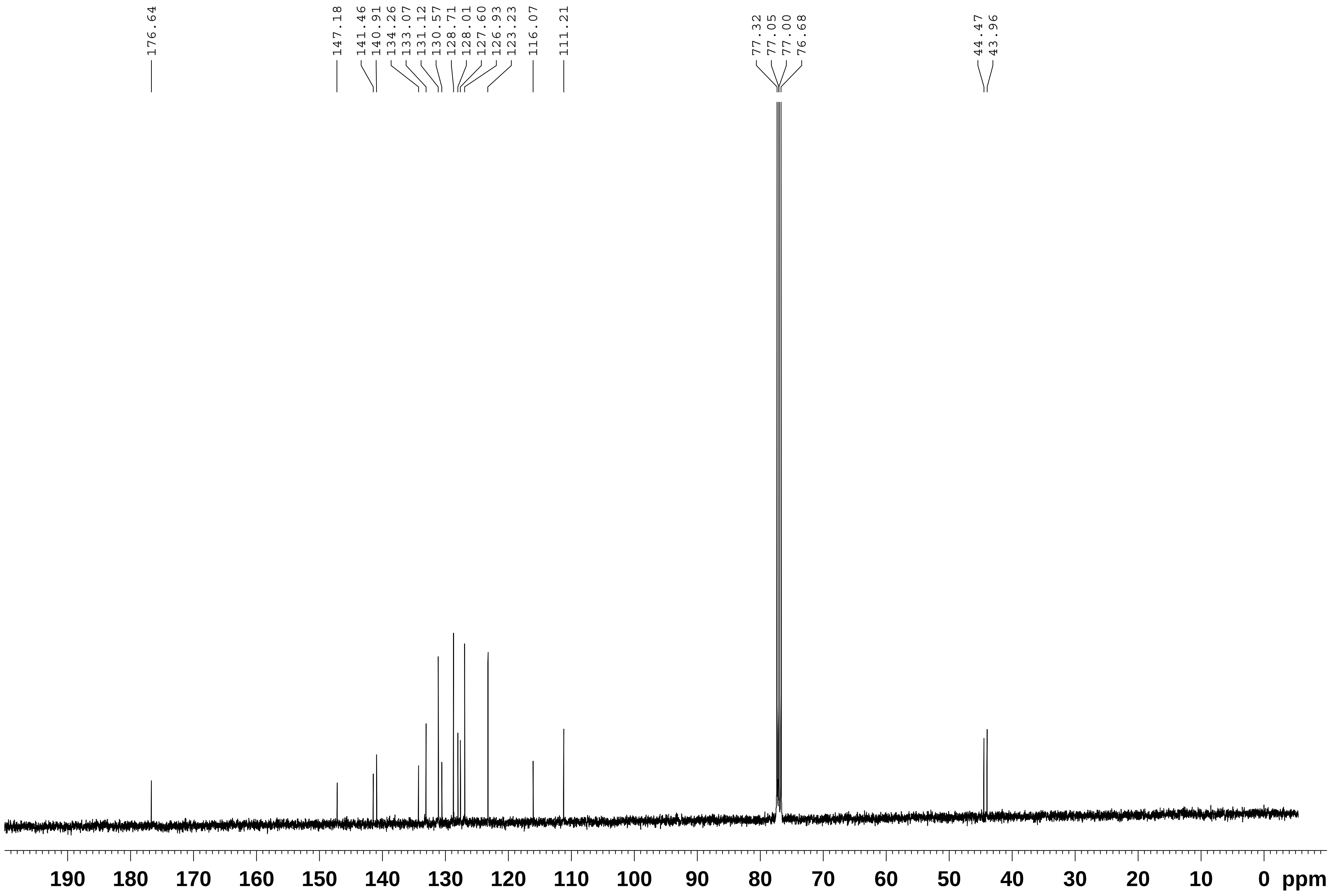

Supplementary Figure 80. ^{1}H NMR Spectrum of substrate 7m

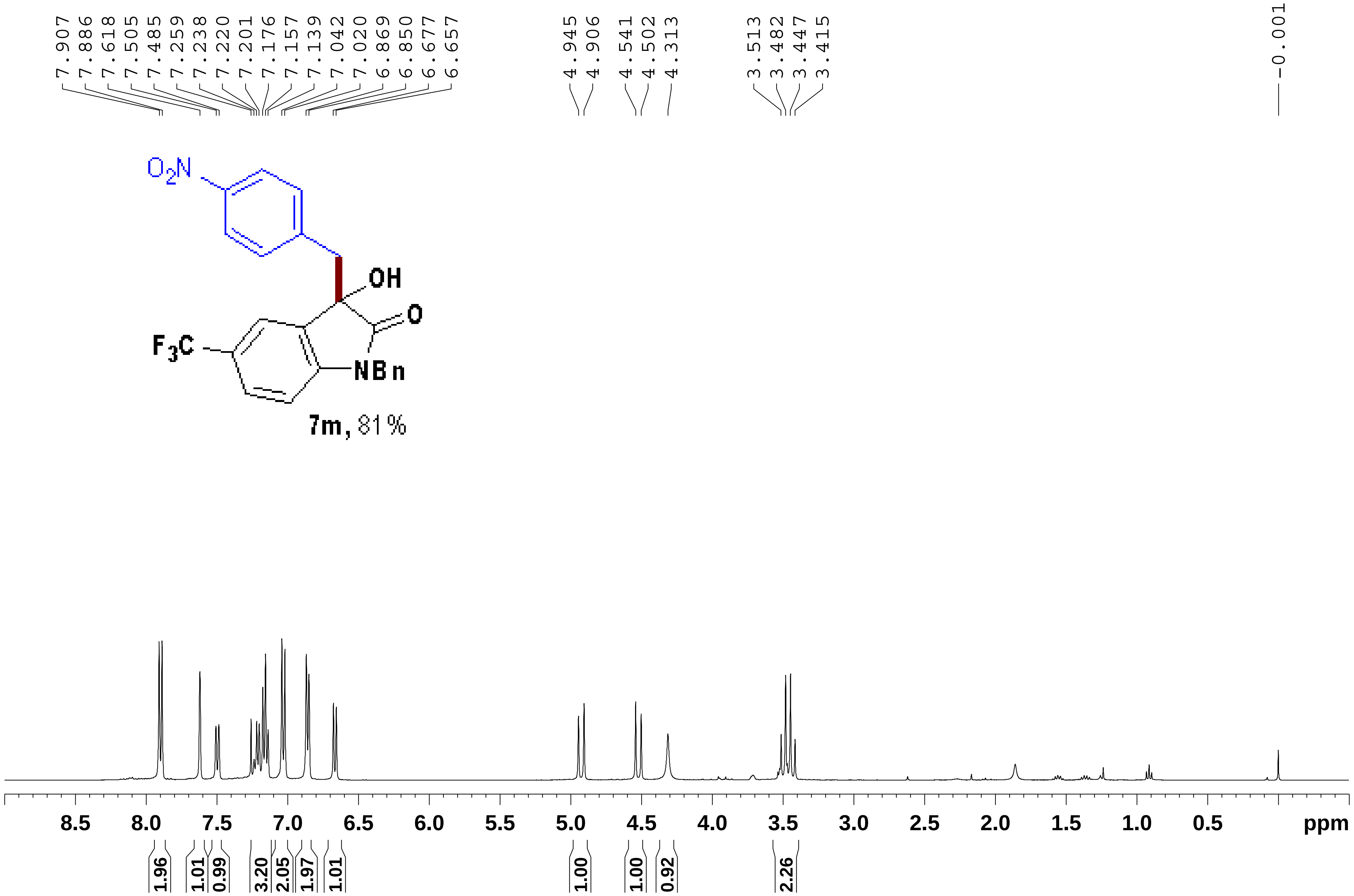

Supplementary Figure 81. ^{13}**C NMR Spectrum of substrate 7m**

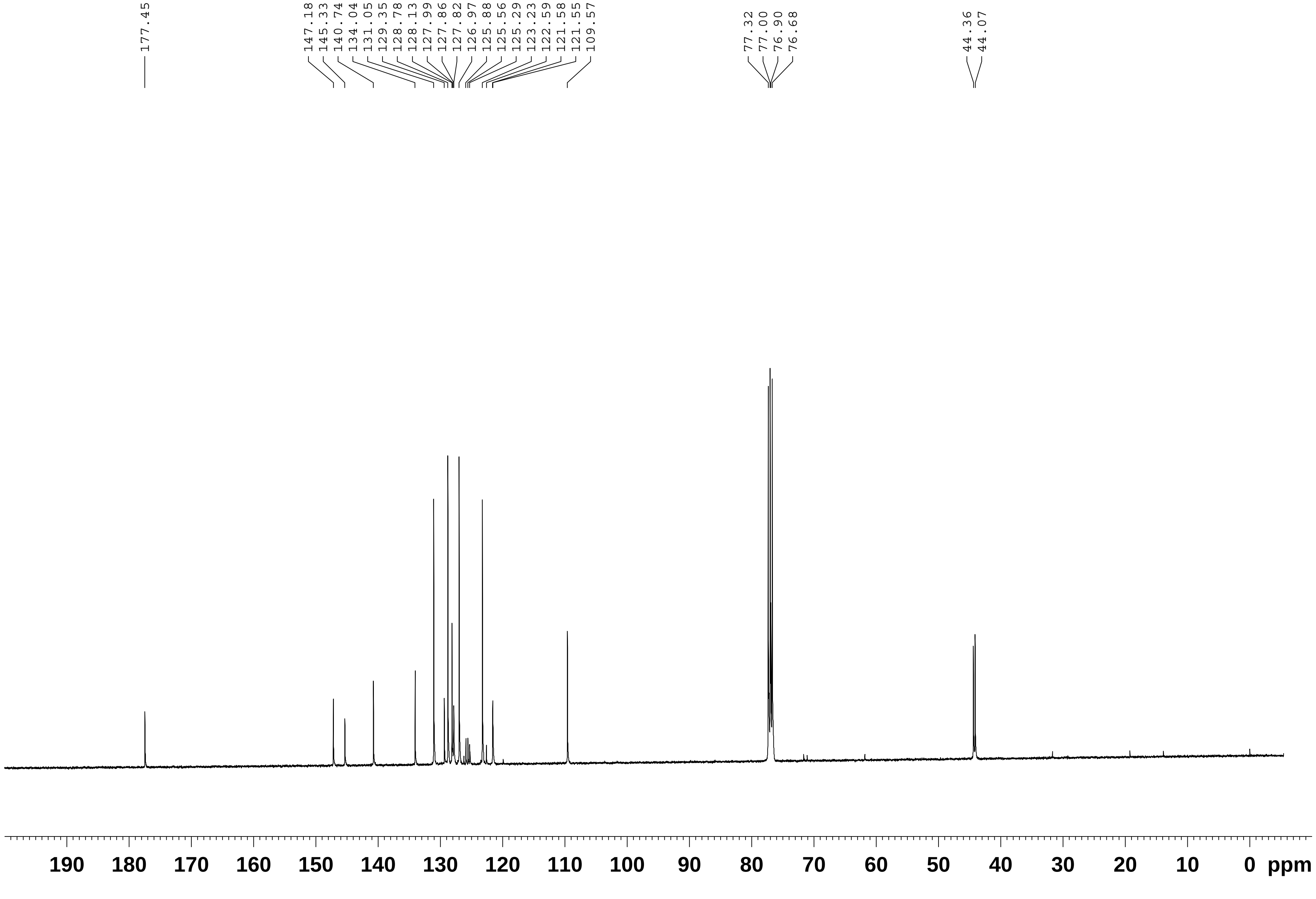

Supplementary Figure 82. ^{19}F NMR Spectrum of substrate 7m

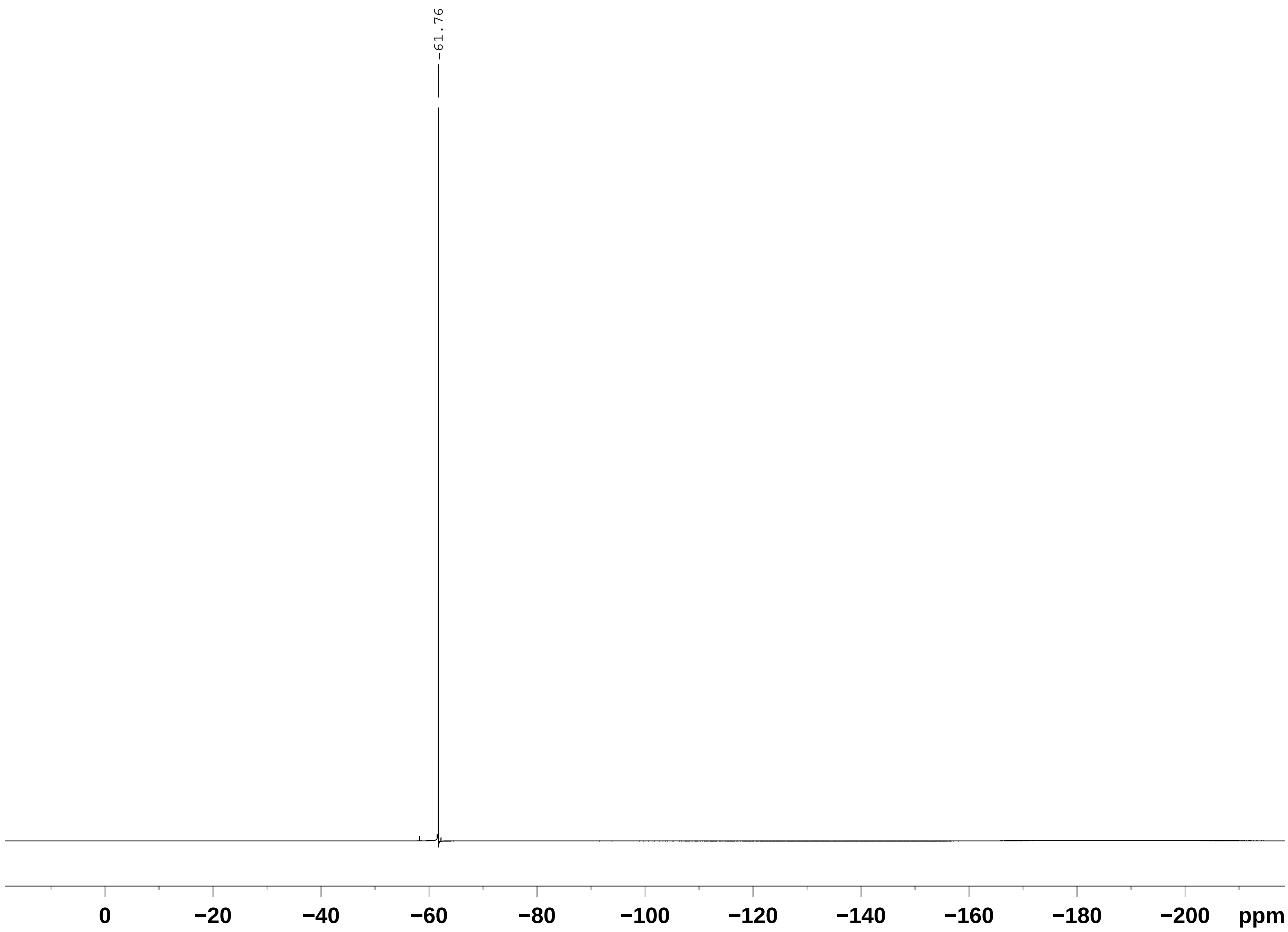

Supplementary Figure 83. ^{1}H NMR Spectrum of substrate 7n

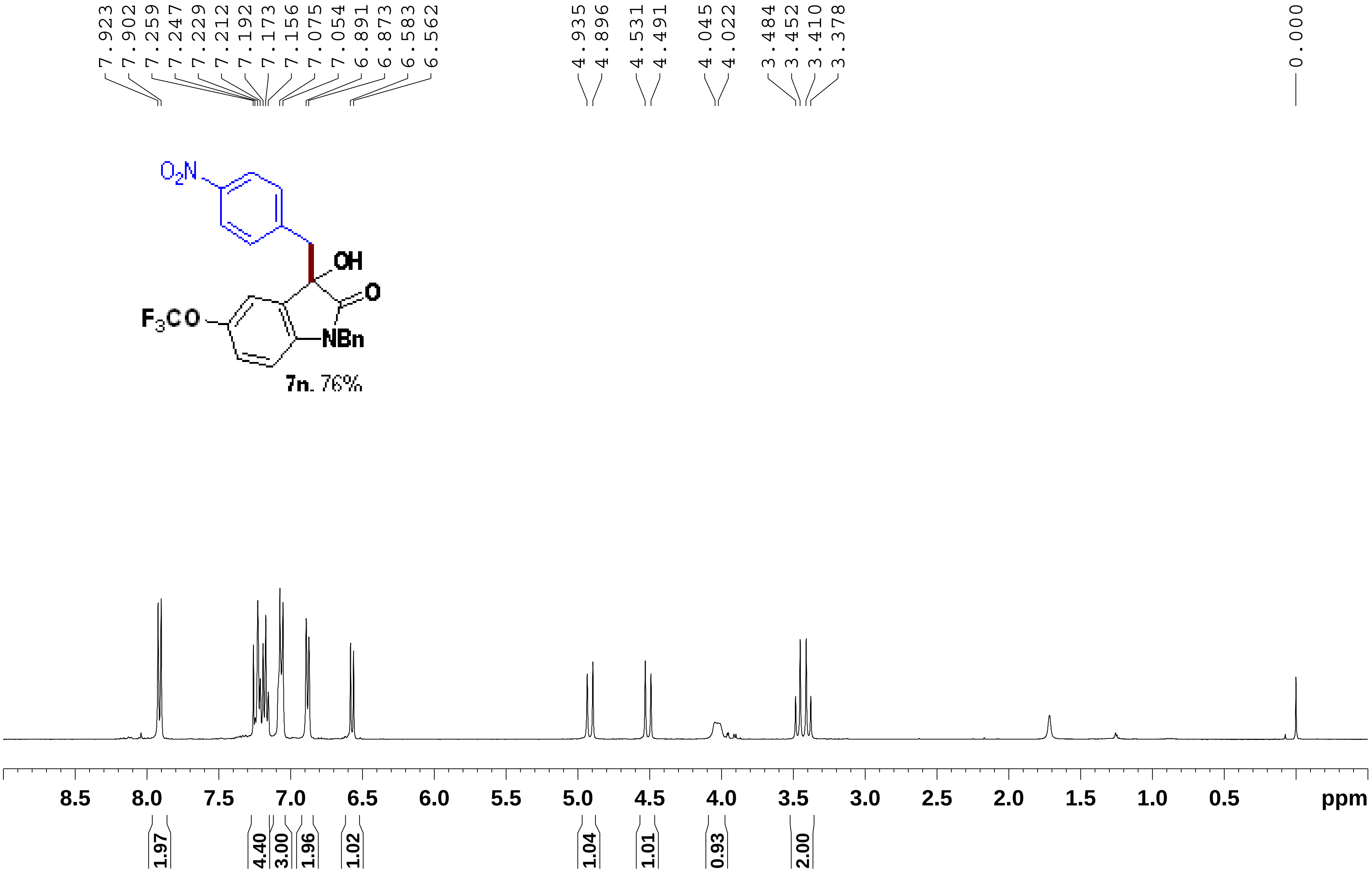

Supplementary Figure 84. ^{13}C NMR Spectrum of substrate 7n

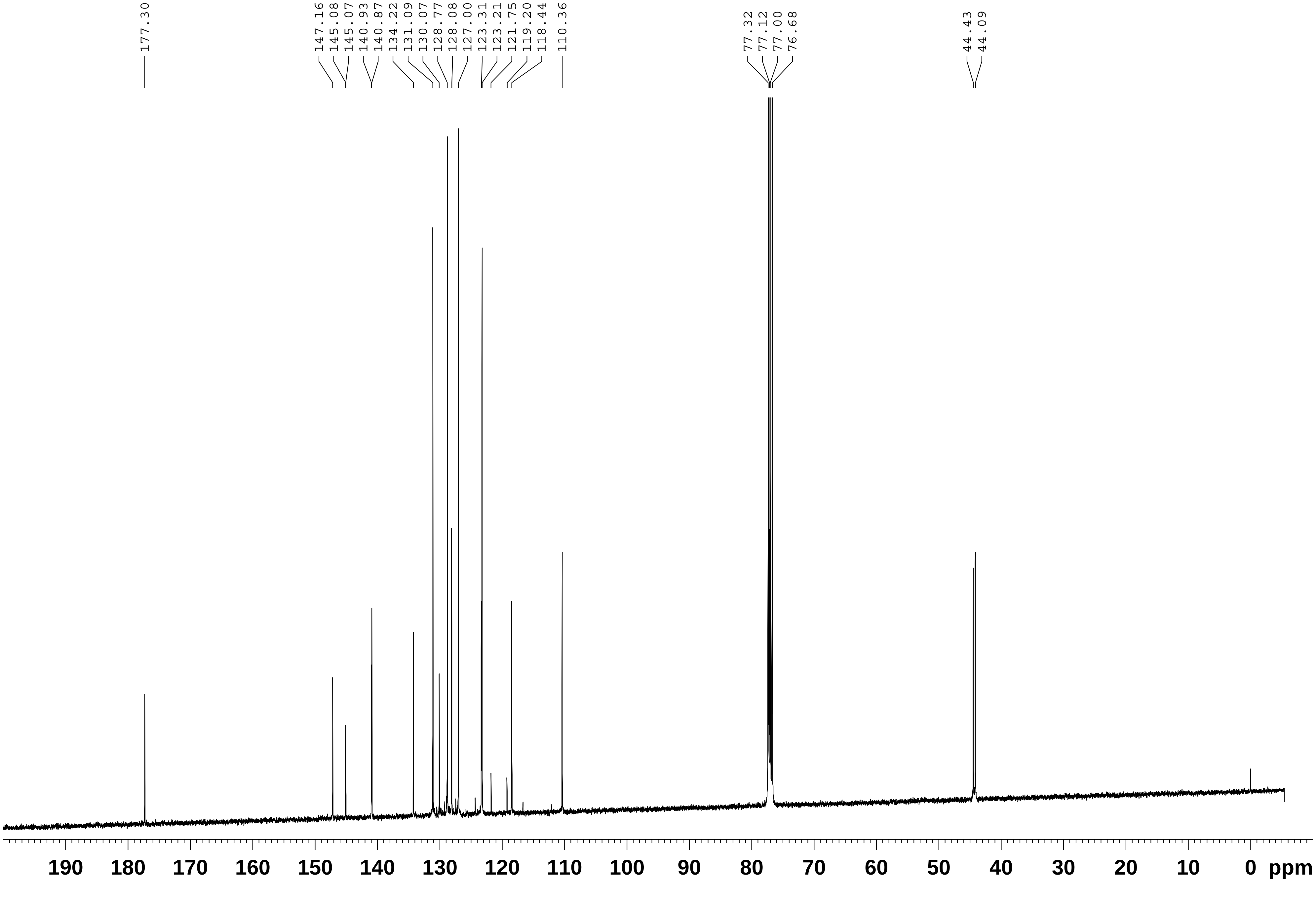

Supplementary Figure 85. ^{19}**F NMR Spectrum of substrate 7n**

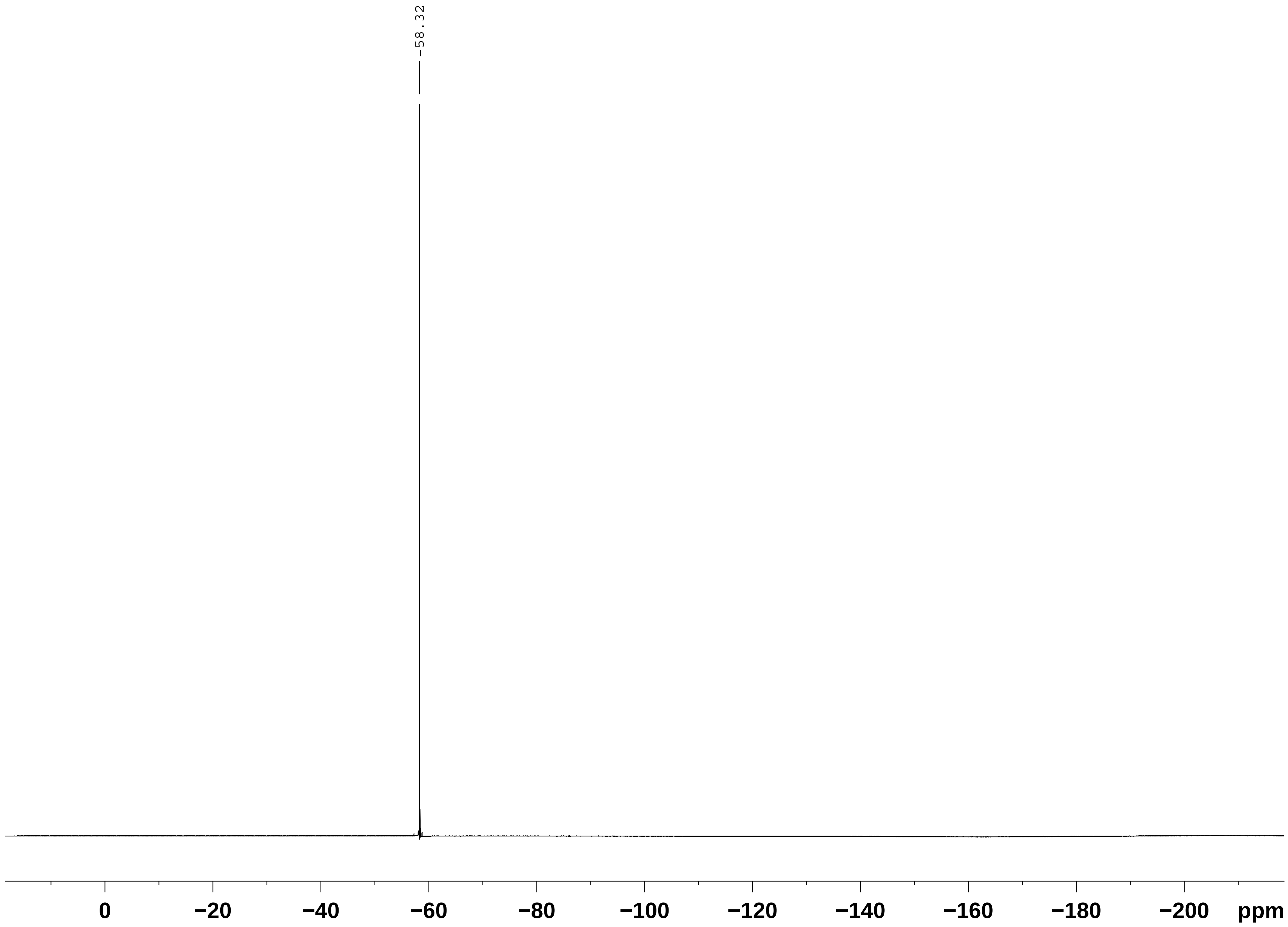

Supplementary Figure 86. ^{1}H NMR Spectrum of substrate 7o

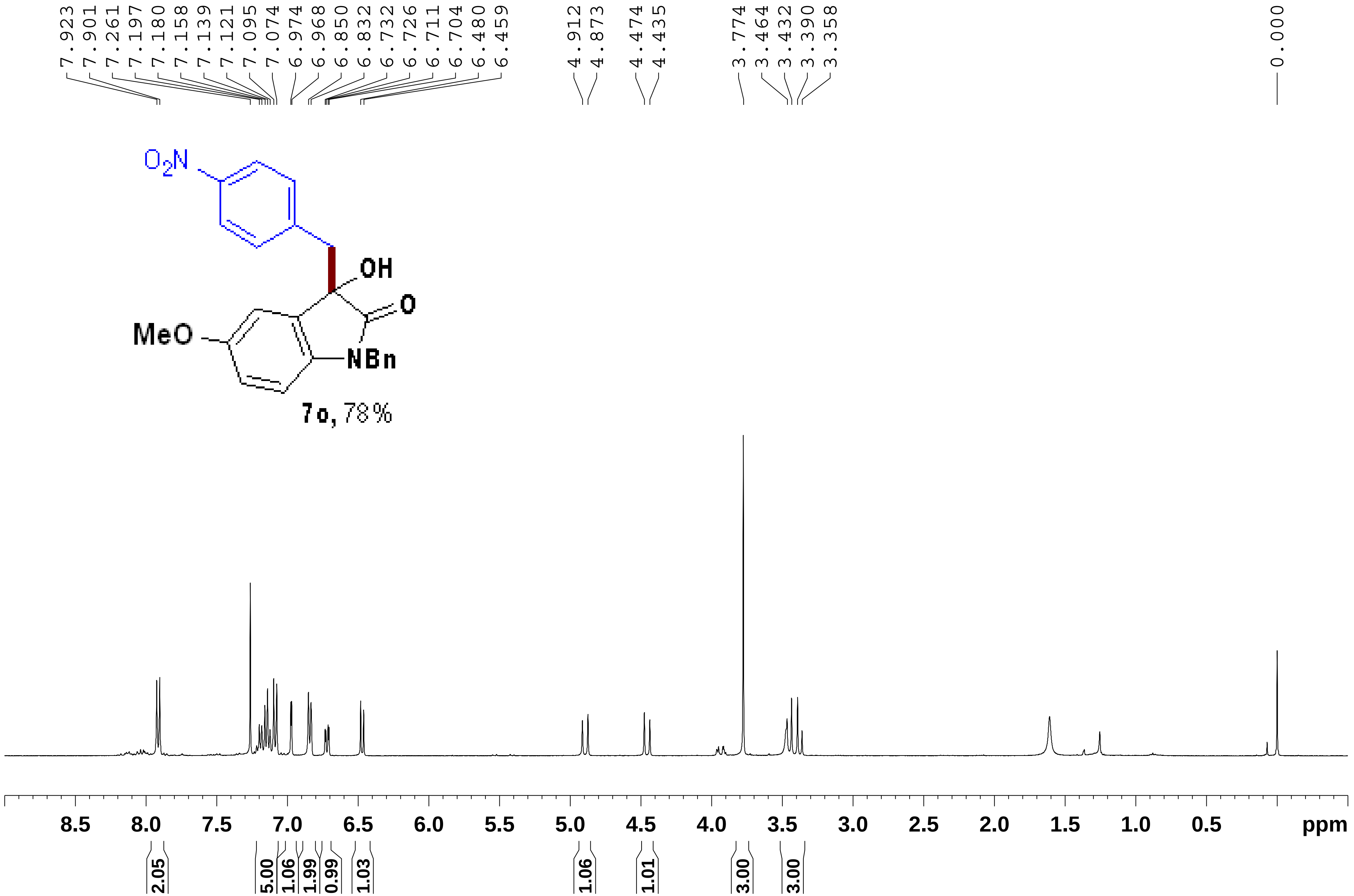

Supplementary Figure 87. ^{13}C NMR Spectrum of substrate 7o

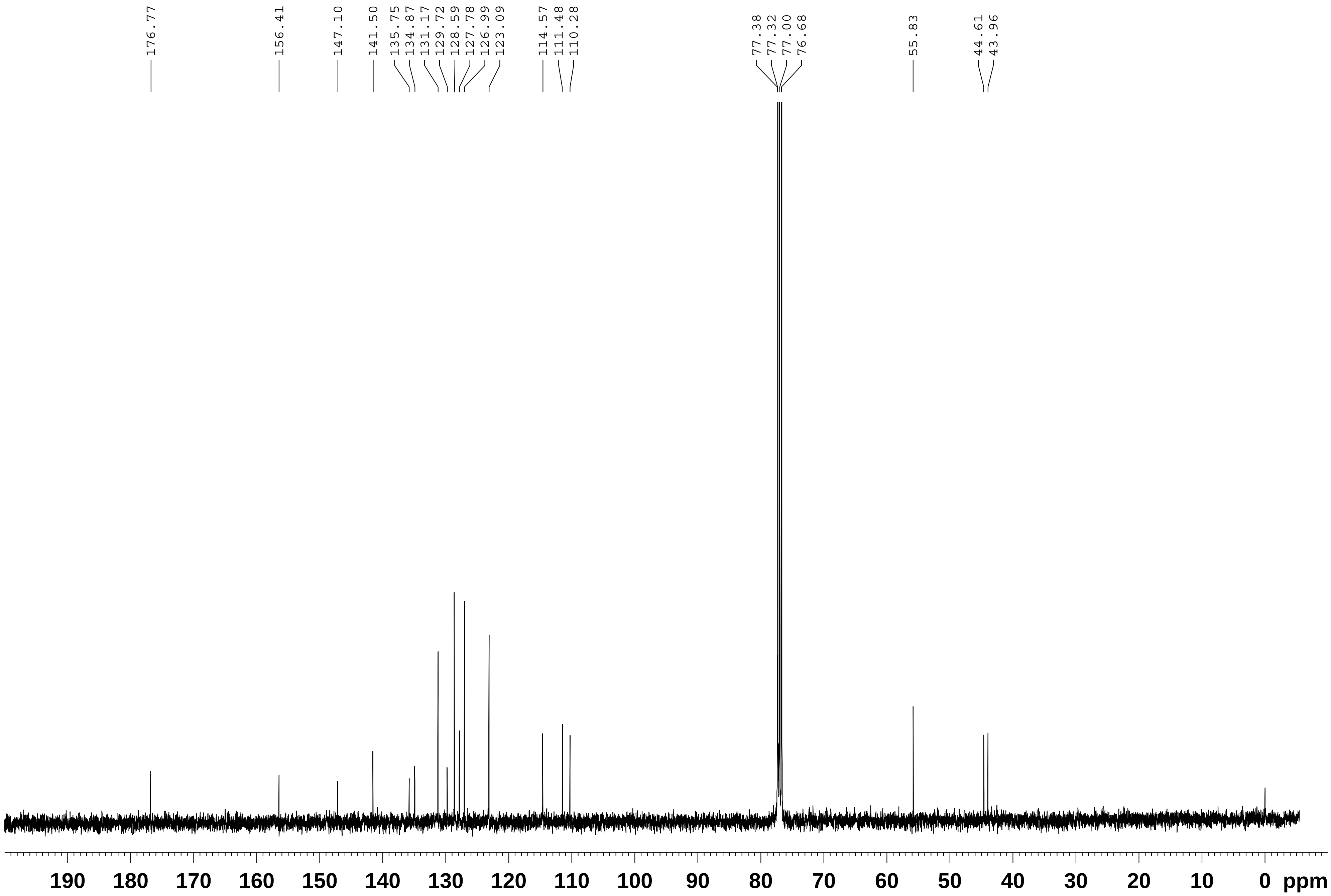

Supplementary Figure 88. ^{1}H NMR Spectrum of substrate 7p

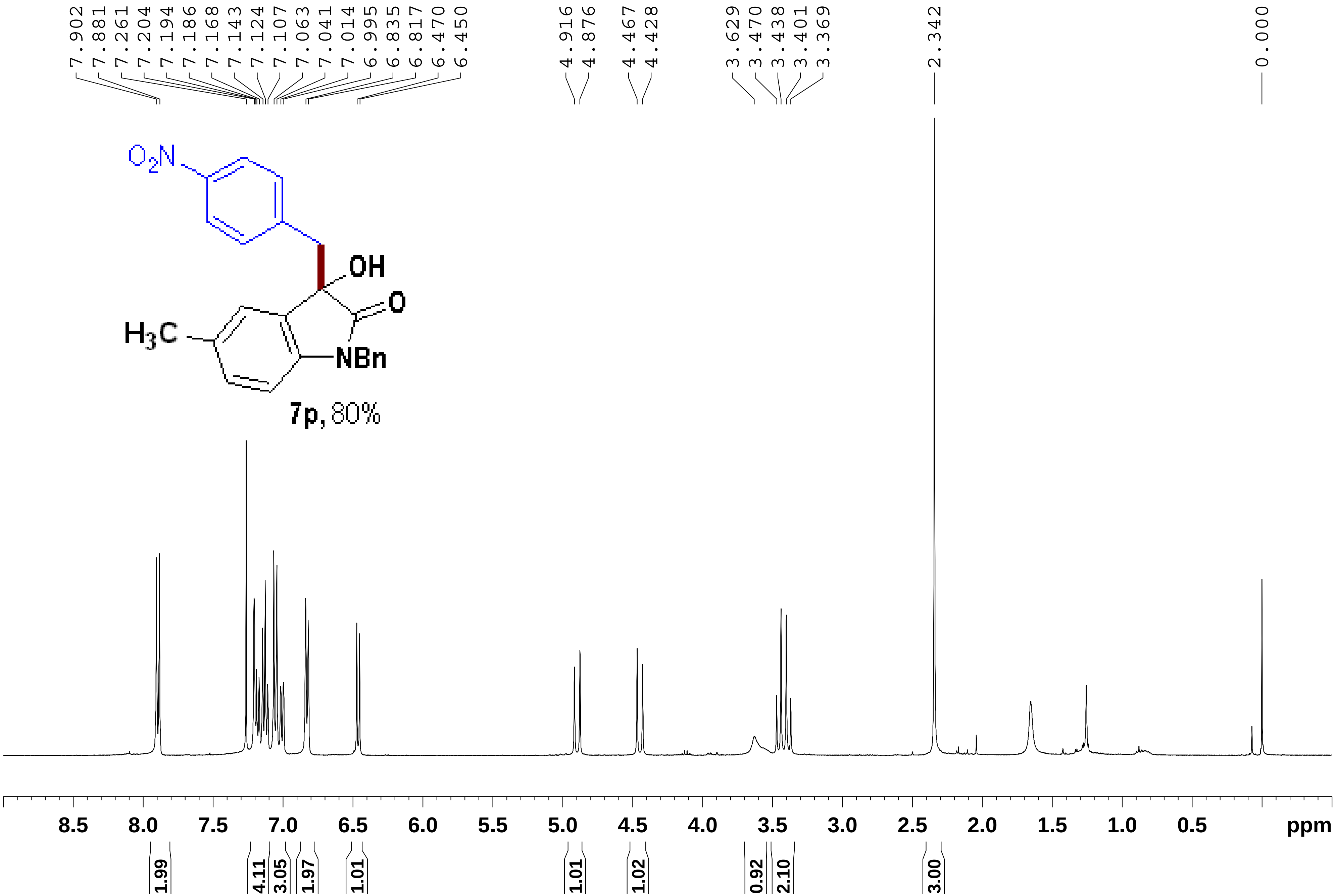

Supplementary Figure 89. ^{13}C NMR Spectrum of substrate 7p

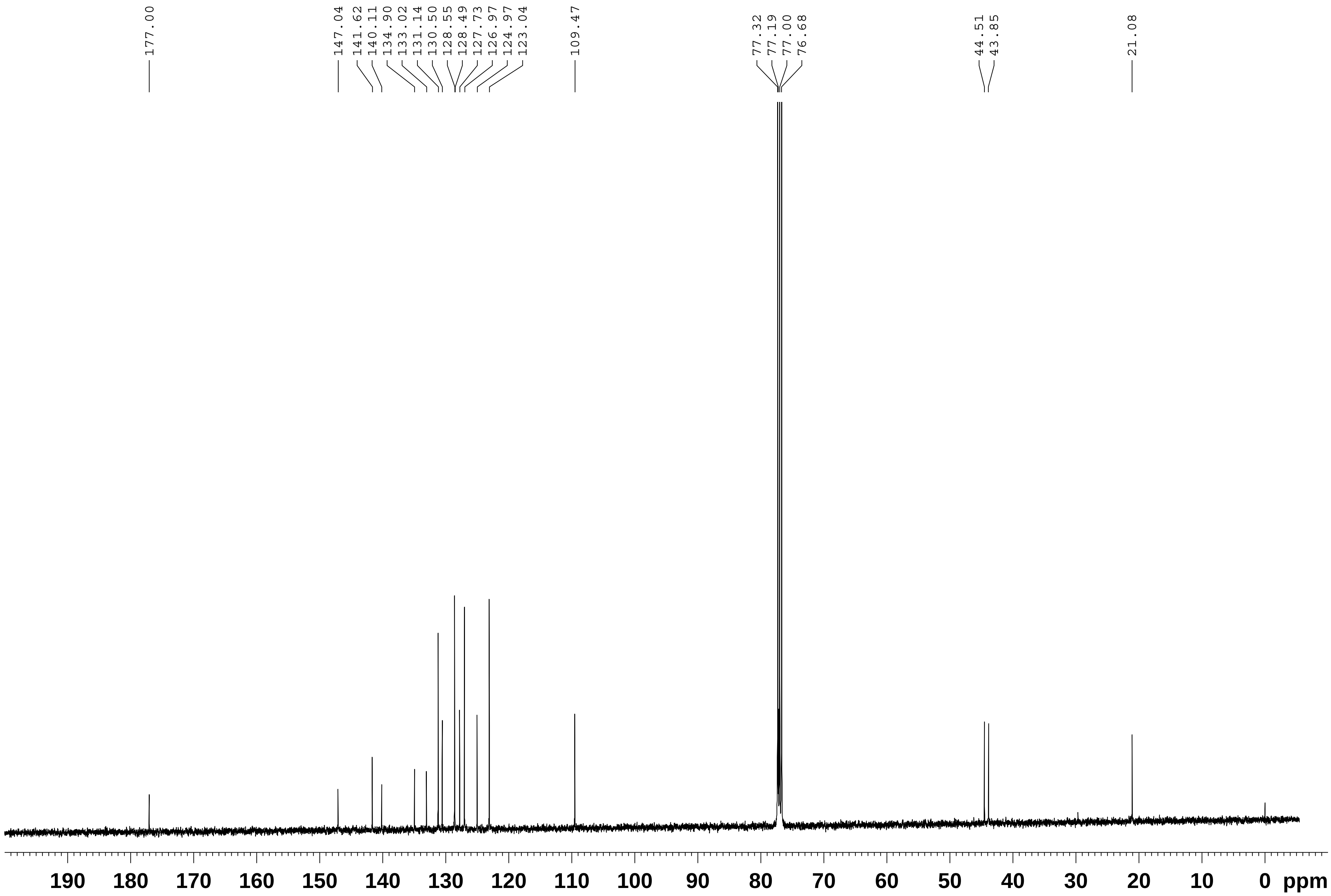

Supplementary Figure 90. ^{1}H NMR Spectrum of substrate 7q

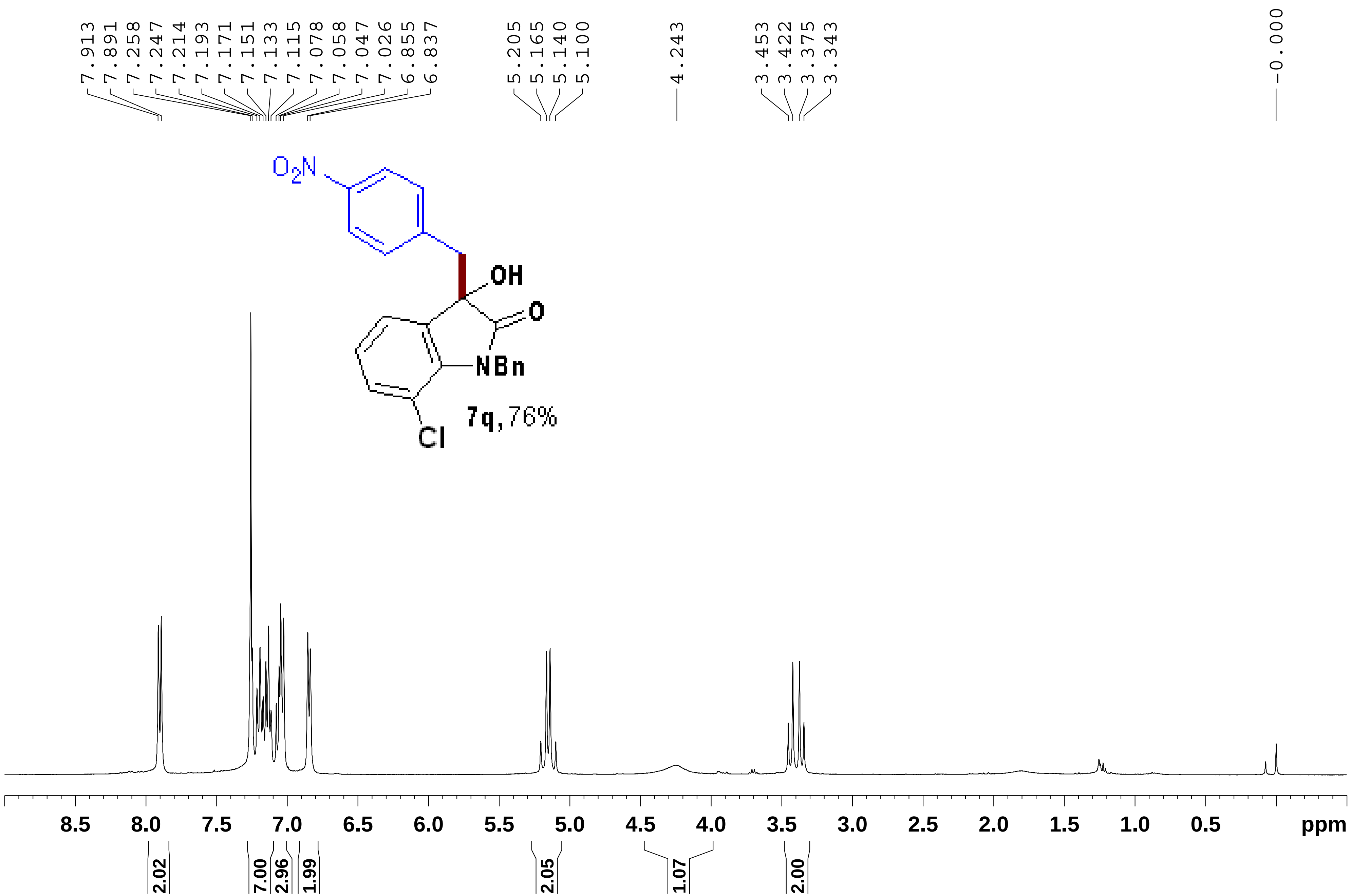

Supplementary Figure 91. ^{13}C NMR Spectrum of substrate 7q

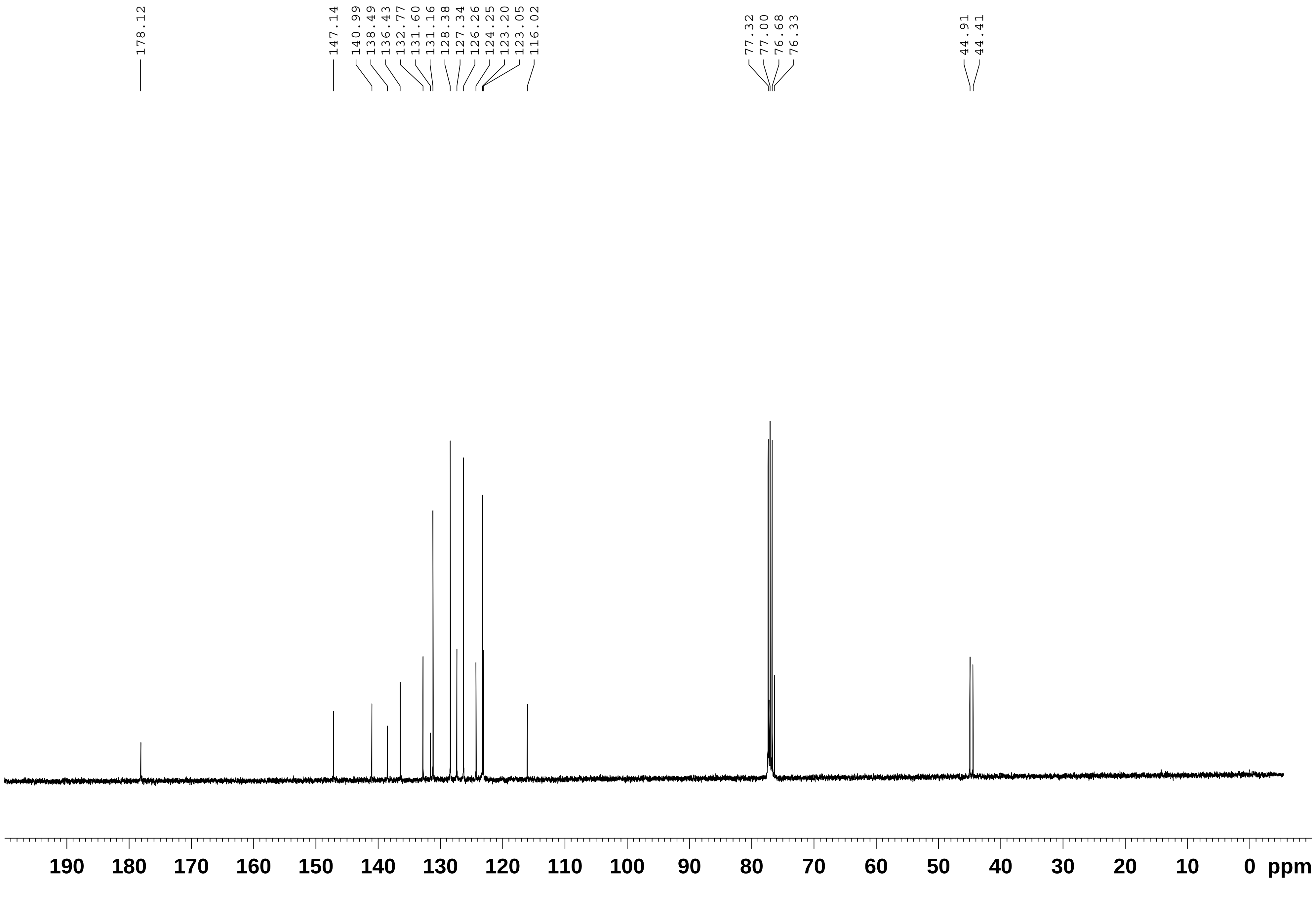

Supplementary Figure 92. ^{1}H NMR Spectrum of substrate 7r

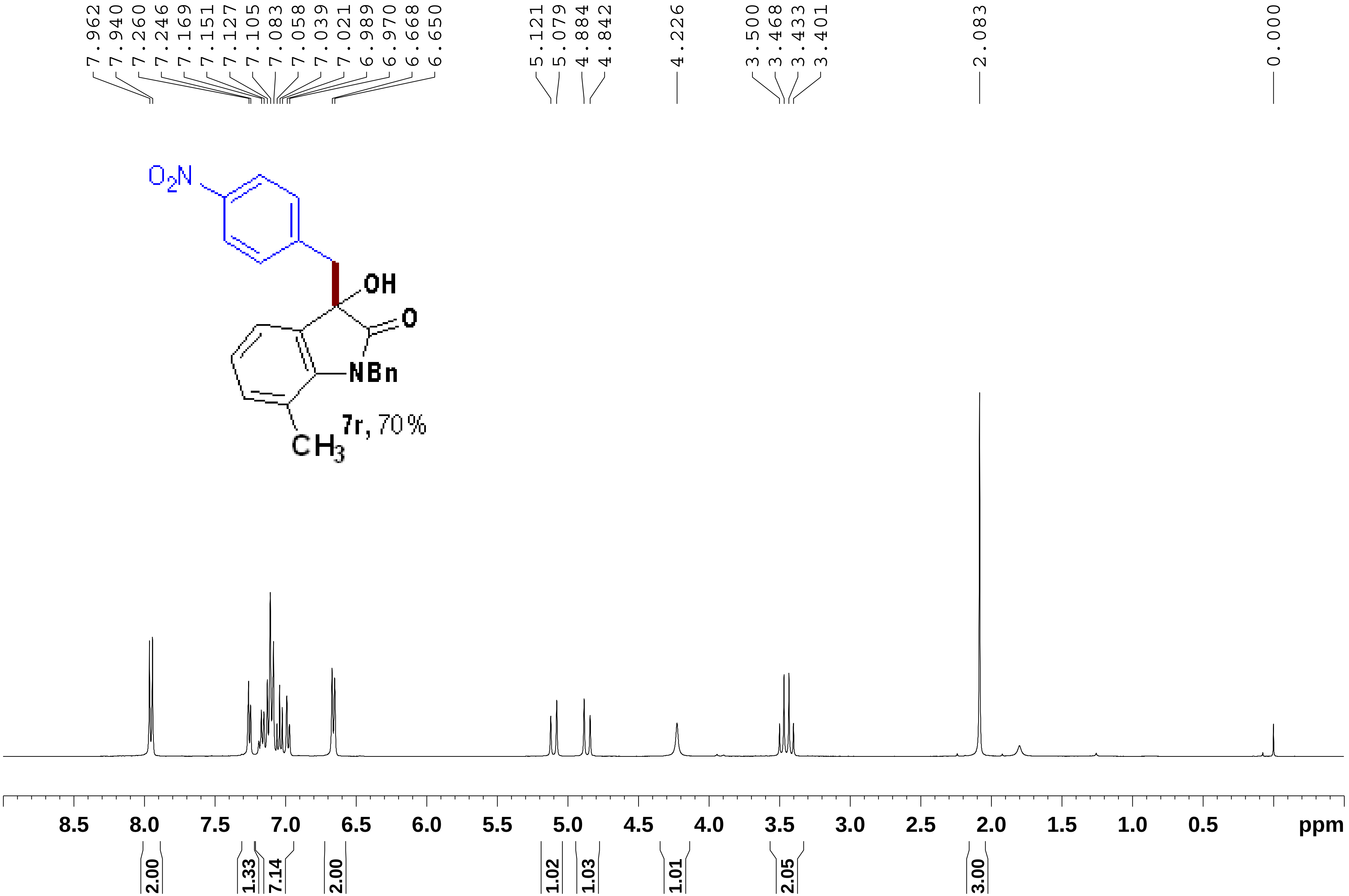

Supplementary Figure 93. ^{13}C NMR Spectrum of substrate 7r

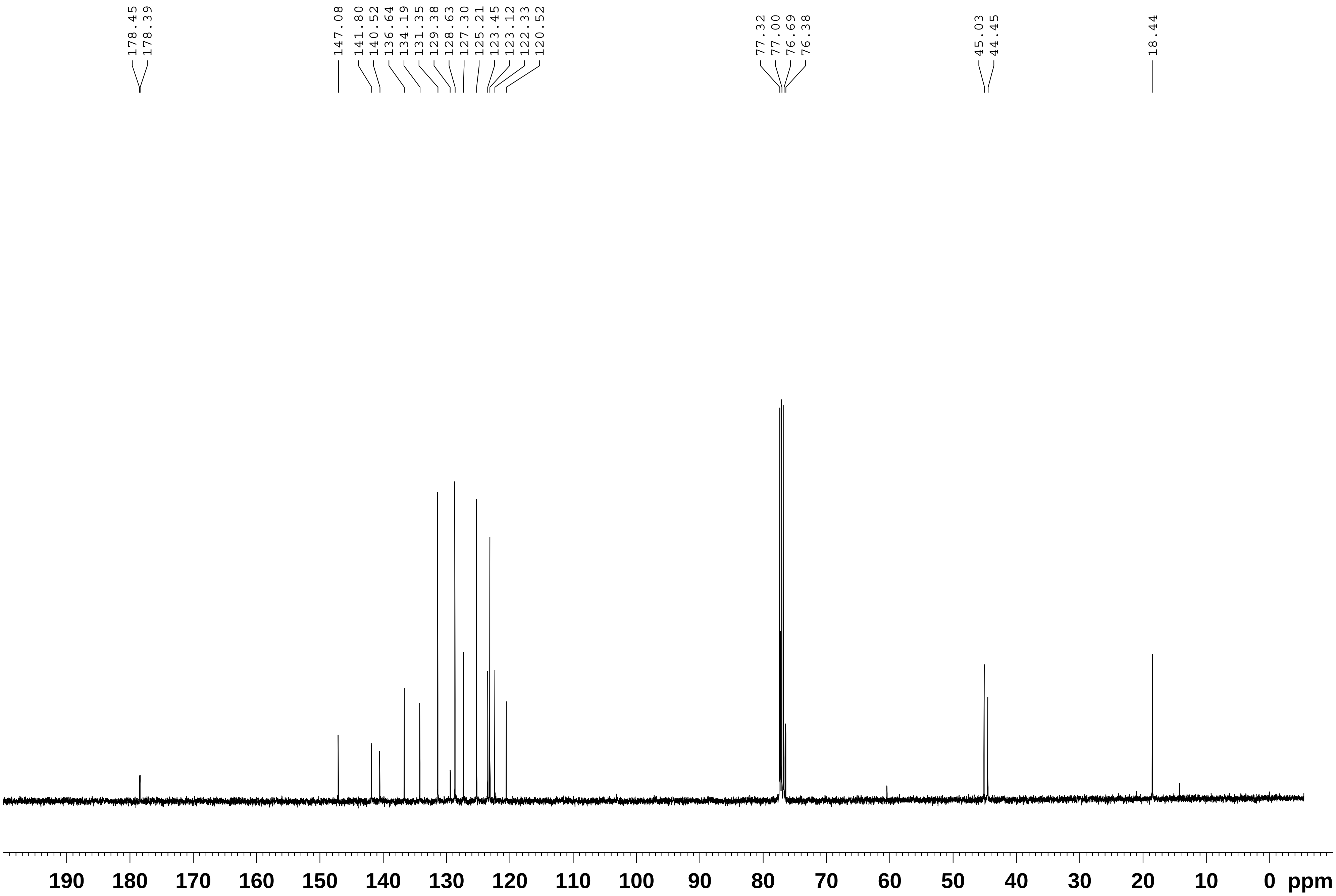

Supplementary Figure 94. ^{1}H NMR Spectrum of substrate 7s

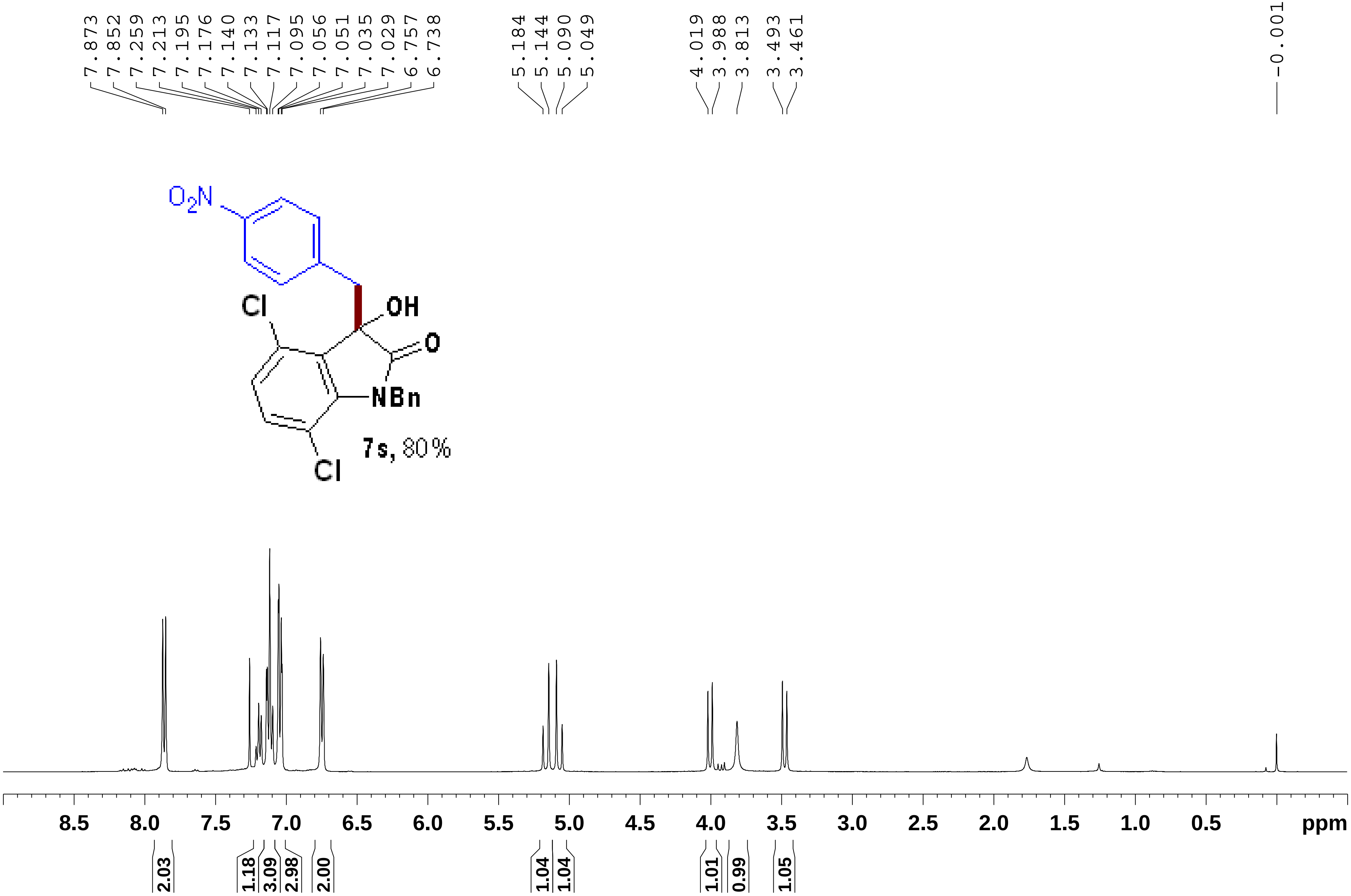

Supplementary Figure 95. ^{13}C NMR Spectrum of substrate 7s

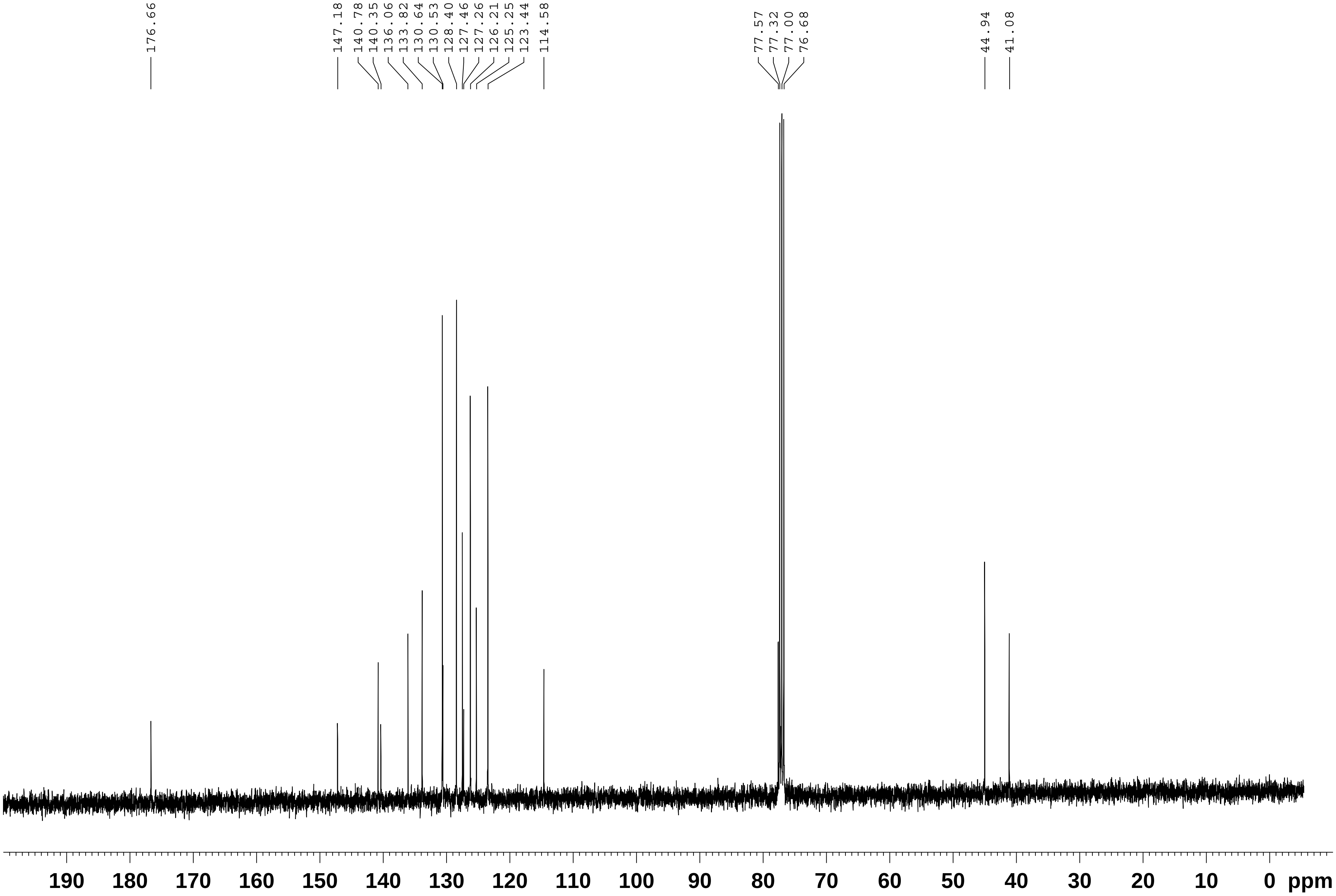

Supplementary Figure 96. ^{1}H NMR Spectrum of substrate 7t

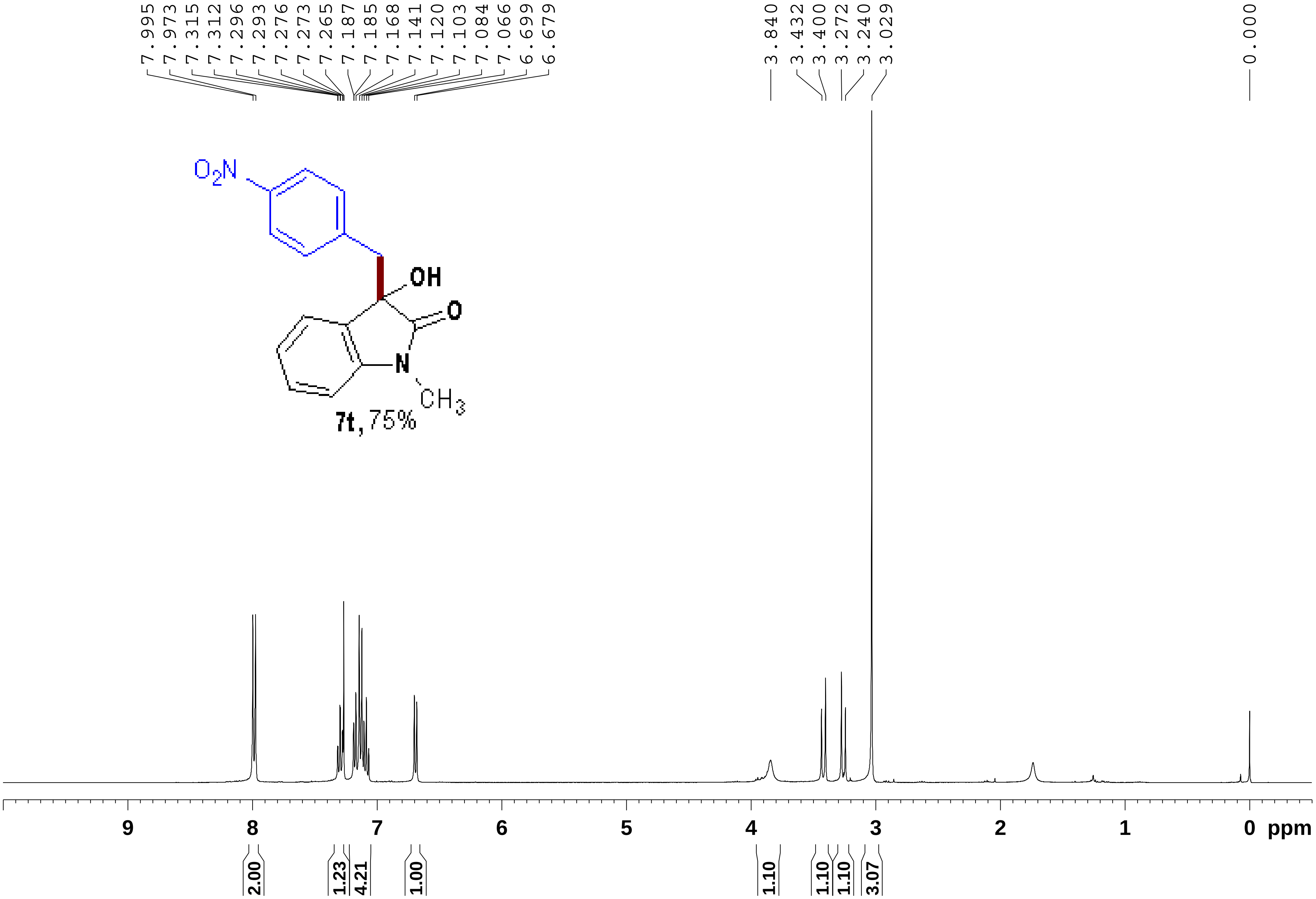

Supplementary Figure 97. ^{13}C NMR Spectrum of substrate 7t

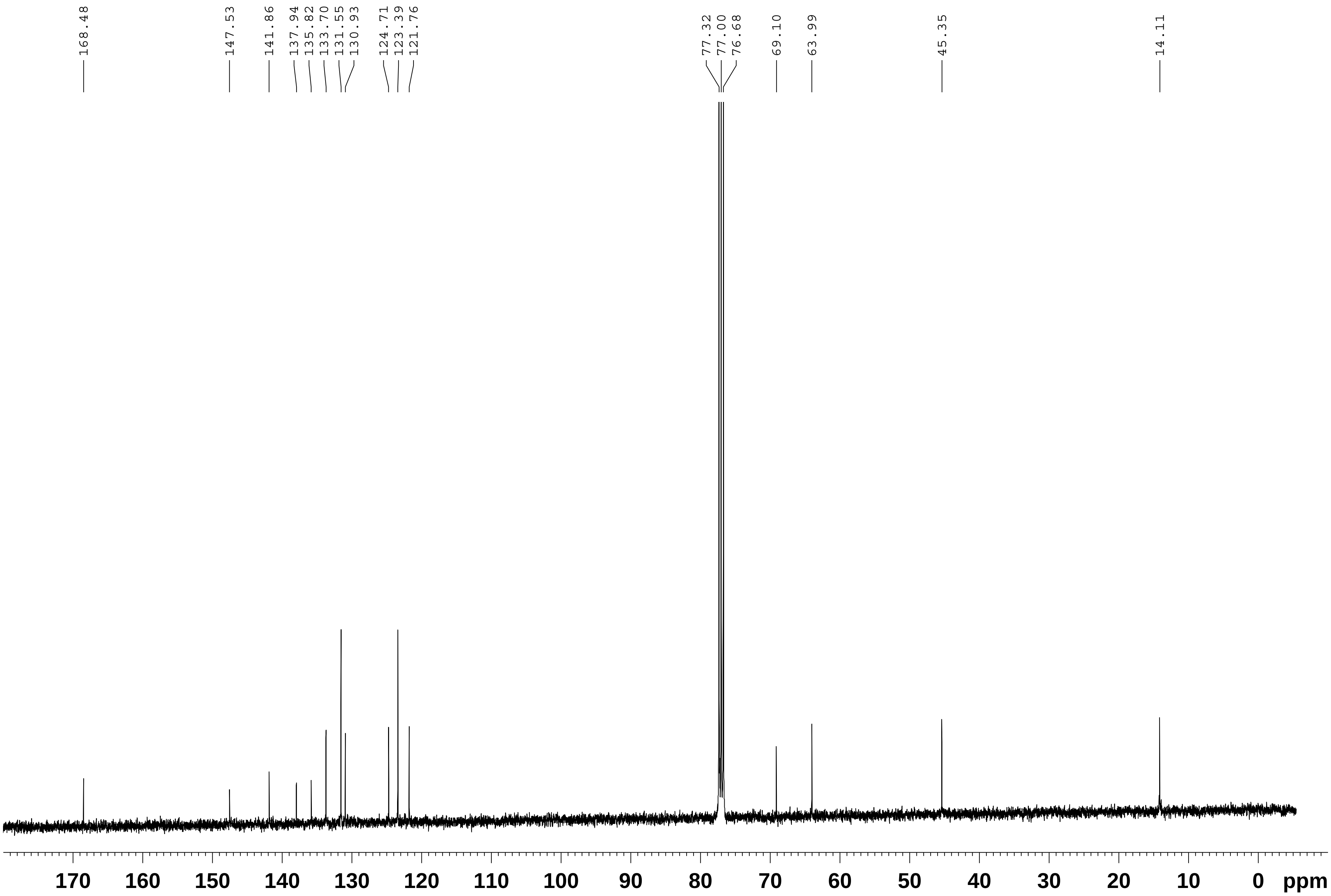

Supplementary Figure 98. ^{1}H NMR Spectrum of substrate 9a

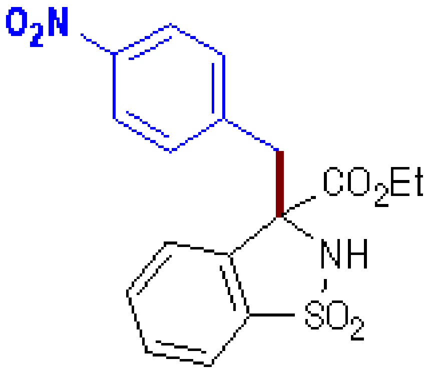

9a, 67%

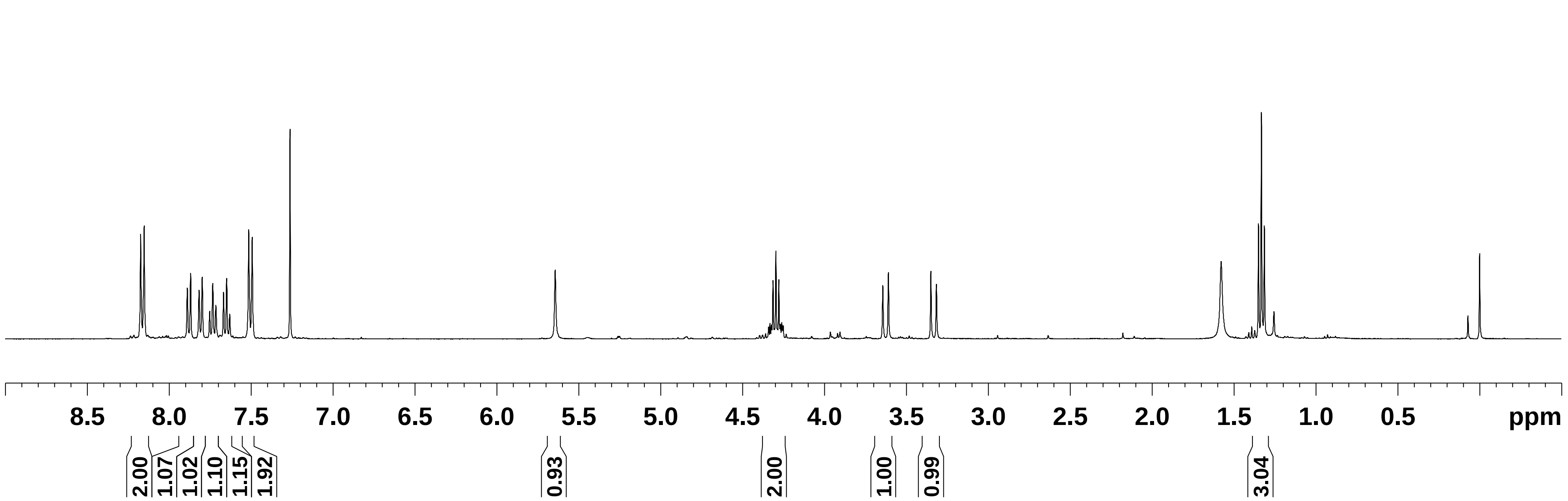

Supplementary Figure 99. ^{13}C NMR Spectrum of substrate 9a

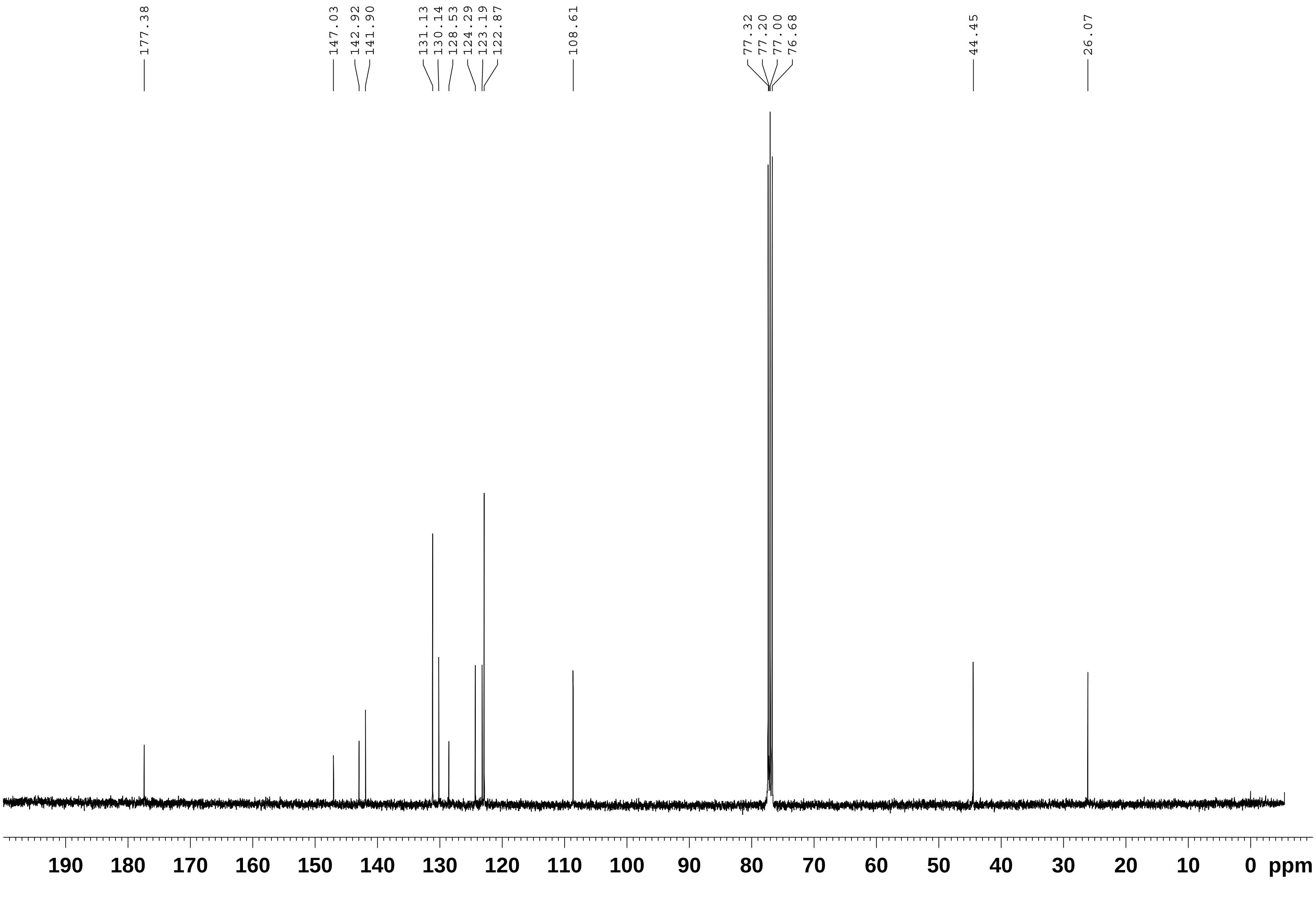

Supplementary Figure 100. ^{1}H NMR Spectrum of substrate 9b

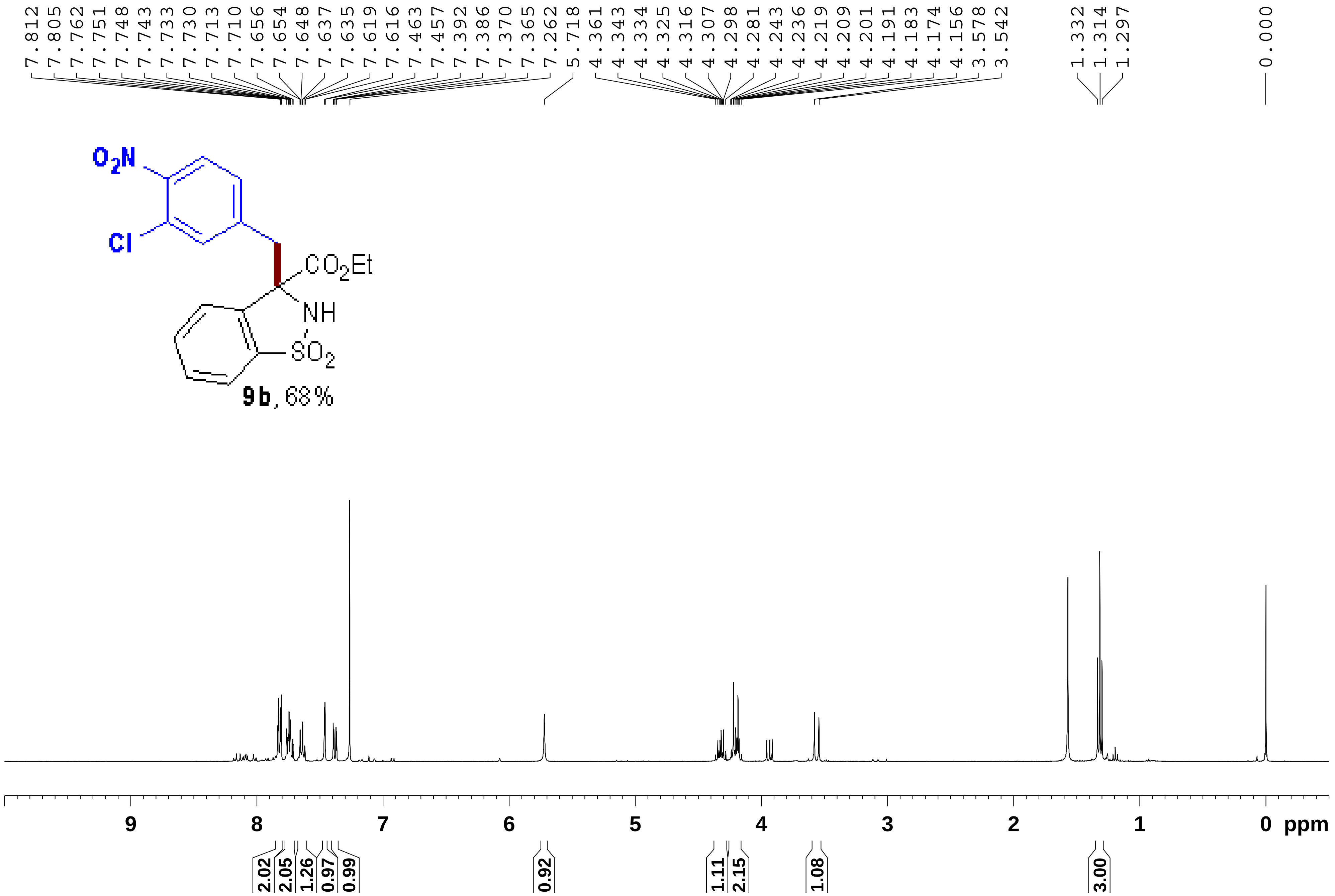

Supplementary Figure 101. ^{13}C NMR Spectrum of substrate 9b

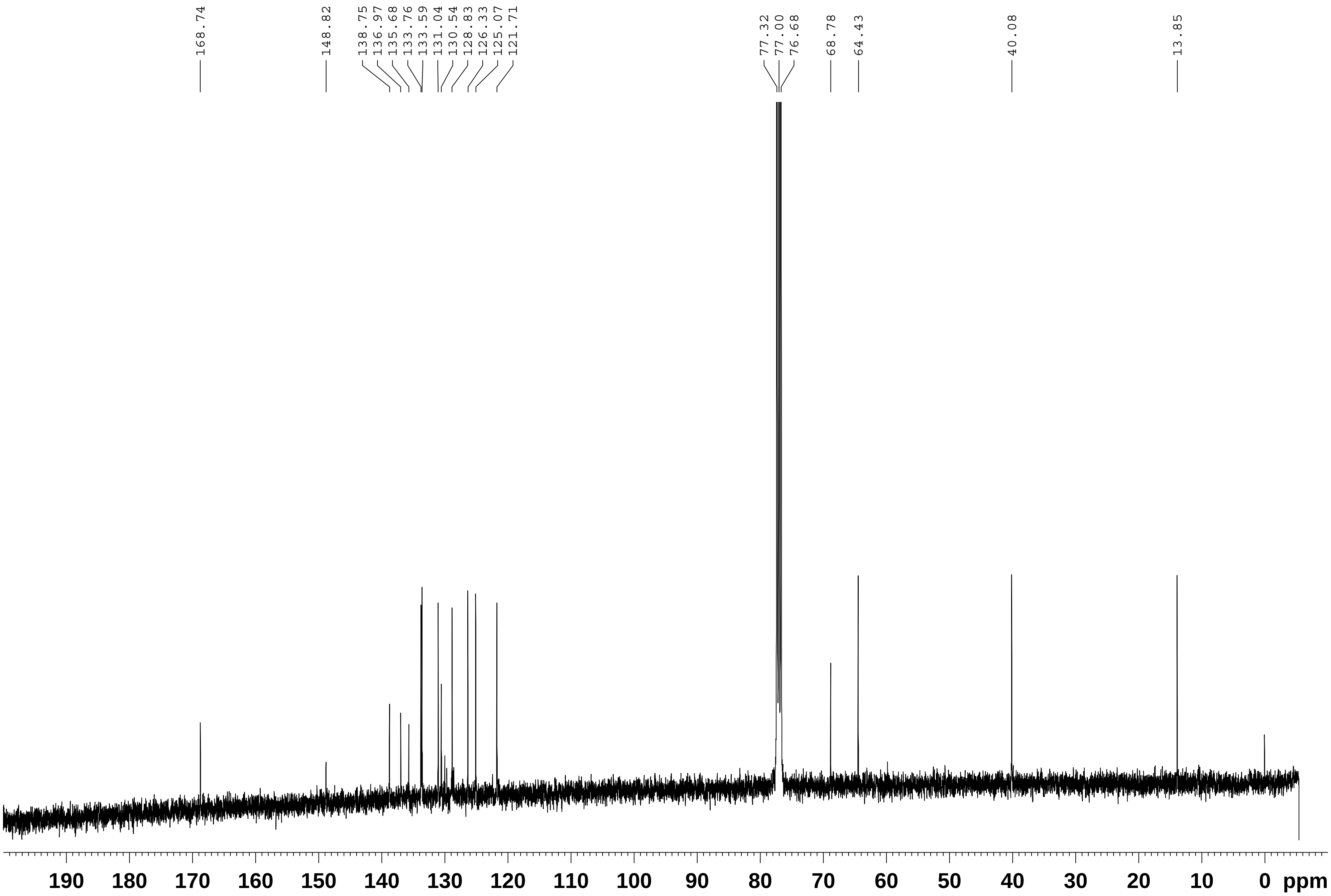

Supplementary Figure 102. ^{1}**H NMR Spectrum of substrate 9c**

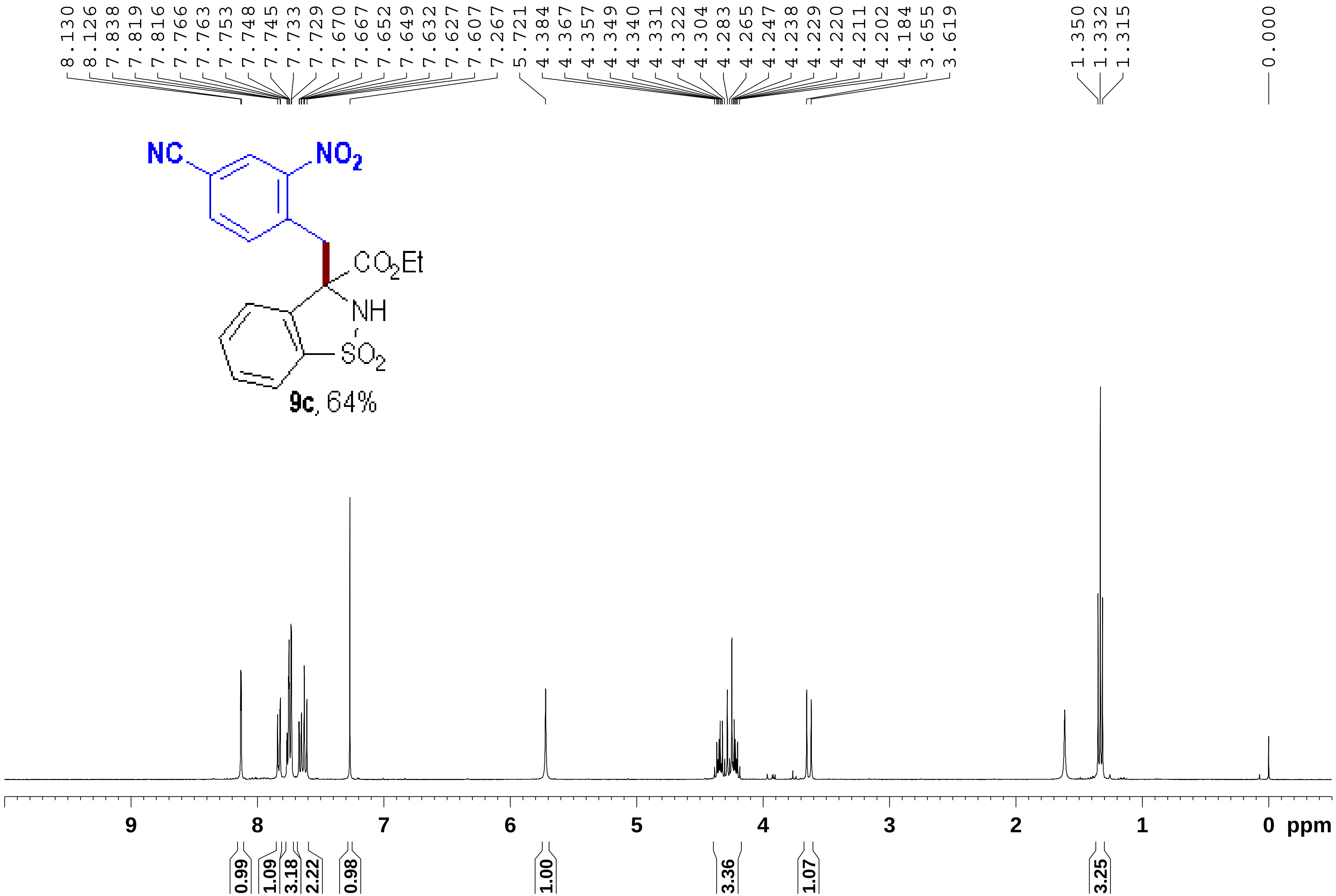

Supplementary Figure 103. ^{13}C NMR Spectrum of substrate 9c

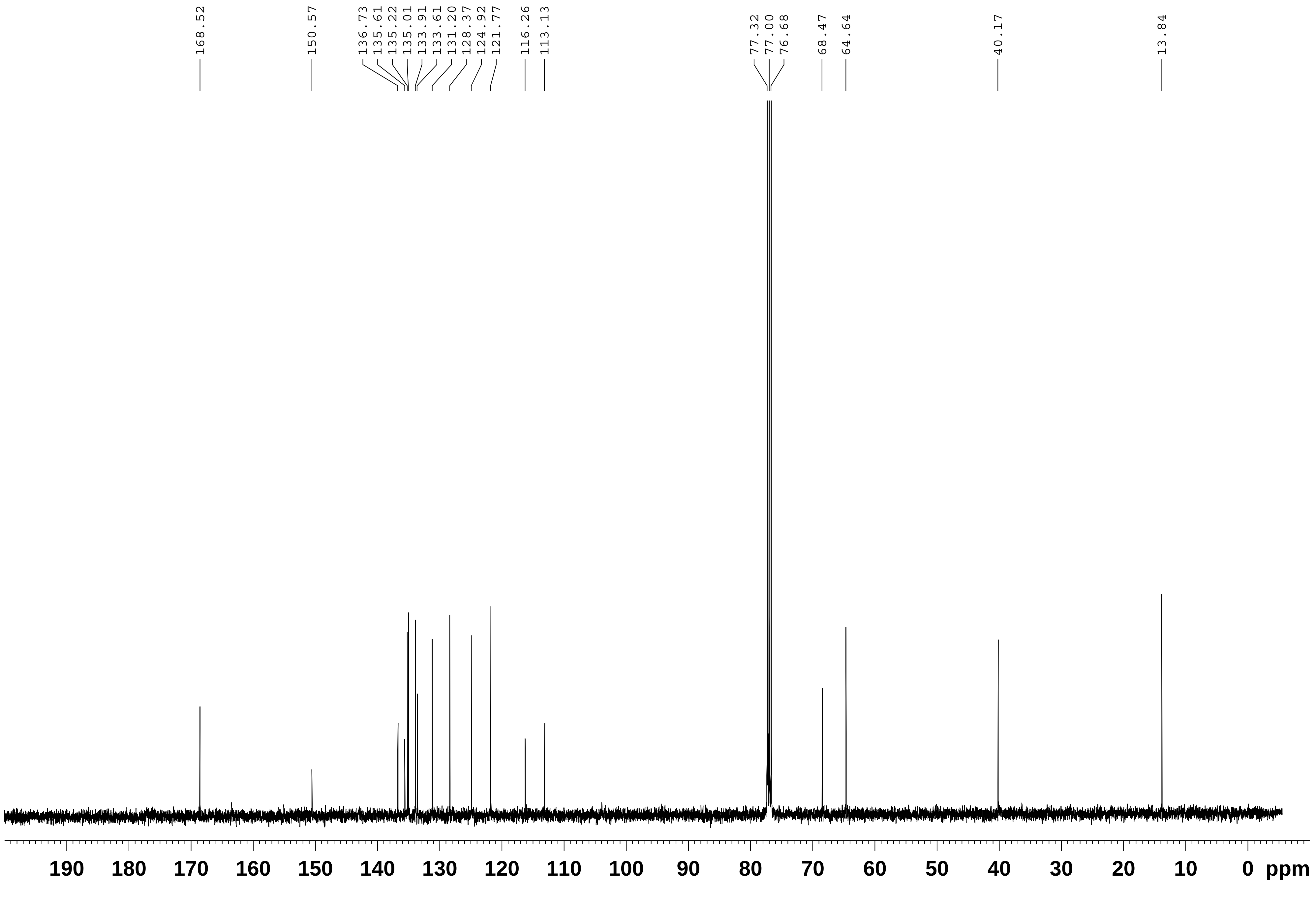

Supplementary Figure 104. ^{1}H NMR Spectrum of substrate 9d

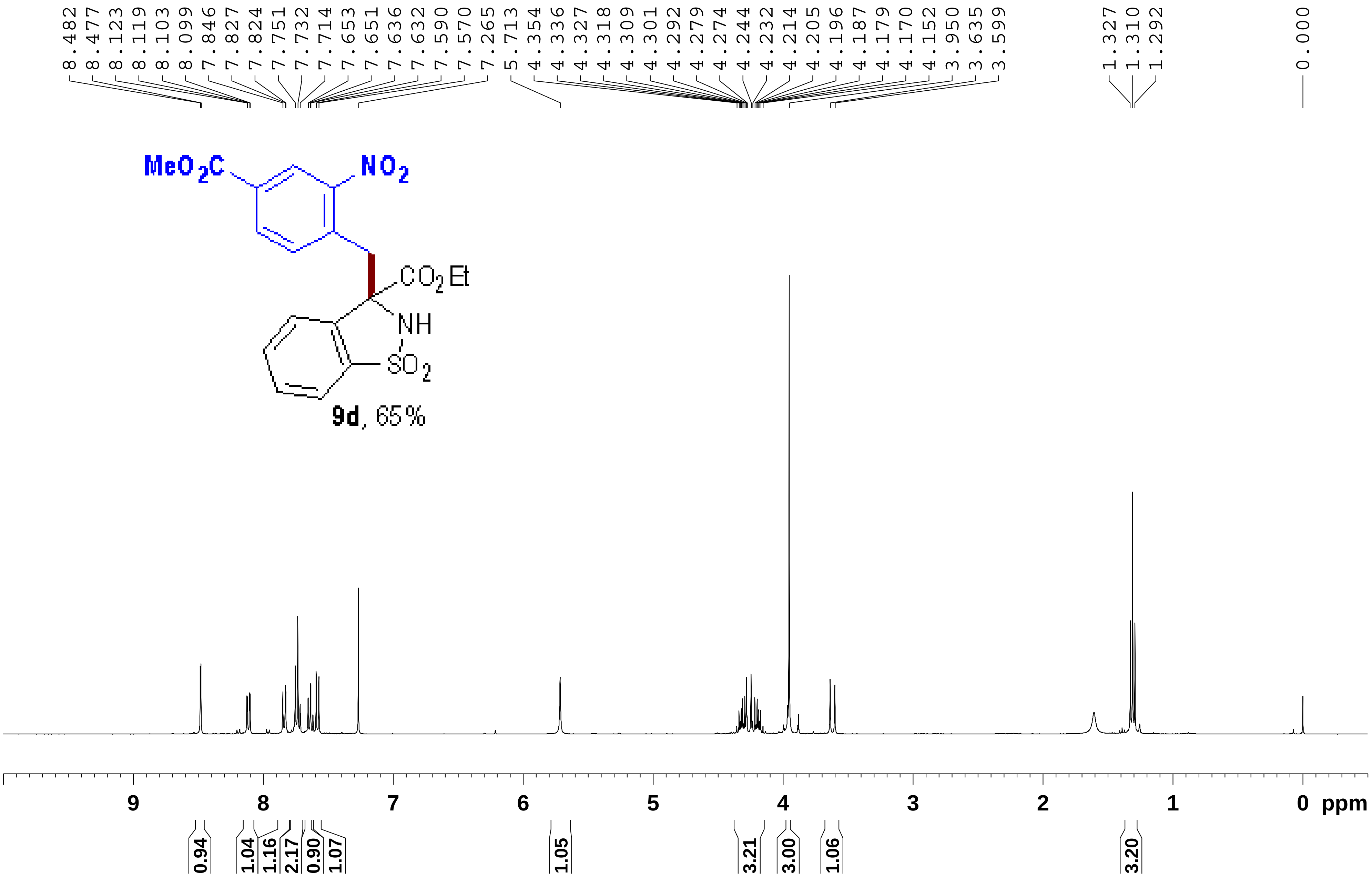

Supplementary Figure 105. ^{13}C NMR Spectrum of substrate 9d

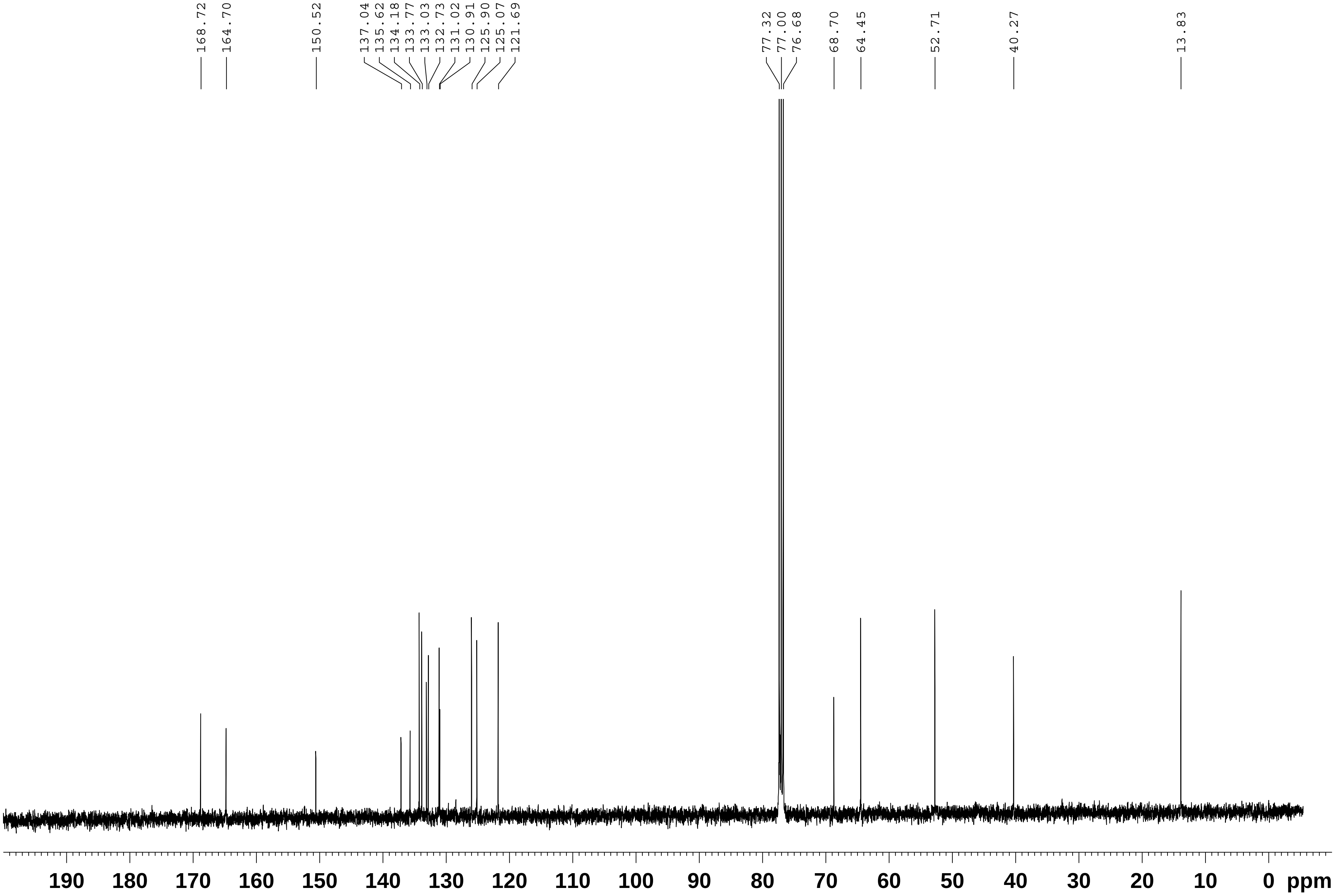

Supplementary Figure 106. ¹H NMR Spectrum of substrate 9e

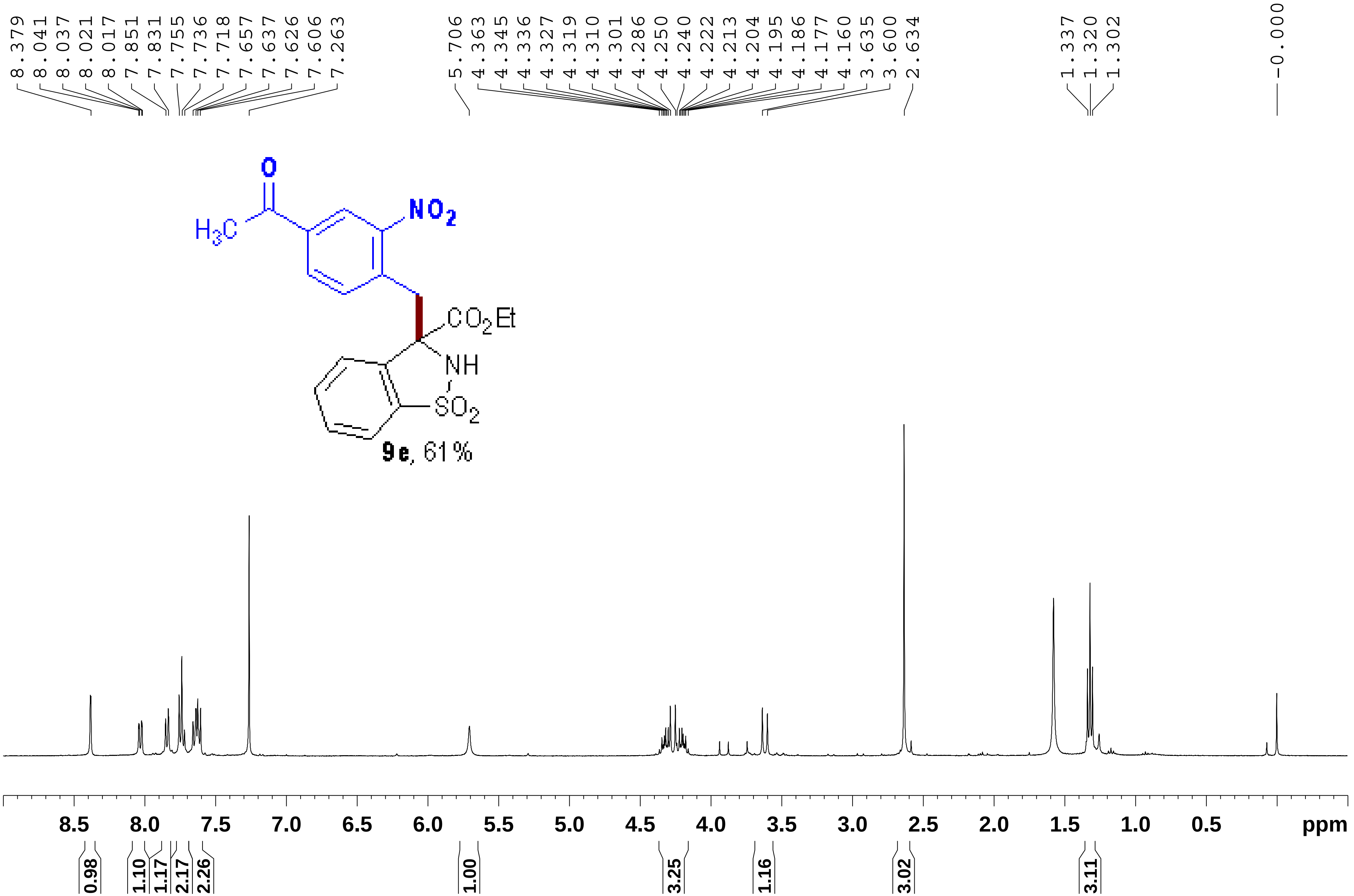

Supplementary Figure 107. ^{13}C NMR Spectrum of substrate 9e

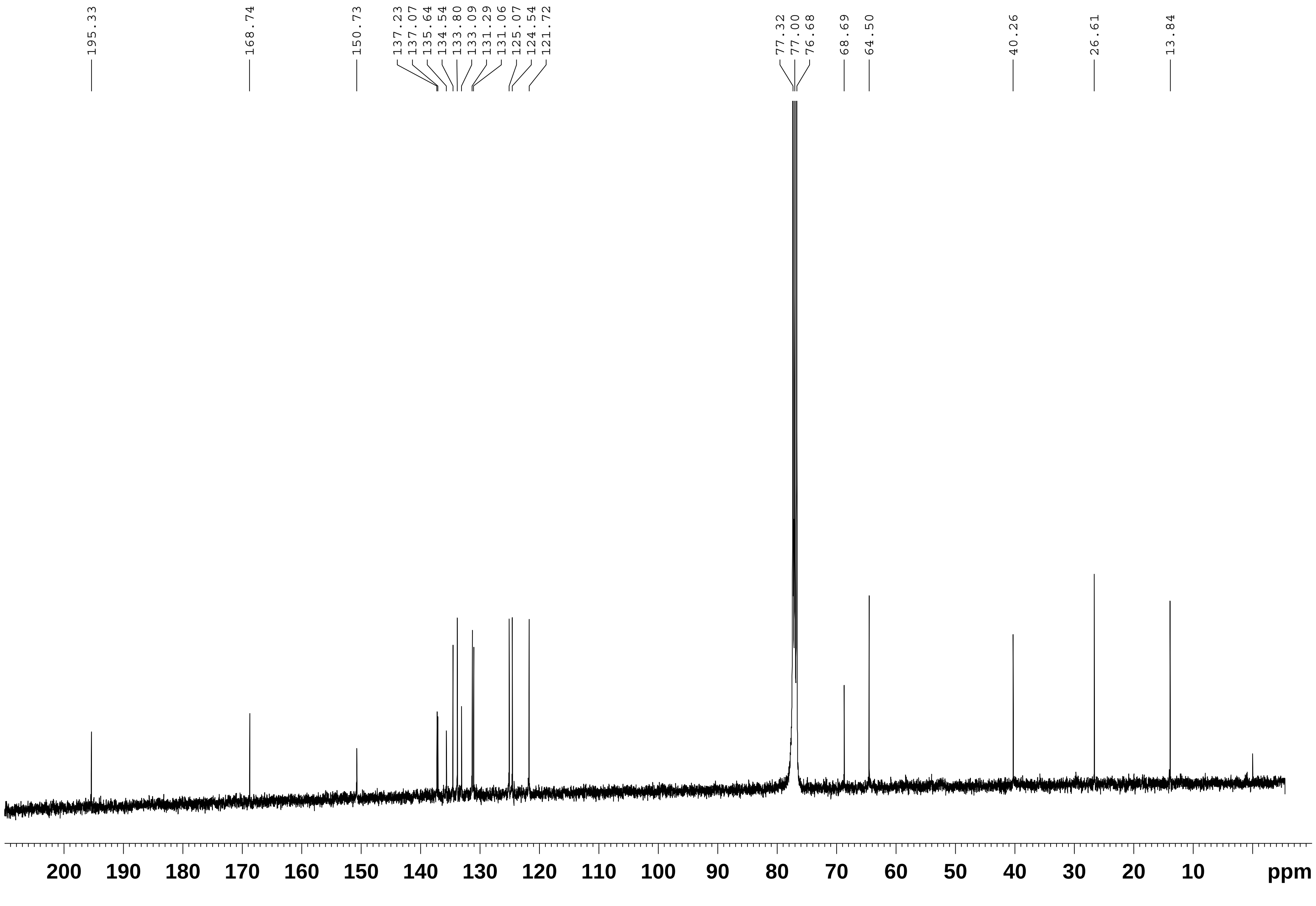

Supplementary Figure 108. ^{1}H NMR Spectrum of substrate 9f

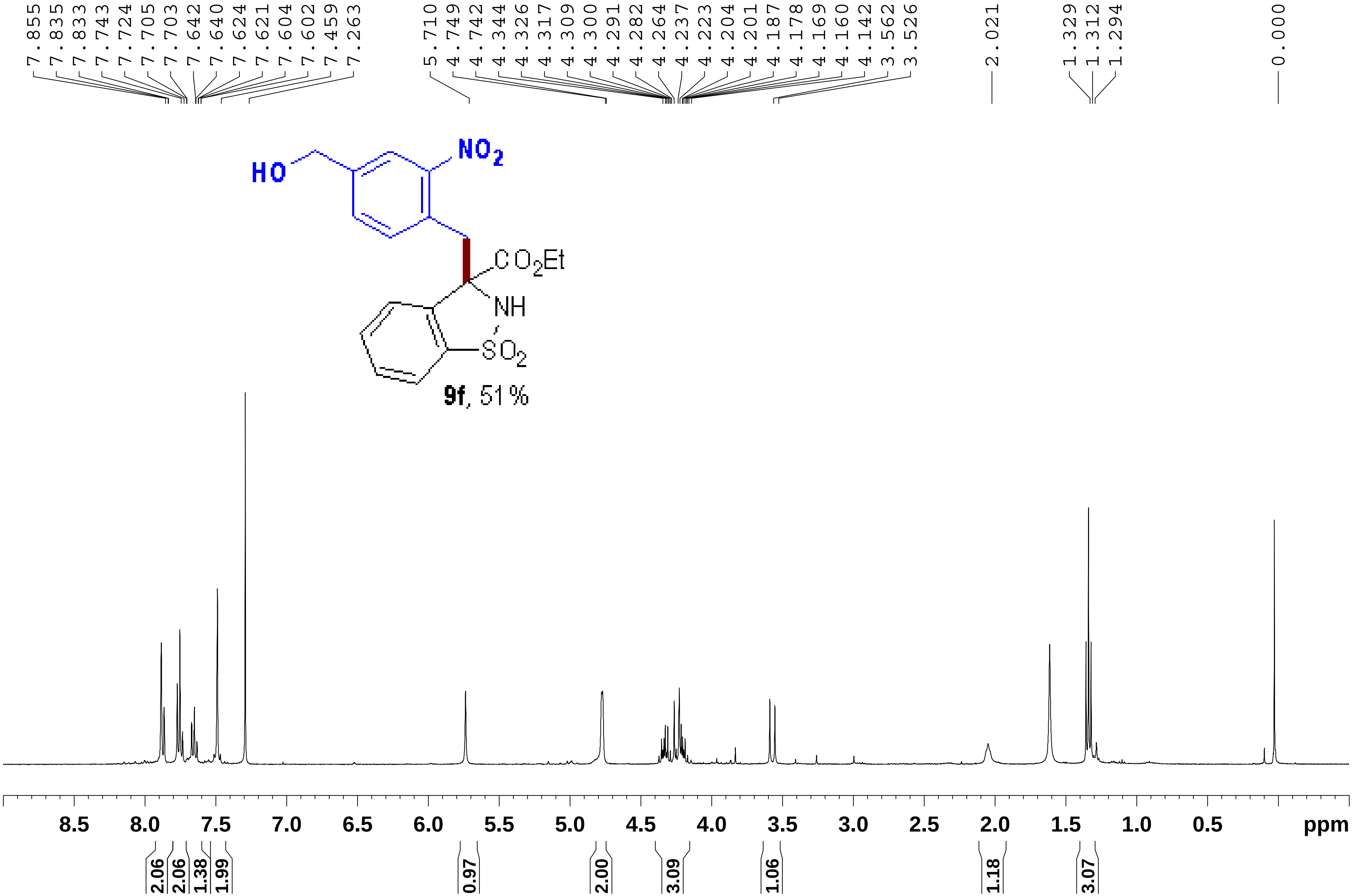

Supplementary Figure 109. ^{13}C NMR Spectrum of substrate 9f

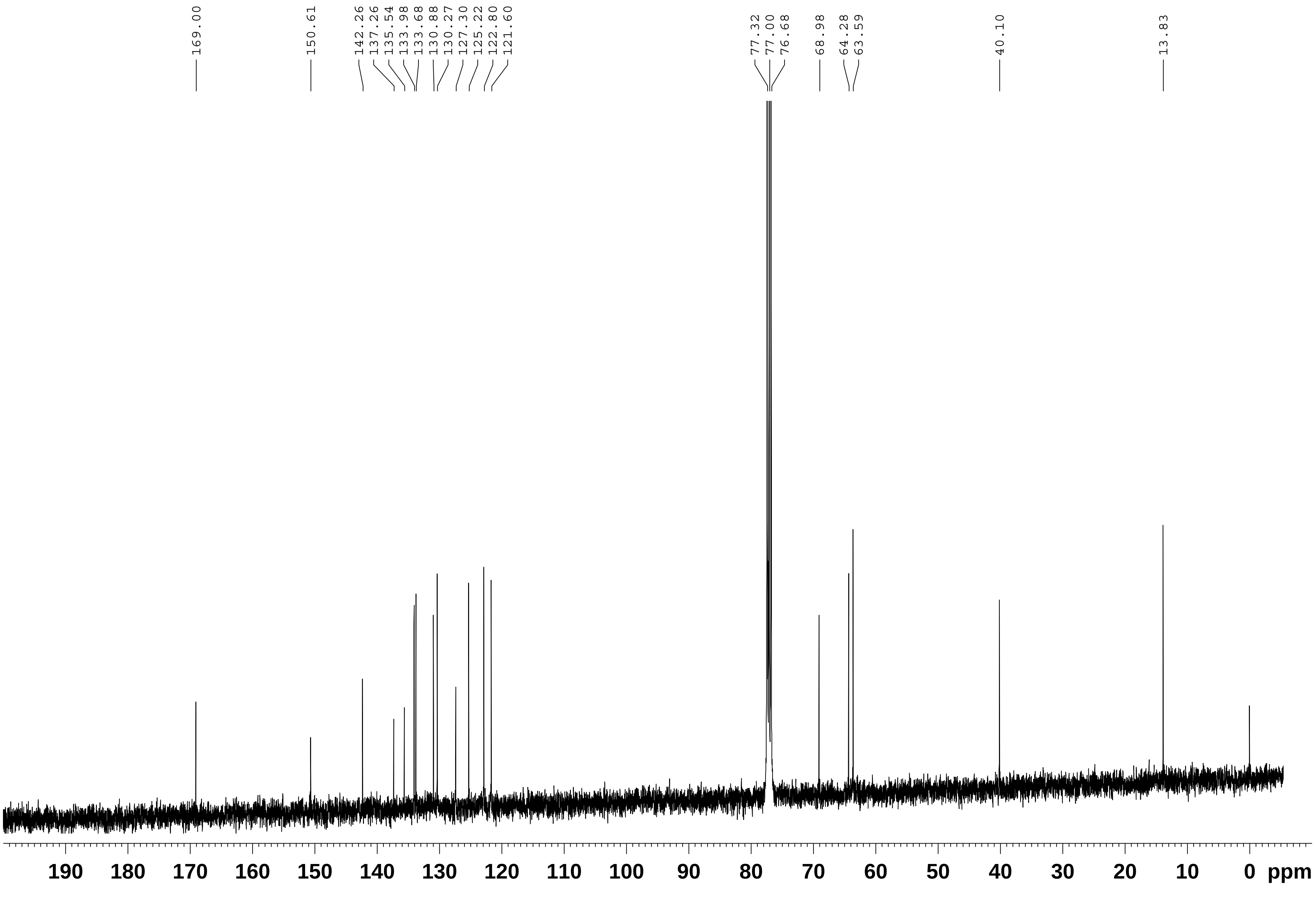

Supplementary Figure 110. ^{1}H NMR Spectrum of substrate 9g

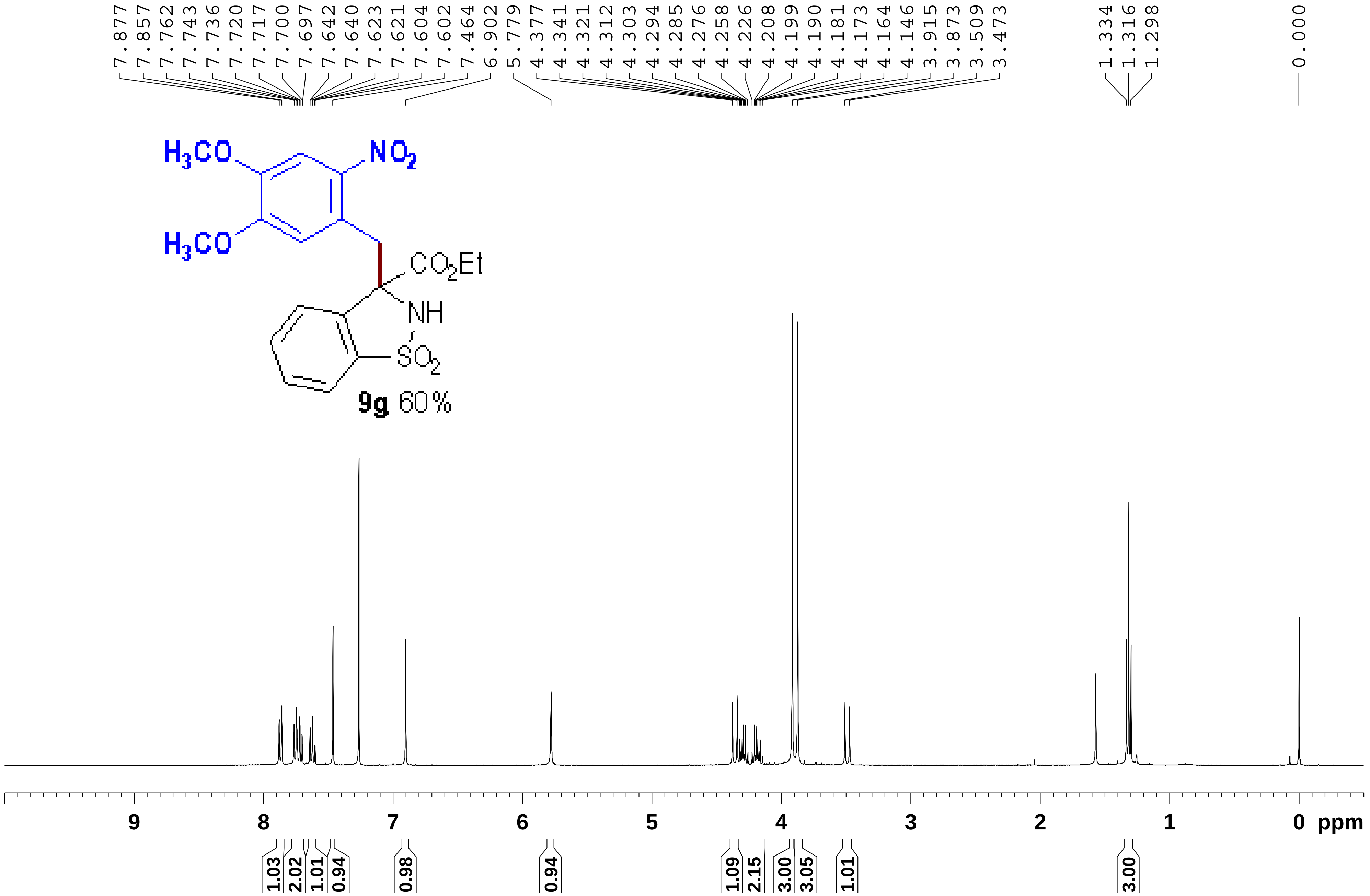

Supplementary Figure 111. ^{13}**C NMR Spectrum of substrate 9g**

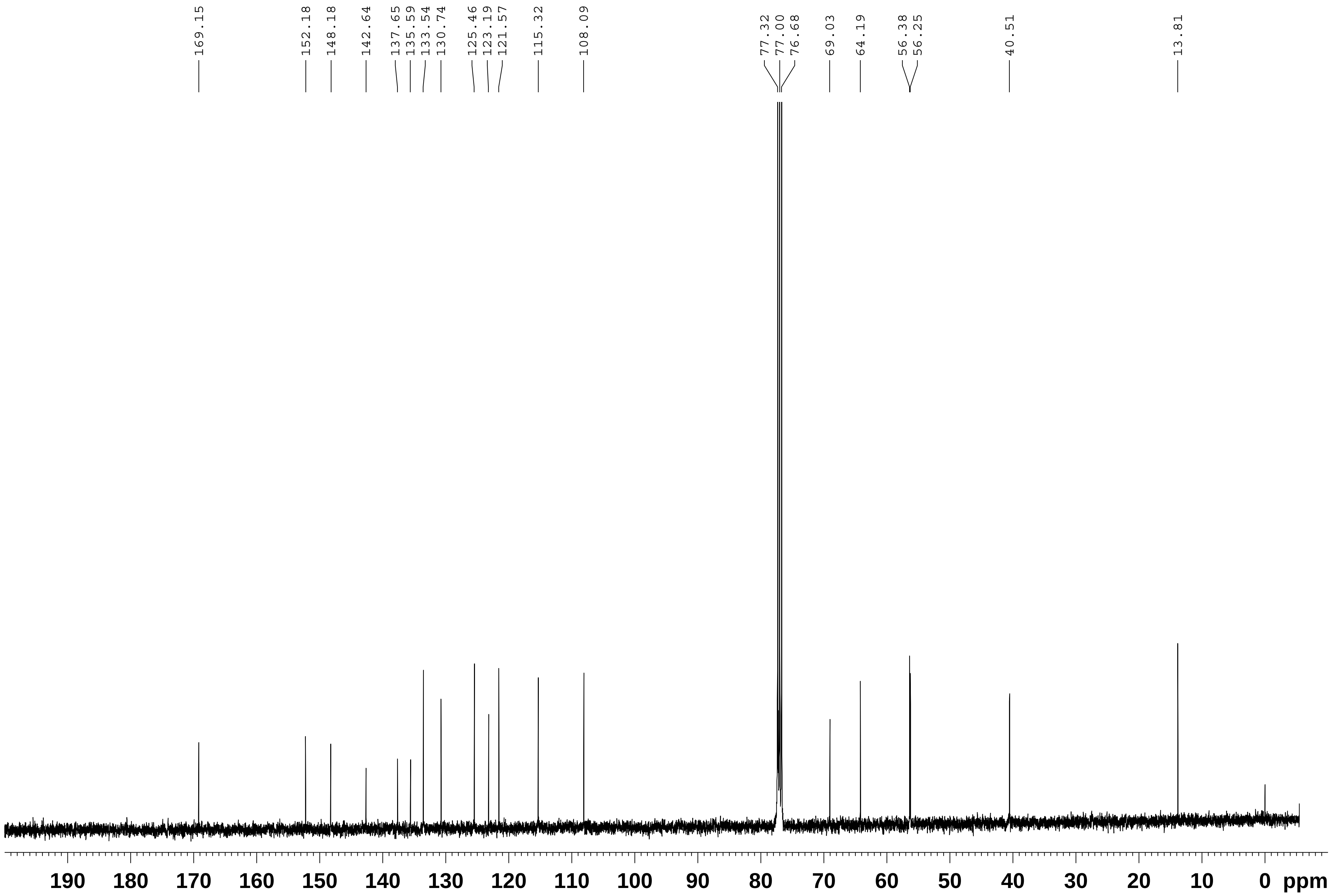

Supplementary Figure 112. ^{1}H NMR Spectrum of substrate 9h

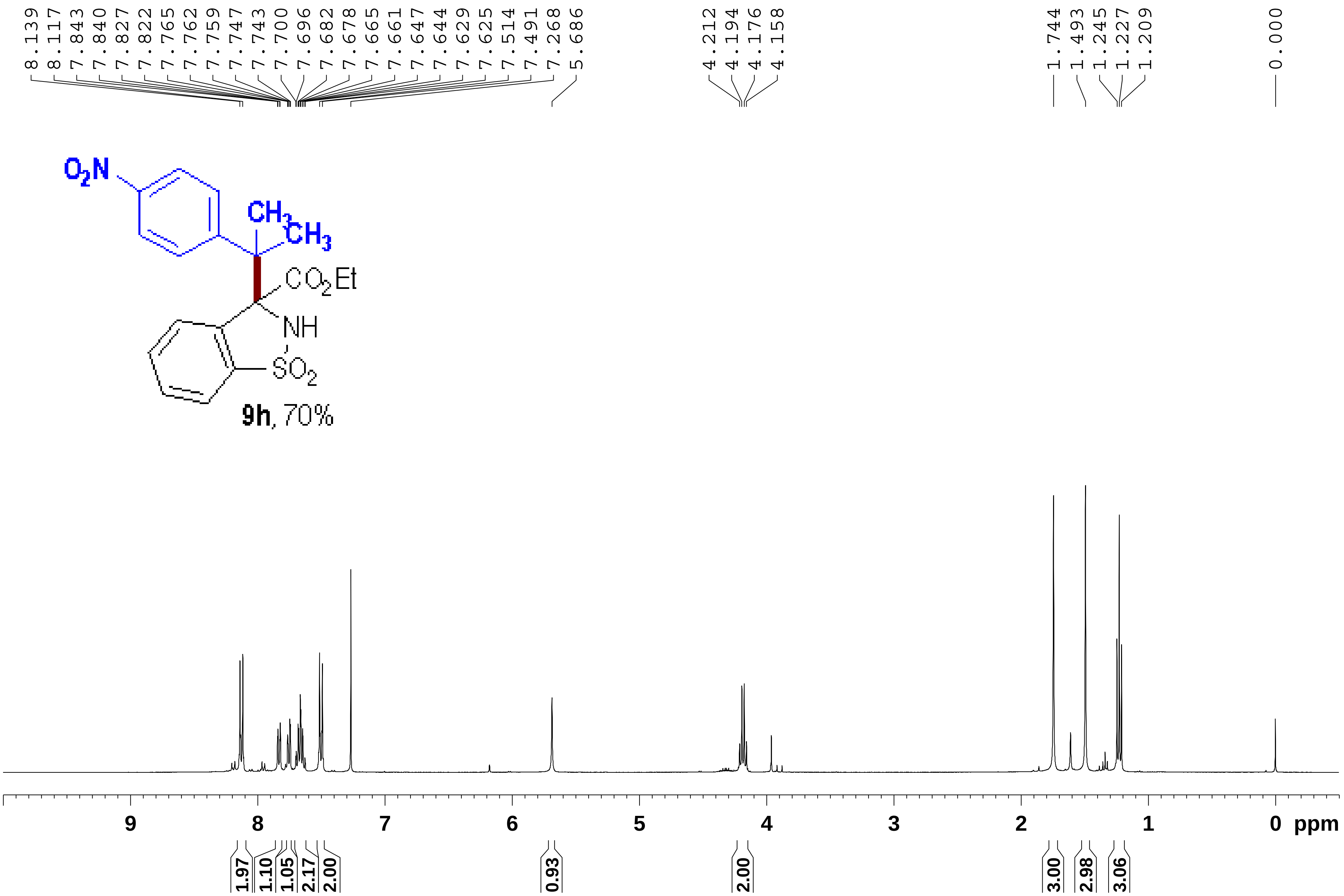

Supplementary Figure 113. ^{13}C NMR Spectrum of substrate 9h

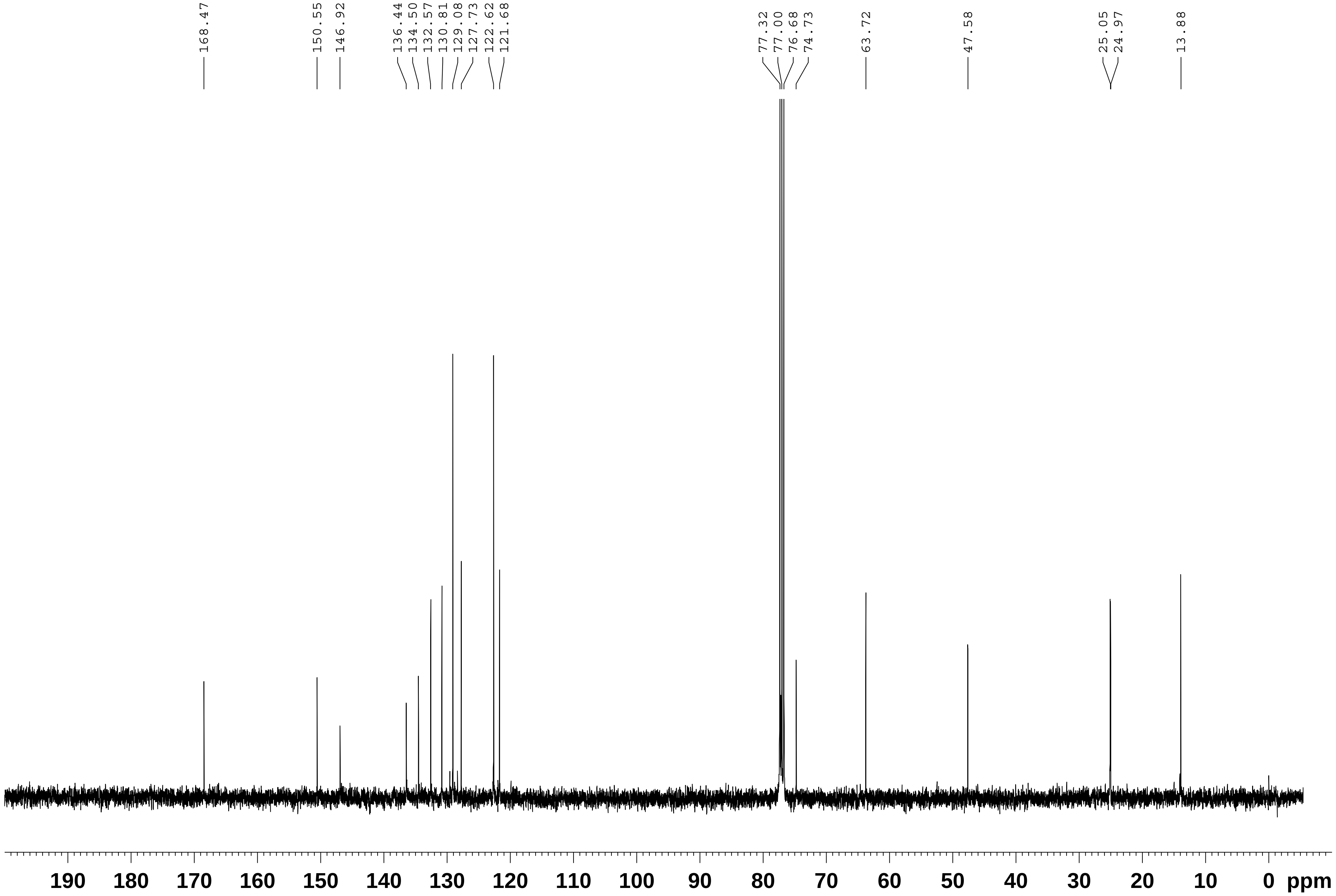

Supplementary Figure 114. ^{1}H NMR Spectrum of substrate 9i

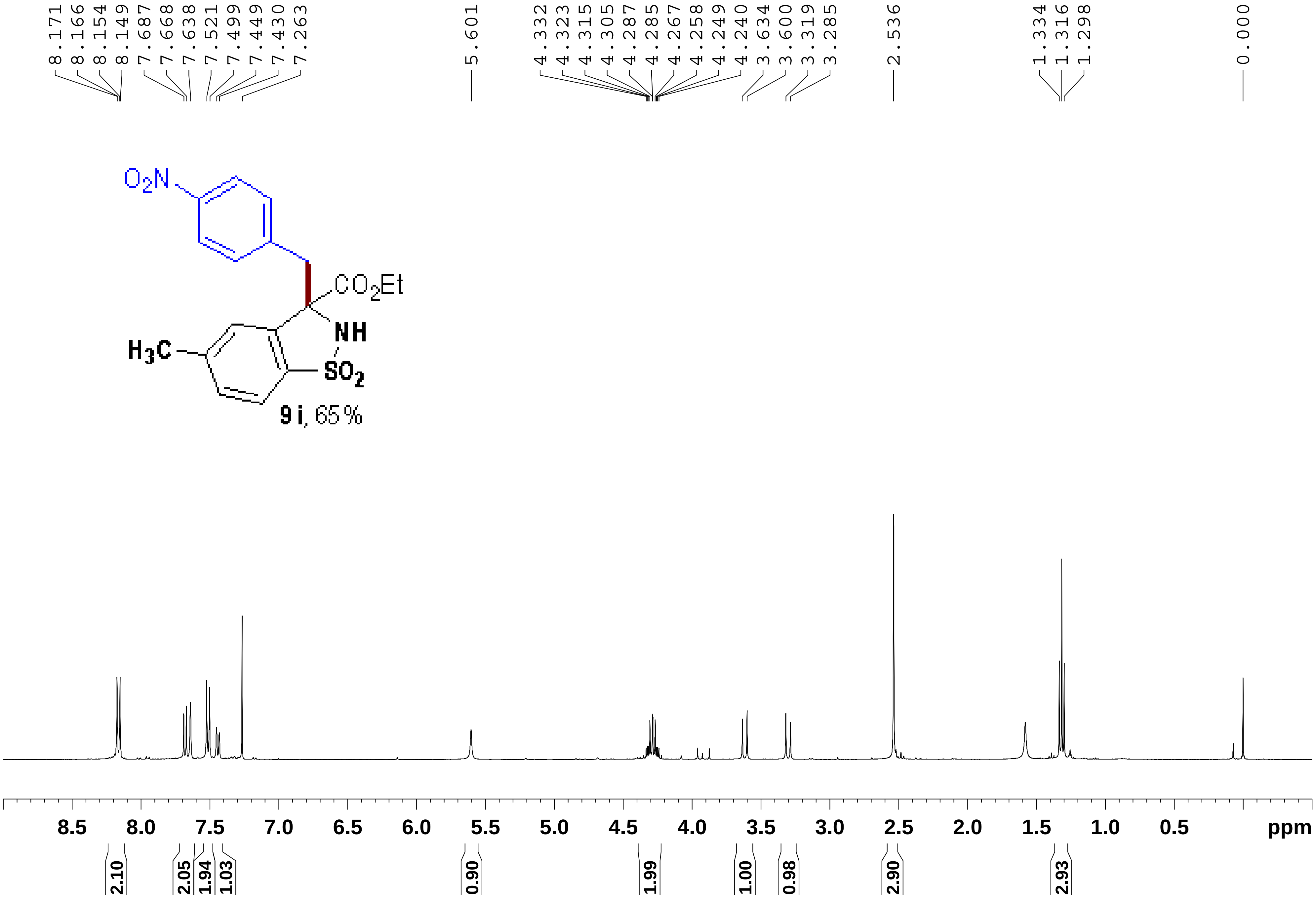

Supplementary Figure 115. ^{13}C NMR Spectrum of substrate 9i

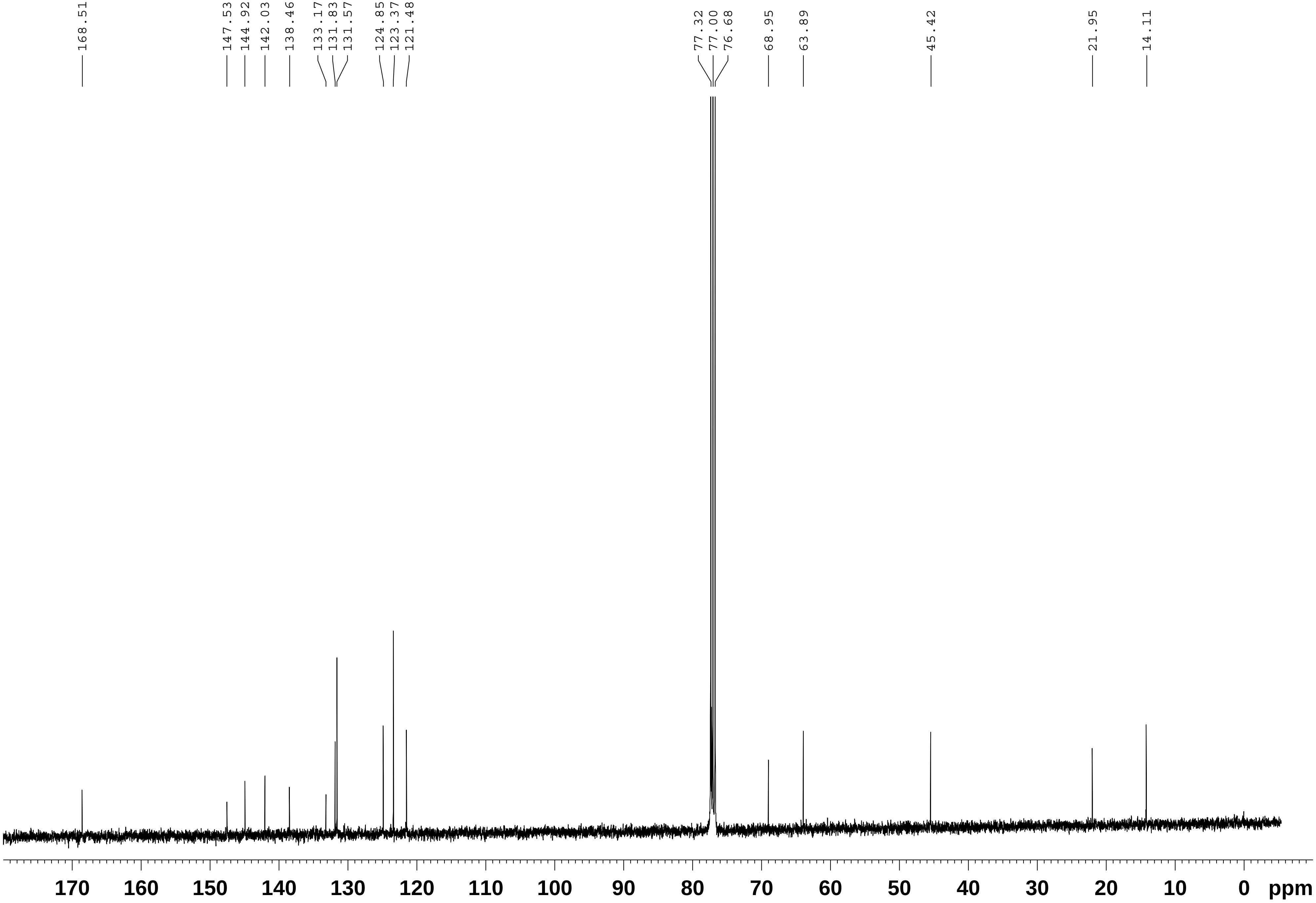

Supplementary Figure 116. ¹H NMR Spectrum of substrate 9j

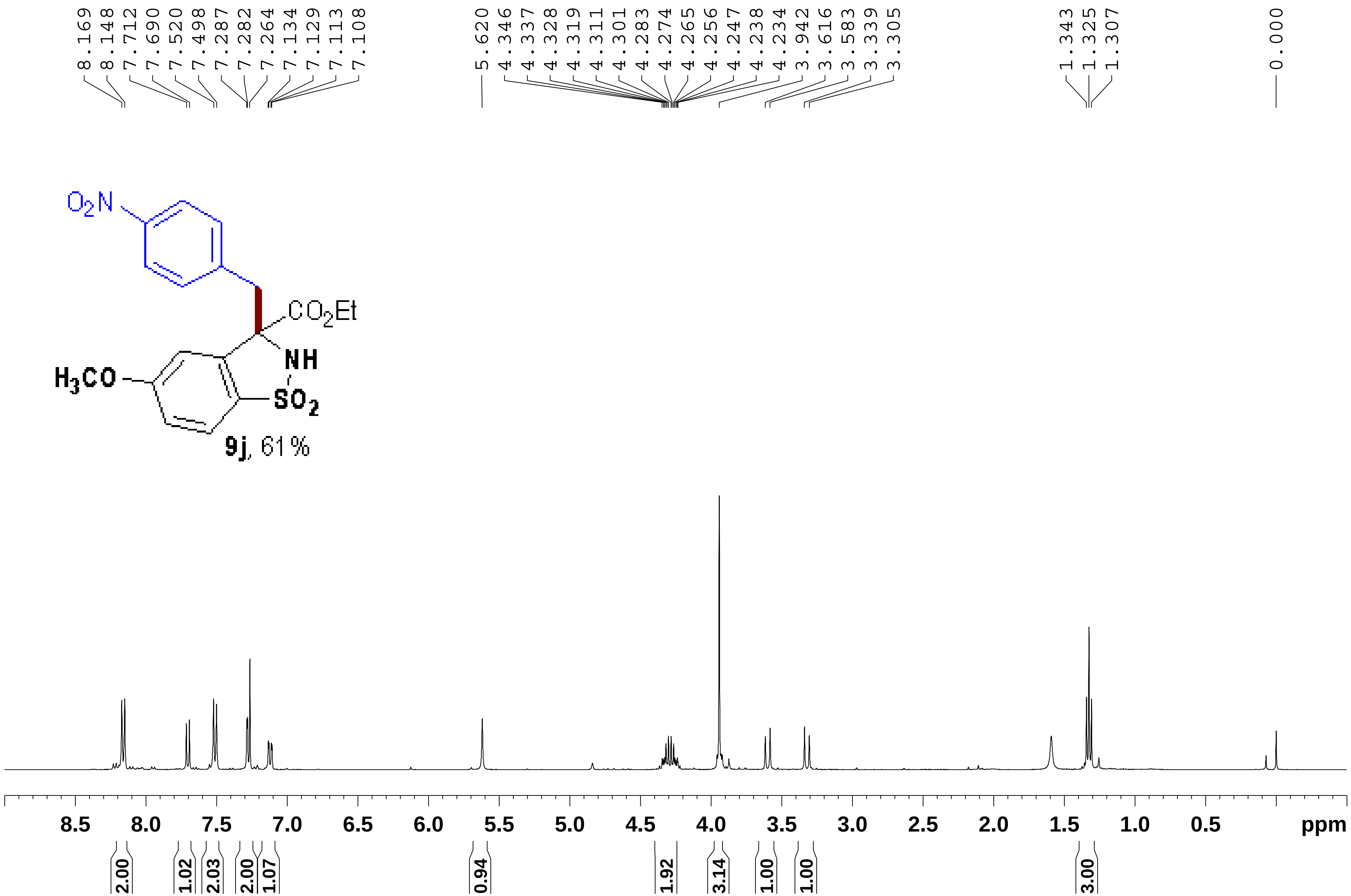

Supplementary Figure 117. ^{13}C NMR Spectrum of substrate 9j

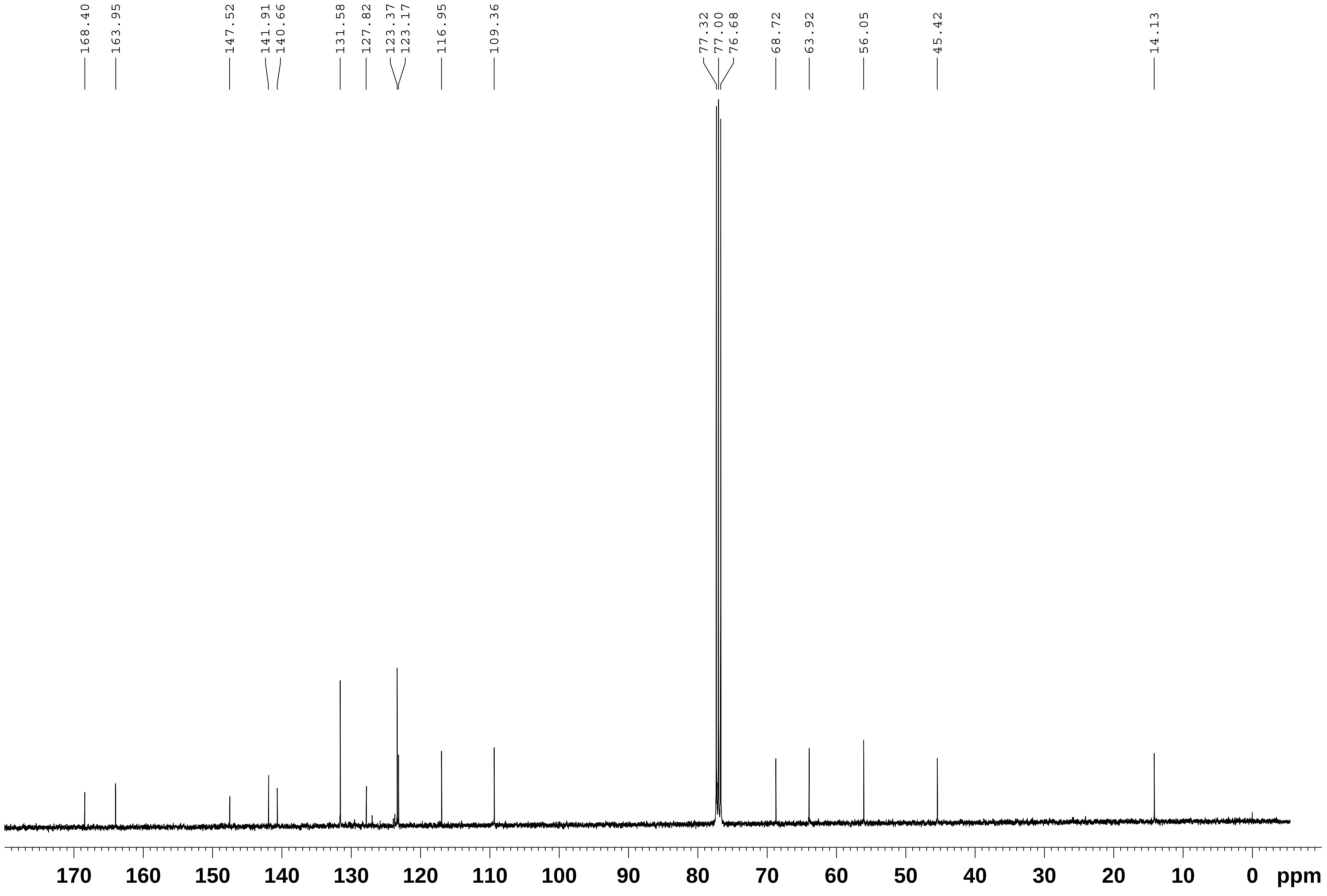

Supplementary Figure 118. ^{1}H NMR Spectrum of substrate 4b-1

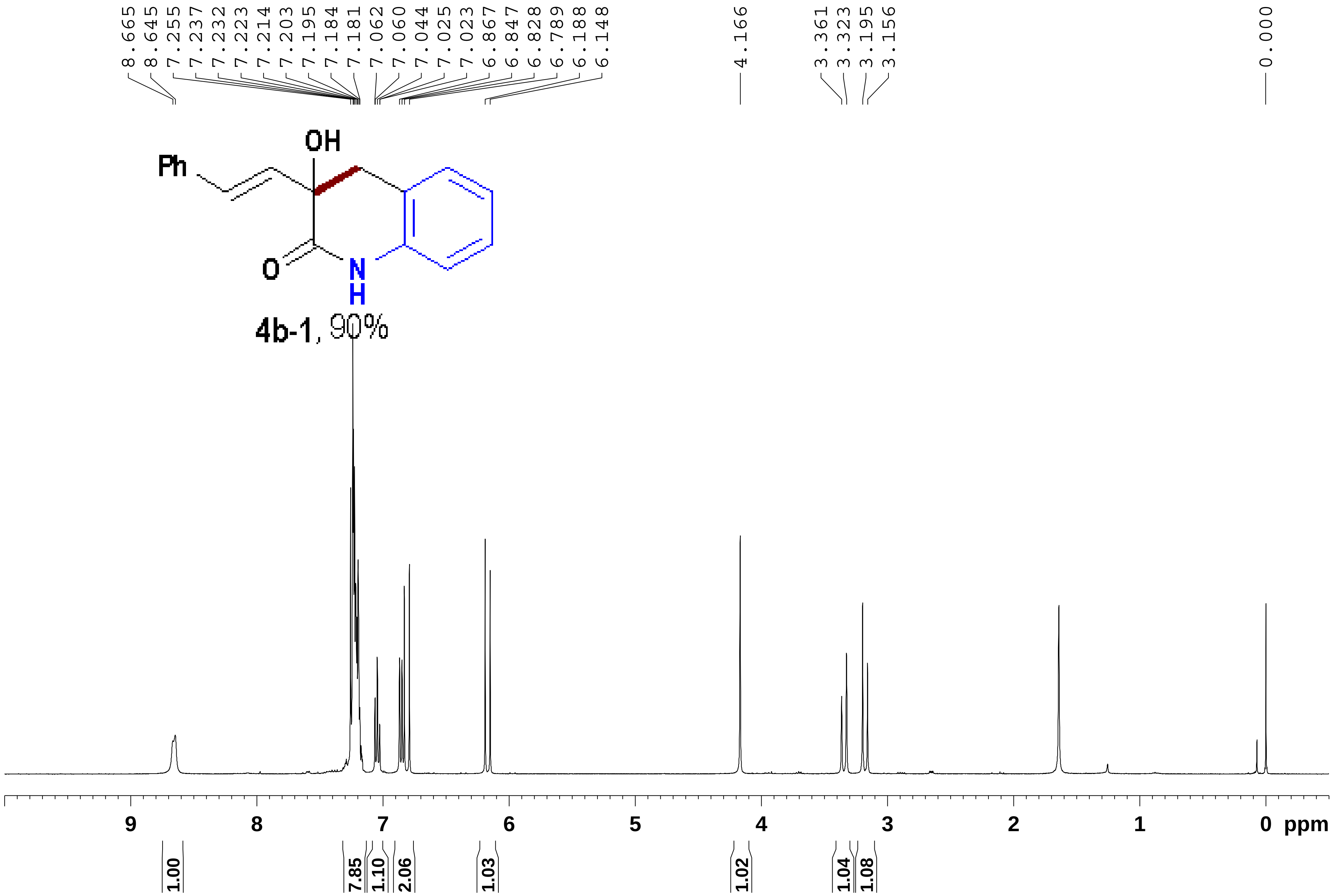

Supplementary Figure 119. ^{13}C NMR Spectrum of substrate 4b-1

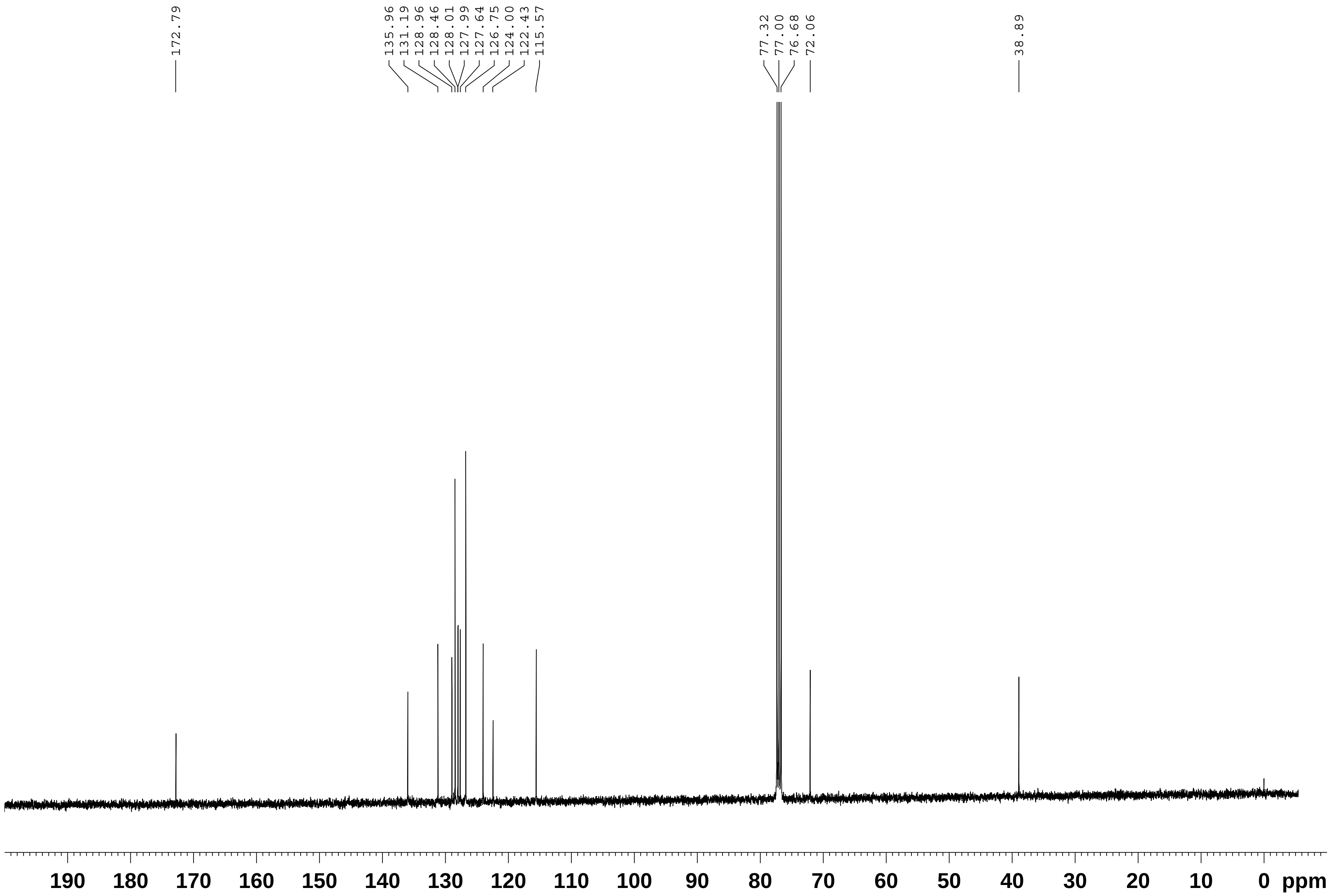

Supplementary Figure 120. ^{1}H NMR Spectrum of substrate 4b-2

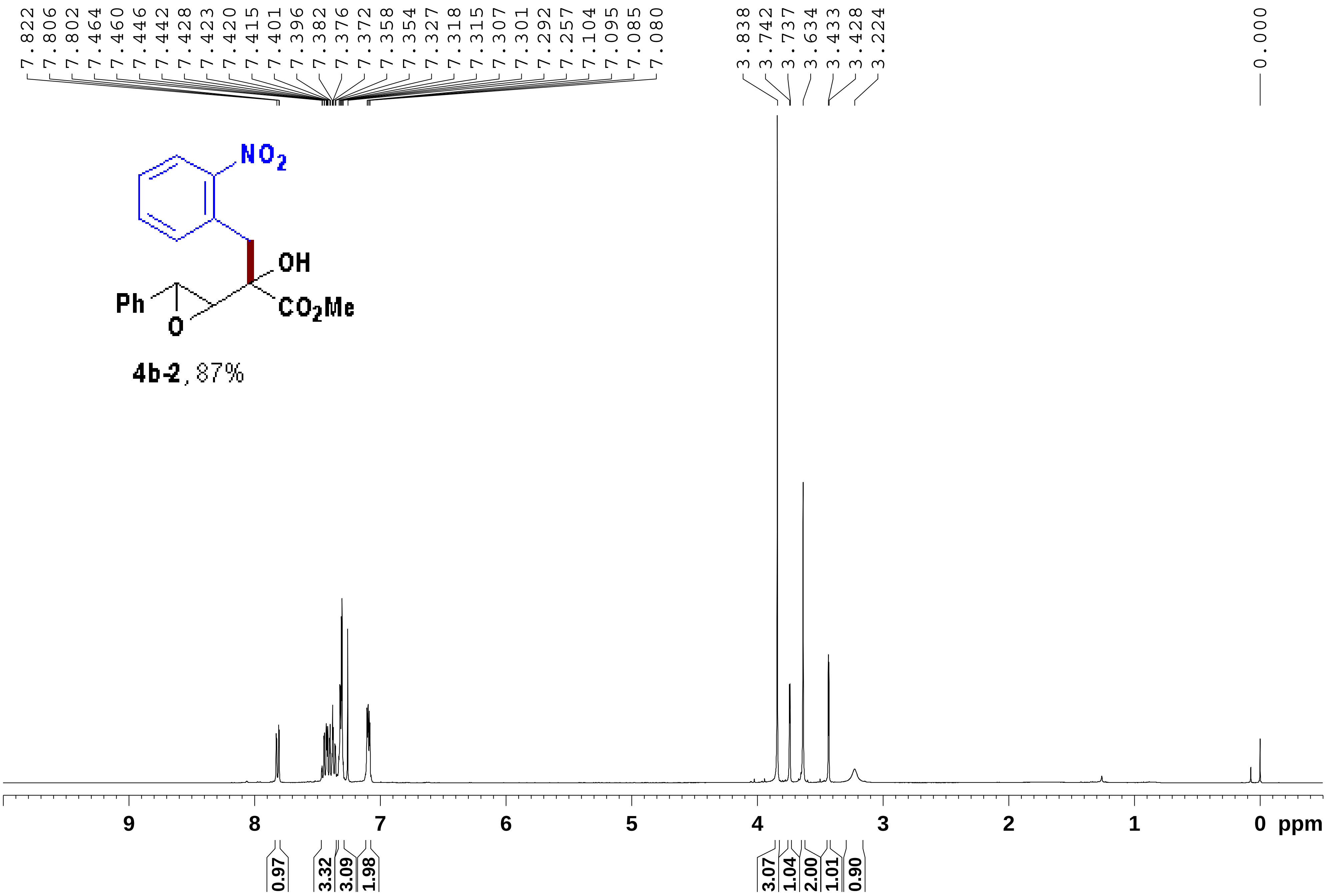

Supplementary Figure 121. ^{13}C NMR Spectrum of substrate 4b-2

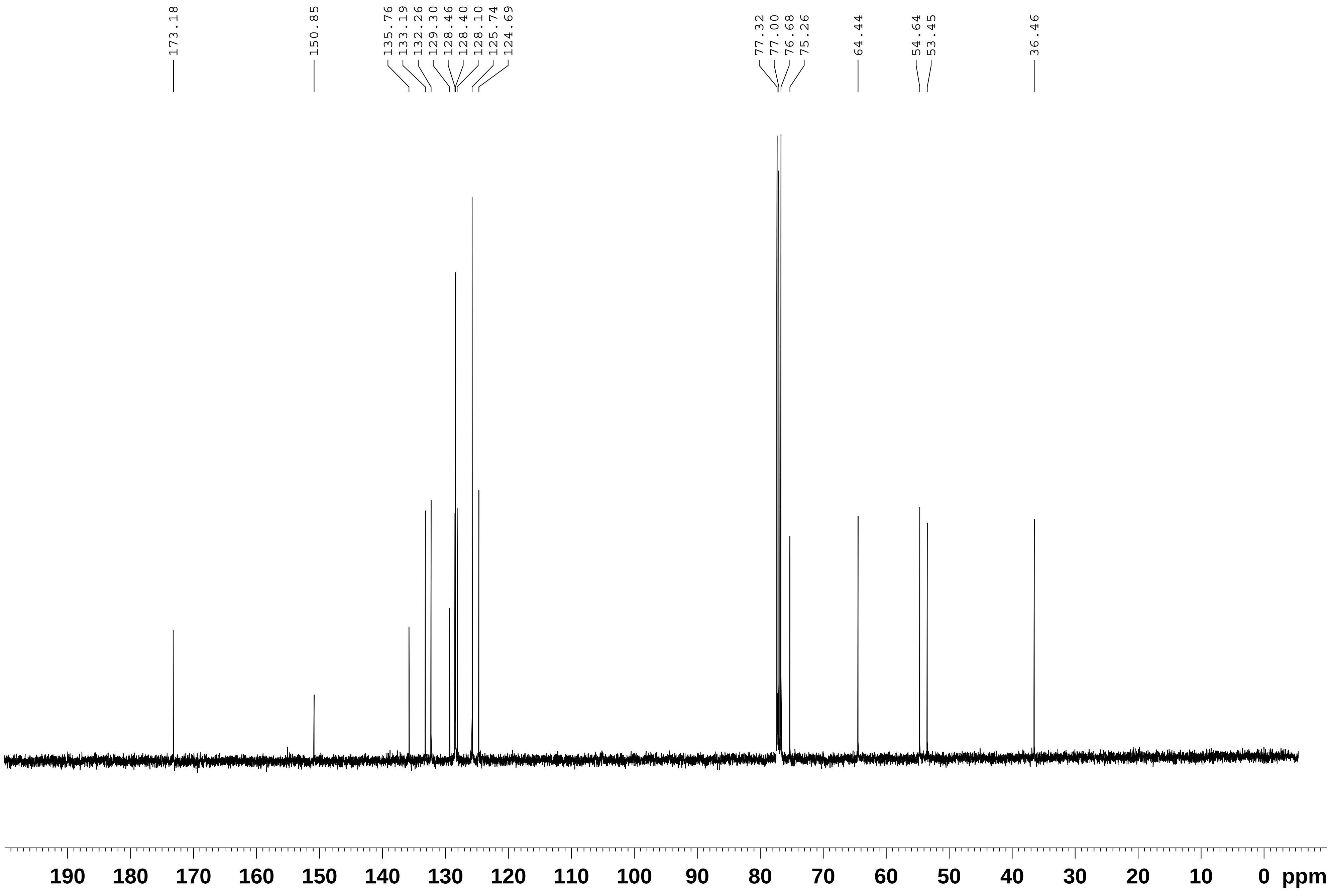

Supplementary Figure 122. ^{1}**H NMR Spectrum of substrate 4b-3**

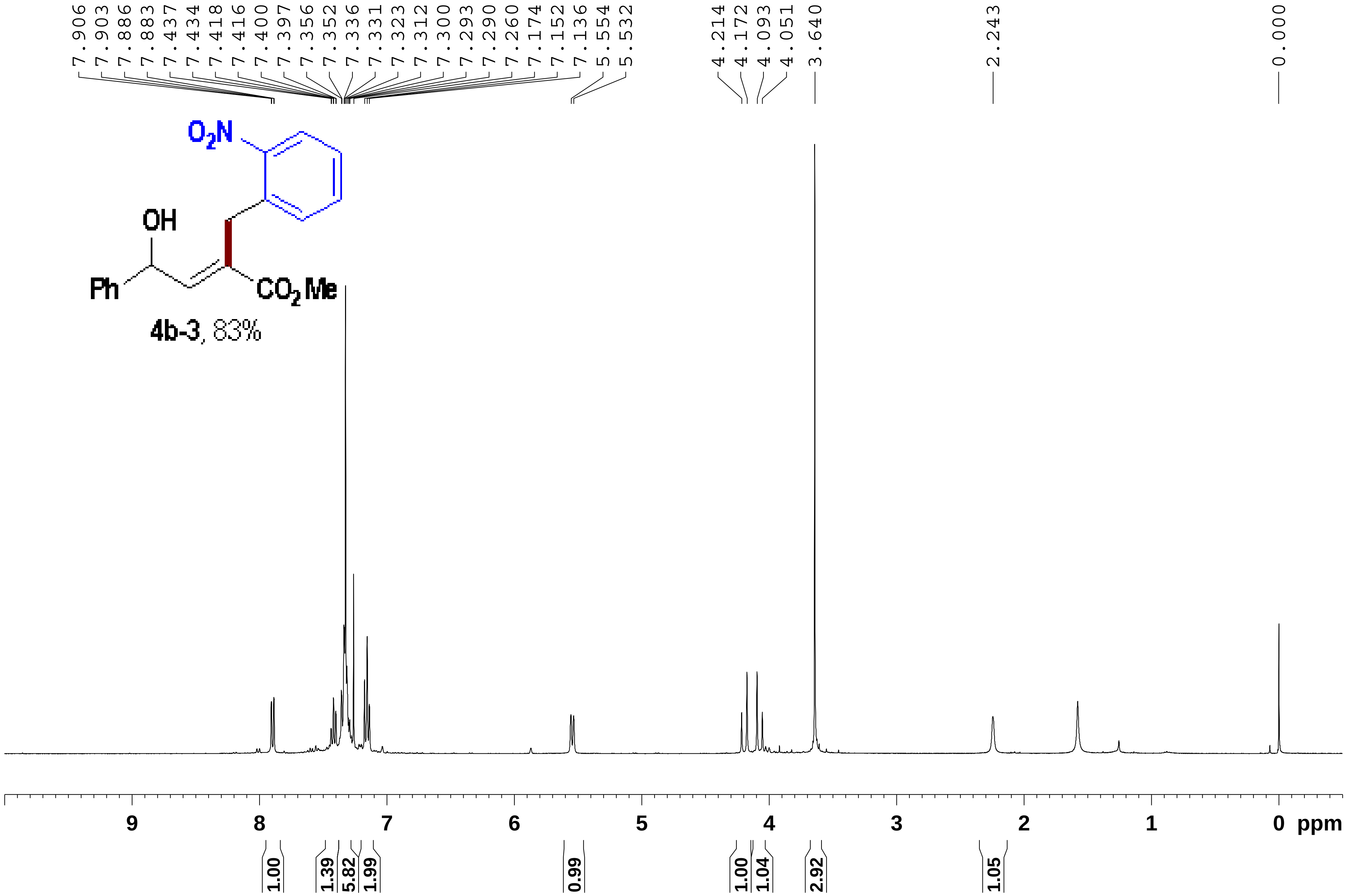

Supplementary Figure 123. ^{13}C NMR Spectrum of substrate 4b-3

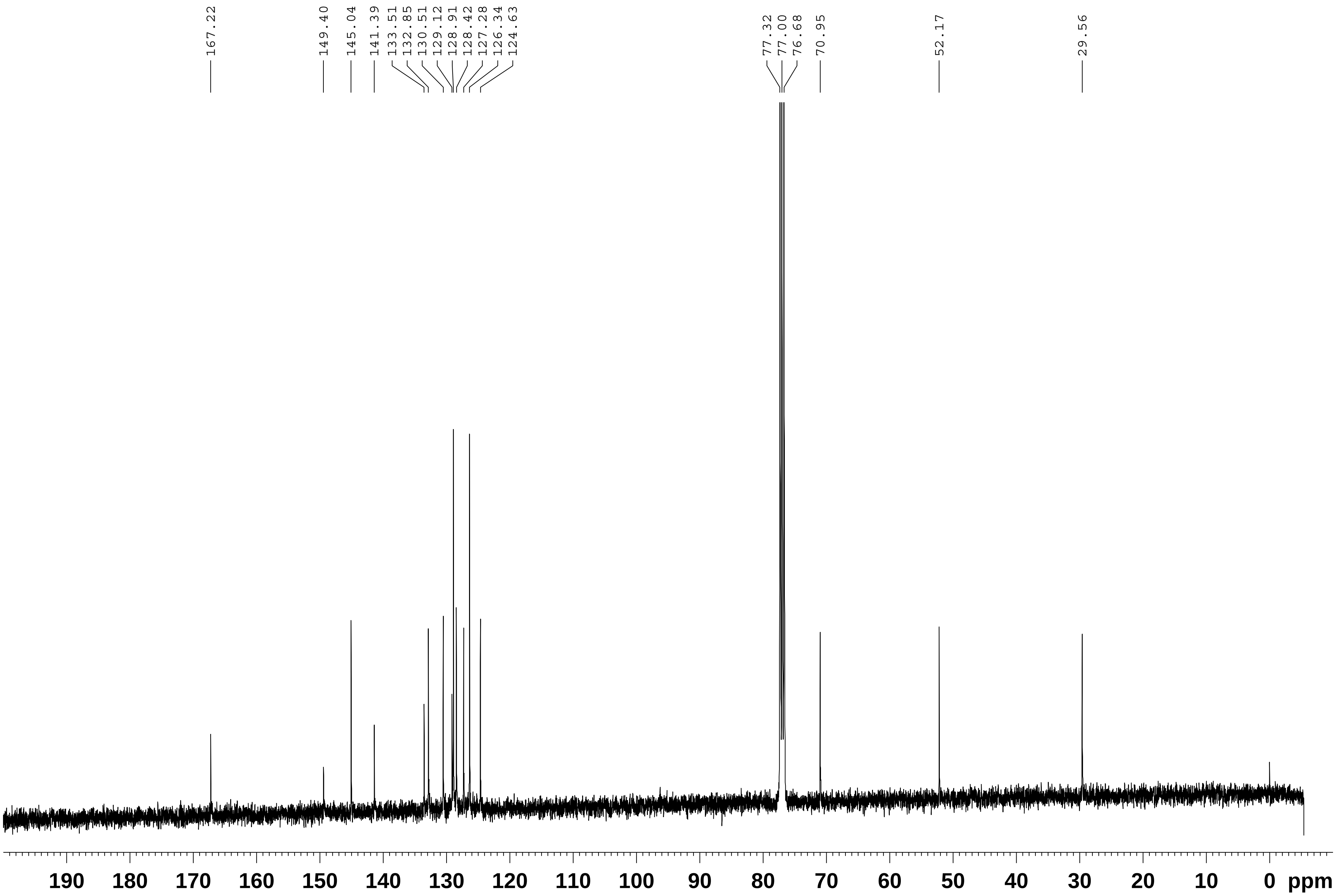

Supplementary Figure 124. ^{1}H NMR Spectrum of substrate 7u-1

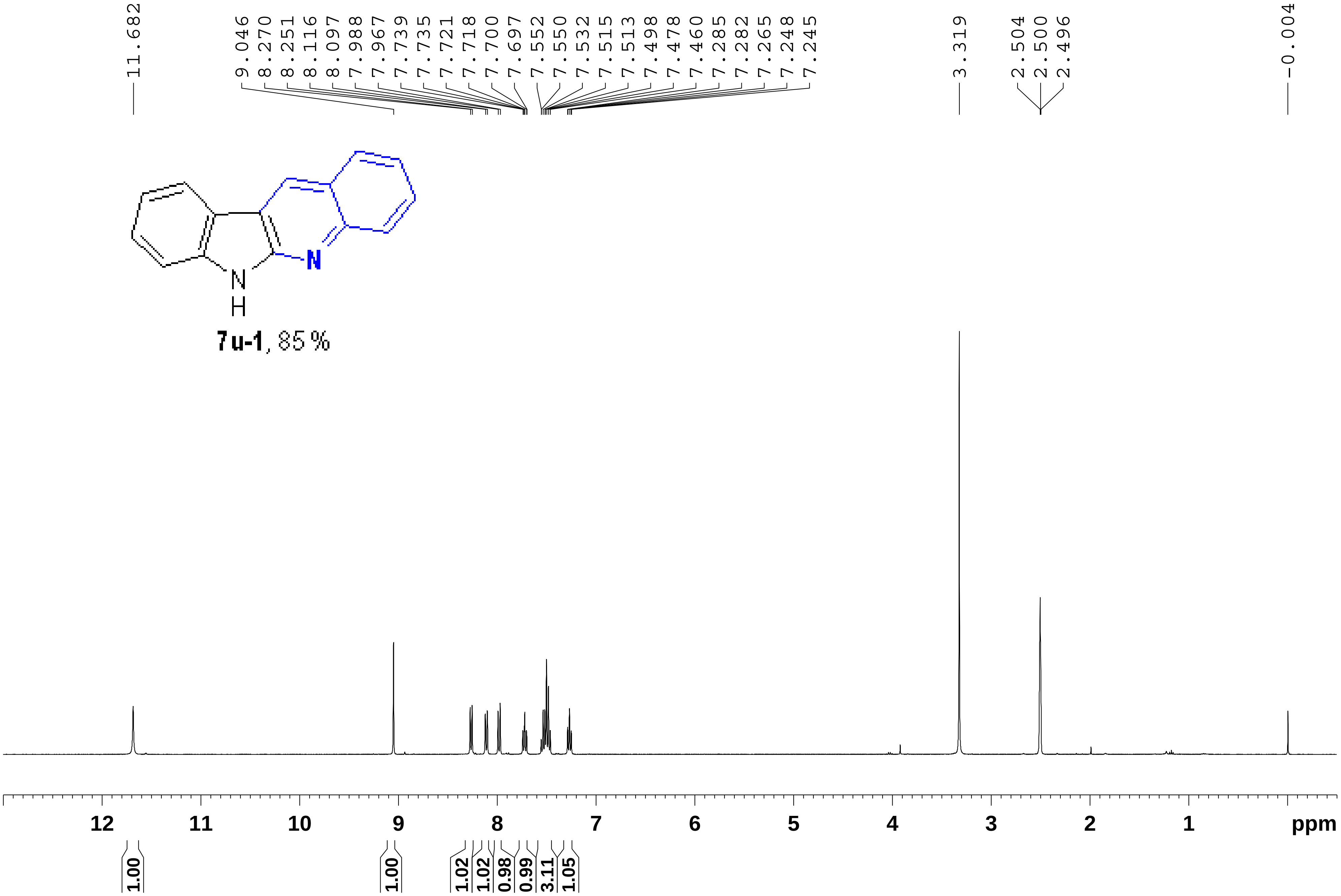

Supplementary Figure 125. ^{13}C NMR Spectrum of substrate 7u-2

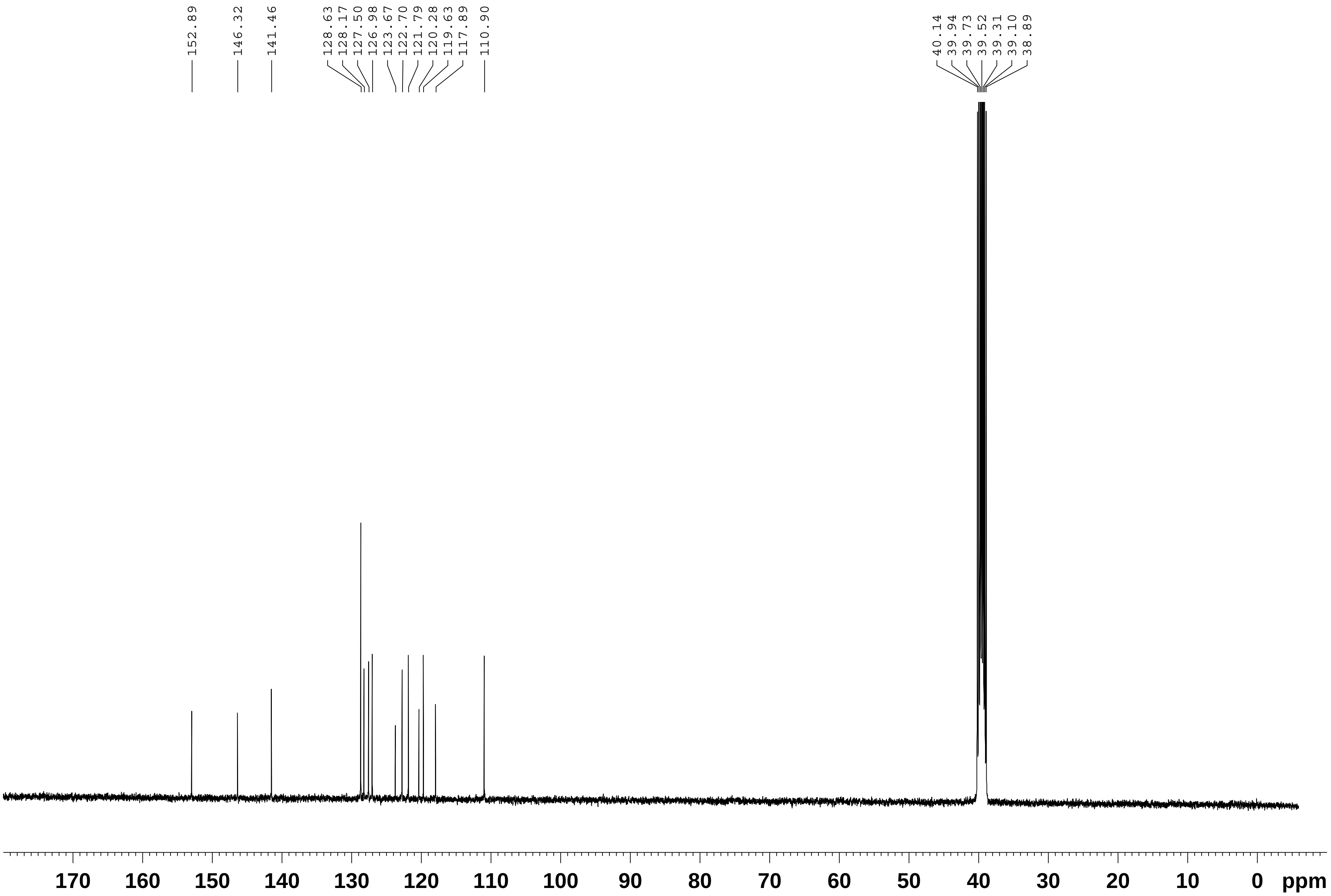

Supplementary Figure 126. ^{1}H NMR Spectrum of substrate neocryptolepine

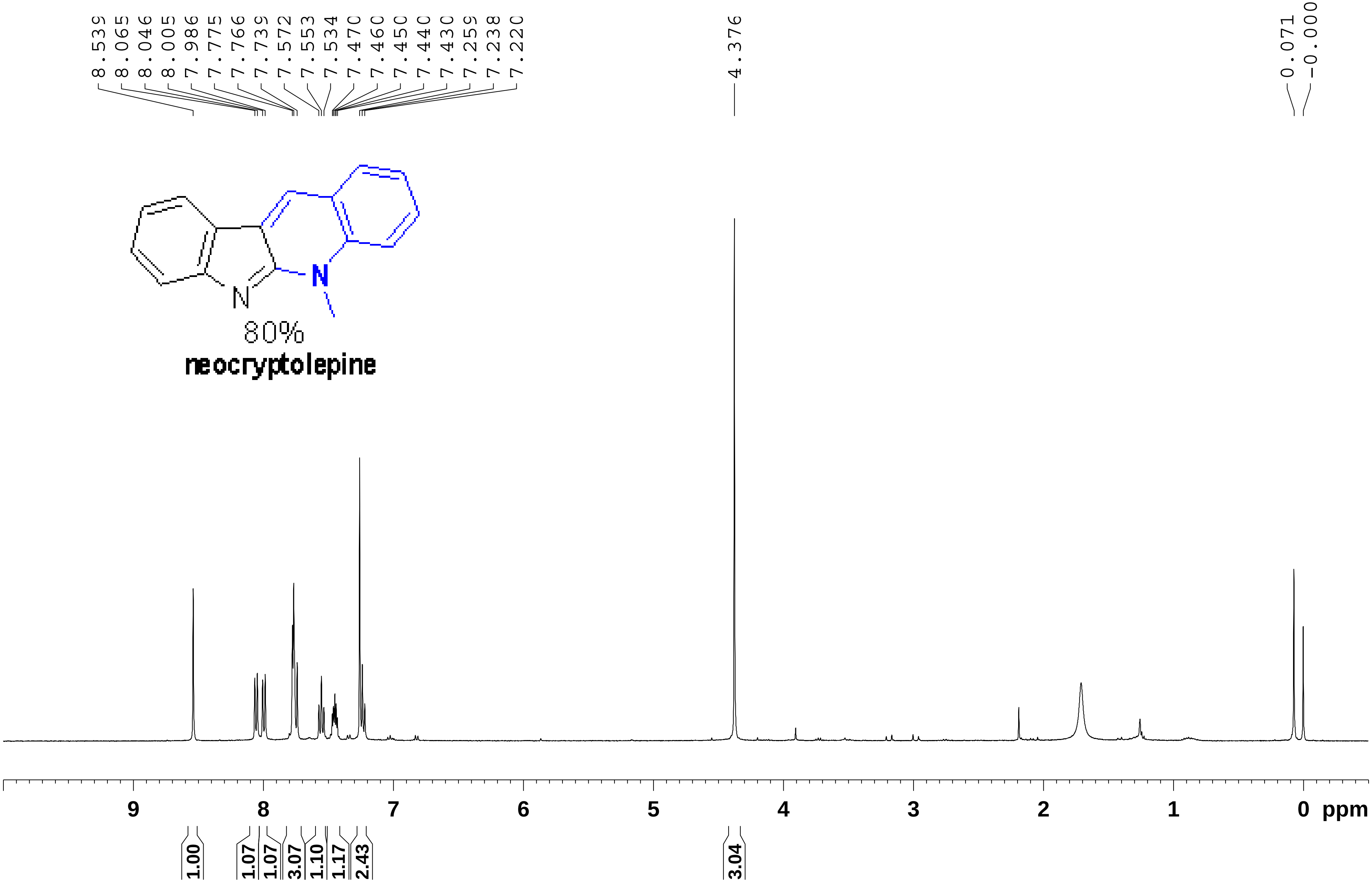

Supplementary Figure 126. ^{13}C NMR Spectrum of substrate neocryptolepine

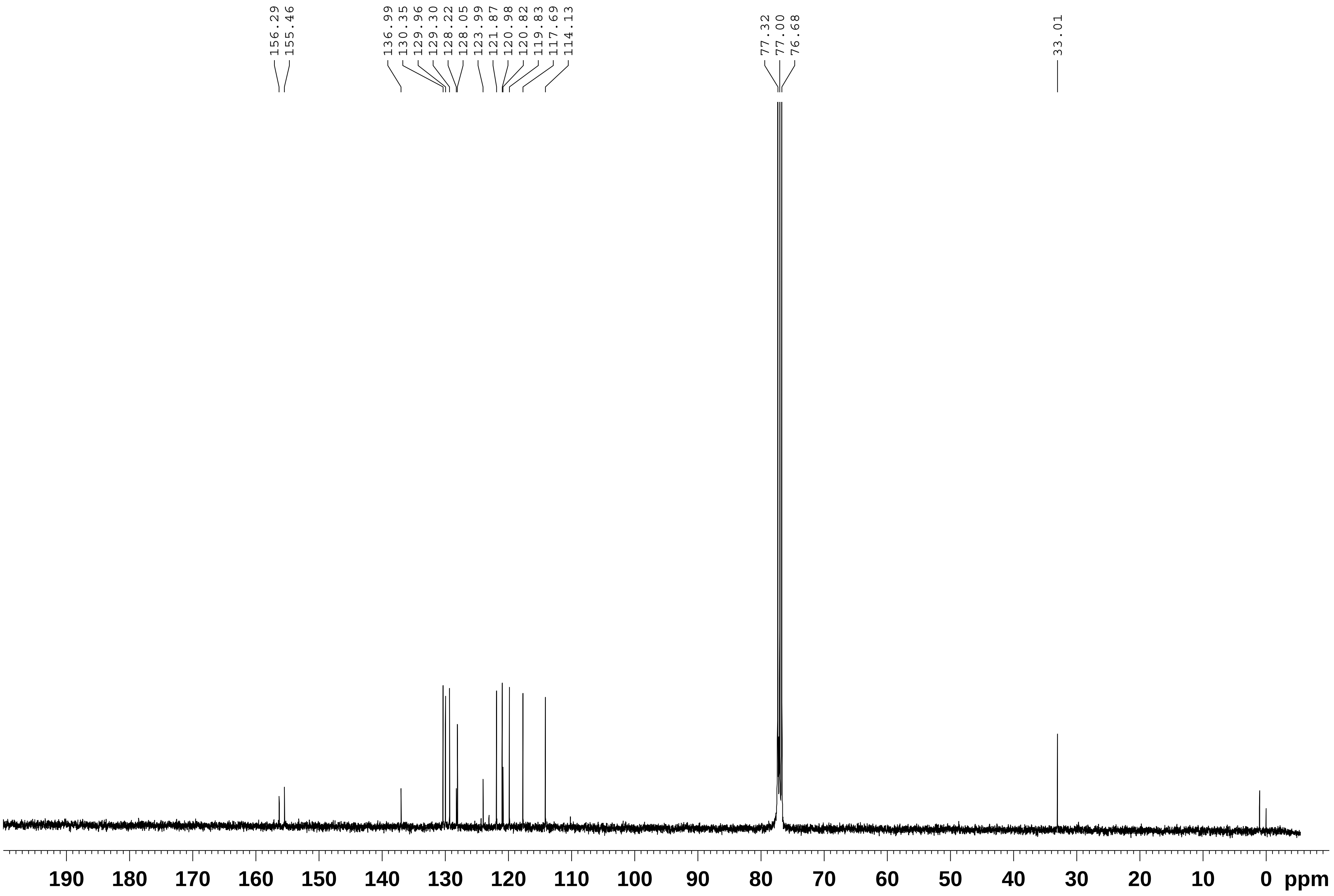

Supplementary Figure 128. ^{1}H NMR Spectrum of substrate 12a

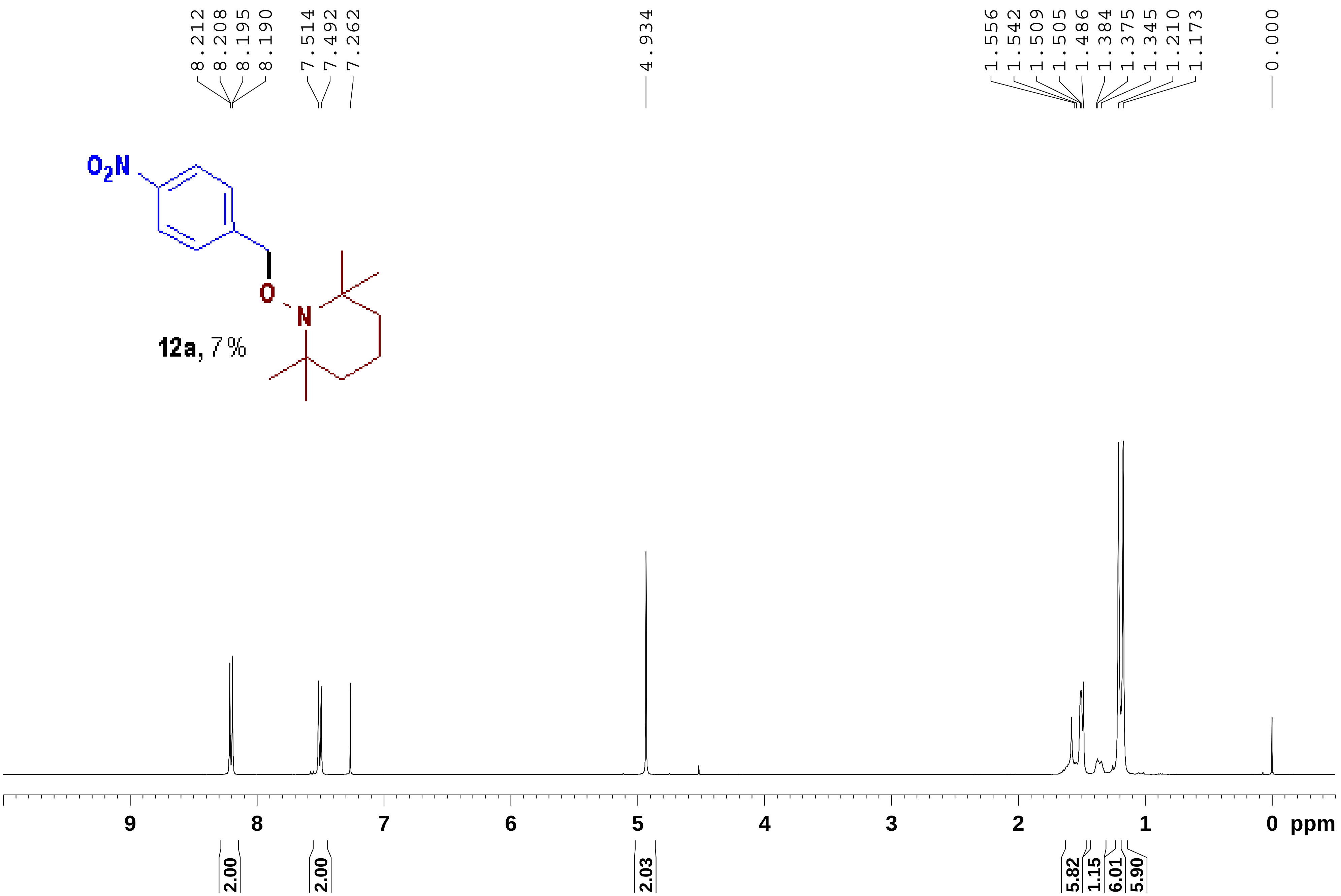

Supplementary Figure 129. ^{13}C NMR Spectrum of substrate 12a

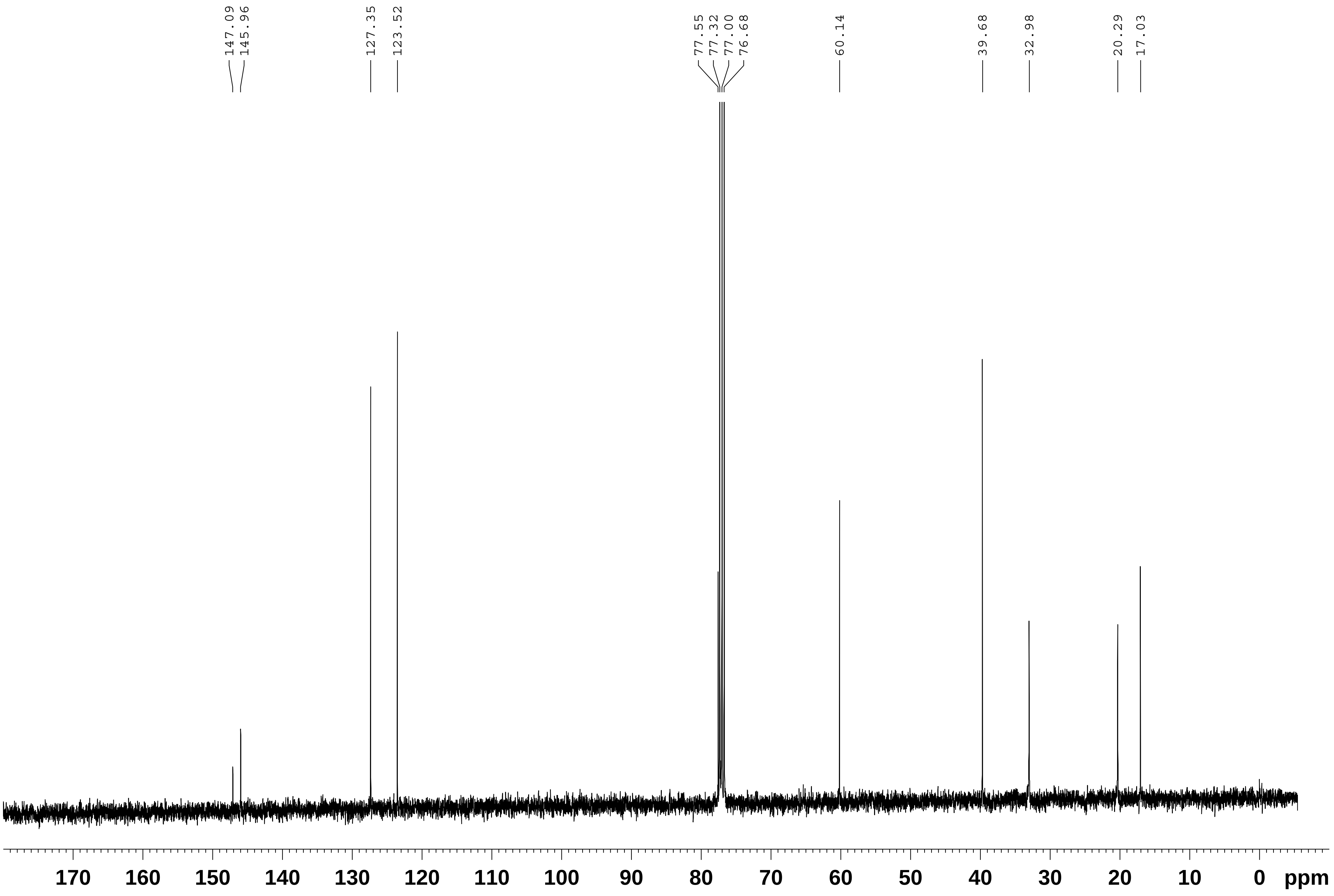

Supplementary Figure 130. The results of cyclic voltammetry experiments

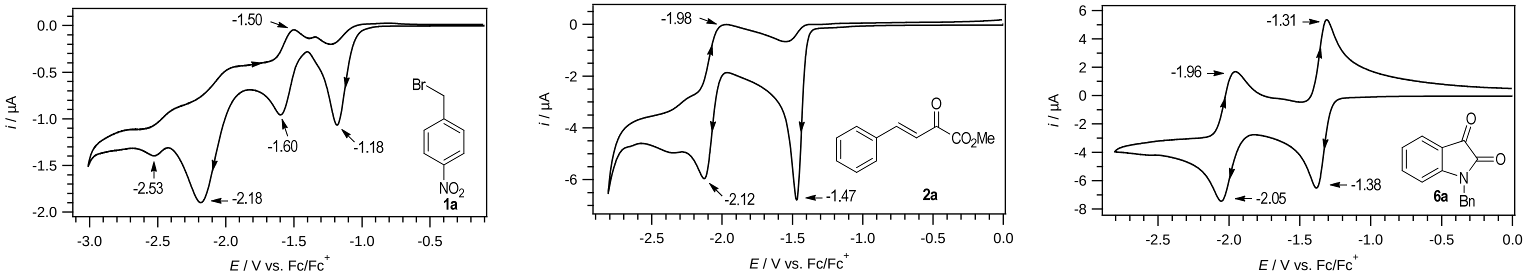

Supplementary Figure 131. ORTEPS drawing of 4w and 7l

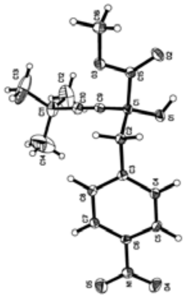

4w (CCDC 1451838)

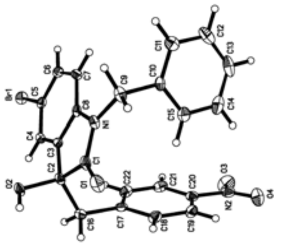

7l (CCDC 1451840)

Supplementary Table 1. Optimization of reaction conditions

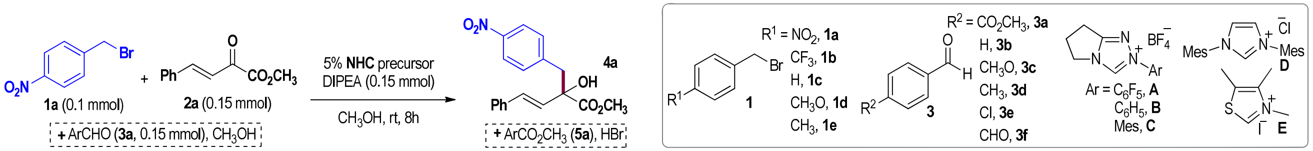

entry	Cat.	**1**	**3**	base	Yield (%)
**1**	**A**	**1a**	**3a**	**DIEA**	**78 (75)** *
2	-	**1a**	**3a**	DIEA	n.r.
3	A	**1a**	**-**	DIEA	n.r.
4	A	**1b**	**3a**	DIEA	n.r.
5	A	**1c**	**3a**	DIEA	n.r.
6	A	**1d**	**3a**	DIEA	n.r.
7	A	**1e**	**3a**	DIEA	n.r.
8	A	**1a**	**3b**	DIEA	36
9	A	**1a**	**3c**	DIEA	27
10	A	**1a**	**3d**	DIEA	34
11	A	**1a**	**3e**	DIEA	61
12	A	**1a**	**3f**	DIEA	60
13	B	**1a**	**3a**	DIEA	35
14	C	**1a**	**3a**	DIEA	29
15	D	**1a**	**3a**	DIEA	23
16	E	**1a**	**3a**	DIEA	n.r.
17	A	**1a**	**3a**	DBU	72
18	A	**1a**	**3a**	Et_3N	45
19	A	**1a**	**3a**	DMAP	45
20	A	**1a**	**3a**	K_2CO_3	50
21	A	**1a**	**3a**	Cs_2CO_3	52

All reactions of **2a** (0.10 mmol, 19.0 mg) with bromide **1** (0.15 mmol) were carried out in presence of catalyst of 5 mol% **A-E**, aldehyde **3** (0.15 eq.) and DIPEA (0.15 mmol, 25 µL) in CH_3OH (1.0 mL) for 8 h. NMR yields. *Isolated yield. DIPEA, N,N-Diisopropylethylamine; Mes, 1,3,5-trimethylbenzene; N.D., Not Detected.

Supplementary Methods

General information

All reactions were carried out under standard conditions using N_2 as shielding gas with magnetic stirring. Analytical thin layer chromatography (TLC) was performed with TLC plates. All reactions and column chromatography were monitored by thin layer chromatography with UV light at 254 nm and colorized with ethanol solution of phosphomolybdic acid, followed by heating using a heat gun. All products could be purified by column chromatography using ethyl acetate and hexane as eluent. Organic solutions were concentrated by rotary evaporation. All solvents were freshly distilled before use. ^1H and ^{13}C NMR chemical shifts are reported in $CDCl_3$ solution of the compound by Bruker AV-400 MHz instruments and marked in ppm relative to tetramethylsilane (TMS) (0) and $CDCl_3$ (77.0 ppm) as standard. The following abbreviations are used to describe peak patterns where appropriate: s = singlet, d = doublet, t = triplet, q = quartet, m = multiplet. Coupling constants (*J*) are reported in Hertz (Hz). High resolution mass spectral analysis (HRMS) was performed on Waters Q-TOF Premier mass spectrometer. X-ray crystallography analysis was performed on Bruker X8 APEX X-ray diffractionmeter.

General procedure for preparation of substrates 1

The substrates **1**[1-3] could be purchased from commercial suppliers or prepared via bromination from the corresponding toluene or alcohol**.** The procedures are as shown below.

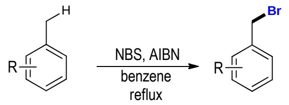

General procedure A[1-2]

The NBS (1.2 equiv) and AIBN (0.1 equiv) were added into the solution of the corresponding 2- or 4-nitro-toluene. The reaction was refluxed until the starting material was fully transferred into product *via* monitoring of TLC. The reaction was poured into cold water. The resulting solution was extracted with ether three times. The organic layer was collected and dried over Na_2SO_4 and concentrated under reduced pressure. The crude product could be purified by column chromatography.

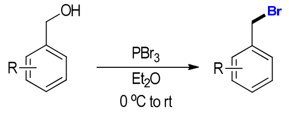

General procedure B[3]

PBr$_3$ (0.5 eq) was added carefully into solution of alcohol (1.0 eq) in Et$_2$O at 0 °C. The resulting mixture was allowed to stir at rt until the starting material was fully transferred into product *via* monitoring of TLC. The reaction mixture was carefully quenched with water and the resulting solution was extracted with ether three times. The organic layer was collected, dried over Na$_2$SO$_4$ and concentrated under reduced pressure. The crude product could be purified by column chromatography.

General procedure for preparation of substrates 2

The ketoester substrates **2** could be prepared according to known literature[4-6] and the procedures are shown below.

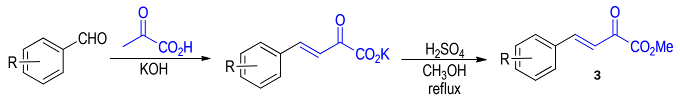

The procedure for alkene ketoesters[4-5]

To a solution of methylpyruvate (1 eq., 15 mmol / mL) and benzaldehyde (1 eq., 15 mmol / mL) in methanol was added at 0 °C a solution of potassium hydroxide (1.5 eq., 5 mmol / mL) in methanol. The first two-thirds of alkali were added dropwise, then the ice-bath was removed and the rest of the potassium hydroxide was run rapidly to complete the condensation before precipitation of potassium pyruvate could occur. The mixture was held at 40 °C for 1h and then at 0°C overnight. The solvent was removed *in vacuo* and the solid was filtered and washed with a little cold methanol to give the potassium salt as a yellow solid.

A saturated solution of potassium salt (1 eq., 0.5 mmol / mL) in water at 40°C was rapidly poured into an excess of 1.6 M HCl. The acid precipitated from water was filtered and dissolved in CH$_2$Cl$_2$, dried (Na$_2$SO$_4$) and the solvent evaporated *in vacuo*. Without further purification, the residue was dissolved in methanol (to 4 mmol / mL) and sulfuric acid (98 %, 0.05 eq.) was added. The mixture was refluxed overnight. The cooled mixture was concentrated *in vacuo*, diluted with CH$_2$Cl$_2$ and washed with saturated brine. The aqueous phase was extracted with CH$_2$Cl$_2$, dried (Na$_2$SO$_4$) and the solvent evaporated in vacuo. The yellow solid was recrystallized from hexane to give the ester product.

The alkyne substrates could be prepared according to known literature[s6] and the procedure is shown below.

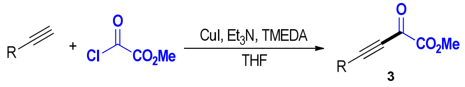

The procedure for alkyne ketoesters[6]

An oven-dried round-bottomed flask tube equipped with a stirrer bar was evacuated while hot and allowed to cool under nitrogen. The flask was first charged with CuI (0.02 eq.), TMEDA (0.05 eq.) and ethyl-2-chloro-2-oxoacetate (1.2 eq.) followed by alkyne (1.0 eq.), Et_3N (3 eq.) and dry THF (conc. 0.2 mmol / mL). The reaction mixture was stirred at room temperature under nitrogen overnight. Saturated $NaHCO_3$ solution (25 mL) was added and the mixture was extracted with EA (3 × 30 mL), dried (Na_2SO_4) and concentrated *in vacuo*. The residue was purified by flash column chromatography.

General procedure for preparation of substrates 6

The isatin-derivate substrates could be prepared according to known literature[7-8] and the procedure is shown below.

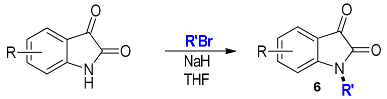

Under a nitrogen atmosphere, NaH (1 eq.) was added portion-wise into a solution of isatin (1 eq., 0.5 mmol / mL) in DMF at 0 °C. After 15 minutes, benzyl bromide (1.4 eq.) was added and the reaction mixture was stirred for 30 minutes at room temperature. Water was added to precipitate the product, which was filtered and washed with hexane to give the protected isatin.

General procedures for the synthetic transformation of products 4b and 7u

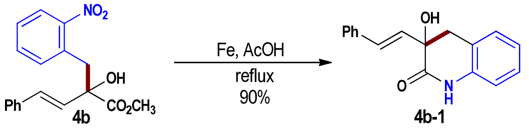

The procedure for synthesis of compound 4b-1:

The solution of **4b** (0.2 mmol, 65 mg, 1.0 eq.) in AcOH was added iron powder (1.2 mmol, 61 mg, 6.0 eq.) and then the reaction system was heated to reflux for 4 h. The solution was cooled to room temperature and neutralized with aq. $NaHCO_3$, the reaction was extracted three times with ethyl acetate. The combined organic extracts were washed with brine, dried over Na_2SO_4, filtered and evaporated under reduced pressure. The product **4b-1** could be obtained in 90% yield through isolation by silica gel column chromatography.

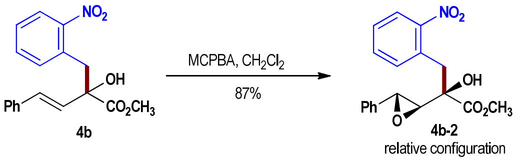

The procedure for synthesis of compound 4b-2:

Under a nitrogen atmosphere, a solution of **4b** (0.2 mmol, 65 mg, 1.0 eq.) in DCM (4.0 mL, 0.05 M) was added to MCPBA (0.4 mmol, 68.4 mg, 2.0 equiv) at 0 °C. After the full conversion of **4b**, the reaction was quenched with saturated solution of $NaHCO_3$. Then the organic phase was separated, and the aqueous phase was extracted with Et_2O three times. The combined organic layers were washed with brine, dried over Na_2SO_4 and concentrated under reduced pressure. The residue was purified by column chromatography to afford product **4b-2** in 87% yield.

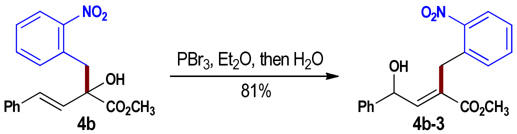

The procedure for synthesis of compound 4b-3:

Under a nitrogen atmosphere, to a stirred solution of compound **43** (0.2 mmol, 65 mg, 1.0 eq.) in Et_2O (1.0 mmol / mL) was added PBr_3 (0.1 mmol, 27 mg, 1.0 eq.) dropwise at 0 °C, then the reaction was allowed to warm to room temperature and stirred. After the reaction was completed, it was cautiously treated with water at 0 °C and then the resulting solution was stirred for 4 h. The organic layer containing the corresponding alcohol compound **4b-3** was separated, washed with brine, dried over anhydrous Na_2SO_4 and concentrated. The residue was purified by column chromatography to afford product **4b-3** in 81% yield.

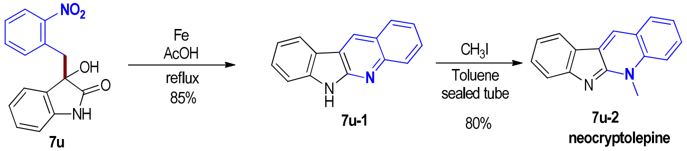

The procedure for synthesis of compound 7u-1:

The solution of **7u** (0.3 mmol, 85 mg, 1.0 eq.) in AcOH was added the iron powder (1.8 mmol, 92 mg, 6.0 eq.) and then the reaction system was heated to reflux for 12 h. The solution was neutralized with an aq. solution of $NaHCO_3$, the reaction was extracted three times with ethyl acetate. The combined organic

solution was washed with brine, dried over Na_2SO_4, filtered and evaporated under the reduce pressure. The product **7u-1** could be obtained in 85% yield through isolation by silica gel column chromatography.

The procedure for total synthesis of neocryptolepine[10]:

Under a nitrogen atmosphere, CH_3I was added into a solution of compound **7u-1** in toluene. The reaction was heated to 130 °C in a sealed tube for 4 h. The solvent was removed under reduce pressure. The nature product neocryptolepine could be obtained in 80% yield through isolation by silica gel column chromatograph.

Experiment of cyclic voltammetry

Cyclic voltammetry (CV) experiments were conducted using a computer-controlled Eco-Chemie Autolab PGSTAT302N potentiostat and a three-electrode setup. Working electrodes were 1-mm diameter planar glassy carbon (GC) disks (eDAQ Pty Ltd), used in conjunction with a Pt wire auxiliary electrode (Metrohm) and an Ag wire miniature reference electrode (eDAQ Pty Ltd) connected to the test solution *via* a salt bridge containing 0.5 M *n*-Bu_4NPF_6 in CH_3CN. Accurate potentials were obtained using ferrocene (Fc) as an internal standard that was added to the solution at the completion of the measurements. All solutions were deoxygenated prior to the experiments by purging with high purity argon gas which was also flowed over the top of the solutions during measurements.

The experiments (Please see Supplementary Figure 130) showed that compound **1a** (E_p^{red} = -1.18 V *vs*) has a higher reduction potential than compounds **2a** (E_p^{red} = -1.47 V *vs*) and **6a** (E_p^{red} = -1.38 V *vs*). The results suggested that most likely **1a** is easier to be reduced, supporting our mechanistic proposal that the SET process first occurs on nitrobenzyl bromide **1a**.

Control experiments

To further examine the reaction pathway, the reactions of nitrobenzene bromide with activated ketones (ketoester or isatin) were investigated in presence of a stronger base (NaOMe). The results showed that the stronger base (NaOMe) could not be promoted the reaction of nitrobenzene with activated ketone in CH_3OH (eq 1 and 2). Subsequently, various solvent (such as THF, Dioxane, toluene, CH_2Cl_2, CH_3CN, DMF) could not also be resulted in the formation of products.

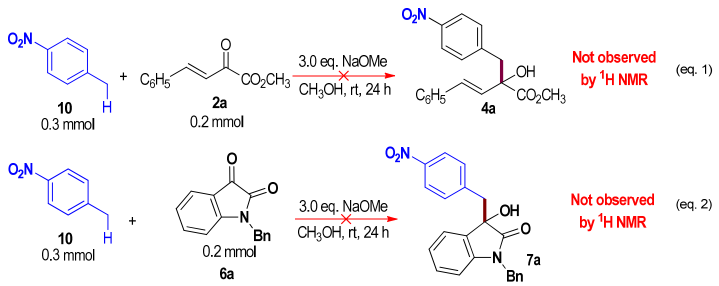

Finally, We mixed para-nitrotoluene and NaOMe using deuterated methanol (CD_3OD) as the solvent, and found that no deuteration of para-nitrotoluene occurred, suggested that the deprotonation of on the CH_3 group of p-nitrotoluene to form an ionic intermediate did not occur in the presence of NaOMe.

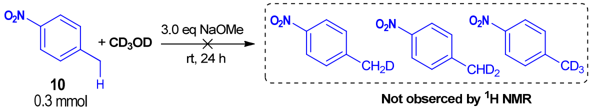

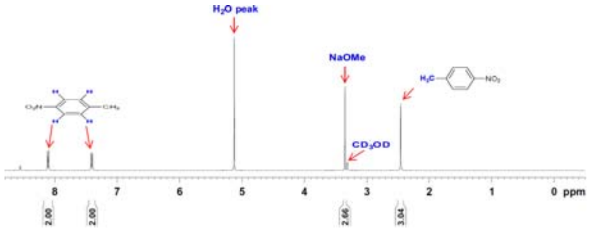

Characterization of compounds

(E)-methyl 2-hydroxy-2-(4-nitrobenzyl)-4-phenylbut-3-enoate

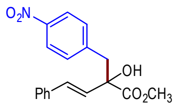

4a, 75%

White solid; m.p. 100–102 °C; ^{1}H NMR (400 MHz, $CDCl_3$): δ 8.13 (d, J = 8.4 Hz, 2H), 7.43-7.38 (m, 4H), 7.35-7.31 (t, J = 8.8 Hz, 2H), 7.29-7.26 (m, 1H), 6.83 (d, J = 15.6 Hz, 1H), 6.38 (d, J = 15.6 Hz, 1H), 3.80 (s, 3H), 3.47 (s, 1H), 3.32 (d, J = 13.2 Hz, 1H), 3.16 (d, J = 13.2 Hz, 1H); ^{13}C NMR (100 MHz, $CDCl_3$): δ 174.2, 147.4, 143.6, 140.8, 131.4, 128.5, 128.3, 125.4, 123.0, 78.6, 53.4, 45.4; IR ν (cm^{-1}) 3495, 1736, 1520, 1346, 1130, 976, 856, 694; HRMS (ESI) calcd. For $C_{18}H_{17}NNaO_5$ $[M+Na]^+$: 350.0999, Found: 350.1003.

(E)-methyl 2-hydroxy-2-(2-nitrobenzyl)-4-phenylbut-3-enoate

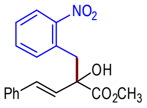

4b, 73%
(1.1 g scale, 70% yield)*

Colourless liquid; ^{1}H NMR (400 MHz, $CDCl_3$): δ 7.79 (s, 1H), 7.50-7.46 (dt, J = 0.8 Hz, 8.0Hz, 1H), 7.43-7.41 (dd, J = 1.2 Hz, 7.6 Hz, 1H), 7.39-7.29 (m, 6H), 6.73 (d, J = 16.0 Hz, 1H), 6.34 (d, J = 15.6 Hz, 1H), 3.78 (s, 3H), 3.62 (s, 2H), 3.48 (s, 1H); ^{13}C NMR (100 MHz, $CDCl_3$): δ 174.4, 151.1, 136.0, 133.4, 132.0, 131.0, 129.7, 128.6, 128.5, 128.0, 128.0, 126.7, 124.5, 77.7, 53.4, 40.3; HRMS (ESI) calcd. For $C_{18}H_{17}NNaO_5$ $[M+Na]^+$: 350.0999, Found: 350.1018.

(E)-methyl 2-(3-chloro-4-nitrobenzyl)-2-hydroxy-4-phenylbut-3-enoate

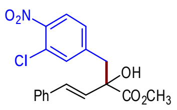

4c, 75%

Colourless liquid; ^{1}H NMR (400 MHz, $CDCl_3$): δ 7.81 (d, J = 8.4 Hz, 1H), 7.46 (d, J = 1.6 Hz, 1H), 7.40-7.38 (m, 2H), 7.36-7.23 (dd, J = 1.2 Hz, 8.0Hz, 2H), 7.32-7.26 (m, 2H), 6.84 (d, J = 15.6 Hz, 1H), 6.36 (d, J = 16.0 Hz, 1H), 3.82 (s, 3H), 3.49 (s, 1H), 3,26 (d, J = 13.6 Hz, 1H), 3.09 (d, J = 13.6 Hz, 1H); ^{13}C NMR (100 MHz, $CDCl_3$): δ 174.0, 146.6, 142.3, 135.8, 133.7, 131.3, 129.4, 128.7, 128.5, 128.2, 126.8, 125.3, 77.6, 53.4, 44.5; HRMS (ESI) calcd. For $C_{18}H_{16}ClNNaO_5$ $[M+Na]^+$: 384.0609, Found: 384.0608.

(E)-methyl 2-(2-cyano-4-nitrobenzyl)-2-hydroxy-4-phenylbut-3-enoate

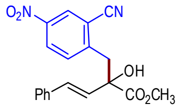

4d, 65%

Colourless liquid; ^{1}H NMR (400 MHz, $CDCl_3$): δ 8.45 (d, J = 2.4 Hz, 1H), 8.34-8.31 (dd, J = 2.4 Hz, 8.4 Hz, 1H), 7.73 (d, J = 8.8 Hz, 1H), 7.39-7.27 (m, 5H), 6.69 (d, J = 15.6 Hz, 1H), 6.44 (d, J = 16.0 Hz, 1H), 3.89 (s, 3H), 3.61 (s, 1H), 3.51 (s, 2H); ^{13}C NMR (100 MHz, $CDCl_3$): δ 174.0, 146.7, 146.2, 135.5, 133.3, 131.8, 128.7, 128.4, 127.5, 127.3, 126.8, 126.5, 116.3, 115.6, 77.3, 53.8, 42.9; HRMS (ESI) calcd. For $C_{19}H_{16}N_2NaO_5$ $[M+Na]^+$: 375.0951, Found: 375.0959.

(E)-methyl 2-(4-cyano-2-nitrobenzyl)-2-hydroxy-4-phenylbut-3-enoate

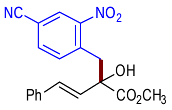

4e, 71%

White solid; m.p. 131–133 °C; ^{1}H NMR (400 MHz, $CDCl_3$): δ 8.08 (d, J = 1.6 Hz, 1H), 7.75-7.23 (dd, J = 1.6 Hz, 8.0 Hz, 1H), 7.58 (d, J = 8.0 Hz, 1H), 7.33-7.32 (m, 5H), 6.68 (d, J = 15.6 Hz, 1H), 6.28 (d, J = 15.6 Hz, 1H), 3.81 (s, 3H), 3.67 (d, J = 2.4 Hz, 2H) 3.49 (d, J = 13.2 Hz, 1H); ^{13}C NMR (100 MHz, $CDCl_3$): δ 173.9, 151.1, 135.5, 135.2, 134.8, 134.6, 131.7, 128.7, 128.3, 128.0, 127.6, 126.8, 116.5, 112.4, 77.4, 53.7, 40.2; IR ν (cm^{-1}) 3502, 2237, 1732, 1539, 1361, 1215, 972, 748; HRMS (ESI) calcd. For $C_{19}H_{17}N_2O_5$ $[M+H]^+$: 353.1132, Found: 353.1131.

(E)-methyl 4-(2-hydroxy-2-(methoxycarbonyl)-4-phenylbut-3-en-1-yl)-3-nitrobenzoate

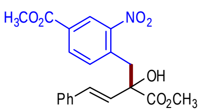

4f, 70%

Colourless liquid; ^{1}H NMR (400 MHz, $CDCl_3$): δ 8.43 (d, *J* = 1.6 Hz, 1H), 8.13-8.10 (dd, *J* = 2.0 Hz, 8.0 Hz, 1H), 7.52 (d, *J* = 8.0 Hz, 1H), 7.35-7.25 (m, 5H), 6.72 (d, *J* = 15.6 Hz, 1H), 6.32 (d, *J* = 16.0 Hz, 1H), 3.94 (s, 3H), 3.79 (s, 3H), 3.66 (d, *J* = 2.0 Hz, 2H), 3,50 (s, 1H); ^{13}C NMR (100 MHz, $CDCl_3$): δ 174.1, 164.9, 151.0, 135.8, 134.5, 133.8, 132.4, 131.4, 130.3, 128.6, 128.2, 128.1, 126.8, 125.5, 77.6, 53.5, 52.6, 40.3; HRMS (ESI) calcd. For $C_{20}H_{19}NNaO_7$ $[M+Na]^+$: 408.1054, Found: 408.1054.

(E)-methyl 2-(4-acetyl-2-nitrobenzyl)-2-hydroxy-4-phenylbut-3-enoate

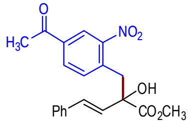

4g, 61%

White solid; m.p. 108–110 °C; ^{1}H NMR (400 MHz, $CDCl_3$): δ 8.34 (d, *J* = 2.0 Hz, 1H), 8.05-8.02 (dd, *J* = 2.0 Hz, 8.0 Hz, 1H), 7.55 (d, *J* = 8.0 Hz, 1H), 7.36-7.29 (m, 4H), 7.28-7.23 (m, 1H), 6.71 (d, *J* = 16.0 Hz, 1H), 6.32 (d, *J* = 16.0 Hz, 1H), 3.80 (s, 3H), 3.71-3.63 (dd, *J* = 9.6 Hz, 13.2 Hz, 2H), 3.51 (s, 1H), 2.62 (s, 3H); ^{13}C NMR (100 MHz, $CDCl_3$): δ 195.5, 174.1, 151.2, 136.7, 135.7, 134.6, 134.0, 131.4, 130.9, 128.6, 128.2, 128.0, 126.8, 124.3, 77.6, 53.6, 40.3, 26.6; IR ν (cm^{-1}) 3507, 1732, 1536, 1362, 1257, 1165, 748, 694; HRMS (ESI) calcd. For $C_{20}H_{19}NNaO_6$ $[M+Na]^+$: 392.1105, Found: 392.1114.

(E)-methyl 2-hydroxy-2-(4-(hydroxymethyl)-2-nitrobenzyl)-4-phenylbut-3-enoate

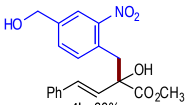

4h, 60%

White solid; m.p. 106–108 °C; ^{1}H NMR (400 MHz, $CDCl_3$): δ 7.80 (s, 1H), 7.48 (d, *J* = 1.2 Hz, 1H), 7.46 (d, *J* = 1.2 Hz, 1H), 7.42-7.23 (m, 6H), 6.73 (d, *J* = 15.6 Hz, 1H), 6.36 (d, *J* = 1.5.6 Hz, 1H), 4.73 (s, 2H), 3.78 (s, 3H), 3.60 (s, 2H), 3.49 (s, 1H), 1.90 (s, 1H); ^{13}C NMR (100 MHz, $CDCl_3$): δ 174.4, 151.1, 141.4, 136.0, 133.6, 131.0, 129.9, 128.7, 128.6, 128.5, 128.0, 126.8, 122.5, 77.7, 63.7, 53.5, 40.1; IR ν (cm^{-1}) 3256, 1748, 1535, 1362, 1250, 1165, 1049, 694; HRMS (ESI) calcd. For $C_{19}H_{19}NNaO_6$ $[M+Na]^+$: 380.1105, Found: 380.1111.

(E)-methyl 2-(5-chloro-2-nitrobenzyl)-2-hydroxy-4-phenylbut-3-enoate

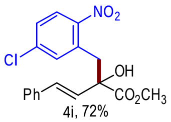

4i, 72%

White solid; m.p. 215–217 °C; ^{1}H NMR (400 MHz, $CDCl_3$): δ 7.76 (d, *J* = 8.4 Hz, 1H), 7.44 (d, *J* = 6.0 Hz, 1H), 7.37-7.30 (m, 5H), 7.27-7.24 (m, 1H), 6.74 (d, *J* = 16.0 Hz, 1H), 6.31 (d, *J* = 16.0 Hz, 1H), 3.80 (s, 3H), 3.64 (d, *J* = 12.0 Hz, 1H), 3.56 (d, *J* = 11.6 Hz, 1H) 3.49 (s, 1H); ^{13}C NMR (100 MHz, $CDCl_3$): δ 174.2, 149.3, 138.2, 135.8, 133.4, 131.9, 131.3, 128.6, 128.1, 128.1, 126.8, 125.9, 77.6, 53.6, 40.1; IR ν (cm^{-1}) 3480, 1717, 1516, 1350, 1258, 1138, 976, 853; HRMS (ESI) calcd. For $C_{18}H_{16}ClNNaO_5$ $[M+Na]^+$: 384.0609, Found: 384.0616.

(E)-methyl 2-(4,5-dimethoxy-2-nitrobenzyl)-2-hydroxy-4-phenylbut-3-enoate

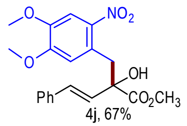

4j, 67%

White solid; m.p. 116–118 °C; ^{1}H NMR (400 MHz, $CDCl_3$): δ 7.45 (s, 1H), 7.37-7.29 (m, 4H), 7.26-7.23 (m, 1H), 6.87 (s, 1H), 6.73 (d, *J* = 15.6 Hz, 1H), 6.38 (d, *J* = 16.0 Hz, 1H), 3.91 (s, 3H), 3.87 (s, 3H), 3.79 (s, 3H), 3.69 (d, *J* = 14.0 Hz, 1H), 3.58 (d, *J* = 14.0 Hz, 1H), 3.57 (s, 1H); ^{13}C NMR (100 MHz, $CDCl_3$): δ 174.6, 151.8, 147.7, 143.1, 136.0, 130.7, 128.7, 128.6, 128.0, 126.7, 124.4， 115.0, 108.0, 77.8, 56.2, 56.2, 53.4, 40.5; IR ν (cm^{-1}) 3445, 1740, 1524, 1300, 1223, 1061, 769, 532; HRMS (ESI) calcd. For $C_{20}H_{21}NNaO_7$ [M+Na]$^+$: 410.1210, Found: 410.1199.

(E)-methyl 2-hydroxy-2-((4-nitrophenyl)(phenyl)methyl)-4-phenylbut-3-enoate

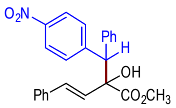

4k, 68%

White solid; ^{1}H NMR (400 MHz, $CDCl_3$): δ 8.14-8.00 (m, 6H), 7.73-7.63 (m, 4H), 7.48-7.46 (m, 2H), 7.39-7.09 (m, 16H), 6.82-6.73 (m, 2H), 6.29-6.25 (m, 2H), 4.91 (s, 1H), 4.70 (s, 1H), 4.69 (s, 1H), 3.85 (s, 1H), 3.72 (s, 6H); ^{13}C NMR (100 MHz, $CDCl_3$): δ 174.4, 174.2, 150.2, 147.8, 147.1, 140.8, 138.5, 137.8, 136.1, 135.9, 132.2, 132.0, 130.9, 130.2, 130.0, 129.2, 129.1, 128.8, 128.7, 128.6, 128.6, 128.5, 128.5, 128.3, 128.3, 128.1, 128.0, 127.6, 127.2, 126.9, 126.7, 126.7, 123.8, 123.4, 123.2, 80.3, 57.7, 57.6, 55.9, 53.5, 53.4; IR ν (cm^{-1}) 3483, 1714, 1515, 1356, 1257, 1134, 976, 853; HRMS (ESI) calcd. For $C_{24}H_{21}NNaO_5$ [M+Na]$^+$: 425.1234, Found: 425.1238.

(E)-methyl 2-hydroxy-2-(2-(4-nitrophenyl)propan-2-yl)-4-phenylbut-3-enoate

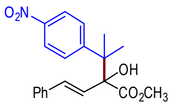

4l, 51%

White solid; m.p. 167–169 °C; ^{1}H NMR (400 MHz, $CDCl_3$): δ 8.12 (d, *J* = 9.2 Hz, 2H), 7.58 (d, *J* = 8.8 Hz, 2H), 7.39 (d, *J* = 7.2 Hz, 2H), 7.34 (t, *J* = 7.2 Hz, 2H), 7.28-7.25 (m, 1H), 6.82 (d, *J* = 16.0 Hz, 1H), 6.48 (d, *J* = 15.6 Hz, 1H), 3.70 (s, 3H), 3.49 (s, 1H), 1.61 (s, 3H), 1.50 (s, 3H); ^{13}C NMR (100 MHz, $CDCl_3$): δ 174.2， 152.4， 146.5， 136.3， 132.2， 128.7， 128.7， 128.0， 126.7， 126.1， 122.4， 81.7， 53.1， 46.6， 24.6， 23.9； IR ν (cm^{-1}) 3480, 2959, 1717, 1516, 1350, 1258, 1138, 853; HRMS (ESI) calcd. For $C_{20}H_{21}NNaO_5$ [M+Na]$^+$: 378.1312, Found: 378.1307.

(E)-methyl 4-(2-fluorophenyl)-2-hydroxy-2-(4-nitrobenzyl)but-3-enoate

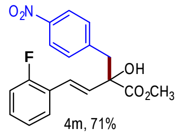

4m, 71%

White solid; m.p. 123–125 °C; ^{1}H NMR (400 MHz, $CDCl_3$): δ 8.13 (d, *J* = 8.4 Hz, 2H), 7.43-7.39 (m, 3H), 7.24-7.21 (m, 1H), 7.11 (t, ^{J} = 7.6 Hz, 1H), 7.07-7.02 (m, 1H), 6.95 (d, *J* = 15.6 Hz, 1H), 6.51 (d, *J* = 16.0 Hz, 1H), 3.81 (s, 3H), 3.49 (s, 1H), 3,32 (d, *J* = 13.2 Hz, 1H), 3.16 (d, *J* = 13.2 Hz, 1H); ^{13}C NMR (100 MHz, $CDCl_3$): δ 174.1, 161.8, 159.3, 147.2, 143.2, 131.5 (d, *J* = 22.6 Hz), 131.2, 129.4 (d, *J* = 33.8 Hz), 128.2 (d, *J* = 11.3 Hz), 124.1 (t, *J* = 15.0 Hz), 124.1, 123.7 (d, *J* = 54.1 Hz), 123.2, 115.9 (d, *J* = 82.7 Hz), 77.8, 53.4, 45.0; IR ν

(cm^{-1}) 3510, 1747, 1740, 1520, 1346, 1112; HRMS (ESI) calcd. For $C_{18}H_{16}FNNaO_5$ [M+Na]$^+$: 368.0905, Found: 368.0909.

(E)-methyl 2-hydroxy-4-(2-methoxyphenyl)-2-(4-nitrobenzyl)but-3-enoate

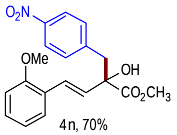

4n, 70%

Colourless liquid; ^{1}H NMR (400 MHz, $CDCl_3$): δ 8.15-8.11 (dt, *J* = 2.4 Hz, 9.6 Hz, 2H) 7.44-7.40 (m, 3H), 7.26-7.323 (m, 1H), 7.12 (d, *J* = 16.0 Hz, 1H), 6.93 (t, *J* = 7.6 Hz, 1H), 6.87 (d, *J* = 16.0 Hz, 1H), 6.42 (d, *J* = 16.0 Hz, 1H), 3.84 (s, 3H), 3.79 (s, 3H), 3.44 (s, 1H), 3,32 (d, *J* = 13.6 Hz, 1H), 3.17 (d, *J* = 13.6 Hz, 1H); ^{13}C NMR (100 MHz, $CDCl_3$): δ 174.3, 157.0, 147.1, 143.5, 131.2, 129.3, 129.2, 127.2, 126.1, 124.8, 123.1, 120.6, 111.0, 78.0, 55.4, 53.2, 45.1； IR ν (cm^{-1}) 3495, 1736, 1520, 1346, 1246, 1103, 976, 745; HRMS (ESI) calcd. For $C_{19}H_{19}NNaO_6$ [M+Na]$^+$: 380.1105, Found: 380.1106.

(E)-methyl 4-(3-fluorophenyl)-2-hydroxy-2-(4-nitrobenzyl)but-3-enoate

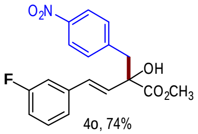

4o, 74%

White solid; m.p. 89–91 °C; ^{1}H NMR (400 MHz, $CDCl_3$): δ 8.13 (d, *J* = 7.6 Hz, 2H), 7.41 (d, *J* = 8.4 Hz, 2H), 7.32-7.28 (m, 1H), 7.13 (d, *J* = 8.0 Hz, 1H), 7.11-7.08 (dd, *J* = 2.0 Hz, 10.0 Hz, 1H), 6.99-6.94 (dt, *J* = 1.6 Hz, 10.8 Hz, 1H), 6.80 (d, *J* = 15.6 Hz, 1H), 6.40 (d, *J* = 15.6 Hz, 1H), 3.81 (s, 3H), 3.49 (s, 1H), 3,32 (d, *J* = 14.4 Hz, 1H), 3.15 (d, *J* = 14.4 Hz, 1H); ^{13}C NMR (100 MHz, $CDCl_3$): δ 174.0, 164.30, 161.9, 147.2, 143.1, 138.2 (d, *J* = 30.1 Hz), 131.2, 130.2, 130.1 (d, *J* = 7.5 Hz), 123.2, 122.8, 122.8, 114.9 (d, *J* = 79.0 Hz), 113.1 (d, *J* = 82.7 Hz), 77.6, 53.4, 45.1; IR ν (cm^{-1}) 3503, 3075, 1740, 1520, 1346, 1130, 961, 698; HRMS (ESI) calcd. For $C_{18}H_{16}FNNaO_5$ [M+Na]$^+$: 368.0905, Found: 368.0899.

(E)-methyl 2-hydroxy-4-(3-methoxyphenyl)-2-(4-nitrobenzyl)but-3-enoate

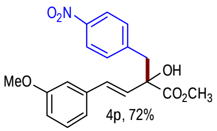

4p, 72%

Colourless liquid; ^{1}H NMR (400 MHz, $CDCl_3$): δ 8.13 (d, *J* = 8.4 Hz, 2H), 7.41 (d, *J* = 8.8 Hz, 2H), 7.27-7.23 (m, 1H), 6.98 (d, *J* = 7.6 Hz, 1H), 6.91 (s, 1H), 6.84 (d, *J* = 2.4 Hz, 1H), 6.79 (d, *J* = 16.0 Hz, 1H), 6.37 (d, *J* = 15.6 Hz, 1H), 3.82 (s, 3H), 3.80 (s, 3H), 3.45 (s, 1H), 3,31 (d, *J* = 13.6 Hz, 1H), 3.15 (d, *J* = 13.6 Hz, 1H); ^{13}C NMR (100 MHz, $CDCl_3$): δ 174.2, 159.9, 147.2, 143.3, 137.3, 131.2, 131.0, 129.7, 129.1, 123.2, 119.4, 113.7, 112.3, 77.7, 55.3, 53.4, 45.1; IR ν (cm^{-1}) 3507, 2920, 1736, 1520, 1246, 1026, 860, 698; HRMS (ESI) calcd. For $C_{19}H_{19}NNaO_6$ [M+Na]$^+$: 380.1105, Found: 380.1094.

(E)-methyl 4-(4-fluorophenyl)-2-hydroxy-2-(4-nitrobenzyl)but-3-enoate

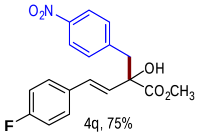

4q, 75%

White solid; m.p. 135–137 °C; ^{1}H NMR (400 MHz, $CDCl_3$): δ 8.13 (d, *J* = 8.0 Hz, 2H), 7.41 (d, *J* = 8.4 Hz, 2H), 7.36 (t, *J* = 6.4 Hz, 2H),

7.04-7.00 (m, 2H), 6.80 (d, *J* = 15.6 Hz, 1H), 6.31 (d, *J* = 15.6 Hz, 1H), 3.81 (s, 3H), 3.47 (s, 1H), 3,31 (d, *J* = 13.6 Hz, 1H), 3.14 (d, *J* = 13.2 Hz, 1H); ^{13}C NMR (100 MHz, $CDCl_3$): δ 174.1, 147.2, 143.2, 132.1 (d, *J* = 11.3 Hz), 131.2, 129.9, 128.4 (d, *J* = 30.1 Hz), 123.2, 115.6 (d, *J* = 79.0 Hz), 77.6, 53.4, 45.1; IR ν (cm^{-1}) 3499, 1754, 1520, 1346, 1215, 1130, 856, 694; HRMS (ESI) calcd. For $C_{18}H_{16}FNNaO_5$ $[M+Na]^+$: 368.0905, Found: 368.0921.

(E)-methyl 2-hydroxy-4-(4-methoxyphenyl)-2-(4-nitrobenzyl)but-3-enoate

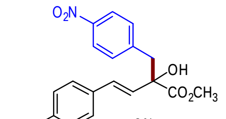

4r, 72%

White solid; m.p. 114–116 °C; ^{1}H NMR (400 MHz, $CDCl_3$): δ 8.13 (d, *J* = 8.4 Hz, 2H), 7.41 (d, *J* = 8.4 Hz, 2H), 7.32 (d, *J* = 8.4 Hz, 2H), 6.86 (d, *J* = 8.4 Hz, 2H), 6.76 (d, *J* = 16.4 Hz, 1H), 6.24 (d, *J* = 16.0 Hz, 1H), 3.81 (s, 3H), 3.79 (s, 3H), 3.44 (s, 1H), 3,31 (d, *J* = 13.6 Hz, 1H), 3.14 (d, *J* = 13.2 Hz, 1H); ^{13}C NMR (100 MHz, $CDCl_3$): δ 174.3, 159.7, 147.1, 143.4, 131.2, 130.4, 128.0, 126.5, 123.2, 114.1, 77.7, 55.3, 53.3, 45.1; IR ν (cm^{-1}) 3487, 1724, 1528, 1126, 1042, 980, 752, 690; HRMS (ESI) calcd. For $C_{19}H_{19}NNaO_6$ $[M+Na]^+$: 380.1105, Found: 380.1097.

(E)-methyl 2-hydroxy-2-(4-nitrobenzyl)-4-(thiophen-2-yl)but-3-enoate

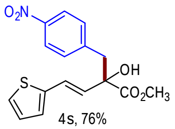

4s, 76%

White solid; m.p. 98–100 °C; ^{1}H NMR (400 MHz, $CDCl_3$): δ 8.13 (d, *J* = 8.4 Hz, 2H), 7.40 (d, *J* = 8.4 Hz, 2H), 7.20 (d, *J* = 4.8 Hz, 1H), 6.98-6.97 (m, 3H), 6.22 (d, *J* = 15.6 Hz, 1H), 3.80 (s, 3H), 3.47 (s, 1H), 3.29 (d, *J* = 13.2 Hz, 1H), 3,14 (d, *J* = 13.6 Hz, 1H); ^{13}C NMR (100 MHz, $CDCl_3$): δ 174.0, 147.2, 143.2, 140.9, 131.2, 128.0, 127.6, 126.9, 125.0, 124.4, 123.2, 77.4, 53.4, 45.1; IR ν (cm^{-1}) 3375, 2237, 1706, 1529, 1192, 1115, 856, 698; HRMS (ESI) calcd. For $C_{16}H_{15}NNaO_5S$ $[M+Na]^+$: 356.0563, Found: 356.0569.

(3E,5E)-methyl 2-hydroxy-2-(4-nitrobenzyl)-6-phenylhexa-3,5-dienoate

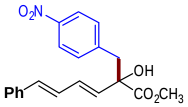

4t, 67%

White solid; m.p. 141–143 °C; ^{1}H NMR (400 MHz, $CDCl_3$): δ 8.14 (d, *J* = 8.4 Hz, 2H), 7.41-7.39 (m, 4H), 7.34-7.30 (m, 2H), 7.24-7.22 (m, 1H), 6.81-6.75 (m, 1H), 6.65-6.58 (m, 2H), 5.98 (d, *J* = 14.8 Hz, 1H), 3.79 (s, 3H), 3.41 (s, 1H), 3,27 (d, *J* = 13.6 Hz, 1H), 3.11 (d, *J* = 13.6 Hz, 1H); ^{13}C NMR (100 MHz, $CDCl_3$): δ 174.1, 147.2, 143.3, 136.9, 134.4, 132.4, 131.4, 131.2, 128.7, 127.9, 127.2, 126.5, 123.2, 77.6, 53.3, 45.0; IR ν (cm^{-1}) 3487, 2920, 1735, 1520, 1269, 1130, 991, 694; HRMS (ESI) calcd. For $C_{20}H_{19}NNaO_5$ $[M+Na]^+$: 376.1155, Found: 376.1157.

(E)-methyl 2-hydroxy-2-(4-nitrobenzyl)-6-phenylhex-3-enoate

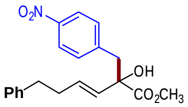

4u, 66%

Colourless liquid; ^{1}H NMR (400 MHz, $CDCl_3$): δ 8.11 (d, *J* = 8.8 Hz, 2H), 7.34 (d, *J* = 8.8 Hz, 2H), 7.29 (d, *J* = 7.6 Hz, 2H), 7.20-7.13 (m,

3H), 5.97-5.90 (m, 1H), 5.63 (d, *J* = 15.6 Hz, 1H), 3.74 (s, 3H), 3.26 (s, 1H), 3.16 (d, *J* = 13.2 Hz, 1H), 3.01 (d, *J* = 13.6 Hz, 1H), 2.69 (t, *J* = 7.6 Hz, 2H), 2.41-2.35 (dd, *J* = 7.6 Hz, 15.2 Hz, 2H); ^{13}C NMR (100 MHz, $CDCl_3$): δ 174.5，147.1，143.7，141.4，131.6，131.2，130.3，128.5，128.3，125.9，123.1，77.3, 53.1, 44.8，35.3，33.7；IR ν (cm^{-1}) 3483, 1736, 1520, 1346, 1207, 1111, 976, 689; HRMS (ESI) calcd. For $C_{20}H_{21}NNaO_5$ $[M+Na]^+$: 378.1312, Found: 378.1318.

methyl 2-hydroxy-3-(4-nitrophenyl)-2-phenylpropanoate

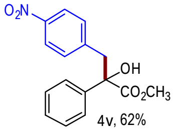

4v, 62%

White solid; m.p. 99–101 °C; ^{1}H NMR (400 MHz, $CDCl_3$): δ 8.09 (d, *J* = 8.4 Hz, 2H), 7.61 (d, *J* = 7.2 Hz, 2H), 7.40-7.33 (m, 5H), 3.78 (s, 3H), 3.69 (s, 1H), 3.60 (d, *J* = 13.6 Hz, 1H), 3,36 (d, *J* = 13.6 Hz, 1H); ^{13}C NMR (100 MHz, $CDCl_3$) 174.2, 147.4, 143.6, 140.8, 131.4, 128.5, 128.3, 125.4, 123.0, 78.6, 53.4, 45.4; IR ν (cm^{-1}) 3507, 2920, 1736, 1520, 1346, 1261, 1111, 698; HRMS (ESI) calcd. For $C_{16}H_{15}NNaO_5$ $[M+Na]^+$: 324.0842, Found: 324.0837.

methyl 2-hydroxy-5,5-dimethyl-2-(4-nitrobenzyl)hex-3-ynoate

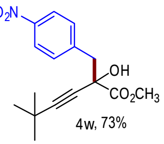

4w, 73%

White solid; m.p. 110–112 °C; ^{1}H NMR (400 MHz, $CDCl_3$): δ 8.15 (d, *J* = 8.4 Hz, 2H), 7.47 (d, *J* = 8.4 Hz, 2H), 3.85 (s, 3H), 3.36 (s, 1H), 3,32 (d, *J* = 13.2 Hz, 1H), 3.16 (d, *J* = 13.6 Hz, 1H), 1.18 (s, 9H); ^{13}C NMR (100 MHz, $CDCl_3$): δ 172.3, 147.3, 142.9, 131.6, 123.0, 95.7, 76.4, 70.9, 53.7, 45.5, 30.6, 27.4; HRMS (ESI) calcd. For $C_{16}H_{19}NNaO_5$ $[M+Na]^+$: 328.1155, Found: 328.1161.

methyl 4-cyclohexyl-2-hydroxy-2-(4-nitrobenzyl)but-3-ynoate

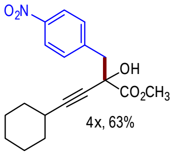

4x, 63%

White solid; m.p. 135–137 °C; ^{1}H NMR (400 MHz, $CDCl_3$): δ 8.17 (dt, *J* = 2.0 Hz, 8.8 Hz, 2H), 7.48-7.45 (dd, *J* = 2.0 Hz, 8.8 Hz, 2H), 3.85 (s, 3H), 3.38 (s, 1H), 3.33 (d, *J* = 13.6 Hz, 1H), 3.25 (d, *J* = 13.6 Hz, 1H), 2.42-2.35 (m, 1H), 1.76-1.73 (m, 2H), 1.65-1.62 (m, 3H), 1.53-1.40 (m, 2H), 1.38-1.19 (m, 3H); δ 172.3, 147.3, 142.8, 131.6, 123.0, 91.7, 77.8, 70.9, 53.7, 45.5, 32.1, 28.9, 25.7, 24.7; IR ν (cm^{-1}) 3507, 2928, 1728, 1508, 1350, 1076, 856, 590; HRMS (ESI) calcd. For $C_{18}H_{21}NNaO_5$ $[M+Na]^+$: 354.1312, Found: 354.1309.

methyl 4-(cyclohex-1-en-1-yl)-2-hydroxy-2-(4-nitrobenzyl)but-3-ynoate

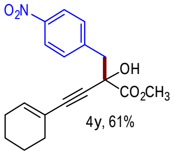

4y, 61%

Colourless liquid; ^{1}H NMR (400 MHz, $CDCl_3$): δ 8.15 (d, *J* = 8.4 Hz, 2H), 7.47 (d, *J* = 8.4 Hz, 2H), 6.13 (t, *J* = 1.6 Hz, 1H), 3.85 (s, 3H), 3.45 (s, 1H), 3,37 (d, *J* = 13.2 Hz, 1H), 3.28 (d, *J* = 13.6 Hz, 1H), 2.11-2.05 (m, 4H), 1.65-1.54 (m, 4H); ^{13}C NMR (100 MHz, $CDCl_3$): δ 172.0, 147.3, 142.7, 137.0, 131.6, 123.1, 119.4, 88.3, 83.7, 71.2, 53.8, 45.4, 28.7, 25.6, 22.1, 21.3; IR ν (cm^{-1}) 3499, 2932, 2218, 1744, 1261,

1219, 1084, 856; HRMS (ESI) calcd. For $C_{18}H_{19}NNaO_5$ $[M+Na]^+$: 352.1155, Found: 352.1168.

methyl 2-hydroxy-2-(2-(4-nitrophenyl)propan-2-yl)-4-phenylbut-3-ynoate

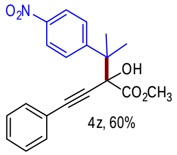

4z, 60%

White solid; m.p. 130–132 °C; ^{1}H NMR (400 MHz, $CDCl_3$): δ 8.15 (d, *J* = 8.8 Hz, 2H), 7.70 (d, *J* = 8.4 Hz, 2H), 7.46-7.44 (m, 2H), 7.36-7.32 (m, 3H), 3.78 (s, 3H), 3.75 (s, 1H), 1.70 (s, 6H); ^{13}C NMR (100 MHz, $CDCl_3$): δ 171.9, 151.2, 146.8, 131.8, 129.0, 128.9, 128.4, 122.5, 121.8, 86.7, 85.8, 77.6, 53.7, 46.8, 24.7, 24.4; IR ν (cm^{-1}) 3476, 1721, 1512, 1350, 1273, 1088, 856, 702; HRMS (ESI) calcd. For $C_{20}H_{19}NNaO_5$ $[M+Na]^+$: 376.1155, Found: 376.1168.

1-benzyl-3-hydroxy-3-(4-nitrobenzyl)indolin-2-one

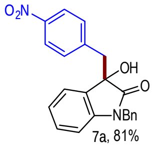

7a, 81%

White solid; m.p. 163–165 °C; ^{1}H (400 MHz, $CDCl_3$) δ 7.87 (d, *J* = 8.8 Hz, 2H), 7.32 (dd, *J* = 7.2 Hz, 0.4 Hz, 1H), 7.23-7.03 (m, 5H), 7.04 (d, *J* = 8.8 Hz, 2H), 6.84 (d, *J* = 7.2 Hz, 2H), 6.58 (d, *J* = 7.6 Hz, 1H), 4.92 (d, *J* = 16.0 Hz, 1H), 4.47 (d, *J* = 16.0 Hz, 1H), 4.27 (s, 1H) 3.50-3.39 (dd, *J* = 32.4 Hz, 12.8 Hz, 2H); ^{13}C NMR (100 MHz, $CDCl_3$): δ 177.4, 147.0, 142.4, 141.6, 134.7, 131.1, 130.2, 128.7, 128.6, 127.8, 127.0, 124.3, 123.3, 123.0, 109.7, 77.2, 44.4, 43.8; IR ν (cm^{-1}) 3318, 1701, 1612, 1346, 1296, 1111, 853, 760; HRMS (ESI) calcd. For $C_{22}H_{18}N_2NaO_4$ $[M+Na]^+$: 397.1159, Found: 397.1168.

1-benzyl-3-(3-chloro-4-nitrobenzyl)-3-hydroxyindolin-2-one

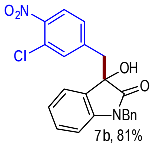

7b, 81%

White solid; m.p. 156–158 °C; ^{1}H (400 MHz, $CDCl_3$) δ 7.57 (d, *J* = 7.6 Hz, 1H), 7.33 (d, *J* = 8.8 Hz, 1H), 7.25-7.21 (m, 4H), 7.14-7.10 (m, 2H), 6.94-6.90 (m, 3H), 6.63 (d, *J* = 7.6 Hz, 1H), 4.97 (d, *J* = 15.6 Hz, 1H), 4.51 (d, *J* = 15.6 Hz, 1H), 4.09 (s, 1H), 3.42 (d, *J* = 12.8 Hz, 1H), 3.31 (d, *J* = 12.8 Hz, 1H); ^{13}C NMR (100 MHz, $CDCl_3$): δ 177.2, 146.5, 142.4, 140.7, 134.7, 133.6, 130.4, 129.4, 128.8, 128.3, 127.9, 126.8, 126.6, 125.2, 124.3, 123.4, 109.9, 76.9, 44.0, 43.8; IR ν (cm^{-1}) 3379, 1709, 1524, 1470, 1339, 1184, 883, 760; HRMS (ESI) calcd. For $C_{22}H_{17}ClN_2NaO_4$ $[M+Na]^+$: 431.0769, Found: 431.0765.

2-((1-benzyl-3-hydroxy-2-oxoindolin-3-yl)methyl)-5-nitrobenzonitrile

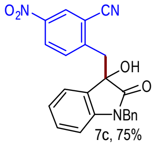

7c, 75%

White solid; m.p. 159–161 °C; ^{1}H (400 MHz, $CDCl_3$) δ 8.34 (d, *J* = 2.4 Hz, 1H), 8.14-8.11 (dd, *J* = 8.4 Hz, 2.4 Hz, 1H), 7.55 (d, *J* = 8.8 Hz, 1H), 7.56-7.24 (m, 4H), 7.19-7.17 (m, 3H), 7.09-7.05 (m, 1H), 6.74 (d, *J* = 8.0 Hz, 1H), 4.93 (d, *J* = 15.6, 1H), 4.72 (d, *J* = 15.6 Hz, 1H), 3.65 (d, *J* = 13.6 Hz, 1H), 3.50 (d, *J* = 13.2 Hz, 1H), 3.38 (s, 1H); ^{13}C NMR (100 MHz, $CDCl_3$): δ 176.7, 146.7, 144.9, 142.0, 134.9, 132.8, 130.6, 128.8,

128.0, 127.9, 127.5, 127.4, 126.5, 124.8, 123.5, 115.9, 115.8, 109.8, 76.2, 44.1, 42.5; IR ν (cm^{-1}) 3437, 2236, 1701, 1612, 1350, 1281, 1080, 745; HRMS (ESI) calcd. For $C_{23}H_{18}N_3O_4$ $[M+H]^+$: 400.1292, Found: 400.1293.

1-benzyl-3-hydroxy-3-(2-nitrobenzyl)indolin-2-one

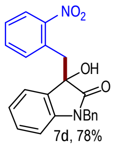

7d, 78%

White solid; m.p. 193–195 °C; ^1H (400 MHz, $CDCl_3$) δ 7.84 (d, *J* = 8.0 Hz, 2H), 7.48-7.37 (m, 3H), 7.29-7.24 (m, 3H), 7.19-7.15 (m, 1H), 7.12 (d, *J* = 1.2 Hz, 2H), 7.03-6.96 (m, 2H), 6.62 (d, *J* = 7.6 Hz, 1H), 4.92 (d, *J* = 15.6 Hz, 1H), 4.67 (d, *J* = 16.0 Hz, 1H), 4.02 (d, *J* = 14.0 Hz, 1H), 3.48 (d, *J* = 14.0 Hz, 1H), 3.39 (s, 1H); ^{13}C NMR (100 MHz, $CDCl_3$): δ 177.4, 150.3, 142.2, 135.1, 134.2, 132.4, 129.9, 129.2, 128.8, 128.2, 127.6, 127.2, 124.9, 124.3, 123.3, 109.6, 76.4, 43.9, 40.0; HRMS (ESI) calcd. For $C_{22}H_{19}N_2O_4$ $[M+H]^+$: 375.1139, Found: 375.1136.

4-((1-benzyl-3-hydroxy-2-oxoindolin-3-yl)methyl)-3-nitrobenzonitrile

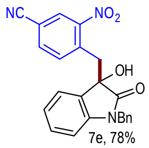

7e, 78%

White solid; m.p. 189–191 °C; ^1H (400 MHz, $CDCl_3$) δ 8.11 (s, 1H), 7.64 (d, *J* = 8.0 Hz, 1H), 7.55 (d, *J* = 8.4 Hz, 1H), 7.30-7.16 (m, 7H), 7.09-7.02 (m, 2H), 6.72 (d, *J* = 8.0 Hz, 1H), 4.89 (d, *J* = 16.8 Hz, 1H), 4.67 (d, *J* = 16.8 Hz, 1H), 3.98 (d, *J* = 14.4 Hz, 1H), 3.56 (d, *J* = 14.4 Hz, 1H), 3.12 (s, 1H); ^{13}C NMR (100 MHz, $CDCl_3$): δ 176.7, 150.2, 142.0, 136.7, 135.3, 135.0, 134.9, 134.6, 130.4, 128.9, 128.4, 128.0, 127.4, 124.1, 123.6, 116.4, 112.6, 109.8, 76.0, 44.0, 40.2; IR ν (cm^{-1}) 3379, 2237, 1705, 1535, 1346, 1192, 1080, 644; HRMS (ESI) calcd. For $C_{23}H_{17}N_3NaO_4$ $[M+Na]^+$: 422.1111, Found: 422.1119.

methyl 4-((1-benzyl-3-hydroxy-2-oxoindolin-3-yl)methyl)-3-nitrobenzoate

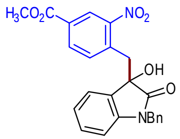

7f, 73%

White solid; m.p. 174–176 °C; ^1H (400 MHz, $CDCl_3$) δ 8.44 (s, 1H), 8.06-8.04 (dd, *J* = 8.0 Hz, 1.6 Hz, 1H), 7.50 (d, *J* = 8.0 Hz, 1H), 7.28-7.12 (m, 6H), 7.03-6.97 (m, 2H), 6.65 (d, *J* = 8.0 Hz, 1H), 4.89 (d, *J* = 15.6 Hz, 1H), 4.66 (d, *J* = 15.6 Hz, 1H), 4.00 (d, *J* = 13.6 Hz, 1H), 3.95 (s, 3H), 3.63 (s, 1H), 3.54 (d, *J* = 13.2 Hz, 1H); ^{13}C NMR (100 MHz, $CDCl_3$): δ 177.2, 164.9, 150.1, 142.1, 135.0, 134.5, 134.0, 132.7, 130.4, 130.1, 128.8, 128.6, 127.7, 127.2, 125.9, 124.2, 123.4, 109.7, 76.3, 52.6, 44.0, 40.1; HRMS (ESI) calcd. For $C_{24}H_{20}N_2NaO_6$ $[M+Na]^+$: 455.1214, Found: 455.1222.

3-(4-acetyl-2-nitrobenzyl)-1-benzyl-3-hydroxyindolin-2-one

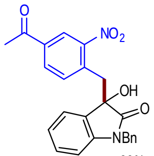

7g, 69%

White solid; m.p. 149–151 °C; ^1H (400 MHz, $CDCl_3$) δ 8.34 (s, 1H), 7.96 (d, *J* = 8.0 Hz, 1H), 7.53 (d, *J* = 8.0 Hz, 1H), 7.24-7.17 (m, 6H), 7.05-6.98 (m, 2H), 6.66 (d, *J* = 8.0 Hz, 1H), 4.90 (d, *J* = 15.6 Hz, 1H), 4.65 (d, *J* = 16.0 Hz, 1H), 3.99 (d, *J* = 13.2 Hz, 1H), 3.80 (s, 1H), 3.56 (d,

J = 13.2 Hz, 1H), 2.59 (s, 3H); ^{13}C NMR (100 MHz, $CDCl_3$): δ 195.5, 177.2, 150.3, 142.0, 136.7, 135.0, 134.7, 134.1, 131.3, 130.1, 128.7, 128.6, 127.7, 127.2, 124.6, 124.1, 123.5, 109.7, 76.3, 43.9, 40.0, 26.5; IR ν (cm^{-1}) 3279, 1694, 1535, 1362, 1258, 1177, 1080, 756; HRMS (ESI) calcd. For $C_{24}H_{20}N_2NaO_5$ $[M+Na]^+$: 439.1264, Found: 439.1268.

1-benzyl-3-hydroxy-3-(4-(hydroxymethyl)-2-nitrobenzyl)indolin-2-one

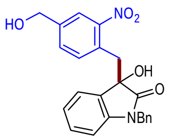

7h, 70%

White solid; m.p. 175–177 °C; ^{1}H (400 MHz, $CDCl_3$) δ 7.85 (s, 1H), 7.45 (d, J = 8.0 Hz, 1H), 7.39 (d, J = 8.0 Hz, 1H), 7.30-7.24 (m, 3H) 7.20-7.14 (m, 3H), 7.07-7.05 (m, 1H), 7.00-6.97 (m, 1H), 6.64 (d, J = 8.0 Hz, 1H), 4.90 (d, J = 15.6 Hz, 1H), 4.72 (d, J = 3.2 Hz, 2H), 4.67 (d, J = 16.0 Hz, 1H), 3.95 (d, J = 13.6 Hz, 1H), 3.95 (d, J = 14.0 Hz, 1H), 3.35 (s, 1H), 2.19 (s, 1H); ^{13}C NMR (100 MHz, $CDCl_3$): δ 177.4, 150.2, 142.1, 141.7, 135.2, 134.3, 130.4, 129.9, 128.9, 128.8, 128.1, 127.7, 127.3, 124.2, 123.3, 122.9, 109.6, 76.3, 63.6, 43.9, 39.8; IR ν (cm^{-1}) 3514, 3402, 1713, 1535, 1358, 1173, 999, 745; HRMS (ESI) calcd. For $C_{23}H_{20}N_2NaO_5$ $[M+Na]^+$: 427.1264, Found: 427.1266.

1-benzyl-3-(4,5-dimethoxy-2-nitrobenzyl)-3-hydroxyindolin-2-one

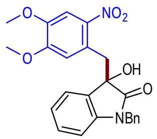

7i, 68%

White solid; m.p. 207–209 °C; ^{1}H (400 MHz, $CDCl_3$) δ 7.48 (s, 1H), 7.29-7.21 (m, 3H), 7.19-7.17 (m, 1H), 7.14-7.09 (m, 3H), 7.02-6.99 (m, 1H), 6.79 (s, 1H), 6.64 (d, J = 7.6 Hz, 1H), 4.95 (d, J = 16.0 Hz, 1H), 4.67 (d, J = 15.6 Hz, 1H), 4.08 (d, J = 13.6 Hz, 1H), 3.91 (s, 3H), 3.79 (s, 3H), 3,45 (d, J = 13.6 Hz, 1H), 3.32 (s, 1H); ^{13}C NMR (100 MHz, $CDCl_3$): δ 177.5, 152.2, 147.9, 142.5, 142.4, 135.2, 129.9, 129.0, 128.8, 127.7, 127.0, 124.4, 123.8, 123.1, 115.4, 109.6, 108.3, 77.2, 56.3, 56.2, 43.9, 40.2; IR ν (cm^{-1}) 3348, 2935, 1694, 1524, 1273, 1061, 799, 756; HRMS (ESI) calcd. For $C_{24}H_{22}N_2NaO_6$ $[M+Na]^+$: 457.1370, Found: 457.1369.

1-benzyl-3-hydroxy-3-(2-(4-nitrophenyl)propan-2-yl)indolin-2-one

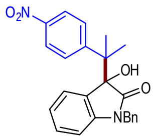

7j, 78%

White solid; m.p. 143–145 °C; ^{1}H (400 MHz, $CDCl_3$) δ 7.98 (d, J = 8.8 Hz, 2H), 7.37 (d, J = 8.4 Hz, 2H), 7.24 (t, J = 3.2 Hz, 3H), 7.18 (t, J = 7.8 Hz, 1H), 7.08 (d, J = 4.4 Hz, 2H), 6.91 (t, J = 7.6 Hz, 1H), 6.68 (d, J = 6.8 Hz, 1H), 6.62 (d, J = 8.0 Hz, 1H), 4.86 (d, J = 15.6 Hz, 1H), 4.49 (d, J = 15.6 Hz, 1H), 3.06 (s, 1H), 1.68 (s, 3H), 1.53 (s, 3H); ^{13}C NMR (100 MHz, $CDCl_3$): δ 177.3, 151.1, 146.5, 143.2, 135.1, 129.9, 128.9, 128.7, 128.4, 127.8, 127.4, 125.5, 122.4, 122.3, 109.1, 80.0, 45.0, 44.0, 22.8, 22.2; IR ν (cm^{-1}) 3433, 1694, 1512, 1346, 1177, 1111, 853, 702; HRMS (ESI) calcd. For $C_{24}H_{23}N_2O_4$ $[M+H]^+$: 403.1652, Found: 403.1661.

1-benzyl-5-chloro-3-hydroxy-3-(4-nitrobenzyl)indolin-2-one

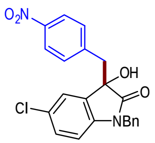
7k, 81%

White solid; m.p. 159–161 °C; ^{1}H (400 MHz, $CDCl_3$) δ 7.91 (d, *J* = 8.8 Hz, 2H), 7.37 (d, *J* = 2.0 Hz, 1H), 7.26-7.12 (m, 4H), 7.07 (d, *J* = 8.8 Hz, 2H), 6.82 (d, *J* = 8.8 Hz, 2H), 6.49 (d, *J* = 8.4 Hz, 1H), 4.89 (d, *J* = 16.0 Hz, 1H), 4.46 (d, *J* = 16.0 Hz, 1H), 4.00 (s, 1H), 3.47-3.39 (dd, *J* = 18.4 Hz, 12.4 Hz, 2H); ^{13}C NMR (100 MHz, $CDCl_3$): δ 176.9, 147.2, 141.0, 140.9, 134.3, 131.1, 130.3, 130.1, 128.9, 128.7, 128.0, 126.9, 124.8, 123.2, 110.8, 77.2, 44.4, 44.0; IR ν (cm^{-1}); 3460, 1721, 1609, 1520, 1350, 1061, 829, 737; HRMS (ESI) calcd. For $C_{22}H_{17}ClN_2NaO_4$ $[M+Na]^+$: 431.0769, Found: 431.0772.

1-benzyl-5-bromo-3-hydroxy-3-(4-nitrobenzyl)indolin-2-one

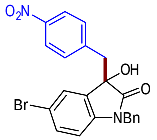
7l, 74%

White solid; m.p. 173–175 °C; ^{1}H (400 MHz, $CDCl_3$) δ 7.91 (d, *J* = 1.6 Hz, 2H), 7.50 (d, *J* = 0.8 Hz, 1H), 7.34-7.32 (dd, *J* = 8.4 Hz, 2.0 Hz, 1H), 7.21-7.12 (m, 3H), 7.07 (d, *J* = 8.4 Hz, 2H), 6.82 (d, *J* = 7.6 Hz, 2H), 6.45 (d, *J* = 8.4 Hz, 1H), 4.89 (d, *J* = 15.6 Hz, 1H), 4.46 (d, *J* = 15.6 Hz, 1H), 3.72 (s, 1H), 3.46-3.38 (dd, *J* = 20.0 Hz, 12.8 Hz, 2H); ^{13}C NMR (100 MHz, $CDCl_3$): δ 176.6, 147.2, 141.5, 140.9, 134.3, 133.1, 131.1, 130.6, 128.7, 128.0, 127.6, 126.9, 123.2, 116.1, 111.2, 77.1, 44.5, 44.0; HRMS (ESI) calcd. For $C_{22}H_{17}BrN_2NaO_4$ $[M+Na]^+$: 475.0264, Found: 475.0268.

1,3-dibenzyl-3-hydroxy-5-(trifluoromethyl)indolin-2-one

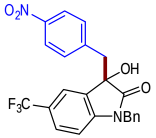
7m, 81%

White solid; m.p. 136–138 °C; ^{1}H (400 MHz, $CDCl_3$) δ 7.90 (d, *J* = 8.4 Hz, 2H), 7.62 (s, 1H), 7.50 (d, *J* = 8.0 Hz, 1H), 7.24-7.14 (m, 3H), 7.03 (d, *J* = 8.8 Hz, 2H), 6.86 (d, *J* = 7.6 Hz, 2H), 6.67 (d, *J* = 8.0 Hz, 1H), 4.93 (d, *J* = 15.6 Hz, 1H), 4.52 (d, *J* = 15.6 Hz, 1H), 4.31 (s, 1H), 3.47-3.39 (dd, *J* = 18.4 Hz, 12.4 Hz, 2H); ^{13}C NMR (100 MHz, $CDCl_3$): δ 177.5, 147.2, 145.3, 140.7, 134.0, 131.1, 129.4, 128.8, 128.1-127.8 (q, *J* = 15.04 Hz), 127.0, 125.9, 125.6, 125.3, 123.2, 122.6, 121.6 (d, *J* = 11.28 Hz), 109.6, 76.9, 44.4, 44.1; IR ν (cm^{-1}) 3341, 1709, 1520, 1346, 1165, 1119, 833, 698; HRMS (ESI) calcd. For $C_{23}H_{17}F_3N_2NaO_4$ $[M+Na]^+$: 465.1033, Found: 465.1023.

1,3-dibenzyl-3-hydroxy-5-(trifluoromethoxy)indolin-2-one

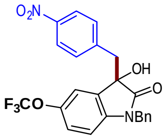
7n, 76%

White solid; m.p. 147–149 °C; ^{1}H (400 MHz, $CDCl_3$) δ 7.91 (d, *J* = 8.4 Hz, 2H), 7.25-7.16 (m, 4H), 7.06 (d, *J* = 8.4 Hz, 3H), 6.88 (d, *J* = 7.2 Hz, 2H), 6.57 (d, *J* = 8.4 Hz, 1H), 4.92 (d, *J* = 15.6 Hz, 1H), 4.51 (d, *J* = 16.0 Hz, 1H), 4.03 (s, 1H), 3.41 (dd, *J* = 29.6 Hz, 12.8 Hz, 2H); ^{13}C NMR (100 MHz, $CDCl_3$): δ 177.3 (d, *J* = 15.0 Hz), 147.2, 145.1, 140.9 (d, *J* = 33.8 Hz), 134.3, 131.1, 130.1, 128.8, 128.1, 127.0, 123.2 (d, *J* = 26.3 Hz), 121.8, 119.2, 118.4, 116.7, 110.4, 77.3, 44.1, 44.5; IR ν (cm^{-1}) 3352, 1717, 1520, 1350, 1215, 1150, 817, 698; HRMS (ESI) calcd. For $C_{23}H_{18}F_3N_2O_5$ $[M+H]^+$: 459.1162, Found: 459.1147.

1-benzyl-3-hydroxy-5-methoxy-3-(4-nitrobenzyl)indolin-2-one

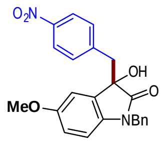

7o, 78%

White solid; m.p. 206–208 °C; ^{1}H (400 MHz, $CDCl_3$) δ 7.91 (d, *J* = 8.8 Hz, 2H), 7.20-7.07 (m, 5H), 6.97 (d, *J* = 2.4 Hz, 1H), 6.84 (d, *J* = 7.2 Hz, 2H), 6.72 (dd, *J* = 8.4 Hz, 2.4 Hz, 1H), 6.47 (d, *J* = 8.4 Hz, 1H), 4.89 (d, *J* = 15.6 Hz, 1H), 4.45 (d, *J* = 15.6 Hz, 1H), 3.77 (s, 3H), 3.41 (m, 3H); ^{13}C NMR (100 MHz, $CDCl_3$): δ 176.8, 156.4, 147.1, 141.5, 135.8, 134.9, 131.2, 129.7, 128.6, 127.8, 127.0, 123.1, 114.6, 111.5, 110.3, 77.4, 55.8, 44.6, 44.0; IR ν (cm^{-1}) 3298, 1709, 1601, 1516, 1342, 1277, 1015, 694; HRMS (ESI) calcd. For $C_{23}H_{20}N_2NaO_5$ $[M+Na]^+$: 427.1264, Found: 427.1266.

1-benzyl-3-hydroxy-5-methyl-3-(4-nitrobenzyl)indolin-2-one

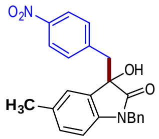

7p, 80%

White solid; m.p. 205–207 °C; ^{1}H (400 MHz, $CDCl_3$) δ 7.89 (d, *J* = 8.4 Hz, 2H), 7.20-7.11 (m, 4H), 7.06-7.00 (m, 3H), 6.83 (d, *J* = 7.2 Hz, 2H), 6.46 (d, *J* = 8.0 Hz, 1H), 4.90 (d, *J* = 16.0 Hz, 1H), 4.45 (d, *J* = 15.6 Hz, 1H), 3.63 (s, 1H), 3.47-3.37 (dd, *J* = 27.6 Hz, 12.8 Hz, 2H), 2.34 (s, 3H); ^{13}C NMR (100 MHz, $CDCl_3$): δ 177.0, 147.0, 141.6, 140.1, 134.9, 133.0, 131.1, 130.5, 128.6, 128.5, 127.7, 127.0, 125.0, 123.0, 109.5, 77.2, 44.5, 43.9, 21; IR ν (cm^{-1}) 3545, 1724, 1497, 1346, 1280, 1049, 818, 698; HRMS (ESI) calcd. For $C_{23}H_{20}N_2NaO_4$ $[M+Na]^+$: 411.1315, Found: 411.1318.

1-benzyl-7-chloro-3-hydroxy-3-(4-nitrobenzyl)indolin-2-one

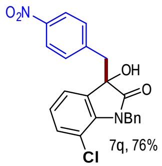

7q, 76%

White solid; m.p. 156–158 °C; ^{1}H (400 MHz, $CDCl_3$) δ 7.90 (d, *J* = 8.8 Hz, 2H), 7.26-7.12 (m, 5H), 7.08-7.03 (m, 3H) 6.85 (d, *J* = 7.2 Hz, 2H), 5.10 (dd, *J* = 26.0 Hz, 16.0 Hz, 2H), 4.24 (s, 1H) 3.45-3.34 (dd, *J* = 31.2 Hz, 12.5 Hz, 2H); ^{13}C NMR (100 MHz, $CDCl_3$): δ 178.1, 147.1, 141.0,138.5, 136.4, 132.8, 131.6, 131.2, 128.4, 127.3, 126.3, 124.3, 123.2, 123.1, 116.0, 76.3, 44.9, 44.4; IR ν (cm^{-1}) 3395, 1709, 1520, 1454, 1346, 1161, 1065, 737; HRMS (ESI) calcd. For $C_{22}H_{17}ClN_2NaO_4$ $[M+Na]^+$: 431.0769, Found: 431.0768.

1-benzyl-3-hydroxy-7-methyl-3-(4-nitrobenzyl)indolin-2-one

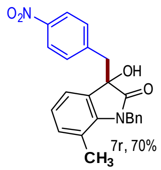

7r, 70%

White solid; m.p. 205–207 °C; ^{1}H (400 MHz, $CDCl_3$) δ 7.95 (d, *J* = 8.8 Hz, 2H), 7.25 (d, *J* = 5.6 Hz, 1H), 7.17-6.79 (m, 7H), 6.66 (d, *J* = 7.2 Hz, 2H), 5.10 (d, *J* = 16.8 Hz, 1H), 4.86 (d, *J* = 16.8 Hz, 1H), 4.23 (s, 1H), 3.47-3.37 (dd, *J* = 26.6 Hz, 12.8 Hz, 2H), 2.08 (s, 3H); ^{13}C NMR (100 MHz, $CDCl_3$): δ 178.4, 147.1, 141.8, 140.5, 136.6, 134.2, 131.4, 129.4, 128.6, 127.3, 125.2, 123.5, 123.1, 122.3, 120.5, 76.7, 45.0, 44.5, 18.4; IR ν (cm^{-1}) 3395, 1701, 1520, 1346, 1184, 1065, 1030, 741; HRMS (ESI) calcd. For $C_{23}H_{20}N_2NaO_4$ $[M+Na]^+$: 411.1315, Found: 411.1318.

1-benzyl-4,7-dichloro-3-hydroxy-3-(4-nitrobenzyl)indolin-2-one

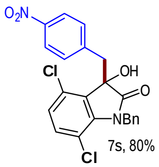

7s, 80%

White solid; m.p. 185–187 °C; ^{1}H (400 MHz, $CDCl_3$) δ 7.86 (d, J = 8.4 Hz, 2H), 7.21-7.18 (m, 1H), 7.14-7.10 (m, 3H), 7.06-7.03 (m, 3H), 6.75 (d, J = 7.6 Hz, 2H), 5.18-5.05 (dd, J = 37.6 Hz, 16.2 Hz, 2H), 4.00 (d, J = 12.4 Hz, 1H), 3.81 (s, 1H), 3.48 (d, J = 12.8 Hz, 1H); ^{13}C NMR (100 MHz, $CDCl_3$): δ 176.7, 147.2, 140.8, 140.4, 136.1, 133.8, 130.6, 130.5, 128.4, 127.5, 127.3, 126.2, 125.3, 123.4, 114.6, 77.6, 44.9, 41.1; HRMS (ESI) calcd. For $C_{22}H_{16}Cl_2N_2NaO_4$ $[M+Na]^+$: 465.0379, Found: 465.0370.

3-hydroxy-1-methyl-3-(4-nitrobenzyl)indolin-2-one

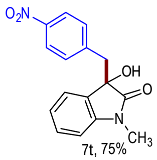

7t, 75%

White solid; m.p. 147–149 °C; ^{1}H (400 MHz, $CDCl_3$) δ 7.98 (d, J = 8.8 Hz, 2H), 7.32-7.27 (m, 1H), 7.19-7.07 (m, 4H), 6.69 (d, J = 8.0 Hz, 1H), 3.84 (s, 1H), 3.42 (d, J = 12.8 Hz, 1H), 3.26 (d, J = 12.8 Hz, 1H), 3.03 (s, 3H); ^{13}C NMR (100 MHz, $CDCl_3$): δ 177.4, 147.0, 142.9, 141.9, 131.1, 130.1, 128.5, 124.3, 123.2, 122.9, 108.6, 77.2, 44.5, 26.1; IR ν (cm^{-1}) 3364, 1701, 1516, 1342, 1223, 1103, 1011, 756; HRMS (ESI) calcd. For $C_{16}H_{15}N_2O_4$ $[M+H]^+$: 299.1026, Found: 299.1026.

ethyl 3-(4-nitrobenzyl)-2,3-dihydrobenzo[d]isothiazole-3-carboxylate 1,1-dioxide

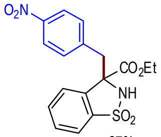

9a, 67%

White solide; m.p. 165–167 °C; ^{1}H (400 MHz, $CDCl_3$) δ 8.16 (d, J = 8.4 Hz, 2H), 7.88 (d, J = 8.0Hz, 1H), 7.80 (d, J = 8.0 Hz, 1H), 7.55 (t, J = 7.6 Hz, 1H), 7.65 (t, J = 7.6 Hz, 2H), 7.50 (d, J = 8.4 Hz, 2H), 5.64 (s, 1H), 4.34-4.25 (m, 2H), 3.63 (d, J = 13.2 Hz, 1H), 3.33 (d, J = 13.6 Hz, 1H), 1.33 (t, J = 7.2 Hz, 3H); ^{13}C NMR (100 MHz, $CDCl_3$): δ 69.1, 64.0, 45.4, 14.1, 168.5, 147.5, 141.9, 137.9, 135.8, 133.7, 131.6, 130.9, 124.7, 123.4, 121.8; IR ν (cm^{-1}) 3535, 2283, 1759, 1559, 1327, 1283, 1168, 853; HRMS (ESI) calcd. For $C_{17}H_{16}N_2NaO_6S$ $[M+Na]^+$: 399.0621, Found: 399.0623.

ethyl 3-(3-chloro-4-nitrobenzyl)-2,3-dihydrobenzo[d]isothiazole-3-carboxylate 1,1-dioxide

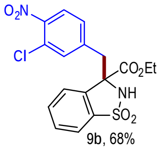

9b, 68%

White solide; m.p. 131–133 °C; ^{1}H (400 MHz, $CDCl_3$) δ 7.86-7.81 (m, 3H), 7.76-7.72 (m, 1H), 7.68-7.64 (m, 1H), 7.53 (d, J = 1.6 Hz), 7.42-7.40 (dd, J = 8.2 Hz, 1.8 Hz, 1H), 5.67 (s, 1H), 4.37-4.29 (m, 2H), 3.56 (d, J = 13.6 Hz, 1H), 3.27 (d, J = 13.6 Hz, 1H), 1.36 (t, J = 7.2 Hz, 3H); ^{13}C NMR (100 MHz, $CDCl_3$): δ 168.3, 147.1, 140.9, 137.9, 135.9, 133.8, 131.0, 130.0, 127.0, 125.5, 124.6, 121.8, 68.9, 64.2, 44.8, 14.2; IR ν (cm^{-1}) 3507, 1709, 1535, 1304, 1165, 1030, 984, 856; HRMS (ESI) calcd. For $C_{17}H_{15}ClN_2NaO_6S$ $[M+Na]^+$: 433.0232, Found: 433.0229.

ethyl3-(4-cyano-2-nitrobenzyl)-2,3-dihydrobenzo[d]isothiazole-3-carboxylate 1,1-dioxide

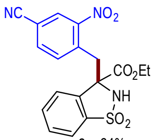

9c, 64%

White solide; m.p. 162–164 °C; ^{1}H (400 MHz, $CDCl_3$) δ 8.13 (d, *J* = 1.6 Hz, 1H), 7.84-7.82 (m, 1H), 7.77-7.73 (m, 3H), 7.67-7.61 (m, 2H), 5.72 (s, 1H), 4.38-4.18 (m, 3H), 3.64 (d, *J* = 14.4 Hz, 1H), 1.33 (t, *J* = 7.0 Hz, 3H); ^{13}C NMR (100 MHz, $CDCl_3$): δ 168.5, 150.6, 136.7, 135.6, 135.2, 135.0, 133.9, 133.6, 131.2, 128.4, 124.9, 121.8, 116.3, 113.1, 68.5, 64.6, 40.2, 13.8; IR ν (cm^{-1}) 3507, 2237, 1740, 1535, 1308, 1234, 1165, 853; HRMS (ESI) calcd. For $C_{18}H_{16}N_3O_6S$ [M+H]$^+$: 402.0754, Found: 402.0760.

ethyl3-(4-(methoxycarbonyl)-2-nitrobenzyl)-2,3-dihydrobenzo[d]isothiazole-3-carboxylate 1,1-dioxide

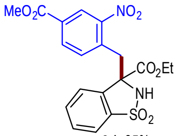

9d, 65%

White solide; m.p. 206–208 °C; ^{1}H (400 MHz, $CDCl_3$) δ 8.48 (d, *J* = 2.0 Hz, 1H), 8.12-8.10 (dd, *J* = 8.0 Hz, 1.6 Hz, 1H), 7.85-7.82 (m, 1H), 7.73 (t, *J* = 7.4 Hz, 2H), 7.65-7.63 (m, 1H), 7.58 (d, *J* = 8.0 Hz, 1H), 5.71 (s, 1H), 4.35-4.15 (m, 3H), 3.95 (s, 3H), 3.62 (d, *J* = 14.4 Hz, 1H), 1.31 Hz (t, *J* = 7.0 Hz, 3H); ^{13}C NMR (100 MHz, $CDCl_3$): δ 168.7, 164.7, 150.5, 137.0, 135.6, 134.2, 133.8, 133.0, 132.7, 131.0, 130.9, 125.9, 125.1, 121.7, 68.7, 64.5, 52.7, 40.3, 13.8; IR ν (cm^{-1}) 3507, 1721, 1535, 1296, 1273, 1184, 1107, 764; HRMS (ESI) calcd. For $C_{19}H_{18}N_2NaO_8S$ [M+Na]$^+$: 457.0676, Found: 457.0622.

ethyl 3-(4-acetyl-2-nitrobenzyl)-2,3-dihydrobenzo[d]isothiazole-3-carboxylate 1,1-dioxide

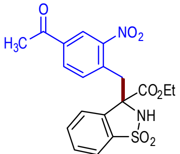

9e, 61%

Colourless liquid; ^{1}H (400 MHz, $CDCl_3$) δ 8.38 (s, 1H), 8.04-8.02 (dd, *J* = 8.0 Hz, 1.6 Hz, 1H), 7.84 (d, *J* = 8.0, 1H), 7.74 (t, *J* = 7.4 Hz, 2H), 7.66-7.61 (dd, *J* = 12.4 Hz, 8.0 Hz, 2H), 5.71 (s, 1H), 4.36-4.16 (m, 3H), 3.62 (d, *J* = 14.0 Hz, 1H), 2.63 (s, 3H), 1.32 (t, *J* = 7.0 Hz, 3H); ^{13}C NMR (100 MHz, $CDCl_3$): δ 195.3, 168.7, 150.7, 137.2, 137.1, 135.6, 134.5, 133.8, 133.1, 131.3, 131.1, 125.1, 124.5, 121.7,68.7, 64.5, 40.3, 26.6, 13.8; IR ν (cm^{-1}) 3507, 1709, 1535, 1304, 1165, 1030, 856, 571; HRMS (ESI) calcd. For $C_{19}H_{18}N_2NaO_7S$ [M+Na]$^+$: 441.0727, Found: 441.0732.

ethyl 3-(4-(hydroxymethyl)-2-nitrobenzyl)-2,3-dihydrobenzo[d]isothiazole-3-carboxylate 1,1-dioxide

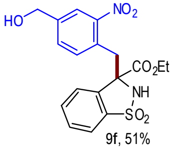

9f, 51%

Colourless liquid; ^{1}H (400 MHz, $CDCl_3$) δ 7.86-7.83 (m, 2H), 7.74-7.70 (m, 2H), 7.64-7.60 (m, 1H), 7.46 (s, 2H), 5.71 (s, 1H), 4.75 (d, *J* = 2.8 Hz, 2H), 4.34-4.14 (m, 3H), 3.54 (d, *J* = 14.4 Hz, 1H), 2.02 (s, 1H), 1.31 (t, *J* = 7.0 Hz); ^{13}C NMR (100 MHz, $CDCl_3$): δ 169.0, 150.6, 142.3, 137.3, 135.5, 134.0, 133.7, 130.9, 130.3, 127.3, 125.2, 122.8, 121.6, 69.0, 64.3, 63.6, 40.1, 13.8; IR ν (cm^{-1}) 3310, 3078, 1721, 1535, 1296, 1184, 1030, 764; HRMS (ESI) calcd. For $C_{18}H_{18}N_2NaO_7S$ [M+Na]$^+$: 429.0727, Found: 429.0719.

ethyl 3-(4,5-dimethoxy-2-nitrobenzyl)-2,3-dihydrobenzo[d]isothiazole-3-carboxylate 1,1-dioxide

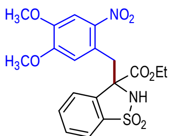

9g, 60%

Colourless liquid; ^1H (400 MHz, $CDCl_3$) δ 7.87 (d, J = 8.0 Hz, 1H), 7.76-7.70 (m, 2H), 7.64-7.60 (m, 1H), 7.46 (s, 1H), 6.90 (s, 1H), 5.78 (s, 1H), 4.36 (d, J = 14.4 Hz, 1H), 4.32-4.15 (m, 2H), 3.92 (s, 3H), 3.87 (s, 3H), 3.49 (d, J = 14.4 Hz, 1H), 1.32 (t, J = 7.2 Hz, 3H); ^{13}C NMR (100 MHz, $CDCl_3$): δ 169.2, 152.2, 148.2, 142.6, 137.7, 135.6, 133.5, 130.7, 125.5, 123.2, 121.6, 115.3, 108.1, 69.0, 64.2, 56.4, 56.3, 40.5, 13.8; IR ν (cm^{-1}) 3507, 2237, 1740, 1535, 1308, 1234, 1165, 853; HRMS (ESI) calcd. For $C_{19}H_{20}N_2NaO_8S$ $[M+Na]^+$: 459.0833, Found: 459.0839.

ethyl 3-(2-(4-nitrophenyl)propan-2-yl)-2,3-dihydrobenzo[d]isothiazole-3-carboxylate 1,1-dioxide

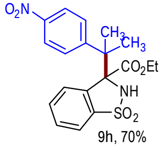

9h, 70%

Colourless liquid; ^1H (400 MHz, $CDCl_3$) δ 8.13 (d, J = 8.8 Hz, 2H), 7.84-7.82 (m, 1H), 7.77-7.74 (m, 1H), 7.70-7.63 (m, 2H), 7.51-7.49 (d, J = 9.2 Hz, 2H), 5.69 (s, 1H), 4.21-4.16 (dd, J = 14.4 Hz, 7.2 Hz, 2H), 1.74 (s, 3H), 1.49 (s, 3H), 1.23 (t, J = 7.2 Hz, 3H); ^{13}C NMR (100 MHz, $CDCl_3$): δ 168.5, 150.6, 146.9, 136.4, 134.5, 132.6, 130.8, 129.1, 127.7, 122.6, 121.7, 74.7, 63.7, 47.6, 25.1, 25.0, 13.9; IR ν (cm^{-1}) 3287, 1728, 1520, 1350, 1254, 1177, 1034, 756; HRMS (ESI) calcd. For $C_{19}H_{20}N_2NaO_6S$ $[M+Na]^+$: 427.0934, Found: 427.0940.

ethyl 5-methyl-3-(4-nitrobenzyl)-2,3-dihydrobenzo[d]isothiazole-3-carboxylate 1,1-dioxide

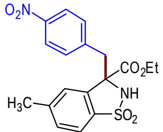

9i, 65%

White solide; m.p. 181–183 °C; ^1H (400 MHz, $CDCl_3$) δ 8.17-8.15 (dd (J = 6.8 Hz, 2.0 Hz, 2H), 7.69-7.4 (m, 2H), 7.51 (d, J = 8.8 Hz, 2H), 7.44 (d, J = 7.6 Hz, 1H), 5.60 (s, 1H), 4.33-4.24 (m, 2H), 3.62 (d, J = 13.6 Hz, 1H), 3.31 (d, J = 13.6 Hz, 1H), 2.54 (s, 3H), 1.32 (t, J = 7.2 Hz, 3H); ^{13}C NMR (100 MHz, $CDCl_3$): δ 168.5, 147.5, 144.9, 142.0, 138.5, 133.2, 131.8, 131.6, 124.9, 123.4, 121.5, 69.0, 63.9, 45.4, 22.0, 14.1; IR ν (cm^{-1}) 3456, 1736, 1601, 1524, 1346, 1184, 1053, 698; HRMS (ESI) calcd. For $C_{18}H_{18}N_2NaO_6S$ $[M+Na]^+$: 413.0778, Found: 413.0786.

ethyl 5-methoxy-3-(4-nitrobenzyl)-2,3-dihydrobenzo[d]isothiazole-3-carboxylate 1,1-dioxide

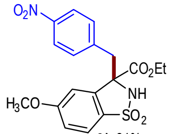

9j, 61%

White solid; m.p. 166–168 °C; ^1H (400 MHz, $CDCl_3$) δ 8.16 (d, J = 8.4 Hz, 2H), 7.70 (d, J = 8.8 Hz, 1H), 7.51 (d, J = 8.8 Hz, 2H), 7.28 (d, J = 2.0 Hz, 2H), 7.13-7.11 (dd, J = 8.4 Hz, 2.0 Hz, 1H), 5.62 (s, 1H), 4.35-4.23 (m, 2H), 3.94 (s, 3H), 3.60 (d, J = 13.2 Hz, 1H), 3.32 (d, J = 13.6 Hz, 1H), 1.33 (t, J = 7.2 Hz, 3H); ^{13}C NMR (100 MHz, $CDCl_3$): δ 168.4, 164.0, 147.5, 141.9, 140.7, 131.6, 127.8, 123.4, 123.2, 117.0, 109.4, 68.7, 63.9, 56.1, 45.4, 14.1; IR ν (cm^{-1}) 3507, 1740, 1589, 1520, 1346, 1180, 1015, 745; HRMS (ESI) calcd. For $C_{18}H_{18}N_2NaO_7S$ $[M+Na]^+$: 429.0727, Found: 429.0732.

(E)-3-hydroxy-3-styryl-3,4-dihydroquinolin-2(1H)-one

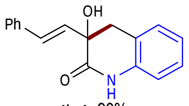

4b-1, 90%

White solid; m.p. 185–187 °C; ^{1}H (400 MHz, $CDCl_3$) δ 8.66 (d, *J* = 8.0 Hz, 1H), 7.26-7.18 (m, 7H), 7.06-7.02 (m, 1H), 6.87-6.79 (m, 2H), 6.17 (d, *J* = 8.0 Hz, 1H), 4.17 (s, 1H), 3.34 (d, *J* = 7.2 Hz, 1H), 3.18 (d, *J* = 7.6 Hz, 1H); ^{13}C NMR (100 MHz, $CDCl_3$): δ 172.8, 136.0, 131.2, 129.0, 128.5, 128.0, 128.0, 127.6, 126.8, 124.0, 122.4, 115.6, 72.1, 38.9; IR ν (cm^{-1}) 3325, 1686, 1489, 1366, 1315, 1150, 1088, 748; HRMS (ESI) calcd. For $C_{17}H_{15}NNaO_2$ $[M+Na]^+$: 288.0995, Found: 288.0987.

methyl 2-hydroxy-3-(4-nitrophenyl)-2-(3-phenyloxiran-2-yl)propanoate

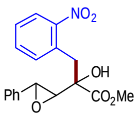

4b-2, 87%

Colourless liquid; ^{1}H (400 MHz, $CDCl_3$) δ 7.82-7.80 (m, 1H), 7.46-7.35 (m, 3H), 7.33-7.29 (m, 3H), 7.10-7.08 (m, 2H), 3.84 (s, 3H), 3.74 (d, *J* = 2.0 Hz, 1H), 3.63 (s, 2H), 3.43 (d, *J* = 20 Hz, 1H), 3.22 (s, 1H); ^{13}C NMR (100 MHz, $CDCl_3$): δ 173.2, 150.9, 135.8, 133.2, 132.3, 129.3, 128.5, 128.4, 128.1, 125.7, 124.7, 75.3, 64.4, 54.6, 53.5, 36.5; HRMS (ESI) calcd. For $C_{18}H_{17}NNaO_6$ $[M+Na]^+$: 366.0948, Found: 366.0945.

(E)-methyl 4-hydroxy-2-(4-nitrobenzyl)-4-phenylbut-2-enoate

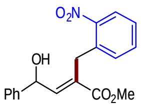

4b-3, 83%

Colourless liquid; ^{1}H (400 MHz, $CDCl_3$) δ 7.91-7.88 (dd, *J* = 8.0 Hz, 1.2 Hz, 1H), 7.44-7.40 (m, 1H), 7.36-7.28 (m, 6H), 7.17-7.14 (m, 2H), 5.55 (d, *J* = 8.8 Hz, 1H), 4.19 (d, *J* = 16.4, 1H), 4.07 (d, *J* = 16.8 Hz, 1H), 3.64 (s, 3H), 2.24 (s, 1H); ^{13}C NMR (100 MHz, $CDCl_3$): δ 167.2, 149.4, 145.0, 141.4, 133.5, 132.9, 130.5, 129.1, 128.9, 128.4, 127.3, 126.3, 124.6, 71.0, 52.2, 29.6; IR ν (cm^{-1}) 3464, 1717, 1524, 1350, 1219, 1115, 1015, 702; HRMS (ESI) calcd. For $C_{18}H_{17}NNaO_5$ $[M+Na]^+$: 350.0999, Found: 350.0997.

6H-indolo[2,3-b]quinoline

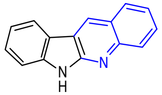

7u-1, 85%

^{1}H (400 MHz, DMSO) δ 11.7 (s, 1H), 9.05 (s, 1H), 8.26 (d, *J* = 7.6 Hz, 1H), 8.11 (d, *J* = 7.6 Hz, 1H), 7.98 (d, *J* = 8.4 Hz, 1H), 7.74-770 (m, 1H), 7.55-7.46 (m, 3H), 7.29-7.25 (m, 1H); ^{13}C NMR (100 MHz, DMSO): δ 152.9, 146.3, 141.5, 128.6, 128.2, 127.5, 127.0, 123.7, 122.7, 121.8, 120.3, 119.6, 117.9, 110.9.

5-methyl-5H-indolo[2,3-b]quinoline[10]

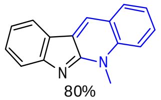

80%
neocryptolepine

^{1}H (400 MHz, $CDCl_3$) δ 8.54 (s, 1H), 8.06 (d, *J* = 7.6 Hz, 1H), 8.00 (d, *J* = 8.0 Hz, 1H), 7.78-7.74 (m, 3H), 7.55 (t, *J* = 7.6 Hz, 1H), 7.47-7.43 (m, 1H), 7.23 (d, *J* = 7.2 Hz, 1H), 4.38 (s, 3H); ^{13}C NMR (100 MHz, $CDCl_3$): δ 156.3, 155.5, 137.0, 130.4, 130.0, 129.3, 128.2, 128.1, 124.0, 121.9, 121.0, 120.8, 119.8, 117.7, 114.1, 33.0.

2,2,6,6-tetramethyl-1-((4-nitrobenzyl)oxy)piperidine

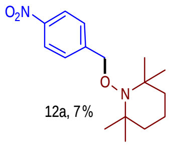

12a, 7%

White solid; m.p. 60–62 °C; ^{1}H (400 MHz, $CDCl_3$) δ 8.21-8.19 (dd, J = 7.0 Hz, 1.8 Hz, 2H), 7.50 (d, J = 8.8 Hz, 2H), 4.93 (s, 2H), 1.56-1.47 (m, 5H), 1.38-1.35 (m, 1H), 1.21 (s, 6H), 1.17 (s, 6H); ^{13}C NMR (100 MHz, $CDCl_3$): δ 147.1, 146.0, 127.4, 123.5, 60.1, 39.7, 33.0, 20.3, 17.0; IR ν (cm^{-1}) 3483, 2936, 1601, 1516, 1342, 1011, 841, 737; HRMS (ESI) calcd. For $C_{16}H_{24}N_2NaO_3$ $[M+Na]^+$: 315.1679, Found: 315.16793.

Supplementary references

1. Kammari, L., šolomek, T., Ngoy, B. P., Heger, D. & Klán, P. Orthogonal photocleavageof a monochromophoric linker. *J. Am. Chem. Soc*. 132, 11431-11433 (2010)..
2. Rajeshwaran, G. G. *et al*. Gold-catalyzed highly efficient access to 3(2H)-furanones from 2-Oxo-3-butynoates and related compounds. *Org. Lett*. 13, 1270-1273 (2006).
3. Kong, C., Jana, N. & Driver, T. G. Rh_2(II)-catalyzed selective aminomethylene migration fromstyryl azides. *Org. Lett*. 15, 824-827 (2013).
4. Ouyang, B. *et al*. The asymmetric Cu(II)-indolinyl methanol complex catalyzed Diels-Alder reaction of 2-vinylindoles with β,γ-unsaturated α-ketoesters: an efficient route to functionalized tetrahydrocarbazoles. *Org. Biomol. Chem.* 12, 4172-4176 (2014).
5. Horwitz, M. A. *et al*. Asymmetric organocatalytic reductive coupling reactions between benzylidene pyruvates and aldehydes. *Org. Lett*. 18, 36-39 (2016).
6. Guo, M., Li, D. & Zhang, Z. Novel synthesis of 2-oxo-3-butynoates by copper-catalyzed cross-coupling reaction of terminal alkynes and monooxalyl chloride. *J. Org. Chem.* 68, 10172-10174 (2003).
7. Dhara, K. *et al*. Synthesis of carbazole alkaloids by ring-closing metathesis and ring rearrangement–aromatization. *Angew. Chem. Int. Ed.* 54, 15831-15835 (2015).
8. Liu, Y.-L. *et al*. Organocatalytic asymmetric synthesis of substituted 3-hydroxy-2-oxindoles via morita-baylis-hillman reaction. *J. Am. Chem. Soc*. 132, 15176-15178 (2010).
9. Wang, H. *et al*. Simple Branched Sulfur–Olefins as Chiral ligands for Rh-catalyzed asymmetric arylation of cyclic ketimines: highly enantioselective construction of tetrasubstituted carbon stereocenters. *J. Am. Chem. Soc.*, 135, 971-974 (2013).
10. Basavaiah, D. & Reddy, D. M. Baylis–Hillman acetates in organic synthesis: convenient one-pot synthesis of α-carboline framework – a concise synthesis of neocryptolepine. *Org. Biomol. Chem*., 10, 8774-8777 (2012).
